# Integration of intragraft transcriptomics and urinary cytokines identifies CXCL10 and FasL signature in subclinical acute rejection

**DOI:** 10.1038/s41598-026-35923-6

**Published:** 2026-02-19

**Authors:** Sharon Natasha Cox, Samantha Chiurlia, Emanuela Pasculli, Luigi Biancone, Davide Diena, Vincenzo Cantaluppi, Andrea Airoldi, Ilaria Gandolfini, Umberto Maggiore, Nicola Bossini, Michele Rossini, Graziano Pesole, Francesco Paolo Schena, Italian SCAR Study Group, Luigi Biancone, Davide Diena, Vincenzo Cantaluppi, Andrea Airoldi, Michele Rossini, Francesco Paolo Schena, Paolo Rigotti, Giovanni Zaninotto, Franco Citterio, Alessia Toscano, Regina Tardanico

**Affiliations:** 1 Department of Biosciences, Biotechnology and Environment, University of Bari Aldo Moro, Bari, Italy; 2 University Hospital Consorziale Policlinico. Piazzale Giulio Cesare 11, 70124 Bari, Italy; 3 Schena Foundation, Bari, Italy; 4 Department of Medical Sciences, University of Turin, 10126 Turin, Italy; 5 Nephrology and Kidney Transplantation Unit, Department of Translational Medicine (DIMET), University of Piemonte Orientale (UPO), Maggiore della Carità University Hospital, Novara, Italy; 6 Nephrology Unit, University Hospital of Parma, Parma, Italy; 7 Division of Nephrology and Dialysis, ASST Spedali Civili, Brescia, Italy; 8 Nephrology, Dialysis and Transplantation Unit, DiMePRe-J, University of Bari Aldo Moro, Bari, Italy; 9 Kidney Pancreas Transplant Unit, Department of Surgery, Oncology and Gastroenterology, Padua University Hospital, Padua, Italy; 10 Department of Surgery and Cancer, Imperial College London, London, UK; 11 Renal Transplantation Unit, Department of Surgery, Catholic University of the Sacred Heart, Rome, Italy

**Keywords:** Kidney transplantation, Subclinical acute rejection, CXCL10, FasL, Kidney biopsy, Renal inflammation, Biomarkers, Diseases, Immunology, Medical research, Nephrology

## Abstract

Subclinical acute rejection (SCAR) in kidney transplantation, defined by histologic lesions without clinical dysfunction, remains a major cause of allograft injury and is currently detectable only by invasive protocol biopsies. We conducted a multicenter study in which transcriptomic profiling of Formalin-Fixed, Paraffin-Embedded biopsies from SCAR and control patients revealed a distinct signature with upregulation of *NFKBIZ, TNFSF14, SLAMF8*, and *CD247*, validated by qRT-PCR and immunohistochemistry but not detectable in urine. Focusing on secreted cytokines, CXCL10 and FasL emerged as candidate urinary biomarkers and were first measured in 12 SCAR patients and 12 controls, showing a significant increase in SCAR. Validation in an independent cohort of 86 kidney transplant recipients, after excluding patients with confounders, confirmed higher CXCL10 and FasL levels in SCAR. When combined as a composite biomarker signature (CXCL10 + FasL), ROC analysis yielded an AUC of 0.711 (95% CI, 0.549–0.874), with 50% sensitivity and 84% specificity at the optimal cutoff. In the still poorly studied context of SCAR, this work is a proof-of-concept approach linking intragraft transcriptomics to urinary cytokine levels. Our findings support the utility of urinary CXCL10 and FasL in assisting clinicians in identifying patients who may benefit from further evaluation, including consideration of a graft biopsy, thereby contributing to improved long-term allograft outcomes.

**Supplementary Information:**

The online version contains supplementary material available at 10.1038/s41598-026-35923-6.

## Introduction

Subclinical acute rejection (SCAR) in kidney transplantation is characterized by the presence of histologic lesions of acute rejection in allograft biopsy and the absence of clinical and laboratory signs of renal dysfunction^1^. SCAR is an active immunological process that can lead to glomerular damage, interstitial fibrosis, and tubular atrophy, resulting in progressive allograft dysfunction and, ultimately, graft loss^2–4^. Due to its asymptomatic presentation, SCAR may go undetected, delaying timely therapeutic intervention and compromising long-term outcomes.

SCAR incidence is variable, with studies reporting rates between 10% and 30% in kidney transplant recipients, depending on the timing of biopsy and follow-up duration^5–11^. Although large-scale, randomized prospective trials are still lacking, multiple studies suggest that the treatment of SCAR improves renal function in both the short and long term^8,12^.

Currently, a protocol biopsy program remains the only way to detect SCAR and guide timely therapeutic intervention aimed at preventing irreversible tubular atrophy and interstitial fibrosis, thereby preventing long-term deterioration of renal function^13^. However, implementing and maintaining an effective protocol biopsy program often requires patient hospitalization and, although rarely, exposes patients to the risk of this invasive procedure^14^. In addition, a protocol biopsy program requires specific organizational resources, and demands careful consideration of its cost to the National Health System. These considerations highlight the urgent need to develop a non-invasive, straightforward, and reliable biomarker for the diagnosis of SCAR^15^.

In recent years, the expansion of high-throughput technologies has brought novel insights into the pathogenesis of acute rejection in kidney transplantation with many genes reported to be involved in the rejection process^16–20^. Several studies have explored non-invasive approaches, such as urinary protein profiles and blood transcriptomic signatures, for detecting SCAR^21–23^; however, the findings have been inconsistent to date.

To our knowledge, this is the first study that applies high-throughput transcriptomic profiling to formalin-fixed, paraffin-embedded (FFPE) renal tissue from kidney transplant recipients with SCAR, and to explore its relationship with urinary protein excretion profiles as a strategy for biomarker discovery.

Our study focuses on the following four main objectives with potential immediate clinical usefulness for SCAR diagnosis: (i) to evaluate the incidence of SCAR in a clinical study of patients with kidney transplant who underwent biopsies at established times, (ii) to analyze the molecular pattern of SCAR tissue, (iii) to identify urinary biomarkers linked to gene expression in renal tissue available for SCAR diagnosis and (iv) to validate the identified biomarkers in an external independent cohort of patients with kidney transplant.

## Material and methods

### Patient collection and clinical management

#### Discovery cohort

We performed a multicenter clinical study in which 150 kidney transplant recipients were enrolled (Table 1). Each participant was identified anonymously using a combination of the center number and patient number. Written informed consent was obtained from all patients. The study protocol was first approved, by the local ethics committee of the coordinating research center (Policlinic, Bari, Italy; approval no. 3914/2012) and, subsequently, by the local ethics committees of all participating transplant centres. All research was conducted in accordance with relevant guidelines and regulations, and in compliance with the principles of the Declaration of Helsinki. Furthermore, no organs, tissues, or clinical samples were obtained from prisoners; all were obtained from the participating institutions.

**Table 1.** Demographic and clinical characteristics of patients enrolled in the study.

	**Discovery cohort**				**Validation cohort**			
	**Study cohort**	**SCAR**	**CNTR**	**p**	**Study Cohort**	**SCAR**	**CNTR**	**p**
**Patients (No.)**	89	12	12		86	17	21	
**Sex, male n (%)**	58 (65)	8 (66)	7 (58)	0,999*	53(61)	11 (64)	11 (52)	0.5205*
**Age at Kidney biopsy (yrs)**	60.00±7.80	55.92±18.61	63.58±8.19	0.205	53.12±12.31	51.00±10.25	51.33±11.91	0.9278
**sCr (mg/dL), mean (SD)**	1.66±0.60	1.74±0.59	1.76±0.61	0.920	1.53±0.40	1.75±0.62	1.38±0.47	0.1402
**eGRF (ml/min/1.73m**^**2**^**)(CKD-EPI)**	45.7±15.2	47.23±24.31	41.54±20.07	0.538	54.10±19.63	50.00±22.31	62.48±27.01	0.1353
**24-h proteinuria (g)**	0.2±0.2	0.2± 0.1	0.2±0.1	0.943	0.2±0.1	0.2±0.2	0.3±0.2	0.2684

Values are expressed as mean±S.D. Abbreviations: sCr, serum creatinine; eGFR, estimated glomerular filtration rate has been calculated using the CKD-EPI creatinine formula (mL min-1 per 1.73 m2); SCAR, Patients with subclinical acute rejection. CNTR Control transplant patients with no evidence of subclinical acute rejection. Demographic and clinical data refer to the time of biopsy-proven diagnosis. * Fisher’s exact test.

The inclusion criteria were as follows: (i) recipient of a single kidney transplant from a deceased or living donor, (ii) age ≥18 years and (iii) a negative pregnancy test. The exclusion criteria were (i) donor age > 65 years, (ii) absence of a preimplantation biopsy, (iii) recipient with panel reactive antibody (PRA) levels >50% before transplantation, (iv) prior graft loss within 1 year of transplantation due to transplant rejection.

Induction therapy was performed using Basiliximab or antithymocyte globulin (ATG). Maintenance therapy was based on corticosteroids, mycophenolate (750–1000 mg/day), and tacrolimus adjusted to routine trough levels. No study participants underwent steroid withdrawal. All episodes of acute rejection were treated with three pulses of methylprednisolone (500–1000 mg/day).

BKPyV screening, management and antibiotic prophylaxis for *Pneumocystis jirovecii* were performed according to center-specific protocols.

### Validation cohort

To validate the urinary markers, we collected an independent cohort of 86 additional kidney transplant recipients from two centres, Brescia and Parma, both of which perform routine protocol kidney biopsies during the first year post-transplantation. The same inclusion and exclusion criteria as those applied to the discovery cohort were used. In accordance with clinical practice and to avoid confounders affecting urinary levels, patients with leukocyturia (n=24), urinary tract infection (n=13), BK polyomavirus replication (n=11), were excluded. A total of 38 patients remained, including 17 biopsy-proven SCAR cases, which were used as the external validation cohort for CXCL10, FasL, and the composite urinary biomarker analysis.

### Laboratory analyses

Blood and urine samples were collected at time points (T) 0, T3 and T12 months during follow-up. Serum samples were tested at different time points to detect anti-HLA antibodies. Urinary creatinine was measured using the automated Jaffé method. Cytokine levels were normalized to urinary creatinine concentration levels.

Midstream urine samples were collected on the day of protocol kidney biopsies and on the day of clinically indicated kidney biopsies. The urine samples were centrifuged at 3,000 rpm. for 15 min and the supernatants were separated and stored at −20°C until used for the measurement of creatinine, proteins and cytokines.

BKPyV screening was based on the detection of decoy cells and the measurement of BKPyV DNAemia levels if ≥ 3 decoy cells per 10 high-power fields were counted.

HLA-Donor-specific antibodies (DSA) were determined using a single-antigen flow bead assay (One Lambda, Los Angeles, CA, USA) on a Luminex platform. Beads showing a normalized mean fluorescence intensity greater than 500 were considered positive.

Urinary tract infection (UTI). Cytobacterial examination of urine was systematically performed at the time of urine sample collection. UTI was diagnosed by the following criteria: bacteriuria ≥ 10^5^ colony-forming units (CFU)/mL and leukocyturia >35 white blood cells/µL.

### Sample collection

Blood, urine, and tissue samples were processed and stored at –80 °C or –20 °C at local centers, then shipped to the coordinating center (Bari, Italy) for centralized analysis. A laboratory manual provided detailed instructions for collection, storage, and shipment. Kidney specimens were obtained using core needle biopsies (18 or 16 gauge) under ultrasonographic guidance. Two cores of renal tissue were obtained from each biopsy. One core was fixed in buffered formalin (4%) for 12 hours, then embedded in paraffin and processed for routine histological staining (hematoxylin-eosin, periodic acid - Schiff, silver-methenamine and Masson’s trichrome procedures). Biopsies were considered adequate when they contained ≥10 glomeruli and ≥2 arteries, with a minimum acceptable threshold of ≥7 glomeruli and ≥1 artery, in accordance with the Banff 1997 Working Classification. This same adequate biopsy core was used for RNA extraction. The second core was immediately snap-frozen in a Tissue-Tek optimum cutting temperature (OCT) medium, stored in liquid nitrogen, and then stained with fluorescein isothiocyanate-labeled goat antihuman immunoglobulin G (IgG), IgA, IgM, C3, C1q, and fibrinogen. Indirect immunofluorescence for C4d on frozen tissue or immunohistochemistry for C4d on formalin-fixed paraffin embedded tissue was carried out according to center policy.

### Pathology review

Histological lesions on the four renal compartments were scored according to the Banff 2009 classification^9^. SCAR was defined as the presence of histological lesions indicative of acute rejection in the allograft biopsy, specifically, Banff criteria scores of i≥2 and t≥2 scores, evidence of microvascular inflammation (g≥1 and/or ptc≥1), with C4d positivity and/or circulating donor-specific antibodies (DSA) or equivalent evidence of antibody–endothelial interaction, in the absence of clinical or laboratory signs of renal dysfunction.  

The first reading of the kidney biopsy was performed by the pathologist at the local institution, and therapeutic decisions were taken based on the biopsy report. After the first six months of the follow-up, and every six months thereafter, all stained slides and two blank slides were sent to the independent Central Renal Pathology Laboratory, where a second blinded reading was performed by two independent pathologists. All cases for which there was agreement between the diagnosis of the two independent pathologists were considered for a microarray study and subsequent data analysis.

### RNA extraction, gene expression profiling, and bioinformatic analysis

Supplementary Figure 1 illustrates the methodological overview of the transcriptomic study. Total RNA was extracted from FFPE renal tissue samples following deparaffinization and lysis. Briefly, eight freshly cut 5 µm sections were deparaffinized using a Qiagen deparaffinization solution at 56 °C for 3 minutes. The sections were then incubated in a lysis buffer containing proteinase K at 56 °C for 15 minutes to release RNA from crosslinked proteins, followed by a 15-minute incubation at 80 °C to reverse formalin-induced crosslinking. RNA was immediately extracted using the RNeasy FFPE Kit (Qiagen, GmbH, Hilden, Germany) and used for both microarray analysis and real-time PCR. Prior to microarray processing, RNA integrity was assessed using Agilent RNA nano chips on an Agilent 2100 Bioanalyzer, yielding RNA integrity numbers (RIN) between 2.1 and 2.9, with RNA fragments ranging from 100 to 200 nucleotides. Quantification was performed using a NanoDrop 1000 spectrophotometer.

The hybridization was carried out according to the Agilent Gene Expression FFPE Workflow. In brief, 400 ng of total RNA was reverse transcribed into a double-stranded cDNA library using the Transplex Whole Transcriptome Amplification (WTA) Kit (Sigma) and HotStarTaq Plus DNA Polymerase (QIAGEN), followed by purification with the QIAquick PCR Purification Kit (QIAGEN). One microgram of the amplified library was then labelled with ULS-Cy3 dye for 30 minutes at 85 °C using the Genomic DNA ULS Lab Kit (Agilent) and subsequently cleaned using Agilent-KREApure columns. The purified, Cy3-labeled samples were combined with Agilent 10x Blocking Agent and Agilent 2x Hi-RPM Hybridization Solution, with Agilent-CGHblock added before denaturation at 95 °C for 3 minutes. Hybridization was performed on the SurePrint G3 Hmn GE v.2 8x60K microarray for 17 hours at 65 °C, followed by washing as per the Agilent One-Color Microarray-Based Gene Expression Analysis manual.

Microarray slides were scanned using an Agilent SureScan Microarray Scanner, and the images were processed with Agilent Feature Extraction v.10.7.3.1 software (protocol GE1_107_Sep09, Grid: 072363_D_F_20150612) to obtain background-subtracted and spatially detrended processed signal intensities. The data were then analyzed with GeneSpring 13.1 (Agilent) using the following import settings: threshold 1.0, log base 2 transformation, Agilent.SingleColor.72363 technology 75th percentile normalization. Differentially expressed genes (DEGs) were identified using the Benjamini–Hochberg false discovery rate correction (adjusted P-value < 0.02) and a log2 fold change greater than 2. The microarray data are MIAME-compliant and have been deposited in the GEO database under the accession number GSE294632.

DEGs were functionally annotated using the DAVID bioinformatics resource (https://david.ncifcrf.gov/tools.jsp, 2022 update). This platform enables the retrieval of biological data related to gene sets and performs enrichment analyses based on Gene Ontology (GO; www.geneontology.org), covering three main categories: biological processes (BP), cellular components (CC), and molecular functions (MF).

To explore the biological significance of the DEGs, canonical pathway analysis was carried out through Ingenuity Pathway Analysis (IPA). The list of DEGs, including log 2fold change (log2FC) values and corresponding FDR q-values, was uploaded into IPA for further interpretation. The relevance of each pathway was assessed using a right-tailed Fisher’s Exact Test, which estimates the likelihood that the observed associations between genes and pathways are due to chance. Moreover, IPA’s z-score algorithm was used to predict pathway activation states by analyzing the direction and strength of gene regulation in the context of known pathway interactions. Pathways with p-values < 0.05 and z-scores either greater than zero (activation) or less than zero (inhibition) were regarded as statistically significant.

### Quantitative real-time (qRT) PCR analysis

Real-time PCR was performed to validate DEGs identified through microarray analysis. Reverse transcription (RT) was carried out using the Whole Transcriptome Amplification Kit (Sigma-Aldrich, St. Louis, MO, USA), specifically optimized for RNA amplification from FFPE samples. Each RT reaction included 250 ng of total RNA, and the resulting cDNA was purified using the MinElute Purification Kit (Qiagen GmbH, Hilden, Germany). For real-time PCR, reactions were performed in triplicate using PrimeTime qPCR Primer Assays (IDT) for the following genes: *Nuclear Factor Kappa B Subunit Zeta* (*NFKBIZ*, Hs.PT.58.39323813), *SLAM Family Member 8* (*SLAMF8*, Hs.PT.58.3496550), *CD247 Molecule* (*CD247*, Hs.PT.56a.239073.g), *Tumor Necrosis Factor Superfamily Member 14* (TNFSF14, Hs.PT.58.1717444), *C-X-C Motif Chemokine Ligand 10* (*CXCL10*), and *Fas Ligand/TNFSF6 (FasL*), using 30 ng of diluted cDNA as input.

Each amplification reaction was conducted in a final volume of 25 µL using SYBR Green chemistry (SensiMix SYBR Hi-ROX kit, Bioline) on a StepOnePlus Real-Time PCR System (Thermo Fisher Scientific). The *β-actin* gene served as the internal reference for normalizing target gene expression. Amplification specificity was verified via melting curve analysis. mRNA target levels were quantified using the comparative cycle threshold (ΔCt) method, and gene expression data were calculated using the 2⁻ΔΔCt approach. Potential outliers were identified using the Smirnov–Grubbs test, implemented in GraphPad Prism (version 8.0.2). Results are presented as mean ± standard deviation (SD).

### Immunohistochemistry

Protein expression was analyzed using immunohistochemistry on kidney biopsy samples from the same patients involved in the microarray study. Tissue sections (2 μm thick) were prepared from paraffin-embedded blocks, then deparaffinized in xylene and rehydrated through a graded series of alcohol. After antigen retrieval, endogenous peroxidase activity was blocked by incubating the sections in a 3% hydrogen peroxide solution for 7 minutes. Finally, the slides were incubated at room temperature for 10 minutes with a serum-free protein blocking reagent (Dako, Glostrup, Denmark). Subsequently, the slides were incubated with rabbit polyclonal antibodies under the following conditions: Anti-human NFKBIZ (Atlas Antibodies, Cat# HPA010547): 1:75 dilution, overnight incubation at +4 °C; Anti-human TNFSF14 (Atlas Antibodies, Cat# HPA012700): 1:10 dilution, overnight incubation at +4 °C; Anti-human SLAMF8 (Atlas Antibodies, Cat# HPA067601): 1:200 dilution, overnight incubation at +4 °C; Anti-human CD247 (Atlas Antibodies, Cat# HPA008750): 1:150 dilution, 1-hour incubation at room temperature. The binding of the secondary biotinylated antibody was detected by the Dako Real EnVision, Peroxidase/DAB kit (Dako), according to the manufacturer’s instructions. The peroxidase reaction was shown by a brown precipitate, counterstained with Mayers hematoxylin (blue), and mounted with Glycergel (DakoCytomation, Carpinteria, CA, USA). Negative controls were obtained incubating serial sections with the blocking solution and then omitting the primary antibody. Slides were scanned using the Aperio ScanScope System (Aperio, Technology; Vista, CA) at 40× magnification to provide a high-resolution digital image. Aperio-specific ImageScope software (available for free from Aperio.com) was used to measure staining intensity and the percentage of positive cells using the Positive pixel count v9 algorithm. Analysis was performed on the entire biopsy, assessing both glomerular and tubular compartments together, as previously described^24^. For each section, the intensity of the staining with absent (0) to strong (+++) was converted into a number. Only high intensity pixels (identified by the software as strong positive) were considered as a positive staining and were normalized to the selected area (total number of pixels in the section).

#### Enzyme‑linked immunosorbent assays

Urinary levels of NFKBIZ (MyBioSource, Cat. No. MBS9321660), TNFSF14 (MyBioSource, Cat. No. MBS065102), SLAMF8 (MyBioSource, Cat. No. MBS9322325), CD247 (MyBioSource, Cat. No. MBS773629), CXCL10 (R&D Systems Quantikine ELISA Kit, Cat. No. DIP100), and FasL (TNFSF6, R&D Quantikine® ELISA, Cat. No. DFL00B) were quantified using commercially available Enzyme-linked immunosorbent assays (ELISA) kits. All assays were performed according to the manufacturers’ protocols. All urinary samples were processed immediately after collection and centrifuged at 3,000×g for 10 min to remove cellular debris. The supernatants were then collected, aliquoted and immediately stored at − 80 °C until use. Undiluted midstream urine samples were analyzed in duplicate in accordance with the manufacturer’s instructions. Samples with measurements below the assay’s detection limit were excluded from the analysis. Outliers were identified and removed using the ROUT method (Q = 1%) to ensure data robustness. Urinary creatinine concentration was determined using the Jaffé method, and cytokine values were normalized to urinary creatinine levels to account for variations in urine concentration.

### Statistical analysis

Continuous variables were expressed as mean ± standard deviation (SD), while categorical variables were summarized as counts and percentages. Normality was assessed using the Shapiro–Wilk test. Comparisons between independent groups were performed using Fisher’s exact test for categorical variables. Differences between the two groups were assessed using the two-tailed Student’s t-test for normally distributed data and the Mann–Whitney U test for non-normally distributed data. A p-value < 0.05 was considered statistically significant. All statistical analyses and graphical representations were performed using GraphPad Prism (version 8.0.2).

Univariate logistic regression was performed and a ROC curve was generated based on urinary CXCL10 ng/mmol creatinine or FasL ng/mmol creatinine as a continuous variable. A range of sensitivities and specificities was then generated based on varying creatinine-normalized cut-off values derived from the ROC curve. The area under the ROC curve (AUC) was calculated to evaluate the overall discriminative ability of CXCL10 and FasL. The optimal cut-off value for the CXCL10 or FasL ratio was determined using the Youden Index, which maximizes the sum of sensitivity and specificity. The corresponding sensitivity and specificity values were reported along with their 95% confidence intervals (CIs). To evaluate whether combining the two biomarkers improved diagnostic performance, we created a composite biomarker value for each subject by summing the creatinine-normalized CXCL10 and FasL concentrations; this simple additive composite signature was then entered into a separate logistic regression model, and a ROC curve was generated from the resulting predicted probabilities, analogous to the single-marker analyses. ROC analysis was conducted using GraphPad Prism (version 8.0.2); the Youden Index was calculated manually and statistical significance was considered at p < 0.05.

## Results

### Patient enrolment and SCAR incidence

Out of the 150 kidney transplant patients initially recruited, 42 were lost to follow-up, 19 dropped out, leaving 89 patients for the final analysis. Patients lost to follow-up were excluded because their graft status could not be verified at the biopsy level; without histological confirmation, it was not possible to accurately classify them as either SCAR or control. Including such cases would have introduced misclassification bias and potentially compromised the reliability of the biomarker performance assessment. Consequently, only patients with biopsy-verified outcomes were retained for the discovery and validation phases, ensuring diagnostic accuracy and data integrity. Of the 89 patients who remained eligible for analysis, all underwent an adequate protocol biopsy at T3 and 32 of them also received an adequate biopsy at both T3 and T12 (Table 1, Supplementary figure 2). Among the participants, 65% were male. The mean age of the kidney recipients was 60.0 ± 7.8 years while the mean age of their donors was 61.7 years (10% were living donors). Induction therapy was administered using Basiliximab in 45.2% of patients or ATG in 36.9%. Triple therapy (corticosteroids, calcineurin-inhibitors, and mycophenolate) was administered in 80% of the transplant recipients, and corticosteroids, calcineurin-inhibitors and everolimus were used in 20% of study participants. The incidence of SCAR was 16%, defined by biopsy lesions occurring despite stable serum creatinine levels and in the absence of clinical or laboratory evidence of renal dysfunction. No differences were observed in the doses of immunosuppressive drugs administered to patients with versus without SCAR.

### Identification of specific gene expression signatures in SCAR

From the cohort of protocol biopsies performed at 3 or 12 months post-transplantation, patients with leukocyturia, urinary tract infections, BK polyomavirus replication were excluded. After these exclusions, 12 patients with biopsy-proven SCAR and 12 transplant recipients with normal histological findings (CNTR, Table 1) remained and were included in the microarray analysis. In the SCAR group, there were 8 males and 4 females, with a mean age at kidney biopsy of 55.92±18.61 years. In the CNTR group, there were 7 males and 5 females with a mean age at kidney biopsy of 63.58 ± 8.19 years. The mean serum creatinine values were 1.74±0.59 mg/dL in the SCAR group and 1.76 ± 0.61 mg/dL in the CNTR group. The mean eGFR values were 47.23 ± 24.31 ml/min/1.73m^2^ in SCAR group and 41.54 ± 20.07 ml/min/1.73m^2^ in the CNTR group.

**Fig. 1:**
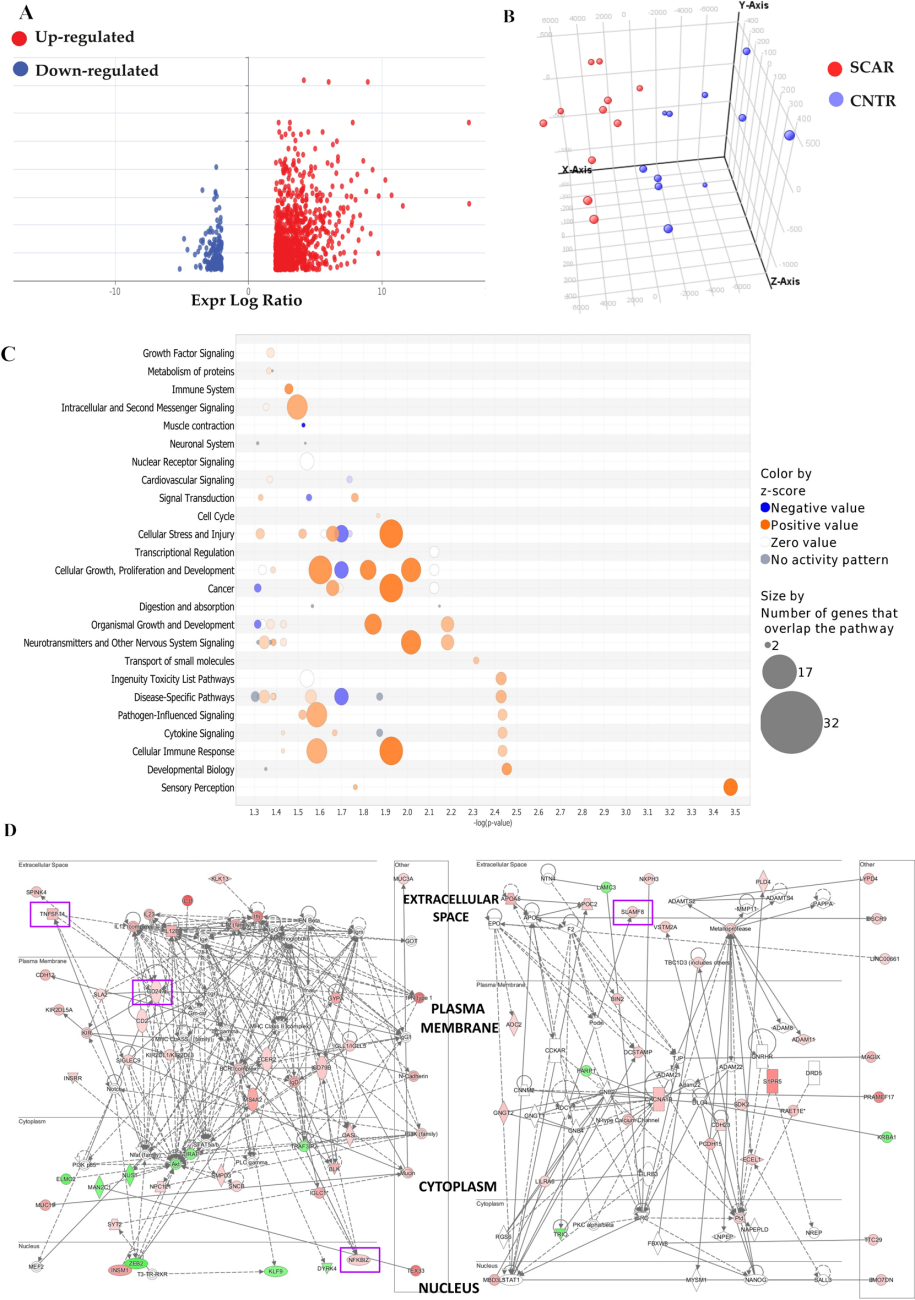
Gene expression analysis in SCAR and control kidney transplant patients. (**A**) Volcano plot showing differentially expressed probes in SCAR patients compared to controls. Red dots represent upregulated probes, and blue dots represent downregulated probes (Log2FC > 2, FDR-adjusted p-value < 0.02). (**B**) Three-dimensional Principal Component Analysis (PCA) illustrating a distinct separation between SCAR (red) and control (blue) samples, indicating a divergent transcriptional profile between the two groups. (**C**) Bubble chart of top canonical pathways identified by Ingenuity Pathway Analysis (IPA), grouped by functional category. Each bubble represents a pathway, with color indicating activation z-score (orange = positive activation, blue = negative activation, white = no change, grey = no prediction), and size corresponding to the number of genes involved. The x-axis denotes the significance of enrichment (–log(p-value)). (**D**) Networks generated with IPA after uploading the 1849 gene probe sets. Top ranked networks generated by differentially expressed genes. The left (score 47, focus molecules 38) and right (score 36, focus molecules 35) panels show molecular interaction networks with key upregulated (red) and downregulated (green) nodes annotated by cellular localization (extracellular space, plasma membrane, cytoplasm, nucleus). Highlighted in magenta, *NFKBIZ, SLAMF8, CD247*, and *TNFSF14* are the key players in the immune response associated with SCAR.

Bioinformatics analysis revealed 1,849 differentially expressed probes in SCAR patients compared to the CNTR group (fold change > 2, FDR-adjusted p-value < 0.02**, **Supplementary Table 1). Among these, 1,257 probes were upregulated and 184 were downregulated (Fig. 1A). Three-dimensional Principal Component Analysis revealed a different spatial distribution between the patterns of SCAR patients and controls therefore the probes successfully distinguished aberrantly modulated genes in SCAR biopsies from those in patients with a normal histological pattern (Fig. 1B).

The DEGs in the dataset were primarily enriched in Biological Processes related to sensory perception and signal transduction. Notably, a significant enrichment was observed in the detection of *chemical stimulus involved in sensory perception of smell* (N=35, adjusted p= 3.2E-5), suggesting a robust involvement of olfactory-related pathways. Additionally, the *G protein-coupled receptor signalling pathway* was highly represented (N=53, adjusted p= 3.1E-4), reflecting the central role of this pathway in mediating extracellular signals. From a Cellular Component perspective, a substantial proportion of DEGs were associated with the *plasma membrane* (N=205, adjusted p= 3.8E-4), indicating that many of the encoded proteins may function as receptors or signalling molecules at the cell surface. In terms of Molecular Function, there was a strong enrichment for olfactory receptor activity (N=35, adjusted p= 9.7E-5) and G protein-coupled receptor activity (N=47, adjusted p=5 1.1E-4), further supporting the prominence of chemosensory and signalling processes (Supplementary Table 2).

The most significantly enriched canonical pathways identified by IPA using DEGs include *‘Sensory Perception*’, *‘Cellular Immune Response’*, *‘Immune System’*, and *‘Cytokine Signalling’*, as well as several pathways related to *cell-to-cell communication* (Fig. 1C). These pathways not only showed strong statistical significance, mostly with a –log(p-value)>1.5, but also involved a large number of DEGs, as indicated by the larger bubble sizes. Additionally, the predominance of orange-coloured bubbles reflects an overall activation of these pathways, particularly highlighting the upregulation of immune and sensory-related responses in the analysed samples (Fig. 1C). Further insights were obtained by generating molecular interaction networks from the same set of DEGs (Fig. 1D). The top-ranked networks using DEGs exhibited a high degree of gene interconnectivity (score = 60, n = 48 associated genes, Fig. 1D, left panel; score = 50, n = 35 associated genes, Fig. 1D, right panel). Notably, these networks identified *NFKBIZ*, *SLAMF8*, *CD247*, and *TNFSF14* genes involved in immune signalling, acting as central hubs potentially contributing to the immune-mediated injury observed in SCAR. These findings reinforce the immunological basis of SCAR and highlight key regulatory nodes that may serve as potential biomarkers or therapeutic targets.

### Quantitative real-time PCR analysis of NFKBIZ, TNFSF14, SLAMF8, and CD247 Gene expression in renal tissue

**Fig. 2:**
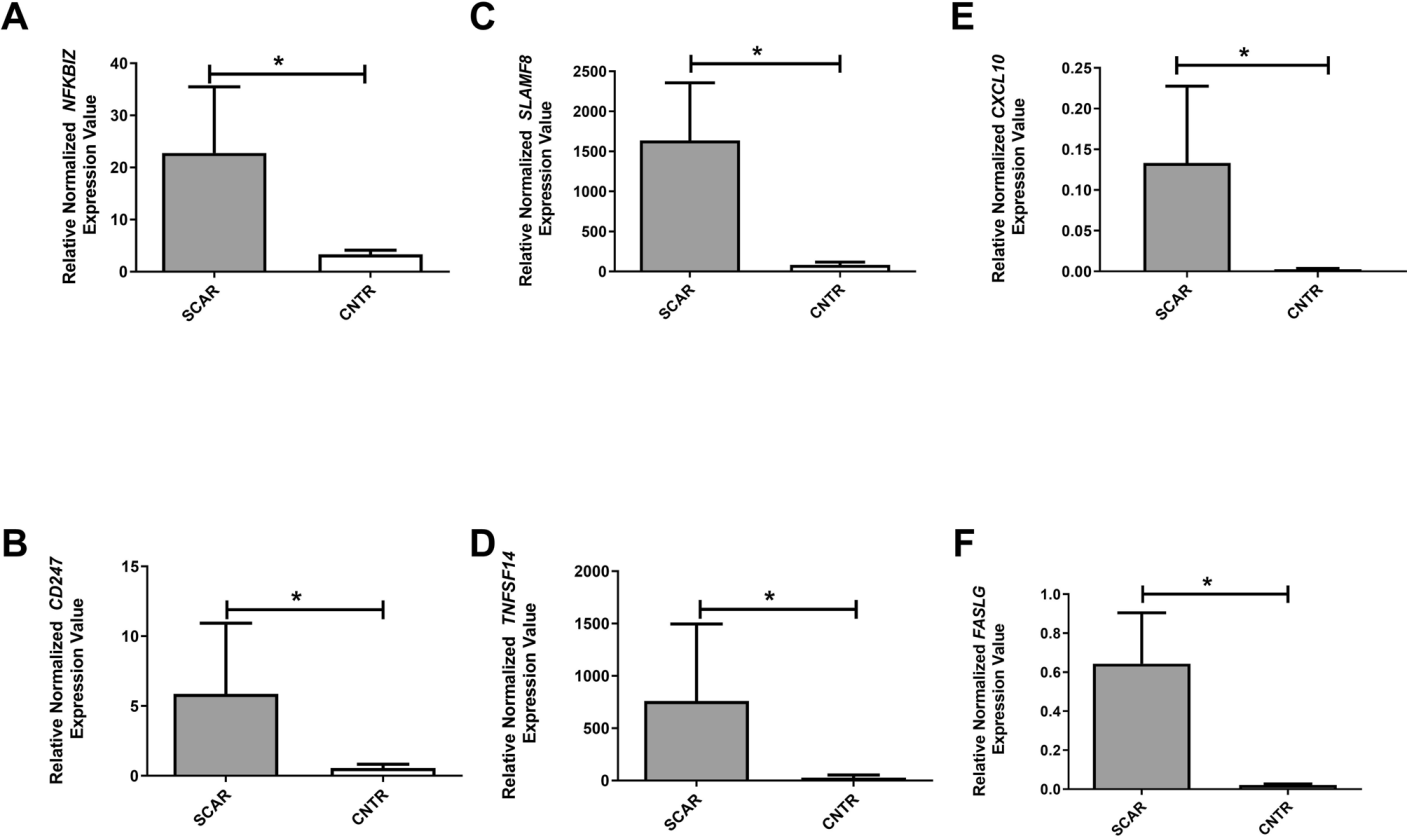
Gene expression levels evaluated by real-time (RT)-PCR in kidney biopsy specimens. Relative normalized expression values of *NFKBIZ* (**A**), *CD247* (**B**), *SLAMF8* (**C**), *TNFSF14* (**D**), *CXCL10* (**E**), and *FASLG* (**F**) were measured in SCAR (subclinical acute rejection) and control (CNTR) samples. In all cases, gene expression was significantly higher in SCAR samples compared to controls. Statistical significance was assessed using the Mann–Whitney or unpaired t-test, as appropriate; *p* < 0.05 for all comparisons. Bars represent mean ± standard deviation (SD).

To further establish the validity of gene expression determined by microarray analysis, we performed RT-PCR on the same patient groups used for the microarray study. Filtering the 1,849 differentially expressed probes for those with the highest fold-change values and lowest corrected p-values, we identified several highly dysregulated non-coding RNAs (e.g., PABPC1L2B-AS1, lnc-HJURP-1, XLOC_l2_006036, LINC00052, lnc-AL035696.1–3, lnc-STXBP1-2; as well as protein-coding genes such as SIGLEC12, TRAF2, OR2B6, Supplementary Table 3). However, these genes did not emerge as central nodes within the SCAR-associated interaction networks. For this reason, fold-change magnitude alone was not used as the primary criterion for downstream validation. Instead, we focused on representative genes that were found differently expressed in SCAR focalizing our attention on those that were present in the top ranked networks (Fig. 1D, Supplementary Figure 3). To reinforce the robustness of our approach, the four transcripts were not arbitrarily selected but objectively emerged as the most interconnected and biologically relevant hub nodes within the unbiased DEG-derived interaction networks. We selected *SLAMF8*, previously associated with renal transplant rejection^25^ and included in a classifier for the molecular diagnosis of T-cell mediated rejection^26^. *NFKBIZ* was also included in a classifier capable of predicting future graft failure^27^; it has been found to be associated with renal fibrosis^28^. *CD247* was selected because it is part of the T-cell receptor-CD3 complex on T cells and activating receptors on NK cells^29^, and its expression is associated with kidney survival^30^. Lastly, we selected *TNFSF14*, a pro-fibrotic factor^31^ that plays an important role in T cell activation and, together with other genes, has been found to discriminate patients with Acute Renal Allograft Rejection^32^. To further assess whether the four selected genes (*SLAMF8*, *NFKBIZ*, *CD247*, and *TNFSF14*) are also involved in overt acute rejection, we explored their expression in publicly available reference datasets. Using the PROMAD atlas^33^, we examined transcript levels across multiple cohorts of biopsy-proven acute allograft rejection. Across PROMAD datasets, all four genes consistently showed higher expression in rejecting grafts compared to stable allografts, although the magnitude of upregulation varied between cohorts (Supplementary Figure 4). This external validation indicates that the four-gene signature identified in SCAR is also detectable in clinically overt rejection and reflects a conserved inflammatory pathway across different rejection phenotypes. Normalized gene expression levels of *NFKBIZ*, *SLAMF8*, *CD247*, and *TNFSF14* were significantly higher in the SCAR group compared to the CNTR group (Mann Whitney test, P = 0.006; Mann Whitney test P = 0.015; Unpaired t test P = 0.037; P = 0.016 Fig. 2 A, B, C, and D, respectively). These results were consistent with those obtained from the gene expression array.

### Renal tissue NFKBIZ, SLAMF8, CD247, and TNFSF14 protein expression levels in SCAR group

**Fig. 3:**
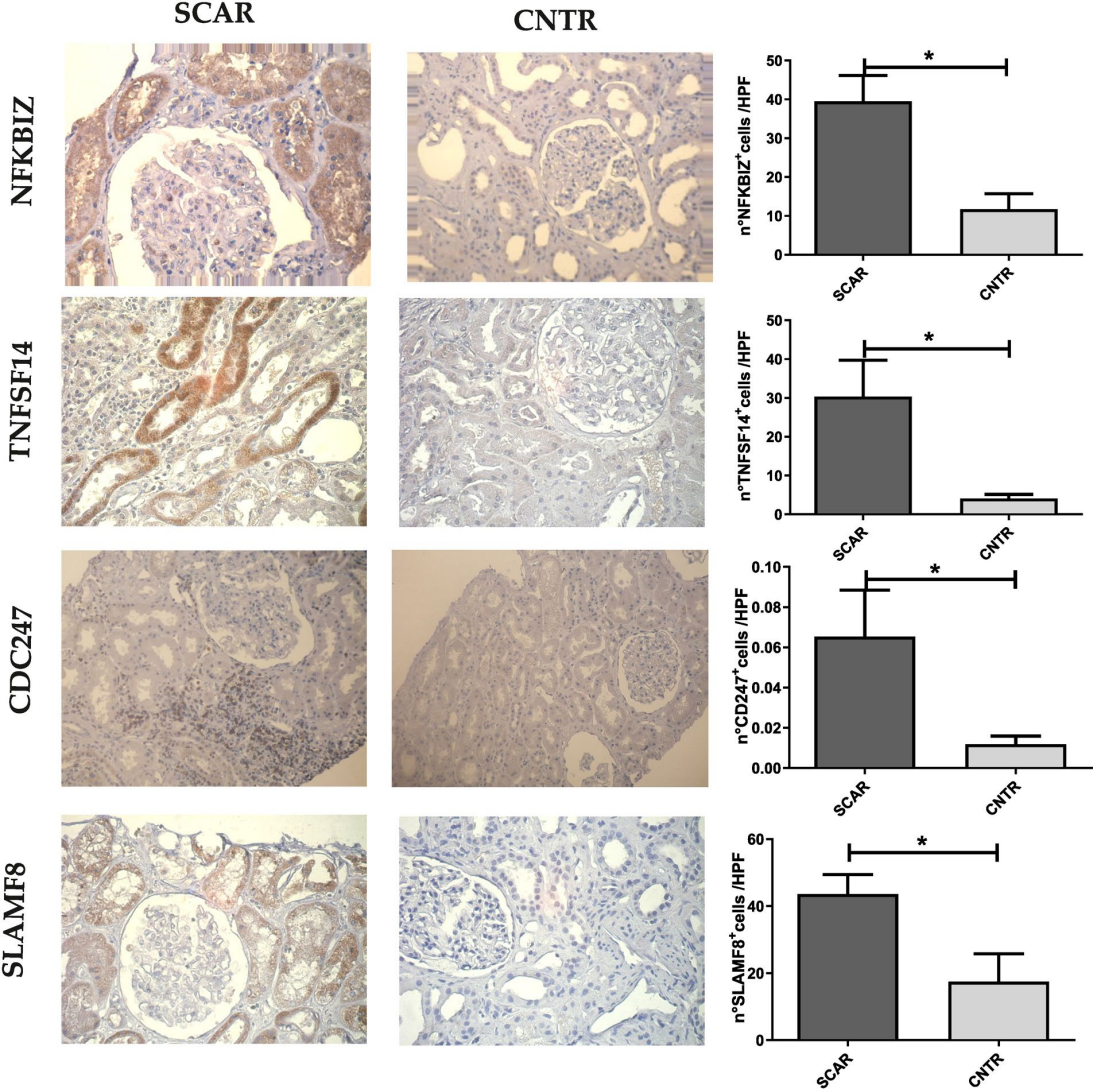
Immunohistochemical analysis of NFKBIZ, TNFSF14, CD247, and SLAMF8 expression in SCAR and CNTR kidney samples. Representative images show strong positive staining for NFKBIZ, predominantly nuclear and cytoplasmic in tubular cells in SCAR samples compared to minimal staining in controls (Unpaired t-test, p = 0.01). TNFSF14 exhibits strong membranous and cytoplasmic staining, mainly in tubular epithelial cells in SCAR samples, while controls show weak staining (Mann–Whitney test, p = 0.03). CD247 staining appears scattered and weak, localized primarily to infiltrating immune cells in the interstitial area in SCAR kidneys, and is barely detectable in controls (Mann–Whitney test, p = 0.03). SLAMF8 displays intense membranous staining in tubular epithelial cells in SCAR samples, with a much weaker signal observed in controls (Unpaired t-test, p = 0.04). Quantification of positive cells per high-power field (HPF) is shown in the corresponding bar graphs. *p < 0.05.

Next, we evaluated the protein expression levels of NFKBIZ, TNFSF14, CD247, and SLAMF8 in SCAR patients compared to CNTRs. A strong positive expression of NFKBIZ was observed in SCAR patients, with nuclear and cytoplasmic staining mainly localized to tubular cells, while CNTR kidneys showed very faint or no staining (Unpaired t-test, p = 0.01; Fig. 3). TNFSF14 immunohistochemical staining showed strong membranous and cytoplasmic localization, predominantly in the tubular epithelial cells of SCAR samples, whereas controls displayed very little signal (Mann–Whitney test, p = 0.03; Fig. 3). CD247 staining appeared scattered and weak, primarily in infiltrating immune cells within the interstitial areas of SCAR kidneys, and was barely detectable in controls (Mann–Whitney test, p = 0.03**; **Fig. 3). Finally, SLAMF8 exhibited intense staining in tubular epithelial cells, suggesting a cell surface/membranous localization in SCAR kidneys, while controls showed a much weaker signal (Unpaired t-test, p = 0.04; Fig. 3).

### Evaluation of urinary levels of NFKBIZ, SLAMF8, CD247, and TNFSF14

Next, we determined whether the proteins NFKBIZ, SLAMF8, CD247, and TNFSF14 upregulated in renal tissue were also detectable in urine samples and whether the significant differences in expression observed in tissue were maintained in the urine. This analysis aimed to identify potential urinary biomarkers concordant with renal gene expression changes, offering a non-invasive diagnostic tool for SCAR. However, ELISA assays for NFKBIZ, SLAMF8, CD247, and TNFSF14 revealed that protein levels in urine were below the limit of quantification in both SCAR patients and CNTRs, indicating that these markers are not detectable in urine using this method.

### Biomarker identification for SCAR

**Fig. 4:**
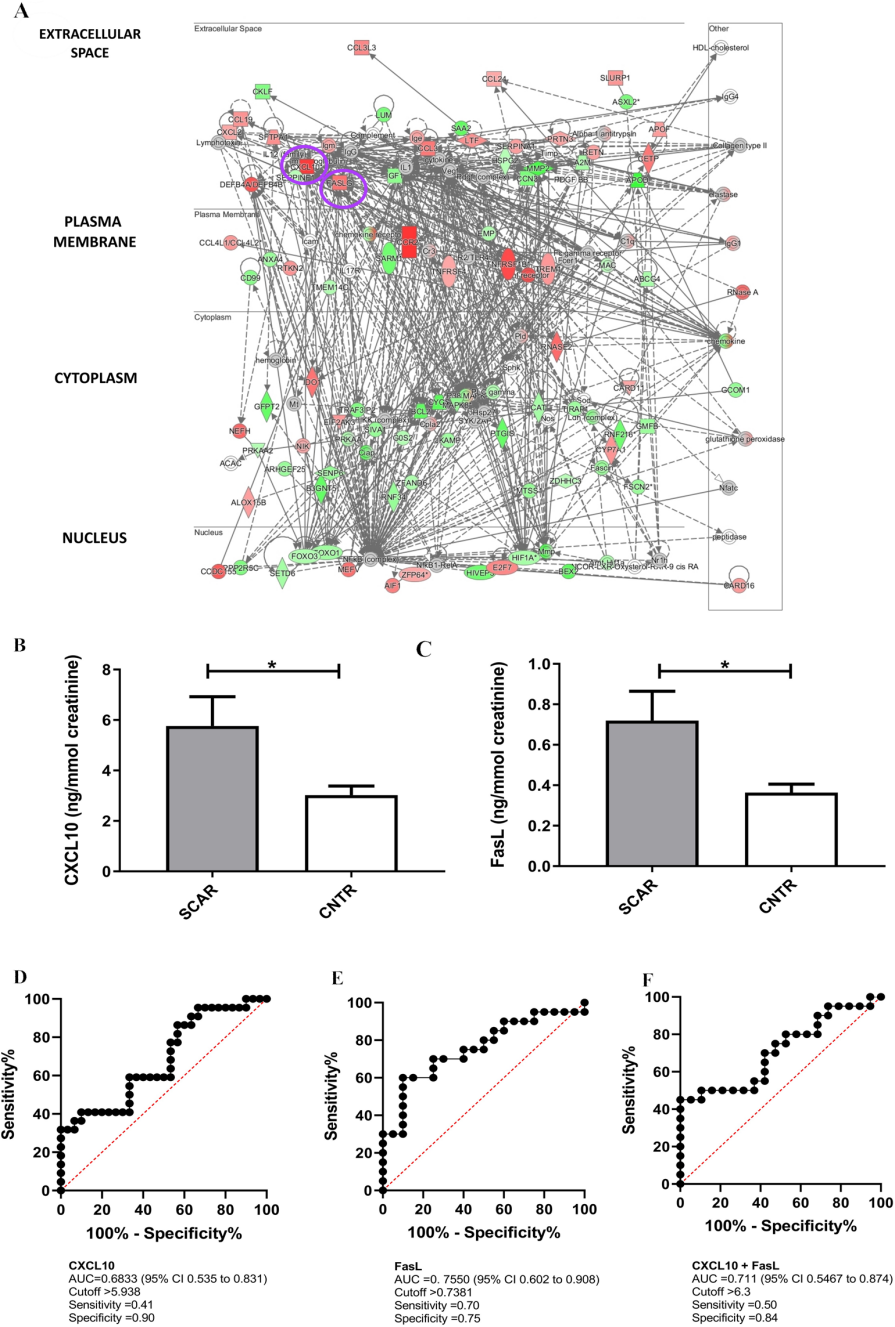
CXCL10 and FasL are upregulated in SCAR and detectable in urine as potential non-invasive biomarkers in an independent validation cohort (**A**) Ingenuity Pathway Analysis (IPA) network visualization generated from the 14,017 differentially expressed probe sets*. CXCL10* and *FasL* are highlighted within the network, both upregulated and localized to the extracellular space, making them suitable candidates for urinary biomarker evaluation. (**B, C**) Urinary levels of CXCL10 (B) and FasL (C) measured by ELISA were significantly elevated in patients with subclinical acute rejection (SCAR) compared to control (CNTR) patients (Unpaired t-test: CXCL10, p = 0.04; FasL, p = 0.02). Results are expressed as ng/mmol creatinine. (**D, E**) Receiver operating characteristic (ROC) curve analysis for urinary CXCL10 (**D**) and FasL (**E**) demonstrating their diagnostic performance in distinguishing SCAR from controls. For CXCL10, the area under the curve (AUC) was 0.6833 (95% CI: 0.5354–0.8313) (**D**), with a sensitivity of 41%, and specificity of 90% at a cutoff value of 5.938 ng/mmol creatinine. FasL showed superior diagnostic accuracy with an AUC of 0.7550 (95% CI: 0.6018–0.9082) (**E**), a sensitivity of 70%, and specificity of 75% at a cutoff of 0.7381 ng/mmol creatinine. The combined ROC analysis of the composite biomarker signature (CXCL10 + FasL), yielded an AUC of 0.711 (95% CI 0.549–0.874, Fig. 4E), with a sensitivity of 50% and specificity of 84% at the optimal cutoff of 6.3 ng/mmol creatinine. (**F**).

To select potential urinary biomarkers that can easily be measured in urine, we expanded our gene expression analysis and performed a multiple-step filtering strategy. First, we applied less stringent criteria to our differential gene expression data (FDR < 0.05, log₂FC > 1.5), resulting in a total of 14,017 probe sets for further analysis. Next, we used IPA to focus on *cytokines* that are secreted into the *extracellular space*, as these are more likely to be found in biological fluids such as urine (Fig. 4A**, **Supplementary Table 4) and we focused on those highly expressed in the kidney using the online resource (https://www.proteinatlas.org/)^34^with the highest fold change. This refined selection criterion identified two very important cytokines included as molecular markers in acute renal transplantation, CXCL10 and FasL^35,36^. CXCL10 is secreted by infiltrating inflammatory cells and renal tubular cells and it can also be produced by several other renal resident cell types, including endothelial cells, mesangial cells, and podocytes^37^; it plays a role in leukocyte recruitment and in the mediation of the CD4+ Th1 immune response^38,39^ and is an extremely good biomarker in kidney transplantation^35^. FasL, on the other hand, is a member of the tumor necrosis factor (TNF) family that plays a key role in inducing apoptosis and modulating immune responses, particularly in the context of graft rejection^40^.

First, we validated the microarray gene expression differences between SCAR and CNTR by qRT-PCR. We found that the normalized gene expression levels of CXCL10 (Mann–Whitney test, p = 0.03; Fig. 2E) and FasL (Mann Whitney test, p =0.0016; Fig. 2F) were significantly higher in SCAR patients compared to CNTR. Consistently elevated expression levels of CXCL10 and FASLG were also observed in rejecting grafts across the PROMAD cohort (Supplementary Figure 5).

Next, we evaluated whether CXCL10 and FasL were detectable in the urine of 12 biopsy-proven SCAR patients and 12 controls. This analysis showed that CXCL10 and FasL levels were significantly elevated in SCAR patients (Unpaired t-test, p=0.04, p=0.02 respectively, Supplementary Figure 6). Our data were tested in an independent validation cohort of 86 transplant patients. After excluding patients with leukocyturia (n=24), urinary tract infection (n=13), BK polyomavirus replication (n=11), 38 patients remained, including 17 with biopsy-proven SCAR (Table 1). These patients confirmed that CXCL10 and FasL levels were significantly elevated in SCAR patients (respectively, Mann Whitney test, p=0.04; Mann Whitney test, p=0.02 Fig. 4B,C). Receiver operating characteristic (ROC) analysis performed on all tested patients showed that CXCL10 had an AUC of 0.6833 (95% CI 0.5354–0.8313, Fig. 4D), with a sensitivity of 41% and specificity of 90% at a cutoff value of >5.938. FasL demonstrated a higher diagnostic performance, with an AUC of 0.7550 (95% CI 0.602–0.9082, Fig. 4E), a sensitivity of 70.0%, and specificity of 75.0% at a cutoff of >0.7381. When considering the composite biomarker signature (CXCL10 + FasL), the combined ROC analysis yielded an AUC of 0.711 (95% CI 0.549–0.874, Fig. 4E), with a sensitivity of 50% and specificity of 84% at the optimal cutoff (Fig. 4F**)**.

## Discussion

Our study focuses on four main objectives that can be translated into clinical usefulness for SCAR diagnosis. First, to evaluate the incidence of SCAR in a clinical study in which patients with kidney transplant underwent protocol biopsies at predefined time points; (ii) to analyze the molecular pattern of SCAR tissue; (iii) to identify urinary biomarkers linked to gene expression in renal tissue available for SCAR diagnosis and (iv) to validate the identified biomarkers in an independent cohort of patients with kidney transplant.

Various studies have reported different rates of SCAR, likely due to variations in the duration of the observation and timing of biopsy collection^4,41,42^. In our study, SCAR was observed in 16% of patient population, which falls within the wide range from 2.6% to 61% reported by previous studies^5,12,43–47^. No difference was observed in the immunosuppressive drug doses administered to patients with or without SCAR. This data indicates that the diagnosis of SCAR remains critical for kidney transplant recipients who receive triple therapy. Furthermore, this evidence suggests the need for more oversight over outpatients on medications to address non-adherence to therapy, which may be responsible for SCAR.

Our transcriptomic analysis of FFPE kidney biopsies revealed multiple genes associated with SCAR. The microarray was performed only on 12 cases and 12 controls, as several samples were excluded due to inflammatory processes such as leukocyturia, urinary tract infections, BK polyomavirus replication. Although not derived from a formal power calculation, this sample size was intended to provide an initial discovery cohort from which candidate biomarkers could be identified and subsequently validated in an independent patient set. The initial gene signature was composed of four key gene*s****,**** NFKBIZ, TNFSF14, SLAMF8,* and *CD247,* which reflect the molecular inflammatory processes and immune cell crosstalk characteristic of SCAR. The involvement of these genes was further validated by RT-qPCR and immunohistochemistry, confirming the presence of the transcripts and their corresponding proteins in kidney tissue specimens. Next, we assessed whether the transcribed proteins were also differently expressed in urine from SCAR patients compared to CNTRs. We focused on protein-level measurements because detecting low-abundance transcripts in urine is technically challenging, even though pre-amplification strategies have been shown to substantially increase sensitivity^48^. However, implementing such approaches requires RNA extraction to be performed within 4 hours of urine collection, a condition that could not be met in our multicenter design. In our workflow, blood, urine, and tissue samples were processed and stored at –80 °C or –20 °C at the respective clinical centers and subsequently shipped to the coordinating site for centralized analyses. For these reasons, we opted for protein evaluation, which is more stable over time under these storage and transport conditions. Despite these efforts, the proteins were undetectable and may be explained by their lack of secretion into the extracellular space. Nevertheless, our findings highlight that these genes may be involved in the molecular mechanisms underlying SCAR. In line with this interpretation, external validation using the PROMAD atlas^33^ confirmed that all four genes were also upregulated in biopsies with overt acute rejection, indicating that this molecular signature reflects core inflammatory pathways shared between subclinical and clinically manifest rejection and represent early indicators of allograft injury that continue to be expressed across different stages of rejection.

Next, we applied a multi-step strategy to our transcriptomic data, expanding our differential gene expression analysis under less stringent criteria (FDR < 0.05, log₂FC > 1.5), which yielded over 14,000 candidate genes. To narrow this extensive list, we used IPA to focus on cytokines secreted into the extracellular space and performed a tissue-specific expression screening via the Human Protein Atlas on kidney that exhibited the highest fold changes in kidney tissue. This process highlighted two key cytokines: CXCL10 and FasL, both known to be important molecular markers in immune-mediated graft injury^35,36^.

Although the gene signature composed of *NFKBIZ, TNFSF14, SLAMF8* and *CD247* and the extracellular biomarkers *CXCL10* and *FasL* were identified using two different filtering strategies, these markers can be positioned within a common immunopathological framework. NFKBIZ modulates NF-κB–dependent transcription^49^, a central upstream pathway involved in the inducible expression of inflammatory mediators such as CXCL10^50^ and FasL^51^. CD247 reflects T-cell receptor signalling competence, which is required for FasL expression in activated cytotoxic lymphocytes, and this axis may contribute to the regulation of immune cell activation and infiltration^52^. TNFSF14 (LIGHT), a member of the TNF superfamily, has been shown to amplify renal inflammation through activation of the TLR4–MyD88–NF-κB signalling pathway. In experimental models of kidney injury, LIGHT deficiency or blockade markedly attenuated NF-κB activation, inflammatory mediator production, and immune cell infiltration, supporting its role as an upstream amplifier of inflammatory signalling in the kidney^53^. SLAMF8 further contributes by modulating innate immune activation through the TLR4 pathway in pro-inflammatory macrophages, thereby shaping the inflammatory microenvironment associated with renal allograft rejection^25^. Thus, intracellular immune activation markers and extracellular effector molecules represent complementary, non-redundant layers of the same inflammatory response underlying SCAR.

To extend our analysis to a clinical setting, we assessed the detectability of CXCL10 and FasL in the urine in the transcriptomic group, as well as an independent cohort of 38 kidney transplant recipients. Urinary levels of CXCL10 and FasL were significantly elevated in SCAR patients. ROC curve analysis reinforced their diagnostic utility: CXCL10 displayed high specificity (90%) but moderate sensitivity (41%), whereas FasL achieved higher sensitivity (70%) with acceptable specificity (75%), suggesting it may serve as a more effective standalone biomarker. The integration of CXCL10 and FasL into a composite urinary signature modestly improved overall diagnostic accuracy, yielding a more balanced performance (AUC = 0.711, sensitivity = 50%, specificity = 84%) compared with either marker alone. These findings support the use of multiplex urinary biomarkers for more reliable discrimination of SCAR patients from controls and highlight the potential clinical utility of composite signatures for risk stratification.

Over the last two decades, three teams of researchers from three different countries (Canada, France and Switzerland) focused mainly on the challenge of improving SCAR diagnosis by measuring certain biomarkers, principally, CXCL9 and CXCL10 chemokines in midstream urine from patients with kidney transplantation without performing the kidney biopsy^35,54,55^. They also showed that the measurement of these cytokines should be done in absence of some confounders such as leukocyturia, urinary tract infections, and active BK-polyomavirus infection (BKPyV)^56–58^. Recently, the French team developed a fully automated urinary CXCL9 and CXCL10 immunoassay for use in the routine surveillance of kidney allograft rejection^58^. Subsequently, Van Loon et al demonstrated in a large population of kidney transplant patients that using this integrated model of urinary cytokines and clinical biomarkers for non-invasive monitoring of rejection can reduce the number of kidney biopsies performed^59^. Furthermore, Tinel et al recently developed an open access web application that uses urinary CXCL9/10 levels to calculate the risk of acute rejection in kidney transplant patients^60^.

Taken together, CXCL10 can be considered a biomarker of SCAR and other types of rejection, even in the absence of elevated serum creatinine levels^35,61,62^ and other inflammatory processes such as leukocyturia, urinary tract infections, and BK virus infection. Because strong evidence have been found by other researchers^57,63,64^, Tinel et al recently proposed to the European Society of Organ Transplantation that a next generation immunoassay be used to quantify urinary excretion of CXCL10 in association with CXCL9^60^.

Moving from the evidence that SCAR is an inflammatory process in which cytokines are involved, many researchers have measured some cytokines in the urine and confirmed their diagnostic value in cases of kidney rejection^55,61,64^.

Also, FasL is of great importance to allograft rejection. The *Fas/FasL* pathway plays a vital role in regulating immune balance, promoting self-tolerance, and preserving immune privilege in critical organs, in addition to its involvement in transplant rejection^65^. Evidence from a study involving 35 patients with delayed graft function revealed elevated levels of *FasL* mRNA in urinary cells from patients undergoing rejection compared to those without signs of rejection^66^. In a separate analysis, Heng and colleagues systematically reviewed the diagnostic value of urinary *FasL* mRNA for acute rejection (AR) following kidney transplantation. Their findings indicated a diagnostic sensitivity of 64% and specificity of 90%, with an impressive area under the ROC curve (AUC) of 0.94, suggesting strong diagnostic potential^40^. Despite these promising results, current research on the utility of *Fas* and *FasL* as biomarkers for transplant rejection remains in its early stages. Most findings to date are based on preliminary studies, and extensive validation across larger, heterogeneous patient populations is necessary before these biomarkers can be reliably integrated into routine clinical practice^67^. Here, we report for the first time an upregulation of *FasL* both at the transcriptional level in biopsy specimens and at the protein level in urine in SCAR patients.

Our study provides transcriptomic evidence linking *CXCL10* and *FasL* to the inflammatory mechanisms underlying SCAR. Importantly, we show that non-invasive measurement of these biomarkers, particularly in midstream urine samples during post-transplant follow-up, may be useful for SCAR monitoring. Urinary CXCL10 and FasL, in particular, may serve as a screening tool to identify patients at risk for SCAR, allowing biopsies to be reserved for those with elevated levels in the absence of confounding factors such as leukocyturia, urinary tract infections, BK virus infection. Furthermore, longitudinal monitoring of urinary CXCL10 and FasL following transplant may provide a practical means of assessing the evolution of SCAR. Based on our results we propose to use this combined measurement of CXCL10 and FasL as a complementary, non-invasive indicator to support clinical decision-making. The high specificity (84%) and acceptable AUC (0.71) observed in our validation cohort suggest potential utility as a confirmatory “rule-in” biomarker in patients with suspected subclinical rejection. While preliminary, our findings align with emerging evidence^68^, indicating that urine-based diagnostics may augment, and in selected scenarios, partially reduce, the reliance on protocol biopsies. The primary value of non-invasive biomarkers lies in identifying clinically stable patients who may nonetheless require a biopsy, and in monitoring treatment response^69^.

We acknowledge that excluding patients with confounding conditions (e.g., urinary tract infection, BK virus replication, leukocyturia) may limit the generalizability of our findings. However, these exclusions were intentionally applied to minimize biological noise in this pilot study and to establish a proof-of-principle under controlled conditions. Importantly, several of these conditions are known to elevate urinary inflammatory markers, and therefore could artificially increase biomarker levels; excluding these cases helped ensure that the observed associations reflected SCAR-related biology rather than infection-driven inflammation. We encourage future investigations expand on these findings by including larger and more heterogeneous patient cohorts and refining diagnostic thresholds to better assess the real-world performance of urinary CXCL10/FasL within multi-parameter, non-invasive monitoring strategies. In this context, SCAR represents a particularly delicate stage of the transplant course: histologic rejection is already present, yet renal function remains apparently stable. Detecting and monitoring SCAR through non-invasive biomarkers such as CXCL10 and FasL could, therefore, provide a critical opportunity for earlier intervention, potentially improving long-term allograft outcomes.

## Supplementary Information

Supplementary Information.

## Data Availability

The microarray data generated and analyzed in this study are MIAME-compliant and have been deposited in the Gene Expression Omnibus (GEO) under accession number GSE294632. These data are publicly accessible at [https://www.ncbi.nlm.nih.gov/geo/query/acc.cgi?acc=GSE294632]. Additional data supporting the findings of this study are available from the corresponding author upon reasonable request.
