# Supplementary material for: Integration of intragraft transcriptomics and urinary cytokines identifies CXCL10 and FasL signature in subclinical acute rejection
Source: Sci Rep. 2026 Feb 19;16:9891. doi: 10.1038/s41598-026-35923-6 (PMC13018632; doi:10.1038/s41598-026-35923-6)
Supplement: Supplementary file 1 — Supplementary Information. [file 41598_2026_35923_MOESM1_ESM.docx]

**S*upplementary data for***

**Integration of Intragraft Transcriptomics and Urinary Cytokines Identifies CXCL10 and FasL Signature in Subclinical Acute Rejection**

Sharon Natasha Cox^1$^, Samantha Chiurlia^2,3^, Emanuela Pasculli^2^, Luigi Biancone^4^, Davide Diena^4^, Vincenzo Cantaluppi^5^, Andrea Airoldi^5^, Ilaria Gandolfini^6^, Umberto Maggiore^6^, Nicola Bossini^7^, Michele Rossini,^8^ Graziano Pesole^1^ , Francesco Paolo Schena^3,8^ and the Italian SCAR Study Group*

^1^Department of Biosciences, Biotechnology and Environment, University of Bari “Aldo Moro”. Bari, Italy

^2^ University Hospital Consorziale Policlinico. Piazzale Giulio Cesare 11, Bari, 70124, Italy

^3^ Schena Foundation, Bari, Italy

^4^ Department of Medical Sciences, University of Turin, 10126, Turin, Italy.

^5^ Nephrology and Kidney Transplantation Unit, Department of Translational Medicine (DIMET), University of Piemonte Orientale (UPO), "Maggiore della Carità" University Hospital, Novara, Italy.

^6^Nephrology Unit, University Hospital of Parma, Parma, Italy.

^7^Division of Nephrology and Dialysis, ASST Spedali Civili, Brescia, Italy.

^8^Nephrology, Dialysis and Transplantation Unit, DiMePRe-J, University of Bari "Aldo Moro", Italy

**Italian SCAR Study Group**

Luigi Biancone^4^, Davide Diena^4^, Vincenzo Cantaluppi^5^, Andrea Airoldi^5^, Michele Rossini^8^, Francesco Paolo Schena^3,8^, Paolo Rigotti⁹, Giovanni Zaninotto^10^, Franco Citterio^11^, Alessia Toscano^11^, Regina Tardanico⁷

⁹ Kidney Pancreas Transplant Unit, Department of Surgery, Oncology and Gastroenterology, Padua University Hospital, Padua, Italy ;

^10^ Department of Surgery and Cancer, Imperial College London, London, UK;

^11^Renal Transplantation Unit, Department of Surgery, Catholic University of the Sacred Heart, Rome, Italy

$Corresponding authors: Sharon Natasha Cox, Department of Biosciences, Biotechnology and Environment,University of Bari “Aldo Moro”. Bari, Italy; e-mail sharonntasha.cox@uniba.it; Francesco Paolo Schena Nephrology, Dialysis and Transplantation Unit, DiMePRe-J, University of Bari "Aldo Moro", Bari, Italy email:paolo.schena@uniba.it


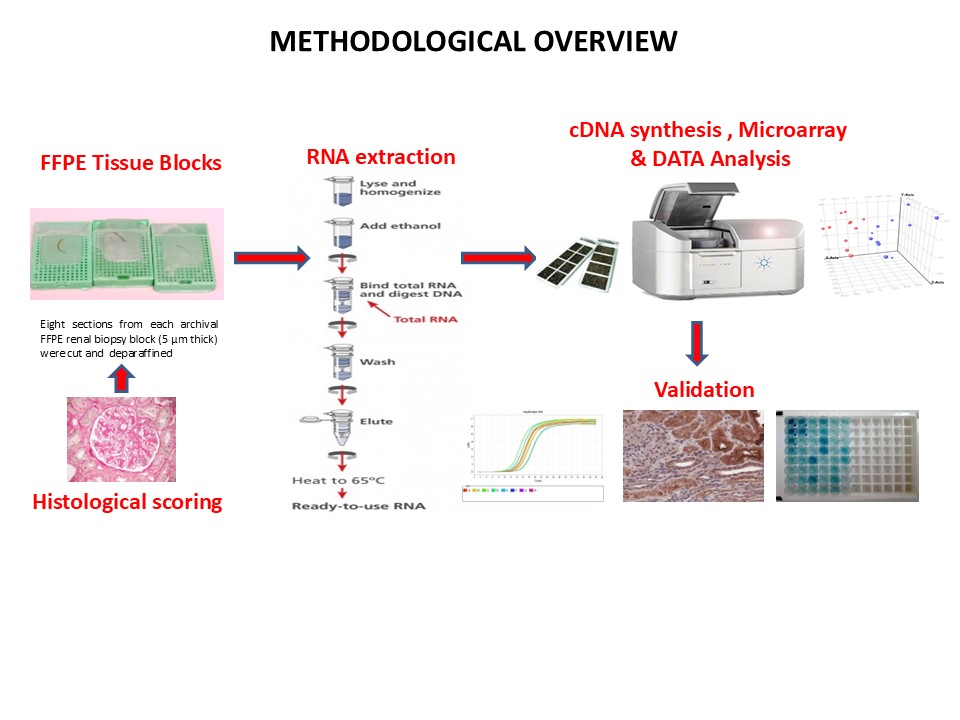


**Supplementary Figure 1** . **Methodological overview of the transcriptomic study**, starting with formalin-fixed, paraffin-embedded (FFPE) tissue blocks and histological scoring, followed by RNA extraction, cDNA synthesis, microarray-based gene expression analysis, and final validation steps


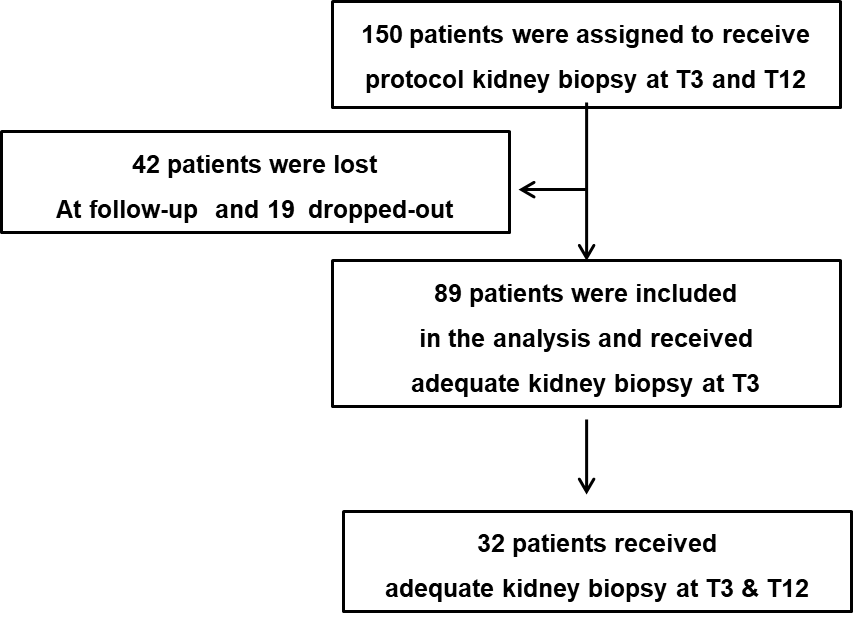


**Supplementary Figure 2.** Schematic overview of discovery cohort recruitment and follow-up

**
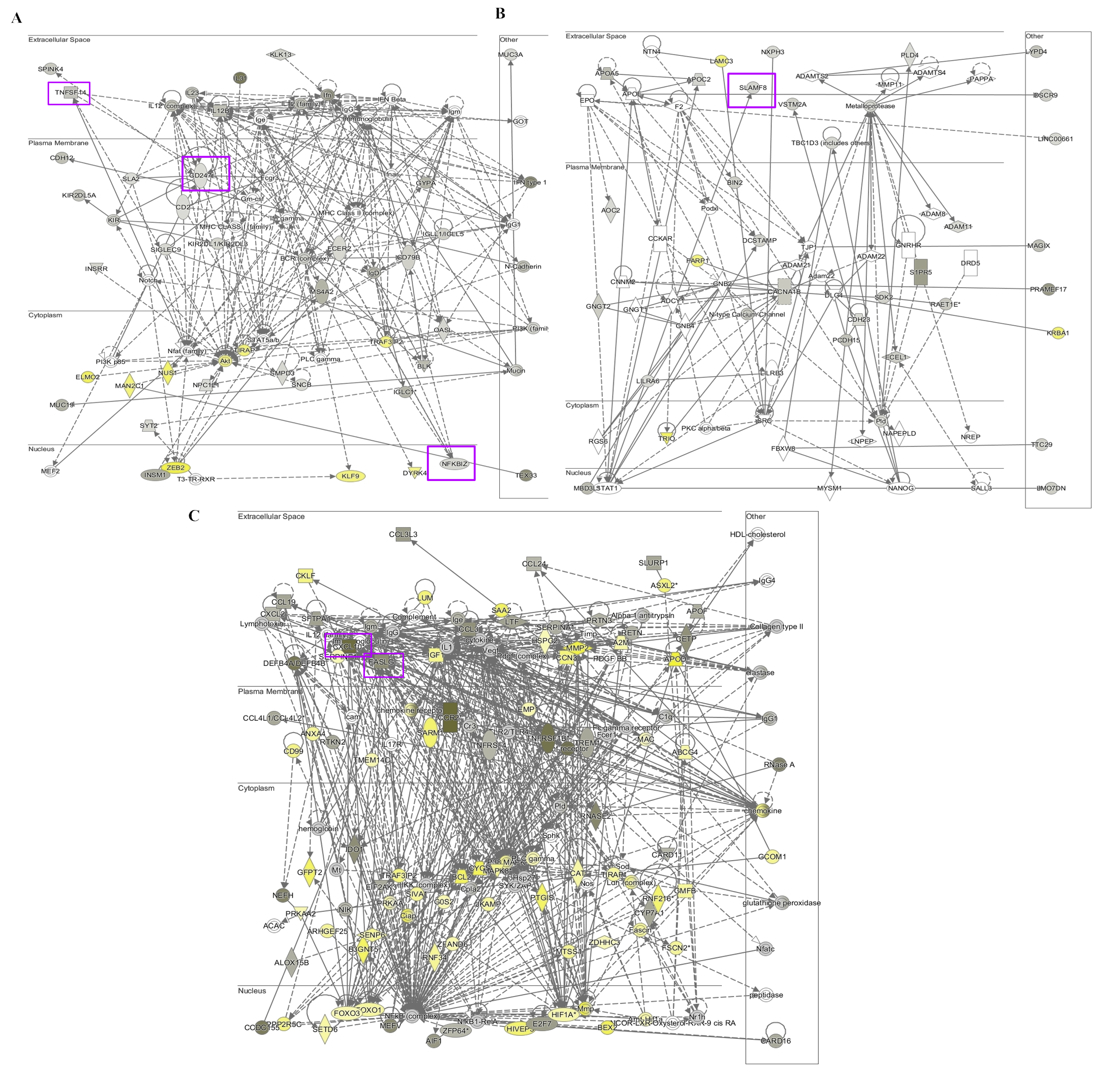
**

**Supplementary Figure 3.** **Networks generated with IPA.** Top-ranked networks generated by differentially expressed genes after uploading the 1,849 gene probe sets. Panels A (score 47, focus molecules 38) and B (score 36, focus molecules 35) show molecular interaction networks annotated by cellular localization (extracellular space, plasma membrane, cytoplasm, nucleus). Key immune-related genes associated with SCAR—NFKBIZ, SLAMF8, CD247, and TNFSF14—are highlighted in magenta. Panel C shows the network visualization generated from the 14,017 differentially expressed probe sets, with CXCL10 and FasL highlighted; both are upregulated and localized to the extracellular space, supporting their suitability as urinary biomarker candidates. All networks were processed through the color-blindness adjustment tool available at https://rgblind.com/. In the adjusted palette, yellow indicates downregulated genes, grey indicates upregulated genes, and white indicates no regulation.


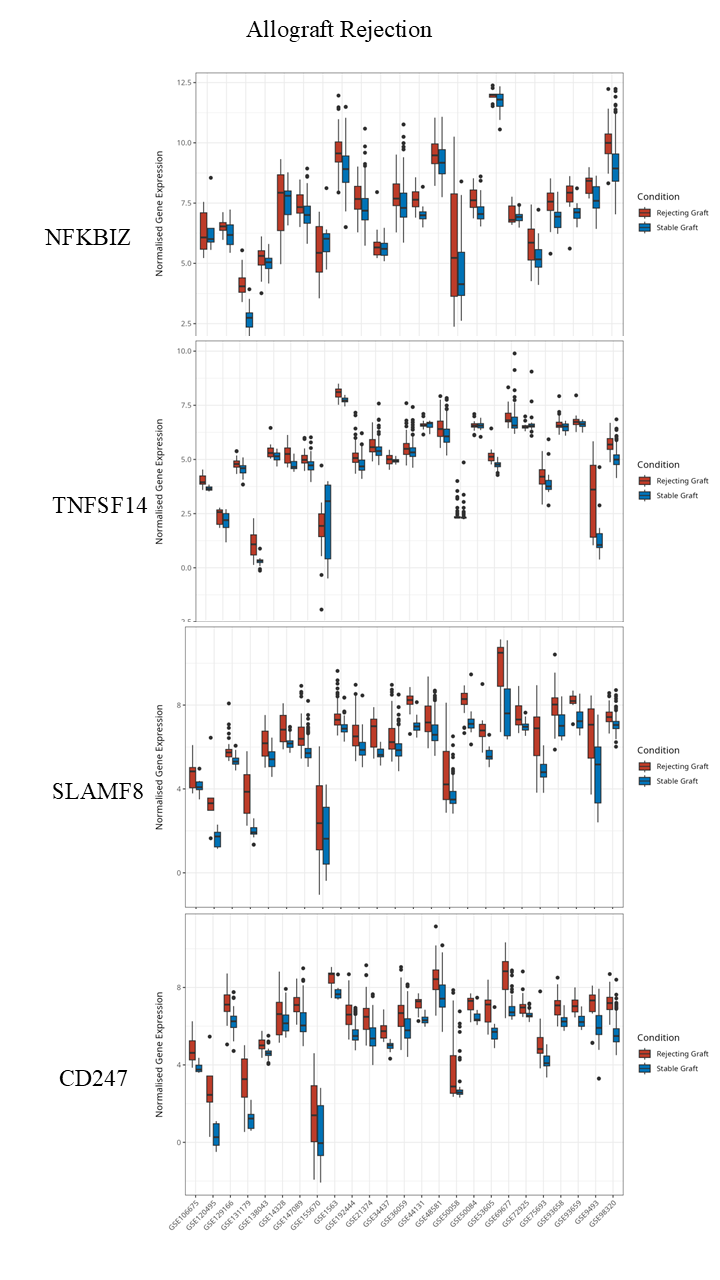


**Supplementary Figure 4. Expression of NFKBIZ, TNFSF14, SLAMF8, and CD247 in publicly available allograft rejection datasets*.*** Boxplots show normalized gene expression levels for each of the four SCAR-associated genes across multiple external cohorts included in the PROMAD atlas. Red and blue boxplots represent biopsies from rejecting grafts and stable grafts, respectively. Across datasets, all four genes consistently display higher expression in rejecting grafts, supporting the reproducibility of our findings and indicating that these genes are upregulated not only in subclinical rejection but also in overt acute allograft rejection.


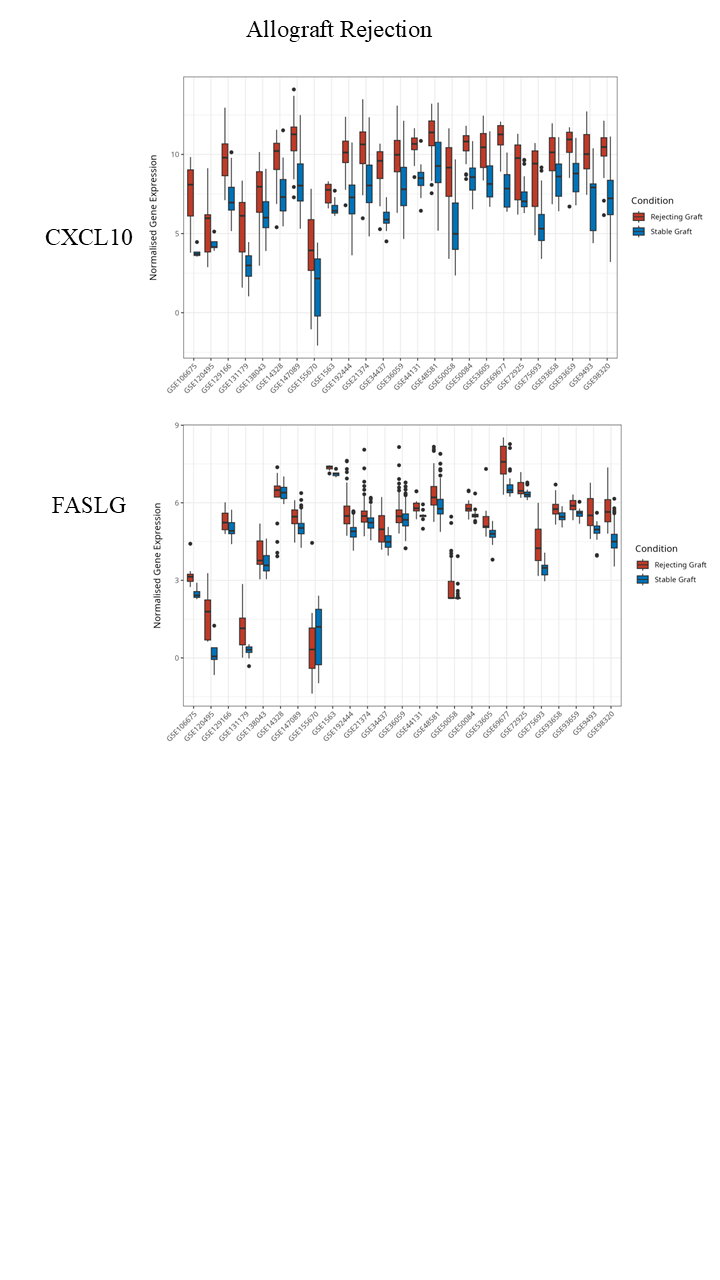


**Supplementary Figure 5. Expression of CXCL10 and FASLG in publicly available allograft rejection datasets.**

Boxplots show normalized gene expression levels for CXCL10 and FASLG across multiple external cohorts included in the PROMAD atlas. Red and blue boxplots represent biopsies from rejecting grafts and stable grafts, respectively. Across datasets, both genes consistently display higher expression in rejecting grafts, supporting their reproducibility and indicating that these inflammatory mediators are upregulated not only in subclinical rejection but also in overt acute allograft rejection.


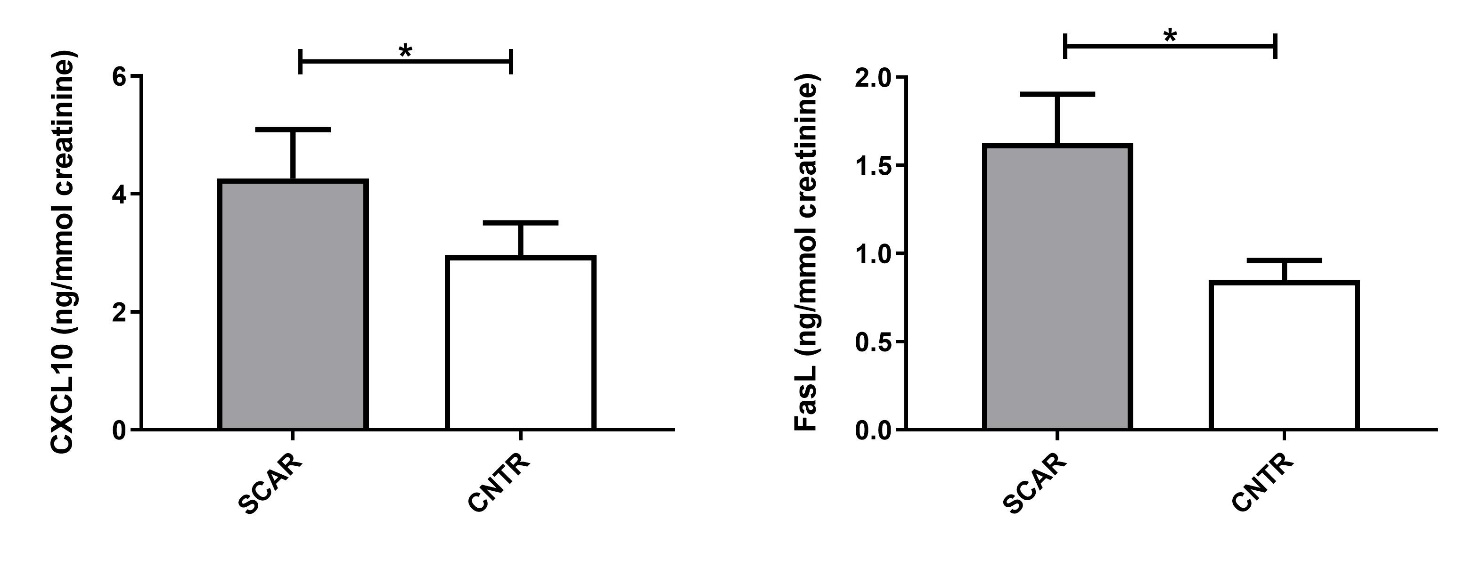


**Supplementary Figure 6** Urinary levels of CXCL10 and FasL measured by ELISA in the microarray transcriptomic cohort were significantly elevated in patients with subclinical acute rejection (SCAR) compared to control (CNTR) patients (Unpaired t-test: CXCL10, p = 0.04; FasL, p = 0.02). Results are expressed as ng/mmol creatinine

**Supplementary Table 1 .** **1,849 differentially expressed probes in Subclinical acute rejection patients compared to the Control group**

| ProbeName | p (Corr) | Log2FC | GeneSymbol | Description |
| --- | --- | --- | --- | --- |
| A_23_P52610 | 0.017965812 | -2.6385279 | DDB2 | Homo sapiens damage-specific DNA binding protein 2, 48kDa (DDB2), mRNA [NM_000107] |
| A_21_P0014207 | 0.001770209 | 2.1892323 | LOC101927721 | PREDICTED: Homo sapiens uncharacterized LOC101927721 (LOC101927721), ncRNA [XR_242321] |
| A_23_P252023 | 0.009533718 | 2.3053207 | SSX2 | Homo sapiens synovial sarcoma, X breakpoint 2 (SSX2), transcript variant 2, mRNA [NM_175698] |
| A_23_P114689 | 0.009337066 | -2.0954456 | ASAP3 | Homo sapiens ArfGAP with SH3 domain, ankyrin repeat and PH domain 3 (ASAP3), transcript variant 1, mRNA [NM_017707] |
| A_21_P0003351 | 0.016755583 | 2.6007693 |  |  |
| A_33_P3339197 | 0.004264157 | 2.8252926 |  |  |
| A_22_P00010339 | 0.014197446 | 3.3356202 | lnc-MYBPH-2 | LNCipedia lincRNA (lnc-MYBPH-2), lincRNA [lnc-MYBPH-2:1] |
| A_22_P00023281 | 0.015903948 | 2.8547454 | SIRPG-AS1 | Homo sapiens SIRPG antisense RNA 1 (SIRPG-AS1), long non-coding RNA [NR_110090] |
| A_21_P0007107 | 0.006112956 | 2.8377035 |  | PREDICTED: Homo sapiens uncharacterized LOC102723350 (LOC102723350), ncRNA [XR_424136] |
| A_22_P00009713 | 0.001489491 | 7.6210012 | lnc-MCCC2-2 | LNCipedia lincRNA (lnc-MCCC2-2), lincRNA [lnc-MCCC2-2:2] |
| A_22_P00002385 | 0.011555376 | 2.300446 |  |  |
| A_22_P00008912 | 0.002772977 | 3.5654042 | LOC101928100 | Homo sapiens uncharacterized LOC101928100 (LOC101928100), long non-coding RNA [NR_120430] |
| A_22_P00017015 | 0.003729382 | 2.6381435 | lnc-TTC35-3 | BX111153 Soares_NFL_T_GBC_S1 Homo sapiens cDNA clone IMAGp998I074001, mRNA sequence [BX111153] |
| A_33_P3821494 | 0.008033049 | 2.1770506 | MAPK8IP2 | mitogen-activated protein kinase 8 interacting protein 2 [Source:HGNC Symbol;Acc:HGNC:6883] [ENST00000008876] |
| A_33_P3339070 | 0.002472808 | 4.236274 | LINC00704 | Homo sapiens long intergenic non-protein coding RNA 704 (LINC00704), long non-coding RNA [NR_024475] |
| A_22_P00023392 | 0.013190938 | 2.0240571 | LOC100506526 | Homo sapiens uncharacterized LOC100506526 (LOC100506526), long non-coding RNA [NR_105000] |
| A_22_P00024466 | 0.014342867 | 4.078234 |  | BX110434 Soares_NFL_T_GBC_S1 Homo sapiens cDNA clone IMAGp998A165209, mRNA sequence [BX110434] |
| A_24_P59236 | 0.001162504 | 2.4199727 | RESP18 | Homo sapiens regulated endocrine-specific protein 18 (RESP18), mRNA [NM_001007089] |
| A_33_P3285570 | 0.010646765 | 2.4308393 |  | DA194689 BRAMY3 Homo sapiens cDNA clone BRAMY3019176 5', mRNA sequence [DA194689] |
| A_33_P3251065 | 3.38E-04 | 2.9008393 | KLK13 | kallikrein-related peptidase 13 [Source:HGNC Symbol;Acc:HGNC:6361] [ENST00000602090] |
| A_33_P3401169 | 0.007743884 | 2.0133097 |  | T cell receptor beta variable 21/OR9-2 (pseudogene) [Source:HGNC Symbol;Acc:HGNC:12199] [ENST00000331828] |
| A_21_P0003339 | 0.017480576 | 2.3639011 |  |  |
| A_22_P00002641 | 0.013551726 | 2.6780183 | lnc-C1orf186-1 | EST643 human nasopharynx Homo sapiens cDNA, mRNA sequence [CD684123] |
| A_22_P00010273 | 0.002847896 | 2.8922532 |  |  |
| A_21_P0010679 | 0.011191177 | 3.1119401 | XLOC_l2_001362 | BROAD Institute lincRNA (XLOC_l2_001362), lincRNA [TCONS_l2_00001843] |
| A_22_P00025517 | 0.011319919 | 2.0713594 | TLX1NB | TLX1 neighbor [Source:HGNC Symbol;Acc:HGNC:37183] [ENST00000425505] |
| A_22_P00006710 | 0.015121243 | 2.2233334 | HGC6.3 | Homo sapiens uncharacterized LOC100128124 (HGC6.3), mRNA [NM_001129895] |
| A_33_P3321263 | 0.011365542 | 4.8365264 |  | olfactory receptor, family 4, subfamily G, member 2 pseudogene [Source:HGNC Symbol;Acc:HGNC:8303] [ENST00000328113] |
| A_23_P6596 | 0.015773153 | -2.5570471 | HES1 | Homo sapiens hes family bHLH transcription factor 1 (HES1), mRNA [NM_005524] |
| A_33_P3278755 | 0.013511646 | 4.2031965 | SULT6B1 | Homo sapiens sulfotransferase family, cytosolic, 6B, member 1 (SULT6B1), mRNA [NM_001032377] |
| A_23_P88222 | 0.005823871 | 2.0299335 | PLD4 | Homo sapiens phospholipase D family, member 4 (PLD4), mRNA [NM_138790] |
| A_23_P133536 | 0.004280046 | 2.444582 | CAPSL | Homo sapiens calcyphosine-like (CAPSL), transcript variant 1, mRNA [NM_144647] |
| A_33_P3301884 | 0.00210958 | 2.5979123 | LINC01257 | Homo sapiens long intergenic non-protein coding RNA 1257 (LINC01257), long non-coding RNA [NR_026670] |
| A_21_P0013242 | 0.002130372 | 3.5655892 | XLOC_l2_013484 | BROAD Institute lincRNA (XLOC_l2_013484), lincRNA [TCONS_l2_00026028] |
| A_21_P0007883 | 0.009337066 | 3.2431498 | lnc-BEST3-1 | LNCipedia lincRNA (lnc-BEST3-1), lincRNA [lnc-BEST3-1:8] |
| A_33_P3252279 | 0.008260069 | 2.0368981 |  |  |
| A_21_P0009437 | 8.68E-04 | 5.570507 | lnc-ADCYAP1-1 | LNCipedia lincRNA (lnc-ADCYAP1-1), lincRNA [lnc-ADCYAP1-1:1] |
| A_33_P3269423 | 0.012329591 | 5.4569736 | ATP11AUN | Homo sapiens ATP11A upstream neighbor (ATP11AUN), mRNA [NM_207440] |
| A_21_P0000910 | 0.002613231 | 2.3765287 | lnc-MIB2-1 | LNCipedia lincRNA (lnc-MIB2-1), lincRNA [lnc-MIB2-1:5] |
| A_33_P3412463 | 0.007746431 | 2.9689798 | PLEKHS1 | Homo sapiens pleckstrin homology domain containing, family S member 1 (PLEKHS1), transcript variant 2, mRNA [NM_001193434] |
| A_33_P3233906 | 0.01306392 | -2.6561704 | RAMP1 | Homo sapiens receptor (G protein-coupled) activity modifying protein 1 (RAMP1), mRNA [NM_005855] |
| ERCC-00002_129 | 7.17E-04 | 2.1518288 |  |  |
| A_21_P0013137 | 0.017546525 | 2.9976234 |  | t-complex 10-like 2 [Source:HGNC Symbol;Acc:HGNC:21254] [ENST00000473271] |
| A_23_P109184 | 0.001729655 | 5.051962 | INSM1 | Homo sapiens insulinoma-associated 1 (INSM1), mRNA [NM_002196] |
| A_21_P0005634 | 0.008821026 | 2.8746085 | LOC101927657 | Homo sapiens uncharacterized LOC101927657 (LOC101927657), long non-coding RNA [NR_125421] |
| A_33_P3415560 | 0.019126587 | 2.0435686 | ZFP82 | Homo sapiens ZFP82 zinc finger protein (ZFP82), mRNA [NM_133466] |
| A_23_P127978 | 9.74E-04 | 7.361301 | B3GNT6 | Homo sapiens UDP-GlcNAc:betaGal beta-1,3-N-acetylglucosaminyltransferase 6 (core 3 synthase) (B3GNT6), mRNA [NM_138706] |
| A_33_P3369979 | 0.007371812 | 3.8328698 | OR2L2 | Homo sapiens olfactory receptor, family 2, subfamily L, member 2 (OR2L2), mRNA [NM_001004686] |
| A_23_P404059 | 0.008130088 | 3.5046833 | PASD1 | Homo sapiens PAS domain containing 1 (PASD1), mRNA [NM_173493] |
| A_24_P786713 | 0.017632438 | 3.1469884 |  |  |
| A_33_P3355508 | 0.007825878 | 2.7979748 | FOXL2 | Homo sapiens forkhead box L2 (FOXL2), mRNA [NM_023067] |
| A_22_P00010900 | 0.010394448 | 3.600132 |  |  |
| A_33_P3338397 | 0.009233085 | 2.9611638 | LOC100128651 | Homo sapiens cDNA FLJ45310 fis, clone BRHIP3004774. [AK127243] |
| A_19_P00320875 | 0.009892808 | 2.50085 |  |  |
| A_22_P00021307 | 0.018644402 | 2.1999166 | LINC00867 | long intergenic non-protein coding RNA 867 [Source:HGNC Symbol;Acc:HGNC:45265] [ENST00000415417] |
| A_21_P0002008 | 0.017281646 | 2.1867886 | LINC00298 | long intergenic non-protein coding RNA 298 [Source:HGNC Symbol;Acc:HGNC:49257] [ENST00000456681] |
| A_33_P3686706 | 0.014963093 | 4.8803043 |  | Q73KV7_TREDE (Q73KV7) Orotidine 5'-phosphate decarboxylase , partial (6%) [THC2638457] |
| A_33_P3278137 | 2.92E-04 | 4.693823 | RPL31P11 | Homo sapiens ribosomal protein L31 pseudogene 11 (RPL31P11), non-coding RNA [NR_002595] |
| A_22_P00008943 | 0.016285634 | 2.1635935 | KRT81 | Homo sapiens keratin 81, type II (KRT81), mRNA [NM_002281] |
| A_33_P3297160 | 0.01676205 | 3.640647 |  | olfactory receptor, family 8, subfamily V, member 1 pseudogene [Source:HGNC Symbol;Acc:HGNC:19613] [ENST00000395193] |
| A_21_P0009741 | 0.018723087 | 2.669487 | lnc-DDX39A-2 | LNCipedia lincRNA (lnc-DDX39A-2), lincRNA [lnc-DDX39A-2:1] |
| A_21_P0006306 | 0.016431227 | 2.5029066 | SMC2-AS1 | Homo sapiens SMC2 antisense RNA 1 (head to head) (SMC2-AS1), long non-coding RNA [NR_121580] |
| A_23_P415395 | 6.53E-04 | 4.122371 | MBD3L1 | Homo sapiens methyl-CpG binding domain protein 3-like 1 (MBD3L1), mRNA [NM_145208] |
| A_21_P0014442 | 0.01601842 | 2.7412646 | LOC100507306 | PREDICTED: Homo sapiens uncharacterized LOC100507306 (LOC100507306), transcript variant X2, ncRNA [XR_251642] |
| A_21_P0003136 | 0.003624071 | 2.5735896 | lnc-VGLL3-1 | LNCipedia lincRNA (lnc-VGLL3-1), lincRNA [lnc-VGLL3-1:1] |
| A_22_P00007371 | 0.009864151 | 2.290636 | lnc-GREM2-7 | LNCipedia lincRNA (lnc-GREM2-7), lincRNA [lnc-GREM2-7:1] |
| A_23_P126031 | 0.013680195 | 2.6103067 | OR10J5 | Homo sapiens olfactory receptor, family 10, subfamily J, member 5 (OR10J5), mRNA [NM_001004469] |
| A_22_P00006825 | 0.012612618 | 2.2746818 | lnc-GABRB2-1 | RST16091 Athersys RAGE Library Homo sapiens cDNA, mRNA sequence [BG196864] |
| A_21_P0005045 | 0.016327148 | 2.4178445 | lnc-ASCC3-1 | LNCipedia lincRNA (lnc-ASCC3-1), lincRNA [lnc-ASCC3-1:1] |
| A_21_P0012665 | 0.011397219 | 2.477046 |  |  |
| A_21_P0009456 | 0.016431227 | 2.889922 | lnc-SLC35G4-2 | LNCipedia lincRNA (lnc-SLC35G4-2), lincRNA [lnc-SLC35G4-2:1] |
| A_22_P00020933 | 0.012143905 | 2.111863 |  |  |
| A_33_P3266609 | 0.006038472 | 2.0267956 |  |  |
| A_21_P0004906 | 0.017040258 | 2.2608693 | lnc-AL079342.1-1 | LNCipedia lincRNA (lnc-AL079342.1-1), lincRNA [lnc-AL079342.1-1:1] |
| A_33_P3317790 | 0.017081076 | 3.443115 | LCN1 | Homo sapiens lipocalin 1 (LCN1), transcript variant 4, mRNA [NM_001252619] |
| A_22_P00012554 | 0.012055238 | 2.0904918 |  | long intergenic non-protein coding RNA 604 [Source:HGNC Symbol;Acc:HGNC:43924] [ENST00000508437] |
| A_21_P0008211 | 0.005823871 | 2.9657345 | lnc-EFNB2-1 | LNCipedia lincRNA (lnc-EFNB2-1), lincRNA [lnc-EFNB2-1:1] |
| A_22_P00001937 | 0.003686147 | 3.015266 | lnc-BARHL1-1 | BC006231 IKBKB protein {Homo sapiens} (exp=-1; wgp=0; cg=0), partial (5%) [THC2687634] |
| A_32_P153773 | 0.016114652 | 3.1275098 | CACNA1B | Homo sapiens calcium channel, voltage-dependent, N type, alpha 1B subunit (CACNA1B), transcript variant 1, mRNA [NM_000718] |
| A_33_P3221403 | 0.016174048 | 3.0121634 | LOC646743 | Homo sapiens clone TESTIS-608 mRNA sequence. [AY726562] |
| A_22_P00022710 | 0.007424729 | 2.0866404 |  |  |
| A_21_P0008628 | 0.005789592 | 4.2759757 | PLA2G4E-AS1 | Homo sapiens PLA2G4E antisense RNA 1 (PLA2G4E-AS1), long non-coding RNA [NR_120334] |
| A_23_P120594 | 0.010031875 | -2.7339776 | ACSS1 | Homo sapiens acyl-CoA synthetase short-chain family member 1 (ACSS1), transcript variant 1, mRNA [NM_032501] |
| A_33_P3374947 | 0.003963198 | 2.3539298 | KLF14 | Homo sapiens Kruppel-like factor 14 (KLF14), mRNA [NM_138693] |
| A_21_P0006230 | 0.007796347 | 3.8646255 | lnc-C9orf16-1 | LNCipedia lincRNA (lnc-C9orf16-1), lincRNA [lnc-C9orf16-1:2] |
| A_33_P3292719 | 0.002277691 | 2.5254557 | PTX4 | Homo sapiens pentraxin 4, long (PTX4), mRNA [NM_001013658] |
| A_23_P207201 | 0.005949104 | 2.6945393 | CD79B | Homo sapiens CD79b molecule, immunoglobulin-associated beta (CD79B), transcript variant 3, mRNA [NM_001039933] |
| A_33_P3331952 | 0.001452869 | 11.569694 | OR2M3 | Homo sapiens olfactory receptor, family 2, subfamily M, member 3 (OR2M3), mRNA [NM_001004689] |
| A_33_P3277183 | 0.003061198 | 3.3687375 | SSPO | SCO-spondin [Source:HGNC Symbol;Acc:HGNC:21998] [ENST00000475488] |
| A_22_P00016943 | 0.017540125 | 2.0848043 | LOC101927447 | PREDICTED: Homo sapiens uncharacterized LOC101927447 (LOC101927447), ncRNA [XR_244442] |
| A_23_P212639 | 0.011723607 | -2.1338167 | TRA2B | Homo sapiens transformer 2 beta homolog (Drosophila) (TRA2B), transcript variant 1, mRNA [NM_004593] |
| A_21_P0009480 | 0.016004086 | 2.1508222 | lnc-DCC-1 | LNCipedia lincRNA (lnc-DCC-1), lincRNA [lnc-DCC-1:5] |
| A_23_P101564 | 0.008900492 | 2.9916732 | FGF21 | Homo sapiens fibroblast growth factor 21 (FGF21), mRNA [NM_019113] |
| A_21_P0003476 | 0.009899479 | 2.5100648 |  |  |
| A_22_P00022943 | 0.003922251 | 4.8863454 |  |  |
| A_19_P00322017 | 0.002174302 | 4.1451397 | LINC00322 | Homo sapiens long intergenic non-protein coding RNA 322 (LINC00322), long non-coding RNA [NR_103713] |
| A_23_P31755 | 0.015121243 | 3.2930033 | CRH | Homo sapiens corticotropin releasing hormone (CRH), mRNA [NM_000756] |
| A_33_P3229552 | 0.017264105 | 2.5057015 | LRFN1 | Homo sapiens leucine rich repeat and fibronectin type III domain containing 1 (LRFN1), mRNA [NM_020862] |
| A_33_P3376419 | 0.019951195 | 4.0062246 | lnc-ST6GALNAC5-1 | ik22g01.x6 HR85 islet Homo sapiens cDNA clone IMAGE:5781697 3', mRNA sequence [CK825693] |
| A_21_P0007028 | 5.71E-04 | 2.3057544 | lnc-CPXM2-1 | LNCipedia lincRNA (lnc-CPXM2-1), lincRNA [lnc-CPXM2-1:2] |
| A_21_P0001766 | 7.31E-04 | 2.0462968 | LINC01121 | Homo sapiens long intergenic non-protein coding RNA 1121 (LINC01121), long non-coding RNA [NR_033831] |
| A_33_P3368991 | 0.004458424 | 3.5471373 | MPZ | Homo sapiens myelin protein zero (MPZ), mRNA [NM_000530] |
| A_22_P00002237 | 0.004883347 | 2.050491 | lnc-C10orf11-3 | MUSMSLO {Mus musculus} (exp=-1; wgp=0; cg=0), partial (6%) [THC2664159] |
| A_21_P0004134 | 0.008900492 | 3.5688841 | LOC101928769 | Homo sapiens uncharacterized LOC101928769 (LOC101928769), long non-coding RNA [NR_105001] |
| A_23_P353149 | 0.005204129 | 7.054948 | TEX33 | Homo sapiens testis expressed 33 (TEX33), transcript variant 2, mRNA [NM_178552] |
| A_33_P3435493 | 0.015845079 | 3.1978953 | LOC400768 | PREDICTED: Homo sapiens uncharacterized LOC400768 (LOC400768), misc_RNA [XR_110483] |
| A_23_P414273 | 0.01928657 | -2.4559307 | SMIM3 | Homo sapiens small integral membrane protein 3 (SMIM3), mRNA [NM_032947] |
| A_33_P3405043 | 0.002130372 | 6.7883315 |  |  |
| A_22_P00003747 | 0.001854229 | 2.9082296 |  | DA764966 NTONG2 Homo sapiens cDNA clone NTONG2005643 5', mRNA sequence [DA764966] |
| A_21_P0003038 | 0.002001687 | 2.2394261 | lnc-CHST2-5 | LNCipedia lincRNA (lnc-CHST2-5), lincRNA [lnc-CHST2-5:1] |
| A_33_P3405535 | 0.012864207 | 3.3604383 | LOC650293 | Homo sapiens seven transmembrane helix receptor (LOC650293), mRNA [NM_001040071] |
| A_21_P0005230 | 0.003686147 | 2.5819242 |  | CR737490 Soares_testis_NHT Homo sapiens cDNA clone IMAGp971C1779 ; IMAGE:1839330 5', mRNA sequence [CR737490] |
| A_23_P11103 | 7.24E-04 | 4.0747404 | BMP15 | Homo sapiens bone morphogenetic protein 15 (BMP15), mRNA [NM_005448] |
| A_21_P0006942 | 0.017546525 | 2.0068734 | lnc-GPR123-1 | LNCipedia lincRNA (lnc-GPR123-1), lincRNA [lnc-GPR123-1:1] |
| A_19_P00322605 | 0.004204114 | 2.9118502 | LINC01013 | long intergenic non-protein coding RNA 1013 [Source:HGNC Symbol;Acc:HGNC:48987] [ENST00000458028] |
| A_33_P3319905 | 0.012386669 | 2.2527502 | TREM1 | Homo sapiens triggering receptor expressed on myeloid cells 1 (TREM1), transcript variant 1, mRNA [NM_018643] |
| A_22_P00011265 | 0.006416088 | 3.0935347 | LOC101927079 | Homo sapiens uncharacterized LOC101927079 (LOC101927079), transcript variant 1, long non-coding RNA [NR_110480] |
| A_21_P0008897 | 0.004018391 | 4.7856665 | LINC00922 | long intergenic non-protein coding RNA 922 [Source:HGNC Symbol;Acc:HGNC:27545] [ENST00000564041] |
| A_22_P00012731 | 0.003922251 | 4.1466293 | lnc-RAB6C-3 | Q5XJH7_MOUSE (Q5XJH7) Adck4 protein, partial (5%) [THC2658709] |
| A_23_P44781 | 0.014990651 | -3.029127 | CDKAL1 | Homo sapiens CDK5 regulatory subunit associated protein 1-like 1 (CDKAL1), mRNA [NM_017774] |
| A_23_P123330 | 0.010515429 | -3.1593626 | RPL30 | Homo sapiens ribosomal protein L30 (RPL30), mRNA [NM_000989] |
| A_23_P168551 | 0.016285634 | 2.621546 | SLC29A4 | Homo sapiens solute carrier family 29 (equilibrative nucleoside transporter), member 4 (SLC29A4), transcript variant 1, mRNA [NM_001040661] |
| A_19_P00320038 | 0.01439562 | 3.788167 | LINC00574 | Homo sapiens long intergenic non-protein coding RNA 574 (LINC00574), long non-coding RNA [NR_026780] |
| A_19_P00322837 | 0.014599714 | 3.7087808 | LINC01470 | long intergenic non-protein coding RNA 1470 [Source:HGNC Symbol;Acc:HGNC:51105] [ENST00000503048] |
| A_23_P143173 | 0.008302389 | 2.1038837 | SLA2 | Homo sapiens Src-like-adaptor 2 (SLA2), transcript variant 1, mRNA [NM_032214] |
| A_33_P3364954 | 0.005823871 | 3.967214 |  | GB |
| A_22_P00021757 | 0.013622328 | 2.8472161 |  |  |
| A_22_P00006568 | 0.00654853 | 3.6381702 | LOC100131315 | Homo sapiens uncharacterized LOC100131315 (LOC100131315), long non-coding RNA [NR_109767] |
| A_21_P0005640 | 0.002373447 | 3.1129353 | lnc-FBXO25-3 | LNCipedia lincRNA (lnc-FBXO25-3), lincRNA [lnc-FBXO25-3:1] |
| A_21_P0003845 | 0.002719358 | 3.8240068 | lnc-AIMP1-1 | LNCipedia lincRNA (lnc-AIMP1-1), lincRNA [lnc-AIMP1-1:8] |
| A_22_P00024850 | 0.012357703 | 2.4308703 | lnc-NOC3L-2 | LNCipedia lincRNA (lnc-NOC3L-2), lincRNA [lnc-NOC3L-2:1] |
| A_23_P31143 | 0.006433252 | -2.169811 | TPD52L1 | Homo sapiens tumor protein D52-like 1 (TPD52L1), transcript variant 2, mRNA [NM_001003395] |
| A_24_P702813 | 0.007883344 | -2.6641345 | XPR1 | Homo sapiens xenotropic and polytropic retrovirus receptor 1 (XPR1), transcript variant 1, mRNA [NM_004736] |
| A_21_P0005496 | 1.16E-04 | 6.592643 | lnc-ZPBP-3 | LNCipedia lincRNA (lnc-ZPBP-3), lincRNA [lnc-ZPBP-3:1] |
| A_32_P810645 | 0.006269174 | 6.014768 | FLJ40288 | Homo sapiens uncharacterized FLJ40288 (FLJ40288), long non-coding RNA [NR_046323] |
| A_33_P3224206 | 0.001167695 | 4.0772557 | lnc-ALX3-1 | LNCipedia lincRNA (lnc-ALX3-1), lincRNA [lnc-ALX3-1:1] |
| A_23_P142856 | 0.003624071 | 2.4040809 | AQP12A | Homo sapiens aquaporin 12A (AQP12A), mRNA [NM_198998] |
| A_33_P3260563 | 0.001574271 | 2.2531486 | SOX30 | Homo sapiens SRY (sex determining region Y)-box 30 (SOX30), transcript variant 2, mRNA [NM_007017] |
| A_33_P3239228 | 0.018622266 | 2.363382 | MUC3A | Homo sapiens mucin 3A, cell surface associated (MUC3A), mRNA [NM_005960] |
| A_23_P8253 | 0.006028791 | 3.198551 | RAET1E | Homo sapiens retinoic acid early transcript 1E (RAET1E), transcript variant 1, mRNA [NM_139165] |
| A_21_P0008258 | 0.001729655 | 3.5734231 | LINC00376 | Homo sapiens long intergenic non-protein coding RNA 376 (LINC00376), long non-coding RNA [NR_126409] |
| A_23_P124402 | 0.002092737 | 5.751573 | HTR3C | Homo sapiens 5-hydroxytryptamine (serotonin) receptor 3C, ionotropic (HTR3C), mRNA [NM_130770] |
| A_22_P00008807 | 0.01693474 | 2.2419608 | lnc-KLC1-1 | LNCipedia lincRNA (lnc-KLC1-1), lincRNA [lnc-KLC1-1:1] |
| A_33_P3383246 | 0.016247164 | 2.2608802 | OR52R1 | Homo sapiens olfactory receptor, family 52, subfamily R, member 1 (gene/pseudogene) (OR52R1), mRNA [NM_001005177] |
| A_33_P3290011 | 0.017281646 | 2.3460617 | LHFPL3 | Homo sapiens lipoma HMGIC fusion partner-like 3 (LHFPL3), mRNA [NM_199000] |
| A_21_P0009414 | 0.012794371 | 3.268787 | lnc-BTBD17-4 | LNCipedia lincRNA (lnc-BTBD17-4), lincRNA [lnc-BTBD17-4:7] |
| A_33_P3691758 | 0.008470241 | 5.5933633 |  | olfactory receptor, family 4, subfamily C, member 1 pseudogene [Source:HGNC Symbol;Acc:HGNC:8292] [ENST00000530880] |
| A_21_P0006930 | 3.24E-04 | 4.466655 | lnc-GPR26-4 | LNCipedia lincRNA (lnc-GPR26-4), lincRNA [lnc-GPR26-4:11] |
| A_22_P00008999 | 0.005789592 | 4.397153 |  | 6450952H1 BRAINOC01 Homo sapiens cDNA clone 6450952 5', mRNA sequence [BU584695] |
| A_24_P291588 | 2.93E-04 | -2.4131007 | DVL3 | Homo sapiens dishevelled segment polarity protein 3 (DVL3), mRNA [NM_004423] |
| A_21_P0005520 | 0.015294367 | 2.4444168 | lnc-RELN-1 | LNCipedia lincRNA (lnc-RELN-1), lincRNA [lnc-RELN-1:1] |
| A_22_P00018902 | 0.016285634 | 2.0898776 | lnc-C11orf39-3 | DA375949 BRTHA2 Homo sapiens cDNA clone BRTHA2007423 5', mRNA sequence [DA375949] |
| A_21_P0005791 | 0.004326516 | 4.091929 | lnc-ANGPT2-5 | LNCipedia lincRNA (lnc-ANGPT2-5), lincRNA [lnc-ANGPT2-5:1] |
| A_32_P204376 | 0.004429192 | 3.2949812 | ANKRD20A2 | Homo sapiens ankyrin repeat domain 20 family, member A2 (ANKRD20A2), mRNA [NM_001012421] |
| A_22_P00014271 | 0.001585742 | 2.0943696 | DLG1-AS1 | Homo sapiens DLG1 antisense RNA 1 (DLG1-AS1), long non-coding RNA [NR_038289] |
| A_22_P00014176 | 0.004270142 | -2.1160815 | lnc-SDCCAG8-1 | RST40535 Athersys RAGE Library Homo sapiens cDNA, mRNA sequence [BG220746] |
| A_23_P615 | 0.018820966 | 2.6140888 | INSRR | Homo sapiens insulin receptor-related receptor (INSRR), mRNA [NM_014215] |
| A_24_P357535 | 0.01569922 | 7.3995123 | OR7A10 | Homo sapiens olfactory receptor, family 7, subfamily A, member 10 (OR7A10), mRNA [NM_001005190] |
| A_21_P0001378 | 0.004264157 | 2.362513 | lnc-CD1D-1 | LNCipedia lincRNA (lnc-CD1D-1), lincRNA [lnc-CD1D-1:1] |
| A_33_P3311373 | 0.017887626 | 3.4425101 | LOC100506747 | Homo sapiens alpha-1,3-mannosyl-glycoprotein 4-beta-N-acetylglucosaminyltransferase-like protein LOC641515 homolog (LOC100506747), non-coding RNA [NR_036557] |
| A_33_P3364060 | 0.01688473 | 2.298658 | HR | Homo sapiens hair growth associated (HR), transcript variant 1, mRNA [NM_005144] |
| A_21_P0005918 | 0.018319929 | 2.853408 | lnc-PLEC-1 | LNCipedia lincRNA (lnc-PLEC-1), lincRNA [lnc-PLEC-1:1] |
| A_33_P3266489 | 0.014171607 | 2.616613 | OR13H1 | Homo sapiens olfactory receptor, family 13, subfamily H, member 1 (OR13H1), mRNA [NM_001004486] |
| A_24_P115529 | 0.002024585 | 3.6789355 | LOC51145 | Homo sapiens uncharacterized LOC51145 (LOC51145), long non-coding RNA [NR_122032] |
| A_23_P354297 | 0.019243294 | -2.3791418 | CHTF18 | Homo sapiens CTF18, chromosome transmission fidelity factor 18 homolog (S. cerevisiae) (CHTF18), mRNA [NM_022092] |
| A_22_P00012642 | 0.01965474 | 4.4128823 | LOC101927766 | Homo sapiens uncharacterized LOC101927766 (LOC101927766), long non-coding RNA [NR_109891] |
| A_22_P00008819 | 0.018736346 | 4.259276 | CCDC37-AS1 | Homo sapiens CCDC37 antisense RNA 1 (head to head) (CCDC37-AS1), transcript variant 1, long non-coding RNA [NR_103787] |
| A_23_P108554 | 0.014342867 | -2.7504637 | DDX1 | Homo sapiens DEAD (Asp-Glu-Ala-Asp) box helicase 1 (DDX1), mRNA [NM_004939] |
| A_22_P00002898 | 0.006629888 | 2.9333086 | LINC01207 | Homo sapiens long intergenic non-protein coding RNA 1207 (LINC01207), long non-coding RNA [NR_038834] |
| A_21_P0010027 | 0.009996453 | 4.5138607 | lnc-C20orf3-2 | LNCipedia lincRNA (lnc-C20orf3-2), lincRNA [lnc-C20orf3-2:1] |
| A_23_P54861 | 0.016494371 | 6.945488 | ADAM6 | Homo sapiens ADAM metallopeptidase domain 6 (pseudogene) (ADAM6), non-coding RNA [NR_002224] |
| A_33_P3366550 | 0.013680195 | 3.8680086 | LOC101929715 | Homo sapiens uncharacterized LOC101929715 (LOC101929715), long non-coding RNA [NR_110597] |
| A_22_P00004856 | 0.005923995 | 2.6268268 | lnc-CYP3A43-2 | LNCipedia lincRNA (lnc-CYP3A43-2), lincRNA [lnc-CYP3A43-2:1] |
| A_22_P00010393 | 0.010075186 | 4.1638947 |  | zt62e09.r1 Soares_testis_NHT Homo sapiens cDNA clone IMAGE:726952 5', mRNA sequence [AA401868] |
| A_22_P00004246 | 0.006797854 | 2.6630177 |  |  |
| A_23_P107795 | 0.014342867 | -3.3032234 | ATP5SL | Homo sapiens ATP5S-like (ATP5SL), transcript variant 4, mRNA [NM_018035] |
| A_22_P00014389 | 0.0121545 | 2.4170358 |  |  |
| A_24_P30557 | 0.004261388 | 5.7970104 | TBX5 | Homo sapiens T-box 5 (TBX5), transcript variant 1, mRNA [NM_000192] |
| A_22_P00007317 | 0.014342867 | 2.8640373 | LINC01146 | long intergenic non-protein coding RNA 1146 [Source:HGNC Symbol;Acc:HGNC:49467] [ENST00000557339] |
| A_22_P00005639 | 0.01693474 | 2.3035274 | lnc-EIF2AK3-4 | Q2S9U3_HAHCH (Q2S9U3) Tfp pilus assembly protein FimT, partial (8%) [THC2741911] |
| A_24_P636974 | 0.017369911 | 2.0188568 | lnc-SEPT7L-1 | LNCipedia lincRNA (lnc-SEPT7L-1), lincRNA [lnc-SEPT7L-1:1] |
| A_33_P3566559 | 0.019596959 | 2.0364087 | DNAJB8-AS1 | Homo sapiens DNAJB8 antisense RNA 1 (DNAJB8-AS1), long non-coding RNA [NR_037890] |
| A_33_P3526458 | 0.006701194 | 2.1670103 | ANKRD30BP3 | Homo sapiens hypothetical protein LOC338579, mRNA (cDNA clone IMAGE:5295086). [BC043208] |
| A_21_P0005984 | 0.003723792 | 2.3111396 | LINC01502 | Homo sapiens long intergenic non-protein coding RNA 1502 (LINC01502), transcript variant 1, long non-coding RNA [NR_034016] |
| A_24_P58488 | 0.001167695 | 5.4268527 | C2orf73 | Homo sapiens chromosome 2 open reading frame 73 (C2orf73), mRNA [NM_001100396] |
| A_23_P87902 | 0.012935037 | -2.090296 | DYRK4 | Homo sapiens dual-specificity tyrosine-(Y)-phosphorylation regulated kinase 4 (DYRK4), transcript variant 1, mRNA [NM_003845] |
| A_33_P3309792 | 0.005789592 | 4.058433 |  | TAR DNA binding protein pseudogene 1 [Source:HGNC Symbol;Acc:HGNC:16536] [ENST00000442013] |
| A_21_P0000872 | 0.001574271 | 7.44293 | LINC01005 | Homo sapiens long intergenic non-protein coding RNA 1005 (LINC01005), long non-coding RNA [NR_039987] |
| A_22_P00008079 | 7.57E-04 | 3.9598851 | lnc-IKZF2-3 | LNCipedia lincRNA (lnc-IKZF2-3), lincRNA [lnc-IKZF2-3:4] |
| A_21_P0006903 | 0.019641617 | 2.049174 | lnc-BTRC-2 | LNCipedia lincRNA (lnc-BTRC-2), lincRNA [lnc-BTRC-2:1] |
| A_23_P74895 | 0.016926367 | 2.12286 | LIN28A | Homo sapiens lin-28 homolog A (C. elegans) (LIN28A), mRNA [NM_024674] |
| A_22_P00014387 | 0.0068485 | 2.0122287 |  |  |
| A_22_P00012334 | 0.012371562 | 3.0263329 |  | DA511341 FCBBF3 Homo sapiens cDNA clone FCBBF3028405 5', mRNA sequence [DA511341] |
| A_33_P3377045 | 0.005823871 | -2.4058487 | CDC37L1 | cell division cycle 37-like 1 [Source:HGNC Symbol;Acc:HGNC:17179] [ENST00000381854] |
| A_22_P00020132 | 0.01875281 | -2.066636 | lnc-RPS4XP21-1 | LNCipedia lincRNA (lnc-RPS4XP21-1), lincRNA [lnc-RPS4XP21-1:1] |
| A_21_P0013016 | 0.003686147 | 2.7001197 | XLOC_l2_012415 | BROAD Institute lincRNA (XLOC_l2_012415), lincRNA [TCONS_l2_00024057] |
| A_23_P354805 | 0.011283191 | -2.2543576 | KLF12 | Homo sapiens Kruppel-like factor 12 (KLF12), mRNA [NM_007249] |
| A_22_P00018563 | 0.003633199 | 2.6538503 |  | DA846917 PLACE6 Homo sapiens cDNA clone PLACE6015523 5', mRNA sequence [DA846917] |
| A_33_P3343007 | 0.015461167 | 2.2292461 |  | T cell receptor alpha variable 25 [Source:HGNC Symbol;Acc:HGNC:12122] [ENST00000390454] |
| A_33_P3316239 | 0.015958356 | 2.2053688 | LOC100130419 | Homo sapiens cDNA FLJ44830 fis, clone BRACE3047018. [AK126781] |
| A_22_P00000436 | 0.015930954 | 2.7427638 |  |  |
| A_21_P0005642 | 0.005469681 | 2.884897 | lnc-DLGAP2-4 | LNCipedia lincRNA (lnc-DLGAP2-4), lincRNA [lnc-DLGAP2-4:1] |
| A_21_P0012160 | 0.018899621 | 2.1064315 |  |  |
| A_22_P00014056 | 0.008372501 | 3.7034042 |  | HA1424 Human fetal liver cDNA library Homo sapiens cDNA, mRNA sequence [AI114816] |
| A_33_P3386686 | 0.019715495 | 2.7788916 | LOC100132874 | PREDICTED: Homo sapiens uncharacterized LOC100132874 (LOC100132874), mRNA [XM_005273698] |
| A_23_P212089 | 0.009405335 | 2.4157348 | NFKBIZ | Homo sapiens nuclear factor of kappa light polypeptide gene enhancer in B-cells inhibitor, zeta (NFKBIZ), transcript variant 1, mRNA [NM_031419] |
| A_21_P0002645 | 0.01978935 | 4.2438154 | MIR7515HG | Homo sapiens MIR7515 host gene (non-protein coding) (MIR7515HG), long non-coding RNA [NR_110497] |
| A_22_P00022282 | 0.017040258 | 2.260151 |  | AY563251 MHC class II antigen beta chain {Aotus nigriceps} (exp=-1; wgp=0; cg=0), partial (18%) [THC2694256] |
| A_22_P00004369 | 3.77E-04 | 3.973603 | lnc-COL4A3BP-1 | LNCipedia lincRNA (lnc-COL4A3BP-1), lincRNA [lnc-COL4A3BP-1:1] |
| A_22_P00000943 | 0.001105211 | 2.6864667 |  | Q4RGK4_TETNG (Q4RGK4) Chromosome undetermined SCAF15099, whole genome shotgun sequence, partial (6%) [THC2710827] |
| A_21_P0012039 | 0.003538653 | 3.7355487 |  | aldehyde oxidase 3, pseudogene [Source:HGNC Symbol;Acc:HGNC:19049] [ENST00000467633] |
| A_21_P0013274 | 0.005237111 | 7.956217 |  | T cell receptor beta variable 1 (pseudogene) [Source:HGNC Symbol;Acc:HGNC:12176] [ENST00000476502] |
| A_32_P174908 | 0.014490932 | -3.1232588 | WAPAL | Homo sapiens wings apart-like homolog (Drosophila) (WAPAL), mRNA [NM_015045] |
| A_21_P0002157 | 0.002772977 | 3.3242884 | lnc-SNTG2-3 | LNCipedia lincRNA (lnc-SNTG2-3), lincRNA [lnc-SNTG2-3:3] |
| A_21_P0009012 | 0.01038959 | 3.4462364 | lnc-PARN-6 | LNCipedia lincRNA (lnc-PARN-6), lincRNA [lnc-PARN-6:3] |
| A_21_P0010335 | 6.91E-05 | 5.559329 | LOC100506271 | Homo sapiens uncharacterized LOC100506271 (LOC100506271), long non-coding RNA [NR_110515] |
| A_21_P0004486 | 4.30E-04 | 3.216847 | lnc-ADCY2-5 | LNCipedia lincRNA (lnc-ADCY2-5), lincRNA [lnc-ADCY2-5:1] |
| A_22_P00023598 | 0.017040258 | 2.881716 | lnc-HSPA4-2 | Homo sapiens cDNA FLJ46297 fis, clone TESTI4035872. [AK128174] |
| A_21_P0004644 | 0.004264157 | 2.2710183 | lnc-SLC39A7-2 | LNCipedia lincRNA (lnc-SLC39A7-2), lincRNA [lnc-SLC39A7-2:1] |
| A_33_P3277808 | 0.017647024 | 2.194501 | LOC101928125 | PREDICTED: Homo sapiens major seminal plasma glycoprotein PSP-I-like (LOC101928125), mRNA [XM_005270310] |
| A_23_P110879 | 0.00340917 | -2.3020911 | TRAF3IP2 | Homo sapiens TRAF3 interacting protein 2 (TRAF3IP2), transcript variant 2, mRNA [NM_147686] |
| A_21_P0003235 | 2.66E-04 | 7.687146 | lnc-GPR27-1 | LNCipedia lincRNA (lnc-GPR27-1), lincRNA [lnc-GPR27-1:3] |
| A_22_P00022858 | 0.008900492 | 3.9459813 |  | DA957234 SPLEN2 Homo sapiens cDNA clone SPLEN2036472 5', mRNA sequence [DA957234] |
| A_22_P00010139 | 0.010704964 | 2.3321137 | D21S2088E | Homo sapiens D21S2088E (D21S2088E), long non-coding RNA [NR_040254] |
| A_23_P80295 | 0.016460778 | 3.1567261 | SYN3 | Homo sapiens synapsin III (SYN3), transcript variant IIIa, mRNA [NM_003490] |
| A_22_P00007799 | 0.008065707 | 3.8937006 | lnc-HOOK1-1 | LNCipedia lincRNA (lnc-HOOK1-1), lincRNA [lnc-HOOK1-1:1] |
| A_23_P55400 | 4.27E-04 | 4.089857 | KRT36 | Homo sapiens keratin 36, type I (KRT36), mRNA [NM_003771] |
| A_23_P64243 | 0.006797854 | 3.046735 | UPK2 | Homo sapiens uroplakin 2 (UPK2), mRNA [NM_006760] |
| A_21_P0005797 | 0.005392477 | 4.1703 | lnc-PPP1R3B-2 | LNCipedia lincRNA (lnc-PPP1R3B-2), lincRNA [lnc-PPP1R3B-2:2] |
| A_21_P0012420 | 0.010246585 | 4.5003047 | LOC100507391 |  |
| A_21_P0006921 | 0.003906062 | 3.2103932 | lnc-EMX2-2 | LNCipedia lincRNA (lnc-EMX2-2), lincRNA [lnc-EMX2-2:4] |
| A_33_P3220352 | 0.010367082 | 4.0453553 | CNPY1 | Homo sapiens canopy FGF signaling regulator 1 (CNPY1), mRNA [NM_001103176] |
| A_21_P0009952 | 0.002192226 | 3.8232558 | lnc-SYNDIG1-4 | LNCipedia lincRNA (lnc-SYNDIG1-4), lincRNA [lnc-SYNDIG1-4:1] |
| A_23_P102258 | 0.009996453 | -2.4556124 | MRPL53 | Homo sapiens mitochondrial ribosomal protein L53 (MRPL53), mRNA [NM_053050] |
| A_21_P0006682 | 0.001645564 | 2.7888157 | lnc-C10orf35-2 | LNCipedia lincRNA (lnc-C10orf35-2), lincRNA [lnc-C10orf35-2:1] |
| A_21_P0007654 | 0.019688005 | 2.9770412 | lnc-TMEM132B-2 | LNCipedia lincRNA (lnc-TMEM132B-2), lincRNA [lnc-TMEM132B-2:3] |
| A_21_P0002470 | 9.86E-04 | 7.711714 | lnc-POLR1A-1 | LNCipedia lincRNA (lnc-POLR1A-1), lincRNA [lnc-POLR1A-1:1] |
| A_21_P0002515 | 0.017598884 | 2.7426815 | lnc-RBMS1-1 | LNCipedia lincRNA (lnc-RBMS1-1), lincRNA [lnc-RBMS1-1:1] |
| A_19_P00322662 | 0.013590261 | 2.0565712 | EWSAT1 | Homo sapiens Ewing sarcoma associated transcript 1 (EWSAT1), long non-coding RNA [NR_026949] |
| A_33_P3214096 | 0.016755583 | 2.234839 | ATF3 | Homo sapiens activating transcription factor 3 (ATF3), transcript variant 4, mRNA [NM_001040619] |
| A_22_P00009179 | 0.018723087 | 2.2294493 | lnc-LOX-1 | Q5T9M3_HUMAN (Q5T9M3) Protein tyrosine phosphatase, receptor type, C, partial (54%) [THC2612931] |
| A_33_P3240078 | 0.007549429 | 2.8287814 |  |  |
| A_21_P0009463 | 0.002044132 | 4.021201 | lnc-MIB1-1 | LNCipedia lincRNA (lnc-MIB1-1), lincRNA [lnc-MIB1-1:1] |
| A_22_P00020637 | 0.004578975 | 2.9160318 |  |  |
| A_24_P68783 | 0.018414054 | 2.8707626 | IL36RN | Homo sapiens interleukin 36 receptor antagonist (IL36RN), transcript variant 1, mRNA [NM_012275] |
| A_22_P00018413 | 0.019137397 | 2.7362313 | LINC00858 | Homo sapiens long intergenic non-protein coding RNA 858 (LINC00858), long non-coding RNA [NR_038220] |
| A_33_P3304948 | 0.012613835 | 5.044515 | XKR7 | Homo sapiens XK, Kell blood group complex subunit-related family, member 7 (XKR7), mRNA [NM_001011718] |
| A_33_P3212764 | 0.008503397 | 9.038464 | FLJ40039 | Homo sapiens cDNA FLJ40039 fis, clone SYNOV2000397. [AK097358] |
| A_22_P00010556 | 0.003175485 | 4.6056247 | NCOA6 | nuclear receptor coactivator 6 [Source:HGNC Symbol;Acc:HGNC:15936] [ENST00000593786] |
| A_21_P0002546 | 0.012042754 | 2.4894626 | LOC101928662 | PREDICTED: Homo sapiens uncharacterized LOC101928662 (LOC101928662), transcript variant X1, ncRNA [XR_241421] |
| A_33_P3336627 | 8.61E-04 | 3.8727329 | OR10G9 | Homo sapiens olfactory receptor, family 10, subfamily G, member 9 (OR10G9), mRNA [NM_001001953] |
| A_33_P3313323 | 0.008503397 | 2.28357 |  |  |
| A_21_P0002692 | 0.01492285 | 2.3248522 | lnc-EPHA4-2 | LNCipedia lincRNA (lnc-EPHA4-2), lincRNA [lnc-EPHA4-2:1] |
| A_33_P3653271 | 0.004081493 | 2.2114835 | LINC01015 | Homo sapiens long intergenic non-protein coding RNA 1015 (LINC01015), transcript variant 1, long non-coding RNA [NR_037179] |
| A_33_P3381777 | 9.14E-04 | 6.1279593 | TREML1 | Homo sapiens triggering receptor expressed on myeloid cells-like 1 (TREML1), transcript variant 1, mRNA [NM_178174] |
| A_24_P270728 | 0.006640496 | -2.5260444 | NUPR1 | Homo sapiens nuclear protein, transcriptional regulator, 1 (NUPR1), transcript variant 1, mRNA [NM_001042483] |
| A_21_P0002872 | 0.001058173 | 3.871664 |  |  |
| A_22_P00025102 | 0.016322235 | 2.4417377 |  |  |
| A_19_P00319528 | 0.005823871 | 3.5141027 | LOC100507420 | Homo sapiens uncharacterized LOC100507420 (LOC100507420), transcript variant 1, long non-coding RNA [NR_121620] |
| A_33_P3253006 | 8.63E-04 | 2.462161 | CRYAA | H.sapiens alpha-A crystallin gene exon 1,2 and pseudoexon. [X14789] |
| A_33_P3284252 | 0.014822709 | -3.803619 |  |  |
| A_24_P124370 | 0.011191177 | -2.9699318 | PARVA | Homo sapiens parvin, alpha (PARVA), mRNA [NM_018222] |
| A_24_P361427 | 0.002315945 | 2.9704056 | TBC1D29 | Homo sapiens TBC1 domain family, member 29 (TBC1D29), mRNA [NM_015594] |
| A_22_P00009070 | 0.012797289 | 2.4591782 |  |  |
| A_23_P63158 | 0.008645061 | 3.7896347 | SPTA1 | Homo sapiens spectrin, alpha, erythrocytic 1 (SPTA1), mRNA [NM_003126] |
| A_23_P109072 | 0.01693474 | 2.553925 | SALL4 | Homo sapiens spalt-like transcription factor 4 (SALL4), mRNA [NM_020436] |
| A_22_P00007108 | 0.012684073 | 4.744449 | lnc-GLT1D1-3 | LNCipedia lincRNA (lnc-GLT1D1-3), lincRNA [lnc-GLT1D1-3:1] |
| A_23_P5258 | 0.006831878 | 6.785956 | OR10H4 | Homo sapiens olfactory receptor, family 10, subfamily H, member 4 (OR10H4), mRNA [NM_001004465] |
| A_24_P223874 | 0.005741094 | 2.6603 | SFTPA1 | Homo sapiens surfactant protein A1 (SFTPA1), transcript variant 1, mRNA [NM_005411] |
| A_21_P0011703 | 0.003624071 | 4.784047 | XLOC_l2_006789 | BROAD Institute lincRNA (XLOC_l2_006789), lincRNA [TCONS_l2_00012636] |
| A_22_P00004472 | 0.007157139 | 2.3852625 |  | Homo sapiens cDNA clone IMAGE:5260625. [BC032898] |
| A_23_P75630 | 0.011419726 | 3.9685032 | APOA5 | Homo sapiens apolipoprotein A-V (APOA5), transcript variant 1, mRNA [NM_052968] |
| A_23_P425880 | 0.003686147 | -2.8325024 | TRIO | Homo sapiens trio Rho guanine nucleotide exchange factor (TRIO), mRNA [NM_007118] |
| A_23_P127684 | 0.011475068 | 3.8708153 | OR5F1 | Homo sapiens olfactory receptor, family 5, subfamily F, member 1 (OR5F1), mRNA [NM_003697] |
| A_21_P0006248 | 0.01693474 | 2.8308868 | lnc-C9orf66-5 | LNCipedia lincRNA (lnc-C9orf66-5), lincRNA [lnc-C9orf66-5:1] |
| A_33_P3286873 | 0.008645061 | 2.0215678 | CD247 | CD247 molecule [Source:HGNC Symbol;Acc:HGNC:1677] [ENST00000483825] |
| A_23_P255672 | 0.014642934 | -2.4647493 | ABLIM2 | Homo sapiens actin binding LIM protein family, member 2 (ABLIM2), transcript variant 6, mRNA [NM_032432] |
| A_21_P0011583 | 1.37E-04 | 9.924531 | XLOC_l2_006036 | BROAD Institute lincRNA (XLOC_l2_006036), lincRNA [TCONS_l2_00011163] |
| A_22_P00015633 | 0.005069702 | 4.925625 | lnc-SUMO1-1 | RST15833 Athersys RAGE Library Homo sapiens cDNA, mRNA sequence [BG196607] |
| A_22_P00004792 | 0.016828412 | 3.1696312 |  |  |
| A_21_P0002460 | 0.001428738 | 2.4934242 | lnc-RAB11FIP5-1 | LNCipedia lincRNA (lnc-RAB11FIP5-1), lincRNA [lnc-RAB11FIP5-1:1] |
| A_23_P89981 | 0.003071663 | 2.8343549 | CYP2F1 | Homo sapiens cytochrome P450, family 2, subfamily F, polypeptide 1 (CYP2F1), mRNA [NM_000774] |
| A_22_P00020273 | 0.012846116 | 3.6521745 | lnc-B4GALNT3-2 | LNCipedia lincRNA (lnc-B4GALNT3-2), lincRNA [lnc-B4GALNT3-2:2] |
| A_33_P3342300 | 0.004784878 | 3.562091 | VSX1 | Homo sapiens visual system homeobox 1 (VSX1), transcript variant 4, mRNA [NM_001256272] |
| A_22_P00018230 | 0.016783917 | 2.9878998 | lnc-ZNF680-2 | LNCipedia lincRNA (lnc-ZNF680-2), lincRNA [lnc-ZNF680-2:1] |
| A_21_P0005193 | 0.00809771 | 2.817017 | lnc-PHTF2-1 | Homo sapiens cDNA FLJ42316 fis, clone TRACH2019673. [AK124308] |
| A_33_P3264569 | 0.011367305 | 2.7675257 |  | T cell receptor beta variable 10-2 [Source:HGNC Symbol;Acc:HGNC:12178] [ENST00000426318] |
| A_21_P0003425 | 0.004571873 | 5.2792296 |  | DA175095 BRAMY2 Homo sapiens cDNA clone BRAMY2038379 5', mRNA sequence [DA175095] |
| A_21_P0012130 | 0.004663298 | 2.2538555 | XLOC_l2_008692 | BROAD Institute lincRNA (XLOC_l2_008692), lincRNA [TCONS_l2_00016363] |
| A_24_P366457 | 0.016248012 | -2.7026079 |  |  |
| A_22_P00019353 | 0.001876189 | 3.2885962 | LINC01043 | long intergenic non-protein coding RNA 1043 [Source:HGNC Symbol;Acc:HGNC:49031] [ENST00000565936] |
| A_21_P0009186 | 0.006057093 | 2.582744 |  |  |
| A_22_P00012892 | 0.008866115 | 2.693961 | lnc-RBM22-2 | 601113406F1 NIH_MGC_16 Homo sapiens cDNA clone IMAGE:3354000 5', mRNA sequence [BE252560] |
| A_21_P0005525 | 6.49E-04 | 3.0098622 | lnc-TSPAN12-2 | LNCipedia lincRNA (lnc-TSPAN12-2), lincRNA [lnc-TSPAN12-2:1] |
| A_33_P3227631 | 8.11E-04 | -2.4688153 | IQCH | Homo sapiens IQ motif containing H (IQCH), transcript variant 1, mRNA [NM_001031715] |
| A_33_P3226212 | 0.019903338 | -2.0257165 | JAM2 | Homo sapiens junctional adhesion molecule 2 (JAM2), transcript variant 3, mRNA [NM_001270408] |
| A_21_P0012838 | 0.014471924 | 3.6647997 | XLOC_l2_011692 | BROAD Institute lincRNA (XLOC_l2_011692), lincRNA [TCONS_l2_00022449] |
| A_21_P0008714 | 0.013622328 | 3.0285597 | lnc-GABRB3-2 | LNCipedia lincRNA (lnc-GABRB3-2), lincRNA [lnc-GABRB3-2:1] |
| ERCC-00157_60 | 0.014681964 | 2.4059165 |  |  |
| A_22_P00009343 | 0.004791047 | 7.0308414 |  | Homo sapiens cDNA FLJ33023 fis, clone THYMU1000527. [AK057585] |
| A_22_P00004672 | 0.003248681 | 3.7858582 |  | Homo sapiens cDNA clone IMAGE:5273698. [BC047414] |
| A_21_P0006291 | 5.41E-04 | 2.81664 | LINC01501 | Homo sapiens long intergenic non-protein coding RNA 1501 (LINC01501), long non-coding RNA [NR_034157] |
| A_21_P0002169 | 0.007240376 | 2.9851499 | lnc-HPCAL1-1 | LNCipedia lincRNA (lnc-HPCAL1-1), lincRNA [lnc-HPCAL1-1:13] |
| A_21_P0007383 | 0.017060386 | 3.047791 | lnc-SESN3-1 | LNCipedia lincRNA (lnc-SESN3-1), lincRNA [lnc-SESN3-1:2] |
| A_22_P00006225 | 5.60E-04 | 3.471464 | lnc-FAM50B-1 | LNCipedia lincRNA (lnc-FAM50B-1), lincRNA [lnc-FAM50B-1:1] |
| A_33_P3279381 | 4.68E-05 | 3.5968611 |  |  |
| A_21_P0007334 | 0.016781604 | 4.2457542 |  | doublecortin domain containing 1 [Source:HGNC Symbol;Acc:HGNC:20625] [ENST00000444572] |
| A_24_P85258 | 0.001854862 | 4.3000135 | CFAP74 | cilia and flagella associated protein 74 [Source:HGNC Symbol;Acc:HGNC:29368] [ENST00000270720] |
| A_23_P376124 | 0.004419687 | 5.992923 | RNASE8 | Homo sapiens ribonuclease, RNase A family, 8 (RNASE8), mRNA [NM_138331] |
| A_21_P0000594 | 9.45E-04 | 4.3485084 | BOK-AS1 | Homo sapiens BOK antisense RNA 1 (BOK-AS1), antisense RNA [NR_033346] |
| A_33_P3672756 | 0.004523509 | 4.3960094 | LOC284561 | PREDICTED: Homo sapiens uncharacterized LOC284561 (LOC284561), misc_RNA [XR_110828] |
| A_21_P0002489 | 0.007733543 | 2.8314905 | lnc-EDAR-1 | LNCipedia lincRNA (lnc-EDAR-1), lincRNA [lnc-EDAR-1:1] |
| A_21_P0008020 | 0.009551783 | 2.111132 |  | long intergenic non-protein coding RNA 404 [Source:HGNC Symbol;Acc:HGNC:42734] [ENST00000418660] |
| A_22_P00008881 | 0.003071663 | 4.12382 |  |  |
| A_23_P117782 | 0.00540034 | -2.150818 | LARP6 | Homo sapiens La ribonucleoprotein domain family, member 6 (LARP6), transcript variant 1, mRNA [NM_018357] |
| A_33_P3395651 | 0.012455245 | -2.3239985 | PCDHGA5 | Homo sapiens protocadherin gamma subfamily A, 5 (PCDHGA5), transcript variant 2, mRNA [NM_032054] |
| A_33_P3254900 | 0.019903338 | 2.4111836 |  | capicua transcriptional repressor pseudogene 26 [Source:HGNC Symbol;Acc:HGNC:48834] [ENST00000425003] |
| A_22_P00008657 | 0.018472334 | 2.133542 | lnc-KIAA0196-3 | LNCipedia lincRNA (lnc-KIAA0196-3), lincRNA [lnc-KIAA0196-3:1] |
| A_21_P0006116 | 7.61E-05 | 2.7800963 |  | 602490627F1 NIH_MGC_18 Homo sapiens cDNA clone IMAGE:4622599 5', mRNA sequence [BG437415] |
| A_21_P0008796 | 0.016422339 | 2.3708563 | lnc-RP11-82I10.1.1-2 | LNCipedia lincRNA (lnc-RP11-82I10.1.1-2), lincRNA [lnc-RP11-82I10.1.1-2:1] |
| A_23_P31399 | 0.014677444 | -2.32388 | PON2 | Homo sapiens paraoxonase 2 (PON2), transcript variant 1, mRNA [NM_000305] |
| A_33_P3243486 | 0.006242589 | 2.2788029 | IGFL4 | Homo sapiens IGF-like family member 4 (IGFL4), mRNA [NM_001002923] |
| A_22_P00012375 | 0.016735058 | 2.3102992 | lnc-PRICKLE2-3 | LNCipedia lincRNA (lnc-PRICKLE2-3), lincRNA [lnc-PRICKLE2-3:1] |
| A_22_P00010929 | 0.012954351 | 4.8499026 | OSTM1-AS1 | OSTM1 antisense RNA 1 [Source:HGNC Symbol;Acc:HGNC:43666] [ENST00000426008] |
| A_22_P00020536 | 0.005637546 | 4.897322 |  | Smith-Magenis syndrome chromosome region, candidate 2 (non-protein coding) [Source:HGNC Symbol;Acc:HGNC:17914] [ENST00000456090] |
| A_21_P0010458 | 0.005949104 | 2.4709833 | lnc-C22orf34-1 | LNCipedia lincRNA (lnc-C22orf34-1), lincRNA [lnc-C22orf34-1:1] |
| A_23_P107744 | 0.010394448 | 5.8171635 | S1PR5 | Homo sapiens sphingosine-1-phosphate receptor 5 (S1PR5), transcript variant 1, mRNA [NM_030760] |
| A_33_P3270262 | 0.009075529 | 2.936815 |  | cyclin Y-like 3 [Source:HGNC Symbol;Acc:HGNC:33206] [ENST00000407030] |
| A_23_P50946 | 0.003703089 | -2.5205235 | RAMP1 | Homo sapiens receptor (G protein-coupled) activity modifying protein 1 (RAMP1), mRNA [NM_005855] |
| A_33_P3324860 | 0.002822993 | 2.100728 | ETV3 | Homo sapiens ets variant 3 (ETV3), transcript variant 1, mRNA [NM_001145312] |
| A_22_P00022160 | 0.012797289 | 2.0637667 | lnc-TXLNB-3 | AF422245 muscle-derived protein MDP77 variant 2 {Mus musculus} (exp=-1; wgp=0; cg=0), partial (5%) [THC2666479] |
| A_22_P00023676 | 0.007596517 | 2.2914097 | LOC100507283 | Homo sapiens uncharacterized LOC100507283 (LOC100507283), long non-coding RNA [NR_103862] |
| A_21_P0005531 | 2.92E-04 | 2.8498824 | lnc-AKR1B1-1 | LNCipedia lincRNA (lnc-AKR1B1-1), lincRNA [lnc-AKR1B1-1:1] |
| A_22_P00025885 | 0.006701194 | 3.37776 |  |  |
| A_23_P211572 | 0.001457917 | 3.5138998 | SEPT3 | Homo sapiens septin 3 (SEPT3), transcript variant A, mRNA [NM_145733] |
| A_22_P00005407 | 8.55E-04 | 5.3033123 |  |  |
| A_22_P00025124 | 0.017209308 | 2.4136708 | lnc-CPPED1-5 | LNCipedia lincRNA (lnc-CPPED1-5), lincRNA [lnc-CPPED1-5:1] |
| A_23_P161331 | 0.002075288 | 3.7162611 | PCDH15 | Homo sapiens protocadherin-related 15 (PCDH15), transcript variant C, mRNA [NM_033056] |
| A_21_P0003139 | 0.002791597 | 3.003235 | lnc-RPL24-2 | LNCipedia lincRNA (lnc-RPL24-2), lincRNA [lnc-RPL24-2:1] |
| A_33_P3394554 | 0.010105339 | 3.8229983 | LOC101929983 | Homo sapiens PRAME family member 9/15-like (LOC101929983), mRNA [NM_001300891] |
| A_23_P28466 | 0.013712222 | 2.227862 | DAW1 | Homo sapiens dynein assembly factor with WDR repeat domains 1 (DAW1), mRNA [NM_178821] |
| A_21_P0013867 | 7.96E-04 | 2.6628928 |  |  |
| A_21_P0010213 | 0.001574271 | 2.2042325 | LINC00313 | long intergenic non-protein coding RNA 313 [Source:EntrezGene;Acc:114038] [ENST00000623549] |
| A_21_P0001312 | 0.014683946 | 3.5981326 | lnc-HOOK1-3 | LNCipedia lincRNA (lnc-HOOK1-3), lincRNA [lnc-HOOK1-3:1] |
| A_33_P3277575 | 0.014343364 | 2.0105438 | CDCA5 | cell division cycle associated 5 [Source:HGNC Symbol;Acc:HGNC:14626] [ENST00000404147] |
| A_22_P00014706 | 0.003703089 | 2.1456964 | RUNDC3A-AS1 | Homo sapiens RUNDC3A antisense RNA 1 (RUNDC3A-AS1), long non-coding RNA [NR_110802] |
| A_33_P3295932 | 0.001645564 | 2.5759544 |  |  |
| A_33_P3268035 | 0.014342867 | 3.9371915 | LYZL1 | Homo sapiens lysozyme-like 1 (LYZL1), mRNA [NM_032517] |
| A_33_P7699408 | 0.015121243 | 2.5513365 | LOC101928973 | Homo sapiens uncharacterized LOC101928973 (LOC101928973), long non-coding RNA [NR_125966] |
| A_21_P0005413 | 0.005147894 | 2.3876498 | LOC340340 | PREDICTED: Homo sapiens uncharacterized LOC340340 (LOC340340), misc_RNA [XR_252283] |
| A_21_P0009717 | 0.002366805 | 2.7004628 | lnc-LSR-1 | LNCipedia lincRNA (lnc-LSR-1), lincRNA [lnc-LSR-1:4] |
| A_21_P0004151 | 3.77E-04 | 7.0691047 |  | Q3X7A0_METFL (Q3X7A0) Transport system permease protein precursor, partial (6%) [THC2690553] |
| A_24_P110983 | 0.006177985 | -2.2308145 | AKT3 | Homo sapiens v-akt murine thymoma viral oncogene homolog 3 (AKT3), transcript variant 1, mRNA [NM_005465] |
| A_21_P0008384 | 0.013079802 | 2.7326946 | lnc-C14orf177-4 | LNCipedia lincRNA (lnc-C14orf177-4), lincRNA [lnc-C14orf177-4:1] |
| A_33_P3318227 | 0.002843195 | 5.6262546 |  |  |
| A_33_P3348127 | 0.011555376 | 2.2766414 | GRIN1 | Homo sapiens glutamate receptor, ionotropic, N-methyl D-aspartate 1 (GRIN1), transcript variant GluN1-5b, mRNA [NM_001185091] |
| A_23_P147397 | 0.003240238 | -2.2093556 | DYNC2H1 | Homo sapiens dynein, cytoplasmic 2, heavy chain 1 (DYNC2H1), transcript variant 2, mRNA [NM_001080463] |
| A_24_P32139 | 0.013324657 | 3.0856287 | HTR3E | Homo sapiens 5-hydroxytryptamine (serotonin) receptor 3E, ionotropic (HTR3E), transcript variant 1, mRNA [NM_182589] |
| A_23_P102202 | 0.007796347 | -2.0322642 | MSH6 | Homo sapiens mutS homolog 6 (MSH6), transcript variant 1, mRNA [NM_000179] |
| A_33_P3224050 | 0.01693474 | 3.5674214 |  |  |
| A_22_P00024439 | 0.001854862 | 7.1527967 | lnc-AC021066.1-1 | LNCipedia lincRNA (lnc-AC021066.1-1), lincRNA [lnc-AC021066.1-1:1] |
| A_21_P0004369 | 0.006341876 | 3.205454 | lnc-CDH9-2 | LNCipedia lincRNA (lnc-CDH9-2), lincRNA [lnc-CDH9-2:2] |
| A_33_P3777165 | 0.002315945 | 2.8112948 | FLJ31715 | Homo sapiens cDNA clone IMAGE:3831619. [BC022164] |
| A_21_P0010819 | 0.013260091 | 2.8409956 |  | ATPase, aminophospholipid transporter, class I, type 8A, member 2 pseudogene 1 [Source:HGNC Symbol;Acc:HGNC:39662] [ENST00000420453] |
| A_19_P00315959 | 0.01171016 | 2.2550957 | lnc-NR2F1-1 | LNCipedia lincRNA (lnc-NR2F1-1), lincRNA [lnc-NR2F1-1:1] |
| A_21_P0005137 | 2.62E-04 | 2.8084896 | lnc-C6orf115-1 | LNCipedia lincRNA (lnc-C6orf115-1), lincRNA [lnc-C6orf115-1:1] |
| A_33_P3364776 | 0.001952262 | 3.1006858 |  | suppressor of Ty 5 homolog (S. cerevisiae) [Source:HGNC Symbol;Acc:HGNC:11469] [ENST00000396843] |
| A_22_P00016721 | 0.01375931 | 2.2341948 | lnc-TRAPPC6B-1 | LNCipedia lincRNA (lnc-TRAPPC6B-1), lincRNA [lnc-TRAPPC6B-1:1] |
| A_33_P3228427 | 0.016850244 | 3.038851 |  | BX103560 NCI_CGAP_GC4 Homo sapiens cDNA clone IMAGp998I073716, mRNA sequence [BX103560] |
| A_33_P3338220 | 0.010407414 | 3.9149265 | SH2D7 | Homo sapiens SH2 domain containing 7 (SH2D7), mRNA [NM_001101404] |
| A_33_P3232290 | 0.005823871 | 2.9430306 | NPS | Homo sapiens neuropeptide S (NPS), mRNA [NM_001030013] |
| A_32_P503113 | 0.003671488 | 3.2683585 | CCDC105 | Homo sapiens coiled-coil domain containing 105 (CCDC105), mRNA [NM_173482] |
| A_33_P3390411 | 0.007549429 | 2.6232696 | F9 | Homo sapiens coagulation factor IX (F9), mRNA [NM_000133] |
| A_22_P00010141 | 0.006022227 | 4.1745195 | lnc-MRPL39-2 | Homo sapiens neuroblastoma cDNA, clone:Nbla00208, full insert sequence. [AB075497] |
| A_22_P00006387 | 0.004883347 | 2.2970195 |  |  |
| A_33_P3261620 | 0.011834213 | 5.0391145 | GHSR | Homo sapiens growth hormone secretagogue receptor (GHSR), transcript variant 1b, mRNA [NM_004122] |
| A_22_P00016781 | 0.015756283 | 2.003045 |  |  |
| A_23_P86252 | 0.019243294 | -2.3786972 | PIGC | Homo sapiens phosphatidylinositol glycan anchor biosynthesis, class C (PIGC), transcript variant 2, mRNA [NM_002642] |
| A_22_P00003531 | 3.69E-04 | 5.8602424 | lnc-CCND2-1 | LNCipedia lincRNA (lnc-CCND2-1), lincRNA [lnc-CCND2-1:1] |
| A_21_P0001219 | 7.96E-04 | 3.9666686 |  | 601868421F1 NIH_MGC_17 Homo sapiens cDNA clone IMAGE:4111180 5', mRNA sequence [BF205649] |
| A_21_P0003253 | 0.006167846 | 2.421236 | lnc-GYG1-1 | LNCipedia lincRNA (lnc-GYG1-1), lincRNA [lnc-GYG1-1:1] |
| A_23_P83200 | 0.015569247 | 2.3606668 | AK8 | Homo sapiens adenylate kinase 8 (AK8), mRNA [NM_152572] |
| A_33_P3398316 | 0.004848211 | 2.10253 | MMP27 | Homo sapiens matrix metallopeptidase 27 (MMP27), mRNA [NM_022122] |
| A_33_P3278789 | 0.019216066 | 2.5900357 | KCNK16 | Homo sapiens potassium channel, two pore domain subfamily K, member 16 (KCNK16), transcript variant 3, mRNA [NM_001135106] |
| A_21_P0001659 | 0.017535977 | 3.252219 | lnc-ACTRT2-1 | LNCipedia lincRNA (lnc-ACTRT2-1), lincRNA [lnc-ACTRT2-1:2] |
| A_21_P0005959 | 0.007384243 | 2.7039812 | lnc-DEFB104B-1 | LNCipedia lincRNA (lnc-DEFB104B-1), lincRNA [lnc-DEFB104B-1:2] |
| A_22_P00017378 | 0.002130372 | 3.8717473 | lnc-UVRAG-1 | LNCipedia lincRNA (lnc-UVRAG-1), lincRNA [lnc-UVRAG-1:1] |
| A_21_P0005263 | 0.004061004 | 2.3013778 |  | 2109260A B cell growth factor. {Homo sapiens} (exp=-1; wgp=0; cg=0), partial (10%) [THC2771421] |
| A_21_P0013536 | 0.017797079 | 3.025001 | XLOC_l2_014182 | BROAD Institute lincRNA (XLOC_l2_014182), lincRNA [TCONS_l2_00028479] |
| A_21_P0008898 | 0.005508725 | 5.685357 |  |  |
| A_32_P23624 | 0.018027226 | 3.4853911 | ABCB10 | Homo sapiens ATP-binding cassette, sub-family B (MDR/TAP), member 10 (ABCB10), mRNA [NM_012089] |
| A_32_P231617 | 0.010950207 | -2.4582489 | TM4SF1 | Homo sapiens transmembrane 4 L six family member 1 (TM4SF1), mRNA [NM_014220] |
| A_23_P54006 | 0.00713022 | -2.0019395 | HECTD1 | Homo sapiens HECT domain containing E3 ubiquitin protein ligase 1 (HECTD1), mRNA [NM_015382] |
| A_22_P00015699 | 0.009564621 | -3.3841538 |  |  |
| A_22_P00009786 | 0.005927372 | 3.6918032 |  | long intergenic non-protein coding RNA 461 [Source:HGNC Symbol;Acc:HGNC:42810] [ENST00000506978] |
| A_22_P00011777 | 0.001457917 | 3.1190183 | lnc-PFKP-3 | LNCipedia lincRNA (lnc-PFKP-3), lincRNA [lnc-PFKP-3:1] |
| A_23_P200096 | 0.016071025 | -2.4249752 | SPSB1 | Homo sapiens splA/ryanodine receptor domain and SOCS box containing 1 (SPSB1), mRNA [NM_025106] |
| A_21_P0008867 | 0.004017944 | 3.2253337 | lnc-RGMA-9 | LNCipedia lincRNA (lnc-RGMA-9), lincRNA [lnc-RGMA-9:2] |
| A_33_P3336587 | 0.019243294 | 2.0112746 | LOC283710 | Homo sapiens uncharacterized LOC283710 (LOC283710), mRNA [NM_001243538] |
| A_21_P0007098 | 0.019543365 | 2.7996023 | lnc-SVIL-2 | LNCipedia lincRNA (lnc-SVIL-2), lincRNA [lnc-SVIL-2:1] |
| A_22_P00013499 | 0.015958356 | 2.857596 | lnc-RP11-362K2.2.1-2 | LNCipedia lincRNA (lnc-RP11-362K2.2.1-2), lincRNA [lnc-RP11-362K2.2.1-2:1] |
| A_24_P30567 | 0.001574271 | -2.1731164 | SPPL3 | Homo sapiens signal peptide peptidase like 3 (SPPL3), mRNA [NM_139015] |
| A_21_P0012094 | 0.005160756 | 2.7696087 |  |  |
| A_21_P0012378 | 0.001452869 | 3.6428072 | LINC00870 | Homo sapiens long intergenic non-protein coding RNA 870 (LINC00870), long non-coding RNA [NR_038221] |
| A_33_P3344991 | 0.003660631 | 2.3975987 | TBC1D3L | Homo sapiens cDNA FLJ16441 fis, clone BRAMY3003205, highly similar to TBC1 domain family member 3. [AK122833] |
| A_22_P00001799 | 0.005823871 | 2.6582808 |  | Homo sapiens cDNA FLJ41764 fis, clone IMR322005833. [AK123758] |
| A_33_P3382849 | 0.001301473 | 2.4986737 | ACR | Homo sapiens acrosin (ACR), mRNA [NM_001097] |
| A_23_P53476 | 0.016979635 | -3.6335676 | LDHB | Homo sapiens lactate dehydrogenase B (LDHB), transcript variant 1, mRNA [NM_002300] |
| A_24_P150931 | 0.003332298 | 2.2923515 | NXPH3 | Homo sapiens neurexophilin 3 (NXPH3), mRNA [NM_007225] |
| A_33_P3337959 | 0.001174549 | 2.189733 | LOC101927468 | Homo sapiens cDNA FLJ46360 fis, clone TESTI4049863. [AK128227] |
| A_22_P00011254 | 0.015893705 | 2.4511445 |  | Q5DYM4_VIBF1 (Q5DYM4) Sulfate transporter, partial (4%) [THC2714711] |
| A_24_P275199 | 0.018723087 | 3.4675741 | KCNC1 | Homo sapiens potassium channel, voltage gated Shaw related subfamily C, member 1 (KCNC1), transcript variant 1, mRNA [NM_001112741] |
| A_23_P253200 | 0.016775608 | -2.2717342 | RPL15 | Homo sapiens ribosomal protein L15 (RPL15), transcript variant 1, mRNA [NM_002948] |
| A_22_P00020319 | 7.41E-04 | 5.5241175 | lnc-TMEM159-1 | LNCipedia lincRNA (lnc-TMEM159-1), lincRNA [lnc-TMEM159-1:1] |
| A_23_P418562 | 0.006551965 | 3.8832855 | SLC6A11 | Homo sapiens solute carrier family 6 (neurotransmitter transporter), member 11 (SLC6A11), mRNA [NM_014229] |
| A_21_P0008346 | 0.014487425 | 2.5739815 | lnc-MNAT1-2 | LNCipedia lincRNA (lnc-MNAT1-2), lincRNA [lnc-MNAT1-2:1] |
| A_24_P344416 | 0.016420968 | 2.2831702 | DSC3 | Homo sapiens desmocollin 3 (DSC3), transcript variant Dsc3b, mRNA [NM_024423] |
| A_23_P319133 | 0.005927372 | -3.407498 | DNAJC10 | Homo sapiens DnaJ (Hsp40) homolog, subfamily C, member 10 (DNAJC10), transcript variant 1, mRNA [NM_018981] |
| A_22_P00007708 | 9.45E-04 | 10.030697 | lnc-HJURP-1 | LNCipedia lincRNA (lnc-HJURP-1), lincRNA [lnc-HJURP-1:1] |
| A_24_P125561 | 0.003570686 | 2.3292387 | SIGLEC9 | Homo sapiens sialic acid binding Ig-like lectin 9 (SIGLEC9), transcript variant 2, mRNA [NM_014441] |
| A_24_P298716 | 0.015872229 | 3.0043058 | FAM197Y2 | Homo sapiens family with sequence similarity 197, Y-linked, member 2, pseudogene (FAM197Y2), non-coding RNA [NR_001553] |
| A_22_P00003631 | 9.30E-04 | 5.4661283 |  |  |
| A_22_P00014692 | 0.01994502 | 2.9816573 | lnc-SLC25A28-1 | AGENCOURT_10554929 NIH_MGC_127 Homo sapiens cDNA clone IMAGE:6715122 5', mRNA sequence [BU942368] |
| A_33_P3210637 | 0.015645228 | 2.0754244 | OR4C16 | Homo sapiens olfactory receptor, family 4, subfamily C, member 16 (gene/pseudogene) (OR4C16), mRNA [NM_001004701] |
| A_21_P0010749 | 0.004234207 | -2.864532 | LOC100288069 | Homo sapiens uncharacterized LOC100288069 (LOC100288069), long non-coding RNA [NR_033908] |
| A_22_P00004129 | 0.013101991 | 2.98381 | lnc-CLDN20-2 | LNCipedia lincRNA (lnc-CLDN20-2), lincRNA [lnc-CLDN20-2:1] |
| A_21_P0001015 | 5.41E-04 | 3.9448278 |  |  |
| A_33_P3585924 | 0.001160449 | 3.5014973 | LOC339529 | Homo sapiens uncharacterized LOC339529 (LOC339529), long non-coding RNA [NR_033883] |
| A_21_P0012225 | 0.002843195 | 2.2769144 | LOC101927843 | Homo sapiens uncharacterized LOC101927843 (LOC101927843), long non-coding RNA [NR_109959] |
| A_23_P395632 | 0.015893705 | 2.018394 | R3HDML | Homo sapiens R3H domain containing-like (R3HDML), mRNA [NM_178491] |
| A_33_P3381206 | 0.001729655 | 4.511625 | IGLC1* (nome mod IPA) | immunoglobulin lambda variable 4-3 [Source:HGNC Symbol;Acc:HGNC:5919] [ENST00000390318] |
| A_32_P155666 | 0.007318523 | 3.0915344 | ECEL1 | Homo sapiens endothelin converting enzyme-like 1 (ECEL1), transcript variant 1, mRNA [NM_004826] |
| A_32_P394491 | 0.008900492 | 2.9183822 | LHFPL3-AS1 | Homo sapiens LHFPL3 antisense RNA 1 (LHFPL3-AS1), transcript variant 2, long non-coding RNA [NR_034141] |
| A_22_P00000953 | 0.007796347 | 3.9669008 | lnc-AKT1-2 | LNCipedia lincRNA (lnc-AKT1-2), lincRNA [lnc-AKT1-2:1] |
| A_22_P00015190 | 0.017540125 | 2.2668846 | LOC101929441 | Homo sapiens uncharacterized LOC101929441 (LOC101929441), long non-coding RNA [NR_123739] |
| A_22_P00024184 | 0.01693474 | 2.3370943 |  |  |
| A_23_P5968 | 0.005934981 | 3.672762 | SEMG2 | Homo sapiens semenogelin II (SEMG2), mRNA [NM_003008] |
| A_32_P104063 | 0.015121243 | -2.4845908 | CRNDE | Homo sapiens colorectal neoplasia differentially expressed (non-protein coding) (CRNDE), transcript variant 3, long non-coding RNA [NR_110453] |
| A_21_P0013180 | 0.00666965 | 2.56668 |  |  |
| A_33_P3228718 | 0.001116542 | 4.364396 |  | C-type lectin domain family 19, member A [Source:HGNC Symbol;Acc:HGNC:34522] [ENST00000476906] |
| A_21_P0008062 | 0.014352151 | 2.0843527 | lnc-LRCH1-1 | LNCipedia lincRNA (lnc-LRCH1-1), lincRNA [lnc-LRCH1-1:1] |
| A_23_P200138 | 0.012461365 | 2.493873 | SLAMF8 | Homo sapiens SLAM family member 8 (SLAMF8), mRNA [NM_020125] |
| A_24_P365025 | 0.018736346 | -2.7569175 | SPAG9 | Homo sapiens sperm associated antigen 9 (SPAG9), transcript variant 3, mRNA [NM_003971] |
| A_32_P123514 | 0.005637546 | -4.82375 | PABPC4L | Homo sapiens poly(A) binding protein, cytoplasmic 4-like (PABPC4L), mRNA [NM_001114734] |
| A_22_P00016493 | 0.01377768 | 2.4417007 | lnc-TMEM99-2 | HHAGE028280 Human liver regeneration after partial hepatectomy Homo sapiens cDNA, mRNA sequence [DW428550] |
| A_22_P00025468 | 0.018533755 | 2.0571473 | lnc-WDFY4-2 | LNCipedia lincRNA (lnc-WDFY4-2), lincRNA [lnc-WDFY4-2:1] |
| A_21_P0007786 | 0.004122045 | 3.1644087 | LINC00944 | Homo sapiens long intergenic non-protein coding RNA 944 (LINC00944), long non-coding RNA [NR_033878] |
| A_22_P00008385 | 0.002262092 | 5.3597436 | lnc-JMJD1C-1 | LNCipedia lincRNA (lnc-JMJD1C-1), lincRNA [lnc-JMJD1C-1:1] |
| A_22_P00003248 | 0.008519789 | 2.8122807 | LOC102723786 | PREDICTED: Homo sapiens uncharacterized LOC102723786 (LOC102723786), ncRNA [XR_429763] |
| A_32_P439662 | 0.009639516 | 3.1478233 | DNAH17 | Homo sapiens dynein, axonemal, heavy chain 17 (DNAH17), mRNA [NM_173628] |
| A_21_P0006196 | 0.018230628 | 2.6240568 | lnc-SMC2-1 | HY054275 RIKEN full-length enriched human cDNA library, testis Homo sapiens cDNA clone H04D192F14, mRNA sequence [HY054275] |
| A_22_P00014482 | 0.015623883 | -2.0487075 | lnc-SHCBP1-2 | Homo sapiens mRNA; cDNA DKFZp686O2462 (from clone DKFZp686O2462). [BX647213] |
| A_22_P00001806 | 0.003703089 | 8.528852 | lnc-ATP6V1B2-2 | Q16673_HUMAN (Q16673) PMS7 protein (HPMS7 protein) (Fragment), partial (16%) [THC2493945] |
| A_24_P296274 | 0.010217075 | 9.726872 | LINC00052 | Homo sapiens long intergenic non-protein coding RNA 52 (LINC00052), long non-coding RNA [NR_026869] |
| A_21_P0008023 | 0.014642934 | 2.7825015 | lnc-RASA3-1 | LNCipedia lincRNA (lnc-RASA3-1), lincRNA [lnc-RASA3-1:1] |
| A_22_P00007450 | 4.70E-04 | 3.0063276 | LNX1-AS1 | Homo sapiens LNX1 antisense RNA 1 (LNX1-AS1), long non-coding RNA [NR_046622] |
| A_33_P3366589 | 7.98E-04 | 8.8301 | OR56B4 | Homo sapiens olfactory receptor, family 56, subfamily B, member 4 (OR56B4), mRNA [NM_001005181] |
| A_22_P00011707 | 0.004017944 | 6.639546 | TLX1NB | TLX1 neighbor [Source:HGNC Symbol;Acc:HGNC:37183] [ENST00000425505] |
| A_22_P00022331 | 0.0094132 | 2.1190734 | lnc-FAM9C-2 | RST37950 Athersys RAGE Library Homo sapiens cDNA, mRNA sequence [BG218223] |
| A_21_P0010992 | 0.014078668 | 2.37587 | XLOC_l2_002433 | BROAD Institute lincRNA (XLOC_l2_002433), lincRNA [TCONS_l2_00004709] |
| A_33_P3233784 | 0.01214542 | 2.362453 | TMEM211 | Homo sapiens transmembrane protein 211 (TMEM211), mRNA [NM_001001663] |
| A_21_P0007121 | 0.006123647 | 2.283676 | lnc-EBF3-4 | LNCipedia lincRNA (lnc-EBF3-4), lincRNA [lnc-EBF3-4:2] |
| A_23_P7866 | 0.003703089 | 4.2034545 | GPR115 | Homo sapiens G protein-coupled receptor 115 (GPR115), mRNA [NM_153838] |
| A_33_P3266646 | 0.004017944 | 2.2525327 | ATP8B3 | Homo sapiens ATPase, aminophospholipid transporter, class I, type 8B, member 3 (ATP8B3), transcript variant 1, mRNA [NM_138813] |
| A_33_P3318681 | 0.013527386 | 2.3870504 | TMC3 | Homo sapiens transmembrane channel-like 3 (TMC3), mRNA [NM_001080532] |
| A_32_P69802 | 0.016285634 | 2.7217517 | SLCO1A2 | Homo sapiens solute carrier organic anion transporter family, member 1A2 (SLCO1A2), transcript variant 1, mRNA [NM_134431] |
| A_24_P787889 | 0.019137397 | 2.4266229 | TMEM191B | Homo sapiens transmembrane protein 191B (TMEM191B), mRNA [NM_001242313] |
| A_22_P00014627 | 0.017646626 | -2.3302572 | lnc-SLC16A3-5 | LNCipedia lincRNA (lnc-SLC16A3-5), lincRNA [lnc-SLC16A3-5:1] |
| A_22_P00013503 | 0.011372771 | 3.6150885 | LOC100507065 |  |
| A_22_P00005355 | 0.001702279 | 3.458719 |  |  |
| A_21_P0002565 | 0.011555376 | 2.746636 | lnc-TPO-4 | LNCipedia lincRNA (lnc-TPO-4), lincRNA [lnc-TPO-4:2] |
| A_23_P97736 | 0.016004086 | -2.6893437 | NCDN | Homo sapiens neurochondrin (NCDN), transcript variant 3, mRNA [NM_014284] |
| A_23_P54079 | 0.003686147 | -2.0292573 | OSGEP | Homo sapiens O-sialoglycoprotein endopeptidase (OSGEP), mRNA [NM_017807] |
| A_21_P0006221 | 0.007882567 | 2.1564674 | lnc-RP11-295D22.1.1-2 | LNCipedia lincRNA (lnc-RP11-295D22.1.1-2), lincRNA [lnc-RP11-295D22.1.1-2:1] |
| A_21_P0012402 | 0.014982661 | 2.164694 | LINC01210 | long intergenic non-protein coding RNA 1210 [Source:HGNC Symbol;Acc:HGNC:49642] [ENST00000469216] |
| A_22_P00000479 | 9.39E-04 | 4.2347946 | lnc-AC092327.1-2 | LNCipedia lincRNA (lnc-AC092327.1-2), lincRNA [lnc-AC092327.1-2:2] |
| A_23_P161983 | 4.99E-04 | 10.260766 | MRGPRX4 | Homo sapiens MAS-related GPR, member X4 (MRGPRX4), mRNA [NM_054032] |
| A_22_P00002672 | 0.007835183 | 3.3176463 | lnc-C1orf31-1 | LNCipedia lincRNA (lnc-C1orf31-1), lincRNA [lnc-C1orf31-1:1] |
| A_21_P0008509 | 0.017540125 | 3.4738648 | TTC6 | tetratricopeptide repeat domain 6 [Source:HGNC Symbol;Acc:HGNC:19739] [ENST00000533625] |
| A_33_P3398406 | 0.016176056 | 3.6311188 | GLP2R | Homo sapiens glucagon-like peptide 2 receptor (GLP2R), mRNA [NM_004246] |
| A_33_P3471466 | 0.018433057 | 2.746671 | LRRC74A | Homo sapiens leucine rich repeat containing 74A (LRRC74A), mRNA [NM_194287] |
| A_33_P3341479 | 0.010685129 | 2.1161132 | MID1IP1-AS1 | Homo sapiens MID1IP1 antisense RNA 1 (MID1IP1-AS1), long non-coding RNA [NR_046706] |
| A_33_P3406110 | 0.017849948 | 2.2703178 |  |  |
| A_22_P00024294 | 0.010018871 | 2.2590833 | lnc-TGS1-1 | LNCipedia lincRNA (lnc-TGS1-1), lincRNA [lnc-TGS1-1:1] |
| A_24_P293114 | 0.007276182 | 3.0601277 | SPTBN4 | Homo sapiens spectrin, beta, non-erythrocytic 4 (SPTBN4), transcript variant sigma1, mRNA [NM_020971] |
| A_21_P0010616 | 0.01865728 | 4.2541466 |  | solute carrier family 25 (mitochondrial carrier; phosphate carrier), member 3 pseudogene 1 [Source:HGNC Symbol;Acc:HGNC:26869] [ENST00000563752] |
| A_22_P00019261 | 0.008065707 | 2.5794141 |  |  |
| A_23_P337658 | 0.018722082 | 2.0175238 | ALPI | Homo sapiens alkaline phosphatase, intestinal (ALPI), mRNA [NM_001631] |
| A_21_P0009861 | 0.015958356 | 3.4881005 | LINC01440 | Homo sapiens long intergenic non-protein coding RNA 1440 (LINC01440), long non-coding RNA [NR_110629] |
| A_21_P0008268 | 8.11E-04 | 5.572903 | lnc-GAS6-4 | LNCipedia lincRNA (lnc-GAS6-4), lincRNA [lnc-GAS6-4:1] |
| A_22_P00004577 | 9.45E-04 | 6.166442 |  |  |
| A_22_P00020409 | 0.008065707 | 4.3342266 | PTCSC2 | PREDICTED: Homo sapiens uncharacterized LOC101928337 (RP11-23B15.1), transcript variant X1, ncRNA [XR_252621] |
| A_21_P0007507 | 0.017540125 | 4.111317 | lnc-C1QL4-3 | Homo sapiens cDNA FLJ40764 fis, clone TRACH2002954. [AK098083] |
| A_21_P0008782 | 0.012797289 | 2.3997688 | LINC00930 | Homo sapiens long intergenic non-protein coding RNA 930 (LINC00930), long non-coding RNA [NR_021493] |
| A_21_P0008529 | 0.015588387 | 3.703052 | lnc-STXBP6-1 | LNCipedia lincRNA (lnc-STXBP6-1), lincRNA [lnc-STXBP6-1:1] |
| A_23_P335981 | 0.009947869 | 2.6416848 | HOXC12 | Homo sapiens homeobox C12 (HOXC12), mRNA [NM_173860] |
| A_21_P0005936 | 0.0068485 | 2.152343 | lnc-UNC5D-1 | LNCipedia lincRNA (lnc-UNC5D-1), lincRNA [lnc-UNC5D-1:2] |
| A_21_P0002014 | 0.017119326 | 2.9826024 |  | AGENCOURT_7186941 NIH_MGC_100 Homo sapiens cDNA clone IMAGE:6002028 5', mRNA sequence [BU157057] |
| A_21_P0001389 | 0.018000286 | 2.3299003 | lnc-RALGPS2-1 | LNCipedia lincRNA (lnc-RALGPS2-1), lincRNA [lnc-RALGPS2-1:1] |
| A_22_P00011779 | 0.001606751 | 2.465113 |  | BX101064 Soares_pregnant_uterus_NbHPU Homo sapiens cDNA clone IMAGp998I121206, mRNA sequence [BX101064] |
| A_22_P00006749 | 0.01038959 | 3.3037484 |  |  |
| A_21_P0002467 | 0.01996856 | 2.8668513 | lnc-SUCLG1-2 | LNCipedia lincRNA (lnc-SUCLG1-2), lincRNA [lnc-SUCLG1-2:1] |
| A_21_P0009268 | 0.015756283 | 2.350096 | lnc-MAP2K6-2 | LNCipedia lincRNA (lnc-MAP2K6-2), lincRNA [lnc-MAP2K6-2:1] |
| A_22_P00009998 | 9.39E-04 | 3.6170008 | LINC01016 | long intergenic non-protein coding RNA 1016 [Source:HGNC Symbol;Acc:HGNC:48991] [ENST00000525912] |
| A_33_P3313125 | 0.001323787 | 5.517366 | lnc-FAM189A2-1 | LNCipedia lincRNA (lnc-FAM189A2-1), lincRNA [lnc-FAM189A2-1:1] |
| A_21_P0011398 | 0.009464912 | 2.0758357 | WHAMMP3 | WAS protein homolog associated with actin, golgi membranes and microtubules pseudogene 3 [Source:HGNC Symbol;Acc:HGNC:27892] [ENST00000622062] |
| A_33_P3289338 | 0.016064143 | 2.5852406 | LOC100131346 | Homo sapiens cDNA FLJ45704 fis, clone FEBRA2026977. [AK127606] |
| A_22_P00010112 | 0.018319929 | 3.1741014 | LOC494141 | Homo sapiens solute carrier family 25, member 51 pseudogene (LOC494141), transcript variant 1, non-coding RNA [NR_026541] |
| A_22_P00008763 | 4.19E-04 | 6.374983 | lnc-KIF13B-1 | LNCipedia lincRNA (lnc-KIF13B-1), lincRNA [lnc-KIF13B-1:1] |
| A_24_P283591 | 9.86E-04 | 3.7521281 | OR1Q1 | Homo sapiens olfactory receptor, family 1, subfamily Q, member 1 (OR1Q1), mRNA [NM_012364] |
| A_21_P0008266 | 0.019158948 | 2.2975898 | lnc-TUBGCP3-6 | LNCipedia lincRNA (lnc-TUBGCP3-6), lincRNA [lnc-TUBGCP3-6:3] |
| A_19_P00322316 | 0.002737081 | 6.7936287 | AOX2P | Homo sapiens aldehyde oxidase 2 pseudogene (AOX2P), non-coding RNA [NR_001557] |
| A_22_P00004634 | 0.011283191 | 4.634013 |  |  |
| A_22_P00012242 | 0.016431227 | 2.346659 | LOC101927729 | PREDICTED: Homo sapiens uncharacterized LOC101927729 (LOC101927729), ncRNA [XR_245767] |
| A_23_P415401 | 0.013372466 | -2.278923 | KLF9 | Homo sapiens Kruppel-like factor 9 (KLF9), mRNA [NM_001206] |
| A_33_P3671291 | 0.0192649 | -2.5089908 | SNORA12 | EST91069 Synovial sarcoma Homo sapiens cDNA 5' end, mRNA sequence [AA378382] |
| A_33_P3393311 | 0.002262092 | 3.285678 | MCHR1 | Homo sapiens melanin-concentrating hormone receptor 1 (MCHR1), mRNA [NM_005297] |
| A_21_P0009462 | 0.017535977 | 3.06158 | lnc-ANKRD30B-3 | LNCipedia lincRNA (lnc-ANKRD30B-3), lincRNA [lnc-ANKRD30B-3:1] |
| A_32_P200308 | 0.015542324 | 2.2788897 | LINC00977 | Homo sapiens long intergenic non-protein coding RNA 977 (LINC00977), long non-coding RNA [NR_033916] |
| A_22_P00001899 | 0.019259837 | 2.5553908 | ARHGAP31-AS1 | Homo sapiens ARHGAP31 antisense RNA 1 (ARHGAP31-AS1), long non-coding RNA [NR_046748] |
| A_21_P0007928 | 0.007681231 | 2.6388364 |  | long intergenic non-protein coding RNA 1079 [Source:HGNC Symbol;Acc:HGNC:49122] [ENST00000431171] |
| A_23_P302595 | 0.016071025 | 2.2650275 | LINC00518 | Homo sapiens long intergenic non-protein coding RNA 518 (LINC00518), long non-coding RNA [NR_027793] |
| A_21_P0007442 | 0.004417648 | 5.781006 | lnc-FOLR3-1 | LNCipedia lincRNA (lnc-FOLR3-1), lincRNA [lnc-FOLR3-1:2] |
| A_33_P3268234 | 7.57E-04 | 3.0647292 | KRT39 | Homo sapiens keratin 39, type I (KRT39), mRNA [NM_213656] |
| A_23_P436158 | 0.005823871 | 2.372491 | TLL2 | Homo sapiens tolloid-like 2 (TLL2), mRNA [NM_012465] |
| A_21_P0010377 | 0.004876962 | 2.9362066 | lnc-ZMAT5-1 | LNCipedia lincRNA (lnc-ZMAT5-1), lincRNA [lnc-ZMAT5-1:3] |
| A_22_P00002943 | 0.008821026 | 3.0341659 | lnc-C5orf42-3 | Q8TAW2_HUMAN (Q8TAW2) CD99L2 protein, partial (28%) [THC2622742] |
| A_24_P6825 | 0.019178513 | 2.7936351 | ZCCHC18 | Homo sapiens zinc finger, CCHC domain containing 18 (ZCCHC18), transcript variant 1, mRNA [NM_001143978] |
| A_24_P246841 | 0.008065707 | -3.634979 | SLC25A27 | Homo sapiens solute carrier family 25, member 27 (SLC25A27), transcript variant 1, mRNA [NM_004277] |
| A_22_P00013907 | 0.002772977 | 3.6939416 | LINC00595 | long intergenic non-protein coding RNA 595 [Source:HGNC Symbol;Acc:HGNC:31430] [ENST00000459633] |
| A_33_P3410121 | 0.011222256 | 3.9485433 | CFAP74 | cilia and flagella associated protein 74 [Source:HGNC Symbol;Acc:HGNC:29368] [ENST00000378592] |
| A_22_P00000970 | 0.010367082 | 3.6955295 | LOC102724152 | PREDICTED: Homo sapiens uncharacterized LOC102724152 (LOC102724152), ncRNA [XR_426111] |
| A_21_P0012489 | 9.73E-04 | 2.8127246 |  |  |
| A_23_P26994 | 0.001578439 | 2.956297 | GNGT2 | Homo sapiens guanine nucleotide binding protein (G protein), gamma transducing activity polypeptide 2 (GNGT2), transcript variant 1, mRNA [NM_031498] |
| A_23_P108751 | 0.018542387 | -3.5730658 | FHL2 | Homo sapiens four and a half LIM domains 2 (FHL2), transcript variant 5, mRNA [NM_001039492] |
| A_21_P0012122 | 0.009899479 | 3.3597152 | LINC01370 | Homo sapiens long intergenic non-protein coding RNA 1370 (LINC01370), transcript variant 1, long non-coding RNA [NR_109936] |
| A_23_P208302 | 0.014343364 | 2.4846325 | APOC2 | Homo sapiens apolipoprotein C-II (APOC2), mRNA [NM_000483] |
| A_21_P0007136 | 0.001699763 | 2.6902494 | LINC00900 | long intergenic non-protein coding RNA 900 [Source:HGNC Symbol;Acc:HGNC:27444] [ENST00000514294] |
| A_23_P123228 | 2.48E-04 | 5.931627 | SLC26A3 | Homo sapiens solute carrier family 26 (anion exchanger), member 3 (SLC26A3), mRNA [NM_000111] |
| A_22_P00010386 | 0.004280046 | -2.3974462 | MYL6B | Homo sapiens myosin, light chain 6B, alkali, smooth muscle and non-muscle (MYL6B), transcript variant 1, mRNA [NM_001199629] |
| A_22_P00019137 | 6.91E-05 | 4.4836903 | LINC00934 | Homo sapiens long intergenic non-protein coding RNA 934 (LINC00934), long non-coding RNA [NR_024246] |
| A_23_P129695 | 0.019117348 | -2.0148299 | VASN | Homo sapiens vasorin (VASN), mRNA [NM_138440] |
| A_21_P0009639 | 0.019903338 | 3.1098878 | lnc-ZNF516-3 | LNCipedia lincRNA (lnc-ZNF516-3), lincRNA [lnc-ZNF516-3:1] |
| A_33_P3340264 | 6.49E-04 | 6.3004947 |  |  |
| A_33_P3240763 | 0.009415058 | 2.8777778 |  | Homo sapiens cDNA clone IMAGE:5302821. [BC040838] |
| A_21_P0005938 | 0.0192649 | 3.0431056 | lnc-RP11-32K4.2.1-2 | LNCipedia lincRNA (lnc-RP11-32K4.2.1-2), lincRNA [lnc-RP11-32K4.2.1-2:2] |
| A_22_P00009785 | 1.78E-04 | 3.0925252 | LINC00461 | Homo sapiens long intergenic non-protein coding RNA 461 (LINC00461), transcript variant 3, long non-coding RNA [NR_015436] |
| A_22_P00015658 | 0.016420968 | 2.7624464 | SVOP | Homo sapiens SV2 related protein homolog (rat) (SVOP), mRNA [NM_018711] |
| A_21_P0002358 | 3.30E-04 | 2.5838423 |  |  |
| A_23_P139654 | 3.30E-04 | 3.0997014 | KLRC1 | Homo sapiens killer cell lectin-like receptor subfamily C, member 1 (KLRC1), transcript variant 2, mRNA [NM_007328] |
| A_21_P0009166 | 0.007796347 | 3.2586997 |  |  |
| A_33_P7587879 | 0.00384717 | 3.0551028 | LOC101927043 | Homo sapiens uncharacterized LOC101927043 (LOC101927043), transcript variant 1, long non-coding RNA [NR_110207] |
| A_23_P24031 | 0.004403577 | 2.1340046 | TLX1 | Homo sapiens T-cell leukemia homeobox 1 (TLX1), transcript variant 1, mRNA [NM_005521] |
| A_22_P00010659 | 0.014822709 | 3.6941276 | lnc-NEK6-1 | 603179887F1 NIH_MGC_121 Homo sapiens cDNA clone IMAGE:5244117 5', mRNA sequence [BI913141] |
| A_21_P0002265 | 0.006104963 | 3.1439226 | lnc-MRPS9-2 | LNCipedia lincRNA (lnc-MRPS9-2), lincRNA [lnc-MRPS9-2:2] |
| A_22_P00024840 | 0.001952262 | 2.48933 |  |  |
| A_22_P00003970 | 0.003383152 | 4.893385 |  |  |
| A_21_P0008280 | 0.004009948 | 3.3636184 | LOC102724190 | Homo sapiens uncharacterized LOC102724190 (LOC102724190), long non-coding RNA [NR_110553] |
| A_22_P00006897 | 0.015461167 | 2.4373374 | LINC00901 | Homo sapiens long intergenic non-protein coding RNA 901 (LINC00901), long non-coding RNA [NR_121607] |
| A_22_P00025855 | 0.007631484 | 3.3241842 | lnc-AMMECR1-1 | LNCipedia lincRNA (lnc-AMMECR1-1), lincRNA [lnc-AMMECR1-1:1] |
| A_22_P00006194 | 0.008900492 | 2.0088243 | lnc-FAM26E-1 | LNCipedia lincRNA (lnc-FAM26E-1), lincRNA [lnc-FAM26E-1:1] |
| A_33_P3272274 | 5.36E-04 | 4.0017734 | DEFB110 | Homo sapiens defensin, beta 110 locus (DEFB110), transcript variant 2, mRNA [NM_001037728] |
| A_22_P00006974 | 0.004403577 | 3.9464858 |  |  |
| A_21_P0005744 | 0.011555376 | 2.6131237 | lnc-GRHL2-5 | LNCipedia lincRNA (lnc-GRHL2-5), lincRNA [lnc-GRHL2-5:1] |
| A_22_P00008348 | 0.010898792 | 4.13796 | lnc-ITSN2-1 | DB072849 TESTI4 Homo sapiens cDNA clone TESTI4016056 5', mRNA sequence [DB072849] |
| A_33_P3237886 | 7.76E-04 | 7.446593 |  | Homo sapiens cDNA FLJ16652 fis, clone TESTI4036767. [AK131480] |
| A_33_P3220615 | 0.019216066 | 2.7306437 | LIN28B | Homo sapiens lin-28 homolog B (C. elegans) (LIN28B), mRNA [NM_001004317] |
| A_23_P164596 | 4.68E-05 | 16.52069 | SIGLEC12 | Homo sapiens sialic acid binding Ig-like lectin 12 (gene/pseudogene) (SIGLEC12), transcript variant 1, mRNA [NM_053003] |
| A_21_P0014831 | 3.27E-04 | 9.30498 |  | PREDICTED: Homo sapiens uncharacterized LOC100129860 (LOC100129860), misc_RNA [XR_133576] |
| A_22_P00002402 | 0.0192649 | 2.1011357 | lnc-C14orf126-2 | LNCipedia lincRNA (lnc-C14orf126-2), lincRNA [lnc-C14orf126-2:1] |
| A_32_P23308 | 0.019596959 | 2.98483 | LINC01366 | Homo sapiens long intergenic non-protein coding RNA 1366 (LINC01366), transcript variant 1, long non-coding RNA [NR_026945] |
| A_22_P00006689 | 0.003560867 | 4.311152 | lnc-FPR1-1 | Q6YFJ2_CICIN (Q6YFJ2) Resistance protein candidate (Fragment), partial (13%) [THC2681418] |
| A_22_P00025397 | 0.001133344 | 2.1017652 | lnc-OR1Q1-1 | 603073365F1 NIH_MGC_119 Homo sapiens cDNA clone IMAGE:5165105 5', mRNA sequence [BI830485] |
| A_21_P0010930 | 3.38E-04 | 3.3525863 | LINC00700 | long intergenic non-protein coding RNA 700 [Source:HGNC Symbol;Acc:HGNC:27422] [ENST00000438372] |
| A_22_P00011505 | 0.002801913 | 2.9678555 | LOC101929625 | Homo sapiens uncharacterized LOC101929625 (LOC101929625), long non-coding RNA [NR_109880] |
| A_22_P00013135 | 0.002130372 | 2.4415433 |  | ribonuclease, RNase A family, 11 (non-active) [Source:HGNC Symbol;Acc:HGNC:19269] [ENST00000413502] |
| A_21_P0010432 | 0.003203461 | 6.561139 | LOC101929416 | PREDICTED: Homo sapiens uncharacterized LOC101929416 (LOC101929416), transcript variant X2, ncRNA [XR_249026] |
| A_22_P00022078 | 4.68E-05 | 2.4219465 |  |  |
| A_22_P00017651 | 0.01657353 | 4.229083 | lnc-WRAP73-2 | LNCipedia lincRNA (lnc-WRAP73-2), lincRNA [lnc-WRAP73-2:1] |
| A_21_P0004538 | 0.002719358 | 3.4093049 |  |  |
| A_22_P00025471 | 0.004094415 | 2.413769 | LOC101928539 | Homo sapiens uncharacterized LOC101928539 (LOC101928539), long non-coding RNA [NR_109900] |
| A_32_P70203 | 4.05E-04 | 3.8586597 | FAM41C | Homo sapiens family with sequence similarity 41, member C (FAM41C), long non-coding RNA [NR_027055] |
| A_22_P00013463 | 0.004395038 | 3.9165804 |  | BX119121 Soares_NFL_T_GBC_S1 Homo sapiens cDNA clone IMAGp998H204000, mRNA sequence [BX119121] |
| A_22_P00014799 | 0.007423808 | 2.0355787 | LINC00673 | Homo sapiens long intergenic non-protein coding RNA 673 (LINC00673), long non-coding RNA [NR_036488] |
| A_21_P0000532 | 0.019269915 | 2.6008673 | MGC27382 | Homo sapiens uncharacterized MGC27382 (MGC27382), long non-coding RNA [NR_027310] |
| A_22_P00017663 | 5.98E-05 | 3.0847538 | lnc-WRN-7 | Homo sapiens, clone IMAGE:5166083, mRNA. [BC037250] |
| A_21_P0000612 | 0.017209308 | 2.8888502 | ANKRD30BP3 | Homo sapiens ankyrin repeat domain 30B pseudogene 3 (ANKRD30BP3), non-coding RNA [NR_033891] |
| A_33_P3403957 | 3.69E-04 | 3.0288305 |  |  |
| A_33_P3248843 | 0.003721795 | 2.5268173 |  |  |
| A_23_P155857 | 0.002366805 | -2.0775716 | NUDT6 | Homo sapiens nudix (nucleoside diphosphate linked moiety X)-type motif 6 (NUDT6), transcript variant 2, mRNA [NM_198041] |
| A_24_P27513 | 0.018723087 | 2.4618535 | CNTNAP4 | Homo sapiens contactin associated protein-like 4 (CNTNAP4), transcript variant 1, mRNA [NM_033401] |
| A_21_P0004154 | 0.005849091 | 2.4460893 |  |  |
| A_33_P3300877 | 0.018311022 | 3.0956771 |  |  |
| A_21_P0003416 | 4.68E-05 | 3.7075617 |  |  |
| A_33_P3370832 | 0.019715518 | 2.032728 | FAM117B | family with sequence similarity 117, member B [Source:HGNC Symbol;Acc:HGNC:14440] [ENST00000481658] |
| ERCC-00156_158 | 0.002705461 | 2.5712354 |  |  |
| A_23_P435874 | 0.003383152 | 5.1237392 | SPATA31A3 | Homo sapiens SPATA31 subfamily A, member 3 (SPATA31A3), mRNA [NM_001083124] |
| A_21_P0004135 | 0.002389498 | 2.5909293 | lnc-CCDC125-7 | LNCipedia lincRNA (lnc-CCDC125-7), lincRNA [lnc-CCDC125-7:1] |
| A_21_P0006486 | 0.015086644 | 2.349704 | lnc-CXorf22-1 | LNCipedia lincRNA (lnc-CXorf22-1), lincRNA [lnc-CXorf22-1:1] |
| A_22_P00017629 | 5.41E-04 | 2.5569496 | LOC101927822 | Homo sapiens uncharacterized LOC101927822 (LOC101927822), long non-coding RNA [NR_125424] |
| A_22_P00012170 | 2.45E-04 | 2.6813996 | LINC01444 | Homo sapiens long intergenic non-protein coding RNA 1444 (LINC01444), long non-coding RNA [NR_110783] |
| A_23_P92517 | 0.016666653 | 2.8319013 | TTC29 | Homo sapiens tetratricopeptide repeat domain 29 (TTC29), transcript variant 2, mRNA [NM_031956] |
| A_22_P00013323 | 0.009778747 | 2.9598866 |  |  |
| A_23_P52914 | 0.016380092 | 3.5150843 | OR4C15 | Homo sapiens olfactory receptor, family 4, subfamily C, member 15 (OR4C15), mRNA [NM_001001920] |
| A_33_P3680462 | 0.002001687 | 3.6197386 | LOC339539 | Homo sapiens uncharacterized LOC339539 (LOC339539), long non-coding RNA [NR_104171] |
| A_22_P00005062 | 0.004271837 | 4.698867 |  |  |
| A_21_P0006861 | 0.0192649 | 2.491618 | lnc-FXYD4-1 | LNCipedia lincRNA (lnc-FXYD4-1), lincRNA [lnc-FXYD4-1:1] |
| A_33_P3227360 | 0.011555376 | 3.675086 | DLX1 | distal-less homeobox 1 [Source:HGNC Symbol;Acc:HGNC:2914] [ENST00000409492] |
| A_22_P00022639 | 0.01604332 | 2.2215827 | LOC100506937 | PREDICTED: Homo sapiens uncharacterized LOC100506937 (LOC100506937), ncRNA [XR_108820] |
| A_24_P937317 | 2.27E-04 | 8.605902 | IL31 | Homo sapiens interleukin 31 (IL31), mRNA [NM_001014336] |
| A_33_P3274599 | 0.011475068 | 3.2467504 | FAM138E | Homo sapiens family with sequence similarity 138, member E (FAM138E), long non-coding RNA [NR_026819] |
| A_22_P00021809 | 0.0103703 | 2.009446 |  | ALU6_HUMAN (P39193) Alu subfamily SP sequence contamination warning entry, partial (6%) [THC2732521] |
| A_22_P00001373 | 0.005147894 | 3.1549842 | LOC101927738 | PREDICTED: Homo sapiens uncharacterized LOC101927738 (LOC101927738), ncRNA [XR_247236] |
| A_22_P00004616 | 0.002420827 | 2.5189452 | TTC21B-AS1 | Homo sapiens TTC21B antisense RNA 1 (TTC21B-AS1), transcript variant 1, long non-coding RNA [NR_038983] |
| A_21_P0006854 | 0.007604716 | 2.8963387 | lnc-C10orf68-4 | LNCipedia lincRNA (lnc-C10orf68-4), lincRNA [lnc-C10orf68-4:1] |
| A_21_P0008045 | 0.01683975 | 2.8653858 | lnc-B3GALTL-1 | LNCipedia lincRNA (lnc-B3GALTL-1), lincRNA [lnc-B3GALTL-1:1] |
| A_21_P0013437 | 0.003471922 | 4.144034 | XLOC_l2_013874 | BROAD Institute lincRNA (XLOC_l2_013874), lincRNA [TCONS_l2_00027392] |
| A_33_P3230578 | 0.001943492 | 3.6262264 |  |  |
| A_21_P0010497 | 0.001128016 | 4.3101983 | HOTTIP | Homo sapiens HOXA distal transcript antisense RNA (HOTTIP), long non-coding RNA [NR_037843] |
| A_21_P0004506 | 9.45E-04 | 2.0788455 | lnc-SREK1-1 | LNCipedia lincRNA (lnc-SREK1-1), lincRNA [lnc-SREK1-1:2] |
| A_22_P00007485 | 4.71E-04 | 2.8610184 | lnc-GTPBP8-2 | LNCipedia lincRNA (lnc-GTPBP8-2), lincRNA [lnc-GTPBP8-2:1] |
| A_22_P00015828 | 0.009373097 | 3.2549376 | LOC284930 | Homo sapiens uncharacterized LOC284930 (LOC284930), long non-coding RNA [NR_122046] |
| A_24_P68068 | 0.002104602 | 3.0097728 |  | Nanog homeobox pseudogene 1 [Source:HGNC Symbol;Acc:HGNC:23099] [ENST00000607111] |
| A_23_P105118 | 0.003672283 | 3.742061 | OR51G1 | Homo sapiens olfactory receptor, family 51, subfamily G, member 1 (gene/pseudogene) (OR51G1), mRNA [NM_001005237] |
| A_21_P0012617 | 0.00823943 | 2.5541315 | LINC00499 | long intergenic non-protein coding RNA 499 [Source:HGNC Symbol;Acc:HGNC:43436] [ENST00000502757] |
| A_21_P0003205 | 0.008900492 | 3.1199422 | lnc-CPN2-2 | LNCipedia lincRNA (lnc-CPN2-2), lincRNA [lnc-CPN2-2:4] |
| A_33_P3740020 | 8.68E-04 | 2.3371975 | SNORD9 | MR0-HT0559-270300-006-b11_1 HT0559 Homo sapiens cDNA, mRNA sequence [BE172294] |
| A_24_P532864 | 0.005823871 | 2.7273197 |  | RST7162 Athersys RAGE Library Homo sapiens cDNA, mRNA sequence [BG188151] |
| A_22_P00023393 | 5.72E-04 | 8.785993 |  |  |
| A_33_P3226109 | 0.01336487 | 2.4164228 | LCE4A | Homo sapiens late cornified envelope 4A (LCE4A), mRNA [NM_178356] |
| A_22_P00002593 | 0.007836055 | 2.2554343 |  |  |
| A_23_P351913 | 0.014106264 | 2.0363698 | LRRN4 | Homo sapiens leucine rich repeat neuronal 4 (LRRN4), mRNA [NM_152611] |
| A_21_P0005696 | 0.005865173 | 2.3128629 | lnc-SOX17-1 | LNCipedia lincRNA (lnc-SOX17-1), lincRNA [lnc-SOX17-1:1] |
| A_22_P00024139 | 3.69E-04 | 2.6821213 | lnc-CSPP1-1 | Homo sapiens cDNA clone IMAGE:5248337. [BC030256] |
| A_33_P3395008 | 0.016946392 | 2.0534165 | ACOXL | Homo sapiens acyl-CoA oxidase-like (ACOXL), mRNA [NM_001142807] |
| A_21_P0006254 | 0.004658177 | 3.2085001 | lnc-PTPRD-5 | LNCipedia lincRNA (lnc-PTPRD-5), lincRNA [lnc-PTPRD-5:2] |
| A_21_P0005330 | 0.011765111 | 5.8390636 |  |  |
| A_33_P3263664 | 0.01833653 | 3.06854 | KRTAP6-2 | Homo sapiens keratin associated protein 6-2 (KRTAP6-2), mRNA [NM_181604] |
| A_22_P00002601 | 0.007377652 | 3.292907 | lnc-C1QBP-1 | LNCipedia lincRNA (lnc-C1QBP-1), lincRNA [lnc-C1QBP-1:1] |
| A_23_P8497 | 0.015048197 | 3.4490507 | GHRHR | Homo sapiens growth hormone releasing hormone receptor (GHRHR), mRNA [NM_000823] |
| A_21_P0009698 | 0.012098864 | 3.6792603 | lnc-VSTM2B-7 | LNCipedia lincRNA (lnc-VSTM2B-7), lincRNA [lnc-VSTM2B-7:1] |
| A_22_P00014978 | 0.002389013 | 3.1358492 | lnc-SMARCC2-4 | LNCipedia lincRNA (lnc-SMARCC2-4), lincRNA [lnc-SMARCC2-4:1] |
| A_21_P0004346 | 0.014990651 | 2.6475189 | lnc-BTNL8-1 | LNCipedia lincRNA (lnc-BTNL8-1), lincRNA [lnc-BTNL8-1:1] |
| A_22_P00019680 | 1.37E-04 | 7.841973 |  |  |
| A_22_P00014780 | 0.01625216 | 2.4320967 | LOC102724163 | PREDICTED: Homo sapiens uncharacterized LOC102724163 (LOC102724163), ncRNA [XR_429783] |
| A_22_P00011352 | 0.01208652 | 2.1969342 | lnc-OVGP1-2 | LNCipedia lincRNA (lnc-OVGP1-2), lincRNA [lnc-OVGP1-2:1] |
| A_21_P0006838 | 0.002151727 | 3.1684175 | lnc-ARL5B-3 | LNCipedia lincRNA (lnc-ARL5B-3), lincRNA [lnc-ARL5B-3:1] |
| A_33_P3377176 | 0.015461167 | 6.9070215 |  | AL356489 T cell receptor beta variable 25/OR9-2 {Homo sapiens} (exp=0; wgp=1; cg=0), complete [THC2581026] |
| A_22_P00005135 | 0.017062232 | 2.1412363 | LOC440117 | Homo sapiens uncharacterized LOC440117 (LOC440117), long non-coding RNA [NR_033970] |
| A_22_P00017556 | 0.007014798 | 3.2961128 | lnc-WDR45L-2 | LNCipedia lincRNA (lnc-WDR45L-2), lincRNA [lnc-WDR45L-2:1] |
| A_22_P00025440 | 0.005147894 | 4.9205456 | EGFLAM-AS4 | Homo sapiens EGFLAM antisense RNA 4 (EGFLAM-AS4), long non-coding RNA [NR_046219] |
| A_22_P00014058 | 0.012042754 | 2.6888275 |  |  |
| A_21_P0012684 | 4.68E-05 | 3.1057649 | XLOC_l2_011289 | BROAD Institute lincRNA (XLOC_l2_011289), lincRNA [TCONS_l2_00021317] |
| A_21_P0003834 | 0.007794031 | 5.1103115 | lnc-POLR2B-1 | LNCipedia lincRNA (lnc-POLR2B-1), lincRNA [lnc-POLR2B-1:11] |
| A_22_P00004192 | 0.001652955 | 3.9578483 |  |  |
| A_21_P0009700 | 0.006968505 | 5.1916814 | LOC102724958 | Homo sapiens uncharacterized LOC102724958 (LOC102724958), long non-coding RNA [NR_110760] |
| A_21_P0005101 | 0.01693474 | -2.4753225 | lnc-WRNIP1-2 | LNCipedia lincRNA (lnc-WRNIP1-2), lincRNA [lnc-WRNIP1-2:20] |
| A_33_P3239037 | 0.01383093 | 2.6124432 | LINC00587 | Homo sapiens long intergenic non-protein coding RNA 587 (LINC00587), long non-coding RNA [NR_103830] |
| A_21_P0008420 | 0.005593198 | 2.826698 | lnc-RALGAPA1-3 | LNCipedia lincRNA (lnc-RALGAPA1-3), lincRNA [lnc-RALGAPA1-3:1] |
| A_33_P3305617 | 0.007796525 | 2.134123 | SPTSSB | serine palmitoyltransferase, small subunit B [Source:HGNC Symbol;Acc:HGNC:24045] [ENST00000617024] |
| A_21_P0005774 | 0.007681231 | 2.3765204 | lnc-DENND3-1 | LNCipedia lincRNA (lnc-DENND3-1), lincRNA [lnc-DENND3-1:1] |
| A_21_P0002469 | 3.69E-04 | 2.257309 | lnc-SFTPB-1 | LNCipedia lincRNA (lnc-SFTPB-1), lincRNA [lnc-SFTPB-1:1] |
| A_22_P00008354 | 0.010815636 | 4.124355 | LINC01350 | Homo sapiens long intergenic non-protein coding RNA 1350 (LINC01350), long non-coding RNA [NR_110793] |
| A_22_P00012574 | 0.008065707 | 4.043236 | BARX1-AS1 | BARX1 antisense RNA 1 (head to head) [Source:HGNC Symbol;Acc:HGNC:50673] [ENST00000453045] |
| A_23_P26062 | 0.010564335 | 3.4161587 | TMEM202 | Homo sapiens transmembrane protein 202 (TMEM202), mRNA [NM_001080462] |
| A_33_P3290792 | 0.008697676 | 2.4952378 | OR10G9 | Homo sapiens olfactory receptor, family 10, subfamily G, member 9 (OR10G9), mRNA [NM_001001953] |
| A_22_P00003603 | 0.015286709 | 2.8923838 | lnc-CD2AP-2 | zt77g02.r1 Soares_testis_NHT Homo sapiens cDNA clone IMAGE:728402 5' similar to gb:M21121 T-CELL SPECIFIC RANTES PROTEIN PRECURSOR (HUMAN);, mRNA sequence [AA397752] |
| A_22_P00020063 | 0.009440952 | 3.5690823 | lnc-TADA2B-1 | LNCipedia lincRNA (lnc-TADA2B-1), lincRNA [lnc-TADA2B-1:1] |
| A_22_P00021976 | 0.01109368 | 4.910488 | lnc-TIMP3-4 | LNCipedia lincRNA (lnc-TIMP3-4), lincRNA [lnc-TIMP3-4:1] |
| A_32_P97862 | 0.005823871 | 3.373934 | TEX38 | Homo sapiens testis expressed 38 (TEX38), transcript variant 1, mRNA [NM_001145474] |
| A_23_P144549 | 0.005923409 | 2.2313895 | IBSP | Homo sapiens integrin-binding sialoprotein (IBSP), mRNA [NM_004967] |
| A_22_P00003975 | 0.004682749 | 7.529939 |  | DB444431 RIKEN full-length enriched human cDNA library, testis Homo sapiens cDNA clone H013014O19 5', mRNA sequence [DB444431] |
| A_22_P00002008 | 0.00704779 | 3.0379913 |  | AGENCOURT_10012793 NIH_MGC_142 Homo sapiens cDNA clone IMAGE:6497858 5', mRNA sequence [BU602920] |
| A_33_P3400700 | 0.011051557 | 4.2077622 | SLC26A5 | Homo sapiens solute carrier family 26 (anion exchanger), member 5 (SLC26A5), transcript variant d, mRNA [NM_206885] |
| A_22_P00023161 | 0.014693059 | 2.28847 | lnc-AC016251.1-2 | LNCipedia lincRNA (lnc-AC016251.1-2), lincRNA [lnc-AC016251.1-2:1] |
| A_22_P00009725 | 0.003501274 | 3.4112945 | LINC01580 | Homo sapiens long intergenic non-protein coding RNA 1580 (LINC01580), transcript variant 2, long non-coding RNA [NR_120323] |
| A_22_P00019914 | 0.015457029 | 2.08924 | lnc-HCRTR1-2 | LNCipedia lincRNA (lnc-HCRTR1-2), lincRNA [lnc-HCRTR1-2:1] |
| A_23_P302302 | 0.004767705 | 3.575355 | LINC00917 | Homo sapiens long intergenic non-protein coding RNA 917 (LINC00917), long non-coding RNA [NR_024406] |
| A_23_P84230 | 0.001585742 | 6.906031 | OTP | Homo sapiens orthopedia homeobox (OTP), mRNA [NM_032109] |
| A_33_P3375145 | 0.006768369 | -2.8653064 | LURAP1L | Homo sapiens leucine rich adaptor protein 1-like (LURAP1L), mRNA [NM_203403] |
| A_23_P201551 | 0.016639534 | -2.3589392 | VAV3 | Homo sapiens vav 3 guanine nucleotide exchange factor (VAV3), transcript variant 1, mRNA [NM_006113] |
| A_24_P386375 | 0.001574271 | 7.1274166 | IFNA21 | Homo sapiens interferon, alpha 21 (IFNA21), mRNA [NM_002175] |
| A_23_P31725 | 0.001162504 | 4.027378 | BLK | Homo sapiens BLK proto-oncogene, Src family tyrosine kinase (BLK), mRNA [NM_001715] |
| A_21_P0008767 | 0.008129106 | 2.33539 | lnc-MEX3B-2 | LNCipedia lincRNA (lnc-MEX3B-2), lincRNA [lnc-MEX3B-2:2] |
| A_22_P00021225 | 0.019688005 | 2.6155715 |  | 603076828F1 NIH_MGC_119 Homo sapiens cDNA clone IMAGE:5168671 5', mRNA sequence [BI825887] |
| A_22_P00023201 | 0.017646626 | 2.6796708 | SCN1A | sodium channel, voltage-gated, type I, alpha subunit [Source:HGNC Symbol;Acc:HGNC:10585] [ENST00000507401] |
| A_22_P00008076 | 0.004550767 | 2.9815385 |  | DA152293 BRAMY2 Homo sapiens cDNA clone BRAMY2010517 5', mRNA sequence [DA152293] |
| A_21_P0009108 | 9.73E-04 | 2.9587438 | lnc-CDH5-3 | LNCipedia lincRNA (lnc-CDH5-3), lincRNA [lnc-CDH5-3:2] |
| A_21_P0007904 | 0.011555376 | 2.5796008 | lnc-SLC15A4-2 | LNCipedia lincRNA (lnc-SLC15A4-2), lincRNA [lnc-SLC15A4-2:3] |
| A_33_P3218450 | 0.014795829 | 2.2745008 | CDC6 | Homo sapiens cell division cycle 6 (CDC6), mRNA [NM_001254] |
| A_23_P53267 | 0.016926367 | -2.627916 | RSRC2 | Homo sapiens arginine/serine-rich coiled-coil 2 (RSRC2), transcript variant 1, mRNA [NM_023012] |
| A_33_P3795644 | 7.98E-04 | 3.5950415 | LINC00527 | Homo sapiens mRNA for hypothetical protein, partial. [AJ011409] |
| A_33_P3354604 | 0.013527386 | 2.0943658 | CCL4L2 | Homo sapiens chemokine (C-C motif) ligand 4-like 2 (CCL4L2), transcript variant CCL4L2b2, mRNA [NM_001291470] |
| A_22_P00014026 | 0.014642934 | 2.5578117 |  |  |
| A_21_P0006876 | 0.011555376 | 2.3942807 | lnc-C10orf107-1 | LNCipedia lincRNA (lnc-C10orf107-1), lincRNA [lnc-C10orf107-1:2] |
| A_23_P133637 | 0.016494371 | 2.9856815 | HTR4 | Homo sapiens 5-hydroxytryptamine (serotonin) receptor 4, G protein-coupled (HTR4), transcript variant d, mRNA [NM_001040172] |
| A_21_P0012545 | 0.011830683 | 3.2161915 | LINC00971 | long intergenic non-protein coding RNA 971 [Source:HGNC Symbol;Acc:HGNC:48737] [ENST00000491849] |
| A_21_P0008859 | 0.002384241 | 2.7303903 | lnc-RP11-210M15.2.1-3 | LNCipedia lincRNA (lnc-RP11-210M15.2.1-3), lincRNA [lnc-RP11-210M15.2.1-3:6] |
| A_22_P00021116 | 0.01682666 | 2.1565208 | lnc-MORF4L1-2 | Homo sapiens cDNA FLJ40018 fis, clone STOMA2006398. [AK097337] |
| A_21_P0007331 | 0.005823871 | 2.7013447 | lnc-MPPED2-2 | LNCipedia lincRNA (lnc-MPPED2-2), lincRNA [lnc-MPPED2-2:1] |
| A_22_P00018452 | 0.005823871 | 2.0657988 | LOC102723906 | PREDICTED: Homo sapiens uncharacterized LOC102723906 (LOC102723906), transcript variant X2, ncRNA [XR_425825] |
| A_21_P0008487 | 0.001976492 | 4.9356065 | lnc-BCL11B-1 | LNCipedia lincRNA (lnc-BCL11B-1), lincRNA [lnc-BCL11B-1:2] |
| A_21_P0012828 | 0.001162504 | 3.5035799 | XLOC_l2_011579 | BROAD Institute lincRNA (XLOC_l2_011579), lincRNA [TCONS_l2_00022305] |
| A_23_P129413 | 0.012090867 | 2.113709 | DPEP3 | Homo sapiens dipeptidase 3 (DPEP3), transcript variant 1, mRNA [NM_022357] |
| A_21_P0009924 | 8.60E-05 | 6.161424 |  | long intergenic non-protein coding RNA 29 [Source:HGNC Symbol;Acc:HGNC:16184] [ENST00000456634] |
| A_23_P393955 | 0.009464912 | 2.5991538 | NUTM1 | Homo sapiens NUT midline carcinoma, family member 1 (NUTM1), transcript variant 3, mRNA [NM_175741] |
| A_22_P00008349 | 0.005838869 | 3.6809473 | lnc-ITSN2-2 | 603072539F1 NIH_MGC_119 Homo sapiens cDNA clone IMAGE:5164617 5', mRNA sequence [BI825590] |
| A_21_P0000922 | 0.016855713 | 2.4143865 | LOC101927787 | Homo sapiens uncharacterized LOC101927787 (LOC101927787), long non-coding RNA [NR_125944] |
| A_33_P3325547 | 0.008953173 | 2.3049133 | C10orf53 | Homo sapiens chromosome 10 open reading frame 53 (C10orf53), transcript variant 1, mRNA [NM_182554] |
| A_22_P00018144 | 0.017963393 | 2.609566 |  | DB031156 TESTI2 Homo sapiens cDNA clone TESTI2015037 5', mRNA sequence [DB031156] |
| A_21_P0001805 | 0.01928657 | 4.9907126 |  | 603022609F1 NIH_MGC_114 Homo sapiens cDNA clone IMAGE:5193099 5', mRNA sequence [BI758452] |
| A_21_P0009667 | 2.59E-04 | 3.4079185 | LOC101928886 | Homo sapiens uncharacterized LOC101928886 (LOC101928886), long non-coding RNA [NR_110741] |
| A_21_P0005210 | 0.019925974 | 2.153772 | LOC101927354 | Homo sapiens uncharacterized LOC101927354 (LOC101927354), long non-coding RNA [NR_108073] |
| A_33_P3362915 | 0.007157139 | 4.519896 | GYPA | Homo sapiens glycophorin A (MNS blood group) (GYPA), mRNA [NM_002099] |
| A_32_P491499 | 0.007423808 | 5.959792 | LOC285191 | PREDICTED: Homo sapiens uncharacterized LOC285191 (AC011298.2), misc_RNA [XR_249328] |
| A_21_P0011557 | 0.006640496 | 4.484224 | XLOC_l2_005915 | BROAD Institute lincRNA (XLOC_l2_005915), lincRNA [TCONS_l2_00010999] |
| A_24_P160001 | 0.00654853 | -2.0048704 | FKBP1A | Homo sapiens FK506 binding protein 1A, 12kDa (FKBP1A), transcript variant 2, mRNA [NM_054014] |
| A_21_P0002113 | 0.007090495 | 2.412327 |  | Q39KM4_BURS3 (Q39KM4) Methyltransferase, partial (5%) [THC2653455] |
| A_21_P0005043 | 0.002813643 | 2.5130455 | lnc-GPR63-4 | LNCipedia lincRNA (lnc-GPR63-4), lincRNA [lnc-GPR63-4:1] |
| A_24_P932736 | 0.018211473 | -2.0078657 | HMBOX1 | homeobox containing 1 [Source:HGNC Symbol;Acc:HGNC:26137] [ENST00000397358] |
| A_23_P149427 | 0.015347909 | 2.2459555 | CAPN9 | Homo sapiens calpain 9 (CAPN9), transcript variant 1, mRNA [NM_006615] |
| A_22_P00001107 | 0.007173597 | 2.606405 | LOC101929418 | PREDICTED: Homo sapiens uncharacterized LOC101929418 (LOC101929418), ncRNA [XR_427015] |
| A_33_P3509233 | 0.012117199 | 6.439989 | LINC00877 | Homo sapiens long intergenic non-protein coding RNA 877 (LINC00877), long non-coding RNA [NR_104116] |
| A_21_P0008466 | 0.01150287 | 2.8829434 | lnc-ALKBH1-1 | LNCipedia lincRNA (lnc-ALKBH1-1), lincRNA [lnc-ALKBH1-1:1] |
| A_22_P00013874 | 0.006341876 | 3.2353342 | lnc-RPP38-3 | AGENCOURT_10279116 NIH_MGC_82 Homo sapiens cDNA clone IMAGE:6592391 5', mRNA sequence [BU561379] |
| A_33_P3314081 | 0.014343364 | 2.5973396 |  | DA959652 SPLEN2 Homo sapiens cDNA clone SPLEN2039783 5', mRNA sequence [DA959652] |
| A_21_P0004623 | 7.75E-06 | 4.0386887 | lnc-AL035696.1-3 | AF153341 winged helix/forkhead transcription factor {Homo sapiens} (exp=-1; wgp=0; cg=0), partial (6%) [THC2667051] |
| A_21_P0004650 | 0.009935914 | 7.1547885 | lnc-DAAM2-3 | Q3Y452_HUMAN (Q3Y452) Testis development related protein 1, partial (43%) [THC2725143] |
| A_23_P500542 | 1.37E-04 | 2.9994545 | DMBX1 | Homo sapiens diencephalon/mesencephalon homeobox 1 (DMBX1), transcript variant 2, mRNA [NM_147192] |
| A_22_P00011185 | 0.008383745 | 2.7232213 | LOC102724000 | Homo sapiens uncharacterized LOC102724000 (LOC102724000), long non-coding RNA [NR_121615] |
| A_22_P00009438 | 0.004106171 | 2.974 | SVIL-AS1 | Homo sapiens SVIL antisense RNA 1 (SVIL-AS1), transcript variant 10, long non-coding RNA [NR_110928] |
| A_24_P324712 | 0.017012546 | 2.1706936 | NPC1L1 | Homo sapiens NPC1-like 1 (NPC1L1), transcript variant 1, mRNA [NM_013389] |
| A_22_P00023704 | 0.0103703 | 5.0651927 |  |  |
| A_33_P3217357 | 0.013079802 | 3.028369 |  | long intergenic non-protein coding RNA 1415 [Source:HGNC Symbol;Acc:HGNC:50709] [ENST00000587320] |
| A_33_P3362291 | 0.019903338 | 4.2841864 | LINC01181 | PREDICTED: Homo sapiens long intergenic non-protein coding RNA 1181 (LINC01181), misc_RNA [XR_242417] |
| A_22_P00008782 | 0.005178843 | 3.7764838 |  |  |
| A_33_P3270147 | 0.017601259 | 2.1097176 | XLOC_l2_001687 | BROAD Institute lincRNA (XLOC_l2_001687), lincRNA [TCONS_l2_00003174] |
| A_22_P00007277 | 0.015057466 | 2.4029505 |  | long intergenic non-protein coding RNA 557 [Source:HGNC Symbol;Acc:HGNC:43701] [ENST00000563184] |
| A_22_P00014750 | 0.015173346 | 3.0733447 |  | 603199352F1 NIH_MGC_97 Homo sapiens cDNA clone IMAGE:5265444 5', mRNA sequence [BI459165] |
| A_21_P0008449 | 0.004280046 | 3.9225698 | lnc-MAX-2 | LNCipedia lincRNA (lnc-MAX-2), lincRNA [lnc-MAX-2:2] |
| A_24_P212811 | 0.012856201 | 2.7621067 | ANKRD34A | Homo sapiens ankyrin repeat domain 34A (ANKRD34A), mRNA [NM_001039888] |
| A_22_P00023730 | 0.017914722 | 2.1620808 |  |  |
| A_24_P724040 | 0.016912727 | 2.077576 | SNRPB2 | Homo sapiens small nuclear ribonucleoprotein polypeptide B (SNRPB2), transcript variant 1, mRNA [NM_003092] |
| A_21_P0007773 | 0.015286709 | 2.9144826 | lnc-TAOK3-7 | LNCipedia lincRNA (lnc-TAOK3-7), lincRNA [lnc-TAOK3-7:1] |
| A_21_P0012519 | 0.001652955 | 3.1174853 |  | DB231946 TRACH3 Homo sapiens cDNA clone TRACH3025715 5', mRNA sequence [DB231946] |
| A_19_P00316423 | 0.016926367 | -2.2599282 |  | tf44h11.x1 NCI_CGAP_Brn23 Homo sapiens cDNA clone IMAGE:2099109 3' similar to SW:IBA2_HUMAN Q14657 ITBA2 PROTEIN ;, mRNA sequence [AI421806] |
| A_21_P0000062 | 0.002705118 | 4.132007 | C22orf39 | Homo sapiens chromosome 22 open reading frame 39 (C22orf39), transcript variant 2, mRNA [NM_001166242] |
| A_33_P3307795 | 0.004047977 | 2.7541528 | FAM124B | family with sequence similarity 124B [Source:HGNC Symbol;Acc:HGNC:26224] [ENST00000243806] |
| A_33_P3560176 | 0.016420968 | 2.7340877 |  | BX096849 Soares_NFL_T_GBC_S1 Homo sapiens cDNA clone IMAGp998D093810, mRNA sequence [BX096849] |
| A_22_P00020041 | 0.001890564 | 4.8351216 | lnc-GREM2-3 | AF218941 formin 2-like protein {Homo sapiens} (exp=-1; wgp=0; cg=0), partial (15%) [THC2737915] |
| A_24_P341000 | 0.007835183 | 2.938748 | LMO7DN | Homo sapiens LMO7 downstream neighbor (LMO7DN), mRNA [NM_001257995] |
| A_22_P00023924 | 0.008852229 | 2.128568 | lnc-OBFC2A-5 | LNCipedia lincRNA (lnc-OBFC2A-5), lincRNA [lnc-OBFC2A-5:1] |
| A_21_P0012587 | 5.75E-04 | 2.5192997 | XLOC_l2_010691 | BROAD Institute lincRNA (XLOC_l2_010691), lincRNA [TCONS_l2_00020549] |
| A_33_P3307207 | 1.37E-04 | 5.0007377 | URAD | Homo sapiens ureidoimidazoline (2-oxo-4-hydroxy-4-carboxy-5-) decarboxylase (URAD), mRNA [NM_001105577] |
| A_33_P3375435 | 0.006488535 | -2.9218328 | AGAP11 | Homo sapiens ankyrin repeat and GTPase domain Arf GTPase activating protein 11 (AGAP11), mRNA [NM_133447] |
| A_23_P102286 | 0.002277691 | 5.654469 | LHCGR | Homo sapiens luteinizing hormone/choriogonadotropin receptor (LHCGR), mRNA [NM_000233] |
| A_21_P0001154 | 6.73E-04 | 5.035094 |  | ALU7_HUMAN (P39194) Alu subfamily SQ sequence contamination warning entry, partial (16%) [THC2681830] |
| A_23_P379630 | 0.016346738 | 2.8389926 | SLC38A10 | Homo sapiens solute carrier family 38, member 10 (SLC38A10), transcript variant 2, mRNA [NM_138570] |
| A_33_P3402654 | 0.003624071 | 2.2432158 | LINC00643 | Homo sapiens long intergenic non-protein coding RNA 643 (LINC00643), long non-coding RNA [NR_015358] |
| A_21_P0000111 | 0.015893705 | 5.8709116 | KLRF2 | Homo sapiens killer cell lectin-like receptor subfamily F, member 2 (KLRF2), mRNA [NM_001190765] |
| A_22_P00023387 | 0.011058508 | 3.7949843 | FLJ46363 | PREDICTED: Homo sapiens uncharacterized FLJ46363 (FLJ46363), transcript variant X1, misc_RNA [XR_253017] |
| A_33_P3367356 | 4.72E-04 | 6.3891473 |  | Homo sapiens chromosome 15 open reading frame 45, mRNA (cDNA clone MGC:168991 IMAGE:9021368), complete cds. [BC137370] |
| A_33_P8916977 | 0.018027226 | 2.9930418 | LOC286083 | Homo sapiens uncharacterized LOC286083 (LOC286083), long non-coding RNA [NR_111948] |
| A_19_P00319404 | 0.015121243 | -2.719045 | LINC00472 | Homo sapiens long intergenic non-protein coding RNA 472 (LINC00472), transcript variant 1, long non-coding RNA [NR_121612] |
| A_33_P3423845 | 0.011283191 | 2.0519085 |  |  |
| A_33_P3673544 | 0.016883848 | 2.1222568 |  |  |
| A_21_P0006387 | 0.017040258 | 2.212733 | lnc-ERP44-3 | LNCipedia lincRNA (lnc-ERP44-3), lincRNA [lnc-ERP44-3:5] |
| A_33_P3248939 | 0.002801913 | 3.9643304 |  |  |
| A_21_P0008748 | 0.01963507 | 2.122645 | lnc-PIF1-1 | LNCipedia lincRNA (lnc-PIF1-1), lincRNA [lnc-PIF1-1:1] |
| A_21_P0012435 | 0.003419576 | 4.5124803 | LINC01267 | Homo sapiens long intergenic non-protein coding RNA 1267 (LINC01267), long non-coding RNA [NR_110135] |
| A_21_P0004332 | 0.014575909 | 3.3273091 | lnc-NEURL1B-4 | LNCipedia lincRNA (lnc-NEURL1B-4), lincRNA [lnc-NEURL1B-4:2] |
| A_22_P00002161 | 0.006165735 | 2.9047084 |  |  |
| A_22_P00017112 | 0.014150378 | 2.0208209 |  |  |
| A_22_P00000982 | 0.005339755 | 2.3828158 | lnc-AL136219.1-3 | LNCipedia lincRNA (lnc-AL136219.1-3), lincRNA [lnc-AL136219.1-3:1] |
| A_21_P0005560 | 0.005597494 | 2.146835 | lnc-ABCA13-2 | LNCipedia lincRNA (lnc-ABCA13-2), lincRNA [lnc-ABCA13-2:1] |
| A_33_P3400918 | 0.015958356 | 2.4584818 | DISC1 | Homo sapiens disrupted in schizophrenia 1 (DISC1), transcript variant m, mRNA [NM_001164550] |
| A_21_P0010159 | 0.003686147 | 2.0727708 | LINC00945 | Homo sapiens long intergenic non-protein coding RNA 945 (LINC00945), long non-coding RNA [NR_104056] |
| A_21_P0007471 | 0.004871668 | 4.4063435 |  |  |
| A_21_P0008623 | 0.007796347 | 5.6418977 |  |  |
| A_23_P409888 | 0.004017944 | 3.5539067 | FAM83C | Homo sapiens family with sequence similarity 83, member C (FAM83C), mRNA [NM_178468] |
| A_33_P3421990 | 9.70E-04 | 9.762954 | lnc-OTUD4-1 | LNCipedia lincRNA (lnc-OTUD4-1), lincRNA [lnc-OTUD4-1:1] |
| A_21_P0009912 | 0.017343516 | 2.3468487 |  |  |
| A_22_P00025283 | 0.019697012 | 2.1167312 | lnc-H2AFV-1 | LNCipedia lincRNA (lnc-H2AFV-1), lincRNA [lnc-H2AFV-1:1] |
| A_21_P0009370 | 0.018820966 | 2.2396462 | lnc-SECTM1-1 | LNCipedia lincRNA (lnc-SECTM1-1), lincRNA [lnc-SECTM1-1:1] |
| A_22_P00010673 | 0.005533089 | 2.5658906 | lnc-NEURL1B-1 | LNCipedia lincRNA (lnc-NEURL1B-1), lincRNA [lnc-NEURL1B-1:1] |
| A_33_P3408938 | 0.017076183 | 3.4495482 | LILRP2 | Homo sapiens leukocyte immunoglobulin-like receptor pseudogene 2 (LILRP2), non-coding RNA [NR_003061] |
| A_23_P303810 | 0.019137397 | -2.4007545 | CRYBG3 | Homo sapiens beta-gamma crystallin domain containing 3 (CRYBG3), mRNA [NM_153605] |
| A_33_P3242733 | 7.57E-04 | -3.448849 | PKDCC | Homo sapiens protein kinase domain containing, cytoplasmic (PKDCC), mRNA [NM_138370] |
| A_22_P00003496 | 0.012917864 | 2.0844727 | LOC101928354 | PREDICTED: Homo sapiens uncharacterized LOC101928354 (RP11-330A16.1), ncRNA [XR_241980] |
| A_21_P0001331 | 0.010407414 | 4.836669 | LOC101927871 | PREDICTED: Homo sapiens uncharacterized LOC101927871 (LOC101927871), transcript variant X1, ncRNA [XR_246366] |
| A_33_P3333485 | 0.01683975 | 3.1901944 |  |  |
| A_23_P259166 | 0.013346144 | -2.4715965 | TCEAL4 | Homo sapiens transcription elongation factor A (SII)-like 4 (TCEAL4), transcript variant 1, mRNA [NM_024863] |
| A_33_P3208975 | 0.01693474 | 2.597046 | OR51S1 | Homo sapiens olfactory receptor, family 51, subfamily S, member 1 (OR51S1), mRNA [NM_001004758] |
| A_23_P205567 | 0.005164835 | 2.5089164 | PRKCH | Homo sapiens protein kinase C, eta (PRKCH), mRNA [NM_006255] |
| A_22_P00008699 | 5.71E-04 | 2.109457 | lnc-KIAA1217-1 | LNCipedia lincRNA (lnc-KIAA1217-1), lincRNA [lnc-KIAA1217-1:2] |
| A_22_P00006766 | 0.007796347 | 2.8759537 | LOC100505530 | Homo sapiens uncharacterized LOC100505530 (LOC100505530), long non-coding RNA [NR_126057] |
| A_21_P0007716 | 3.77E-04 | 3.0477645 | lnc-AVPR1A-1 | LNCipedia lincRNA (lnc-AVPR1A-1), lincRNA [lnc-AVPR1A-1:1] |
| A_23_P300033 | 0.008252968 | -3.1475897 | PDGFRA | Homo sapiens platelet-derived growth factor receptor, alpha polypeptide (PDGFRA), mRNA [NM_006206] |
| A_33_P3225507 | 0.010939572 | 4.6476088 | OR10G2 | Homo sapiens olfactory receptor, family 10, subfamily G, member 2 (OR10G2), mRNA [NM_001005466] |
| A_23_P25246 | 0.017806867 | -2.9775226 | AVPR1A | Homo sapiens arginine vasopressin receptor 1A (AVPR1A), mRNA [NM_000706] |
| A_22_P00025522 | 0.003916526 | 2.2018678 | CLEC4GP1 | Homo sapiens C-type lectin domain family 4, member G pseudogene 1 (CLEC4GP1), non-coding RNA [NR_002931] |
| A_22_P00010044 | 0.006488535 | 2.9680414 | lnc-MNX1-2 | LNCipedia lincRNA (lnc-MNX1-2), lincRNA [lnc-MNX1-2:1] |
| A_23_P91970 | 0.002526455 | 5.382169 | AADACL2 | Homo sapiens arylacetamide deacetylase-like 2 (AADACL2), mRNA [NM_207365] |
| A_22_P00013426 | 0.001860961 | 2.7169545 |  |  |
| A_33_P3325685 | 0.019951195 | 2.184423 | SMIM21 | small integral membrane protein 21 [Source:HGNC Symbol;Acc:HGNC:27598] [ENST00000579022] |
| A_19_P00806883 | 0.017647024 | 3.1254044 |  | Homo sapiens cDNA FLJ30260 fis, clone BRACE2002495. [AK054822] |
| A_22_P00004210 | 0.003383524 | 2.3129778 | SRD5A3-AS1 | Homo sapiens SRD5A3 antisense RNA 1 (SRD5A3-AS1), long non-coding RNA [NR_037969] |
| A_21_P0004248 | 0.002801913 | 2.1423035 | lnc-MRPS30-4 | LNCipedia lincRNA (lnc-MRPS30-4), lincRNA [lnc-MRPS30-4:1] |
| A_22_P00007066 | 1.01E-04 | 6.6266646 |  |  |
| A_23_P301521 | 0.008289699 | -2.029442 | KIAA1462 | Homo sapiens KIAA1462 (KIAA1462), mRNA [NM_020848] |
| A_21_P0007751 | 7.96E-04 | 3.1134036 | lnc-C12orf42-1 | LNCipedia lincRNA (lnc-C12orf42-1), lincRNA [lnc-C12orf42-1:1] |
| A_33_P3235721 | 0.003686147 | 2.386883 | C11orf87 | Homo sapiens chromosome 11 open reading frame 87 (C11orf87), mRNA [NM_207645] |
| A_21_P0013496 | 0.001677055 | 6.4482946 | XLOC_l2_014234 | BROAD Institute lincRNA (XLOC_l2_014234), lincRNA [TCONS_l2_00027818] |
| A_24_P366859 | 2.62E-04 | 2.6907551 | USHBP1 | Homo sapiens Usher syndrome 1C binding protein 1 (USHBP1), transcript variant 1, mRNA [NM_031941] |
| A_22_P00008534 | 0.003292375 | 3.030879 |  | AGENCOURT_7905480 NIH_MGC_82 Homo sapiens cDNA clone IMAGE:6105748 5', mRNA sequence [BQ438646] |
| A_22_P00012314 | 0.008104733 | 2.083948 | lnc-PPP6C-1 | LNCipedia lincRNA (lnc-PPP6C-1), lincRNA [lnc-PPP6C-1:1] |
| A_21_P0011762 | 0.009450137 | 2.7663488 | LOC284379 |  |
| A_21_P0009040 | 0.002770355 | 2.5716207 | lnc-CES5A-1 | LNCipedia lincRNA (lnc-CES5A-1), lincRNA [lnc-CES5A-1:1] |
| A_33_P3310327 | 0.011796018 | 2.5914397 |  |  |
| A_21_P0005487 | 0.017647024 | 4.674846 | lnc-RP1-170O19.17.1-3 | LNCipedia lincRNA (lnc-RP1-170O19.17.1-3), lincRNA [lnc-RP1-170O19.17.1-3:1] |
| A_24_P357406 | 0.015121243 | 2.0938365 | RBMY1B | Homo sapiens RNA binding motif protein, Y-linked, family 1, member B (RBMY1B), mRNA [NM_001006121] |
| A_23_P33759 | 0.012034409 | -2.5167267 | DHRS3 | Homo sapiens dehydrogenase/reductase (SDR family) member 3 (DHRS3), mRNA [NM_004753] |
| A_33_P3279847 | 0.00665872 | 3.2621636 | RAET1E | Homo sapiens retinoic acid early transcript 1E (RAET1E), transcript variant 1, mRNA [NM_139165] |
| A_23_P118203 | 0.007733543 | 2.248504 | ZG16B | Homo sapiens zymogen granule protein 16B (ZG16B), mRNA [NM_145252] |
| A_23_P435636 | 0.001162504 | 5.153986 | DAND5 | Homo sapiens DAN domain family member 5, BMP antagonist (DAND5), mRNA [NM_152654] |
| A_21_P0002455 | 0.008645061 | 2.1686454 | lnc-C1D-3 | LNCipedia lincRNA (lnc-C1D-3), lincRNA [lnc-C1D-3:1] |
| A_22_P00007102 | 0.019923652 | 3.1831446 | LOC100506999 | PREDICTED: Homo sapiens uncharacterized LOC100506999 (LOC100506999), ncRNA [XR_110241] |
| A_22_P00014370 | 0.010613757 | 2.6540425 |  | PREDICTED: Homo sapiens uncharacterized LOC102723523 (LOC102723523), ncRNA [XR_424709] |
| A_21_P0013031 | 0.003729382 | 2.6035628 | XLOC_l2_012661 | BROAD Institute lincRNA (XLOC_l2_012661), lincRNA [TCONS_l2_00024354] |
| A_21_P0013377 | 0.017850475 | 3.3571904 | LOC100506289 |  |
| A_21_P0004327 | 0.001585742 | 3.0683372 | lnc-MAT2B-3 | LNCipedia lincRNA (lnc-MAT2B-3), lincRNA [lnc-MAT2B-3:15] |
| A_22_P00005367 | 0.002277691 | 2.447777 |  |  |
| A_21_P0009958 | 0.016926367 | 3.9832387 | lnc-PI3-1 | LNCipedia lincRNA (lnc-PI3-1), lincRNA [lnc-PI3-1:5] |
| A_21_P0013217 | 0.005949104 | 4.242812 | XLOC_l2_013448 | BROAD Institute lincRNA (XLOC_l2_013448), lincRNA [TCONS_l2_00025938] |
| A_33_P3215317 | 0.006584617 | 2.049581 | PRKACB | Homo sapiens protein kinase, cAMP-dependent, catalytic, beta (PRKACB), transcript variant 12, mRNA [NM_001300917] |
| A_33_P3374970 | 0.00654853 | 2.8125975 | TRIM49D1 | Homo sapiens tripartite motif containing 49D1 (TRIM49D1), mRNA [NM_001206627] |
| A_21_P0014487 | 0.009337066 | 3.4447575 | KRBOX1-AS1 | Homo sapiens KRBOX1 antisense RNA 1 (KRBOX1-AS1), long non-coding RNA [NR_122033] |
| A_22_P00008326 | 4.01E-04 | 4.962049 | lnc-ITIH2-3 | BX461541 Homo sapiens FETAL BRAIN Homo sapiens cDNA clone CS0DF029YC11 5-PRIME, mRNA sequence [BX461541] |
| A_22_P00018944 | 3.97E-04 | 8.159055 |  | Homo sapiens cDNA FLJ43688 fis, clone TBAES2003492. [AK125676] |
| A_33_P3343506 | 9.45E-04 | 5.188316 | OR5M3 | Homo sapiens olfactory receptor, family 5, subfamily M, member 3 (OR5M3), mRNA [NM_001004742] |
| A_22_P00004895 | 0.0192649 | 2.4918785 |  |  |
| A_21_P0004269 | 8.61E-04 | 2.96523 | lnc-CMYA5-1 | LNCipedia lincRNA (lnc-CMYA5-1), lincRNA [lnc-CMYA5-1:1] |
| A_23_P424900 | 0.007446792 | 2.4177368 | PIFO | Homo sapiens primary cilia formation (PIFO), transcript variant 1, mRNA [NM_181643] |
| A_24_P263310 | 0.00704779 | 3.6209538 | FAM169B | Homo sapiens family with sequence similarity 169, member B (FAM169B), mRNA [NM_182562] |
| A_23_P47941 | 3.69E-04 | 2.6160116 | HOXC11 | Homo sapiens homeobox C11 (HOXC11), mRNA [NM_014212] |
| A_23_P126658 | 0.002705118 | 5.5324407 | PRAMEF8 | Homo sapiens PRAME family member 8 (PRAMEF8), mRNA [NM_001012276] |
| A_21_P0009531 | 0.012042754 | 3.067159 | lnc-MPPE1-1 | LNCipedia lincRNA (lnc-MPPE1-1), lincRNA [lnc-MPPE1-1:1] |
| A_33_P3560878 | 0.007348139 | 2.0310102 | LINC01146 | Homo sapiens long intergenic non-protein coding RNA 1146 (LINC01146), long non-coding RNA [NR_046094] |
| A_22_P00004987 | 2.99E-04 | 5.003153 | LINC00290 | Homo sapiens long intergenic non-protein coding RNA 290 (LINC00290), long non-coding RNA [NR_033918] |
| A_21_P0006479 | 6.91E-05 | 2.815475 | lnc-CXorf28-1 | LNCipedia lincRNA (lnc-CXorf28-1), lincRNA [lnc-CXorf28-1:1] |
| A_22_P00003478 | 0.008821026 | 4.300465 | lnc-CCDC71L-1 | LNCipedia lincRNA (lnc-CCDC71L-1), lincRNA [lnc-CCDC71L-1:1] |
| A_33_P3263747 | 0.008503397 | 2.699127 |  |  |
| A_21_P0007336 | 0.005090478 | 2.9400582 | lnc-LMO2-1 | LNCipedia lincRNA (lnc-LMO2-1), lincRNA [lnc-LMO2-1:1] |
| A_21_P0013033 | 8.45E-05 | 2.4853947 | CCDC162P | coiled-coil domain containing 162, pseudogene [Source:HGNC Symbol;Acc:HGNC:21565] [ENST00000508210] |
| A_23_P60079 | 0.01865728 | -2.2616613 | ANGPT2 | Homo sapiens angiopoietin 2 (ANGPT2), transcript variant 1, mRNA [NM_001147] |
| A_21_P0013954 | 0.011555376 | 5.2664332 |  |  |
| A_21_P0007062 | 0.008900492 | -2.5251896 | LINC00844 | Homo sapiens long intergenic non-protein coding RNA 844 (LINC00844), long non-coding RNA [NR_108046] |
| A_23_P428366 | 0.011913257 | 2.4302504 | HORMAD2 | Homo sapiens HORMA domain containing 2 (HORMAD2), mRNA [NM_152510] |
| A_23_P143817 | 0.016810589 | -2.0137663 | MYLK | Homo sapiens myosin light chain kinase (MYLK), transcript variant 1, mRNA [NM_053025] |
| A_22_P00009699 | 0.010518414 | 2.1192963 |  |  |
| A_22_P00006201 | 0.015568356 | 3.021244 | SMKR1 | Homo sapiens small lysine-rich protein 1 (SMKR1), mRNA [NM_001195243] |
| A_22_P00009104 | 0.009724057 | 2.8718784 | lnc-LIFR-4 | LNCipedia lincRNA (lnc-LIFR-4), lincRNA [lnc-LIFR-4:1] |
| A_23_P2083 | 0.015294367 | 3.207799 | ASCL3 | Homo sapiens achaete-scute family bHLH transcription factor 3 (ASCL3), mRNA [NM_020646] |
| A_22_P00015641 | 0.010557873 | 4.8762846 | lnc-SUPT3H-1 | LNCipedia lincRNA (lnc-SUPT3H-1), lincRNA [lnc-SUPT3H-1:6] |
| A_23_P123308 | 0.018723087 | 4.3015547 | TEX15 | Homo sapiens testis expressed 15 (TEX15), mRNA [NM_031271] |
| A_21_P0001380 | 4.02E-04 | 4.448676 | lnc-NOS1AP-1 | LNCipedia lincRNA (lnc-NOS1AP-1), lincRNA [lnc-NOS1AP-1:1] |
| A_22_P00011278 | 0.012954351 | 4.095307 | LOC101927120 | Homo sapiens uncharacterized LOC101927120 (LOC101927120), transcript variant 1, long non-coding RNA [NR_110136] |
| A_33_P3289401 | 0.01150287 | 3.4398685 | OR52J3 | Homo sapiens olfactory receptor, family 52, subfamily J, member 3 (OR52J3), mRNA [NM_001001916] |
| A_22_P00003140 | 0.006437182 | 3.358077 | PTCSC2 | PREDICTED: Homo sapiens uncharacterized LOC101928337 (RP11-23B15.1), transcript variant X4, ncRNA [XR_428581] |
| A_21_P0006251 | 0.010888848 | 3.0824914 | lnc-C9orf123-1 | LNCipedia lincRNA (lnc-C9orf123-1), lincRNA [lnc-C9orf123-1:1] |
| A_21_P0007232 | 0.015201804 | 3.9701114 | lnc-NADSYN1-2 | LNCipedia lincRNA (lnc-NADSYN1-2), lincRNA [lnc-NADSYN1-2:1] |
| A_22_P00018597 | 0.002139024 | 4.815428 |  |  |
| A_22_P00003263 | 0.003917424 | 2.6647437 | LINC00642 | long intergenic non-protein coding RNA 642 [Source:HGNC Symbol;Acc:HGNC:44293] [ENST00000444942] |
| A_33_P3319248 | 0.013260878 | 6.4744287 | ZNF705G | Homo sapiens zinc finger protein 705G (ZNF705G), mRNA [NM_001164457] |
| A_23_P45361 | 0.015618757 | -2.875125 | GLUD2 | Homo sapiens glutamate dehydrogenase 2 (GLUD2), mRNA [NM_012084] |
| A_21_P0005305 | 3.77E-04 | 3.8282874 |  |  |
| A_21_P0002818 | 0.001245341 | 4.421177 | lnc-EIF4A2-1 | DB302275 BRAMY2 Homo sapiens cDNA clone BRAMY2024252 3', mRNA sequence [DB302275] |
| A_22_P00001912 | 0.00704779 | 2.4806058 | PRIM2 | Homo sapiens primase, DNA, polypeptide 2 (58kDa) (PRIM2), transcript variant 1, mRNA [NM_000947] |
| A_21_P0001257 | 0.017359223 | 3.121543 | lnc-DFFB-4 | LNCipedia lincRNA (lnc-DFFB-4), lincRNA [lnc-DFFB-4:1] |
| A_33_P3329063 | 0.009464912 | 2.3248034 | NCR1 | Homo sapiens natural cytotoxicity triggering receptor 1 (NCR1), transcript variant 1, mRNA [NM_004829] |
| A_33_P3236213 | 0.010815636 | 4.804328 |  |  |
| A_33_P3256565 | 0.009028136 | 2.231255 |  |  |
| A_22_P00025414 | 0.015121832 | 2.6396146 | LOC102723308 | Homo sapiens cDNA FLJ32811 fis, clone TESTI2002752. [AK057373] |
| A_33_P3295119 | 0.01601929 | 3.615143 | LYPD4 | Homo sapiens LY6/PLAUR domain containing 4 (LYPD4), transcript variant 1, mRNA [NM_173506] |
| A_24_P67395 | 0.01693474 | 2.1199028 | KRT8 | Homo sapiens keratin 8, type II (KRT8), transcript variant 2, mRNA [NM_002273] |
| A_24_P322635 | 0.015840286 | -2.9072602 | ELMO2 | Homo sapiens engulfment and cell motility 2 (ELMO2), transcript variant 2, mRNA [NM_182764] |
| A_21_P0002288 | 0.00654853 | 3.8993628 | lnc-CNTNAP5-1 | LNCipedia lincRNA (lnc-CNTNAP5-1), lincRNA [lnc-CNTNAP5-1:1] |
| A_23_P149529 | 0.010797242 | -3.2010422 | TACSTD2 | Homo sapiens tumor-associated calcium signal transducer 2 (TACSTD2), mRNA [NM_002353] |
| A_23_P47967 | 0.002580904 | 2.9429784 | LHX5 | Homo sapiens LIM homeobox 5 (LHX5), mRNA [NM_022363] |
| A_22_P00004875 | 0.013589985 | 3.33911 |  |  |
| A_21_P0007341 | 0.018425573 | 4.803662 | LOC103312105 | Homo sapiens uncharacterized LOC103312105 (LOC103312105), transcript variant 2, long non-coding RNA [NR_120555] |
| A_22_P00010681 | 0.014688863 | 2.9865355 |  |  |
| A_21_P0001422 | 0.014343364 | 2.7498846 | lnc-LYPLAL1-3 | LNCipedia lincRNA (lnc-LYPLAL1-3), lincRNA [lnc-LYPLAL1-3:2] |
| A_21_P0004721 | 0.008645061 | 2.823811 | LOC101927888 | PREDICTED: Homo sapiens uncharacterized LOC101927888 (LOC101927888), ncRNA [XR_241961] |
| A_21_P0006864 | 0.013996482 | 2.8377883 | lnc-C10orf136-1 | LNCipedia lincRNA (lnc-C10orf136-1), lincRNA [lnc-C10orf136-1:3] |
| A_22_P00020862 | 0.013260091 | 2.8779705 | lnc-NAPB-2 | 601435234F1 NIH_MGC_72 Homo sapiens cDNA clone IMAGE:3920217 5', mRNA sequence [BE891551] |
| A_33_P3325250 | 0.005983065 | 5.130159 |  |  |
| A_23_P145089 | 0.01437141 | -2.2565427 | HSP90AB1 | Homo sapiens heat shock protein 90kDa alpha (cytosolic), class B member 1 (HSP90AB1), transcript variant 2, mRNA [NM_007355] |
| A_33_P3274955 | 0.016781604 | 2.0447319 | TKTL2 | Homo sapiens transketolase-like 2 (TKTL2), mRNA [NM_032136] |
| A_22_P00009659 | 0.008821026 | 2.805884 | LOC101929406 | Homo sapiens uncharacterized LOC101929406 (LOC101929406), long non-coding RNA [NR_110790] |
| A_23_P395566 | 0.002620795 | -2.3441195 | FBXO31 | Homo sapiens F-box protein 31 (FBXO31), transcript variant 1, mRNA [NM_024735] |
| A_22_P00016082 | 0.002526455 | 2.1291952 | lnc-THBS3-1 | LNCipedia lincRNA (lnc-THBS3-1), lincRNA [lnc-THBS3-1:1] |
| A_32_P67623 | 0.007542613 | -2.237641 | FAM120C | Homo sapiens family with sequence similarity 120C (FAM120C), transcript variant 3, mRNA [NM_001300788] |
| A_22_P00011835 | 0.001976492 | 3.0046468 |  | Homo sapiens mRNA; cDNA DKFZp313N076 (from clone DKFZp313N076). [BX537873] |
| A_21_P0002140 | 0.018025374 | 2.269549 | lnc-OBSL1-1 | LNCipedia lincRNA (lnc-OBSL1-1), lincRNA [lnc-OBSL1-1:1] |
| A_33_P3333282 | 0.014502258 | 2.4851305 | FGF11 | fibroblast growth factor 11 [Source:HGNC Symbol;Acc:HGNC:3667] [ENST00000293829] |
| A_23_P349373 | 0.002131335 | 8.369575 |  | IQ motif and Sec7 domain 3 [Source:HGNC Symbol;Acc:HGNC:29193] [ENST00000621764] |
| A_23_P140423 | 0.017703129 | -2.8417313 | NDUFB1 | Homo sapiens NADH dehydrogenase (ubiquinone) 1 beta subcomplex, 1, 7kDa (NDUFB1), mRNA [NM_004545] |
| A_22_P00022949 | 0.001719849 | 7.333712 | lnc-ATAD1-1 | LNCipedia lincRNA (lnc-ATAD1-1), lincRNA [lnc-ATAD1-1:1] |
| A_23_P90497 | 0.018820966 | 2.0808427 | LILRA4 | Homo sapiens leukocyte immunoglobulin-like receptor, subfamily A (with TM domain), member 4 (LILRA4), mRNA [NM_012276] |
| A_22_P00021185 | 0.017963393 | 2.317437 | lnc-RFC5-1 | LNCipedia lincRNA (lnc-RFC5-1), lincRNA [lnc-RFC5-1:1] |
| A_21_P0012704 | 0.011502676 | 2.832376 | XLOC_l2_011423 | BROAD Institute lincRNA (XLOC_l2_011423), lincRNA [TCONS_l2_00021489] |
| A_22_P00012997 | 0.009052684 | 2.0553367 | LOC101927640 | Homo sapiens uncharacterized LOC101927640 (LOC101927640), long non-coding RNA [NR_121193] |
| A_21_P0007994 | 0.011646548 | 2.2336926 | LINC00462 | Homo sapiens long intergenic non-protein coding RNA 462 (LINC00462), long non-coding RNA [NR_051983] |
| A_33_P3210180 | 0.011675597 | 5.185099 | TLE1 | Homo sapiens clone FP17926 unknown mRNA. [AY129016] |
| A_32_P38637 | 0.015893705 | -2.5081928 | KRBA1 | Homo sapiens KRAB-A domain containing 1 (KRBA1), transcript variant 1, mRNA [NM_032534] |
| A_23_P78664 | 0.018319929 | -2.7875443 | DDX39A | Homo sapiens DEAD (Asp-Glu-Ala-Asp) box polypeptide 39A (DDX39A), transcript variant 1, mRNA [NM_005804] |
| A_33_P3259817 | 0.009551783 | -3.767657 | DOCK9 | Homo sapiens dedicator of cytokinesis 9 (DOCK9), transcript variant 2, mRNA [NM_001130048] |
| A_21_P0009374 | 0.018820966 | 2.1300116 | lnc-FAM57A-1 | LNCipedia lincRNA (lnc-FAM57A-1), lincRNA [lnc-FAM57A-1:1] |
| A_22_P00022688 | 0.016883848 | 2.3298345 | lnc-CCL2-3 | LNCipedia lincRNA (lnc-CCL2-3), lincRNA [lnc-CCL2-3:1] |
| A_33_P3330761 | 0.003693373 | 2.7321079 |  |  |
| A_24_P306304 | 1.16E-04 | 6.3271 | PRAMEF16 | Homo sapiens PRAME family member 16 (PRAMEF16), mRNA [NM_001045480] |
| A_33_P3283700 | 0.014765953 | 5.0124006 |  |  |
| A_22_P00002191 | 0.01688473 | 2.4299872 | TXNDC12-AS1 | Homo sapiens TXNDC12 antisense RNA 1 (TXNDC12-AS1), long non-coding RNA [NR_126385] |
| A_32_P4403 | 0.007348139 | -2.6042626 | C16orf72 | Homo sapiens chromosome 16 open reading frame 72 (C16orf72), mRNA [NM_014117] |
| A_33_P3238182 | 0.002580904 | 3.565642 | LINC00588 | Homo sapiens long intergenic non-protein coding RNA 588 (LINC00588), long non-coding RNA [NR_026772] |
| A_22_P00011090 | 0.0072738 | 3.0139844 | lnc-NUDT11-2 | LNCipedia lincRNA (lnc-NUDT11-2), lincRNA [lnc-NUDT11-2:1] |
| A_24_P57993 | 7.31E-04 | 5.085035 | KIAA1644 | Homo sapiens KIAA1644 (KIAA1644), mRNA [NM_001099294] |
| A_21_P0005736 | 0.015893541 | 2.2607563 | lnc-PTDSS1-2 | LNCipedia lincRNA (lnc-PTDSS1-2), lincRNA [lnc-PTDSS1-2:1] |
| A_21_P0005715 | 0.0103703 | 2.0694885 | LOC101926892 | Homo sapiens uncharacterized LOC101926892 (LOC101926892), long non-coding RNA [NR_110653] |
| A_23_P722 | 0.014471924 | 2.6050386 | SYCP1 | Homo sapiens synaptonemal complex protein 1 (SYCP1), transcript variant 1, mRNA [NM_003176] |
| A_21_P0009716 | 0.01682666 | 2.9421945 | lnc-LSR-1 | LNCipedia lincRNA (lnc-LSR-1), lincRNA [lnc-LSR-1:1] |
| A_22_P00006966 | 0.019925974 | 2.1605542 | lnc-GDAP1-1 | LNCipedia lincRNA (lnc-GDAP1-1), lincRNA [lnc-GDAP1-1:1] |
| A_33_P3322383 | 0.007559916 | 3.1919436 | lnc-PAX9-2 | LNCipedia lincRNA (lnc-PAX9-2), lincRNA [lnc-PAX9-2:1] |
| A_32_P115438 | 0.007313196 | 4.07779 | LOC100128239 | Homo sapiens uncharacterized LOC100128239 (LOC100128239), long non-coding RNA [NR_027276] |
| A_22_P00023337 | 4.68E-05 | 5.3977585 |  | PREDICTED: Homo sapiens uncharacterized LOC102723446 (LOC102723446), ncRNA [XR_426177] |
| A_23_P397391 | 0.017040258 | 2.1715348 | FFAR2 | Homo sapiens free fatty acid receptor 2 (FFAR2), mRNA [NM_005306] |
| A_22_P00013655 | 0.018848084 | 2.4526944 | lnc-RP11-677O4.1.1-2 | LNCipedia lincRNA (lnc-RP11-677O4.1.1-2), lincRNA [lnc-RP11-677O4.1.1-2:2] |
| A_22_P00009823 | 4.68E-05 | 7.81589 | lnc-METAP1-3 | Homo sapiens, clone IMAGE:5165147, mRNA. [BC038532] |
| A_23_P97195 | 0.002785906 | -2.0889857 | MTFR1L | Homo sapiens mitochondrial fission regulator 1-like (MTFR1L), transcript variant 1, mRNA [NM_019557] |
| A_19_P00319509 | 0.002130372 | 2.6087635 | LINC00698 | Homo sapiens long intergenic non-protein coding RNA 698 (LINC00698), long non-coding RNA [NR_027104] |
| A_23_P345564 | 0.013260091 | 3.2360334 | OPRL1 | Homo sapiens opiate receptor-like 1 (OPRL1), transcript variant 1, mRNA [NM_182647] |
| A_21_P0009671 | 0.015057466 | 3.5084004 | lnc-ANKRD27-3 | LNCipedia lincRNA (lnc-ANKRD27-3), lincRNA [lnc-ANKRD27-3:1] |
| A_22_P00006956 | 0.0029769 | 3.1162121 | TFAP2A-AS1 | Homo sapiens TFAP2A antisense RNA 1 (TFAP2A-AS1), long non-coding RNA [NR_033910] |
| A_22_P00017899 | 0.00654853 | 2.3661778 |  | RST31110 Athersys RAGE Library Homo sapiens cDNA, mRNA sequence [BG211543] |
| A_22_P00024164 | 0.010097712 | 2.4333456 |  | Q3H5Y9_9ACTO (Q3H5Y9) Aldo/keto reductase, partial (5%) [THC2740705] |
| A_33_P3395562 | 0.009315367 | 4.0438485 | C1orf141 | Homo sapiens chromosome 1 open reading frame 141 (C1orf141), transcript variant 1, mRNA [NM_001276351] |
| A_21_P0004330 | 0.016926367 | 3.6490238 | lnc-CTB-78H18.1.1-1 | LNCipedia lincRNA (lnc-CTB-78H18.1.1-1), lincRNA [lnc-CTB-78H18.1.1-1:2] |
| A_33_P3397318 | 6.07E-04 | 2.2622514 |  | zinc finger protein 847, pseudogene [Source:HGNC Symbol;Acc:HGNC:34384] [ENST00000412638] |
| A_23_P42565 | 0.007687863 | 5.0526 | HTR5A | Homo sapiens 5-hydroxytryptamine (serotonin) receptor 5A, G protein-coupled (HTR5A), mRNA [NM_024012] |
| A_21_P0006704 | 0.006167271 | 2.3698204 |  |  |
| A_22_P00024716 | 0.004395038 | 3.3894491 |  | CR747973 NCI_CGAP_GC6 Homo sapiens cDNA clone IMAGp971B0892 ; IMAGE:2244480 5', mRNA sequence [CR747973] |
| A_21_P0014748 | 0.013374802 | -2.38059 |  |  |
| A_21_P0004961 | 0.001978732 | 3.8119066 | lnc-ARID1B-2 | LNCipedia lincRNA (lnc-ARID1B-2), lincRNA [lnc-ARID1B-2:1] |
| A_33_P3403942 | 0.016597783 | 2.2168822 | OCSTAMP | Homo sapiens osteoclast stimulatory transmembrane protein (OCSTAMP), mRNA [NM_080721] |
| A_21_P0008635 | 0.010518414 | 2.4571471 |  |  |
| A_33_P3406861 | 0.016775608 | 2.3364582 | SORBS2 | Homo sapiens sorbin and SH3 domain containing 2 (SORBS2), transcript variant 9, mRNA [NM_001270771] |
| A_22_P00007853 | 0.005949104 | 2.1104198 |  |  |
| A_22_P00012124 | 0.004017944 | 2.4636052 | lnc-POLN-1 | zl35c04.r1 Soares_pregnant_uterus_NbHPU Homo sapiens cDNA clone IMAGE:503910 5', mRNA sequence [AA131780] |
| A_23_P1904 | 0.003624071 | 4.4674187 | MS4A2 | Homo sapiens membrane-spanning 4-domains, subfamily A, member 2 (MS4A2), transcript variant 1, mRNA [NM_000139] |
| A_22_P00012978 | 0.009722025 | 2.3671148 |  | ho53b11.x1 Soares_NFL_T_GBC_S1 Homo sapiens cDNA clone IMAGE:3041085 3', mRNA sequence [BE041531] |
| A_21_P0014720 | 7.96E-04 | 2.325386 | LOC100508631 | PREDICTED: Homo sapiens uncharacterized LOC100508631 (LOC100508631), ncRNA [XR_112629] |
| A_22_P00013064 | 0.00340917 | 5.0545254 | lnc-RHD-1 | LNCipedia lincRNA (lnc-RHD-1), lincRNA [lnc-RHD-1:1] |
| A_23_P407096 | 0.010417916 | 2.8256593 | ZNF366 | Homo sapiens zinc finger protein 366 (ZNF366), mRNA [NM_152625] |
| A_21_P0009060 | 0.017540125 | 2.1247675 | LOC101928035 | Homo sapiens uncharacterized LOC101928035 (LOC101928035), long non-coding RNA [NR_104657] |
| A_24_P128727 | 0.015536374 | 4.5578494 |  | olfactory receptor, family 7, subfamily E, member 62 pseudogene [Source:HGNC Symbol;Acc:HGNC:8438] [ENST00000456741] |
| A_21_P0010793 | 0.014558029 | 2.0573401 | LOC100132287 | Homo sapiens uncharacterized LOC100132287 (LOC100132287), long non-coding RNA [NR_028322] |
| A_19_P00319963 | 0.016775608 | 2.7215044 | LOC101929683 | PREDICTED: Homo sapiens uncharacterized LOC101929683 (LOC101929683), ncRNA [XR_242023] |
| A_21_P0000710 | 0.002895626 | 4.8760557 | HMGA1P7 | Homo sapiens high mobility group AT-hook 1 pseudogene 7 (HMGA1P7), non-coding RNA [NR_037938] |
| A_33_P3788772 | 0.004017944 | 4.703628 | LOC200609 | Homo sapiens mRNA; cDNA DKFZp434E2221 (from clone DKFZp434E2221) [AL137458] |
| A_21_P0007326 | 0.016494371 | 5.251729 | lnc-FANCF-1 | LNCipedia lincRNA (lnc-FANCF-1), lincRNA [lnc-FANCF-1:2] |
| A_23_P144896 | 0.016420968 | -2.370794 | PDLIM7 | Homo sapiens PDZ and LIM domain 7 (enigma) (PDLIM7), transcript variant 1, mRNA [NM_005451] |
| A_21_P0001243 | 0.006177985 | 2.2295423 |  | Homo sapiens cDNA FLJ45859 fis, clone OCBBF2032539. [AK127758] |
| A_33_P3264895 | 0.007794031 | 2.1371667 | RHEBL1 | Homo sapiens Ras homolog enriched in brain like 1 (RHEBL1), transcript variant 2, mRNA [NM_001303126] |
| A_22_P00007837 | 2.27E-04 | 3.7742393 | lnc-HPS4-1 | LNCipedia lincRNA (lnc-HPS4-1), lincRNA [lnc-HPS4-1:1] |
| A_22_P00019569 | 2.62E-04 | 2.4992654 | C8orf87 | Homo sapiens chromosome 8 open reading frame 87 (C8orf87), mRNA [NM_001242668] |
| A_21_P0002425 | 4.27E-04 | 2.7635808 | lnc-AC013480.1.1-4 | Q221K3_9BURK (Q221K3) Flagellar hook-basal body complex protein, partial (12%) [THC2775122] |
| A_22_P00009142 | 0.004017944 | 2.3839762 | SNHG22 | Homo sapiens small nucleolar RNA host gene 22 (non-protein coding) (SNHG22), long non-coding RNA [NR_117096] |
| A_21_P0009905 | 0.001486759 | 5.6445146 | lnc-PREX1-6 | LNCipedia lincRNA (lnc-PREX1-6), lincRNA [lnc-PREX1-6:2] |
| A_19_P00321223 | 0.018736346 | 2.4515247 |  |  |
| A_22_P00012603 | 0.002822993 | 2.7299528 |  |  |
| A_21_P0010099 | 0.004264157 | 2.4329588 | lnc-SLC17A9-2 | LNCipedia lincRNA (lnc-SLC17A9-2), lincRNA [lnc-SLC17A9-2:1] |
| A_21_P0001135 | 0.015542324 | 3.4000368 |  |  |
| A_22_P00018081 | 7.61E-05 | 3.3534791 | LOC101929464 | Homo sapiens uncharacterized LOC101929464 (LOC101929464), long non-coding RNA [NR_125978] |
| A_21_P0009478 | 0.014148441 | 5.8235707 | lnc-DCC-2 | LNCipedia lincRNA (lnc-DCC-2), lincRNA [lnc-DCC-2:1] |
| A_22_P00014369 | 0.002822993 | 7.764116 | LOC101927580 | Homo sapiens uncharacterized LOC101927580 (LOC101927580), long non-coding RNA [NR_110917] |
| A_33_P3369063 | 6.83E-04 | 10.038211 |  | olfactory receptor, family 5, subfamily BB, member 1 pseudogene [Source:HGNC Symbol;Acc:HGNC:15271] [ENST00000528843] |
| A_21_P0006555 | 0.018245582 | 2.969999 | lnc-ASMT-2 | LNCipedia lincRNA (lnc-ASMT-2), lincRNA [lnc-ASMT-2:1] |
| A_21_P0003642 | 0.004754042 | 2.2528698 | lnc-SYNPO2-2 | LNCipedia lincRNA (lnc-SYNPO2-2), lincRNA [lnc-SYNPO2-2:1] |
| A_23_P165180 | 0.004228714 | -2.6731994 | RFXANK | Homo sapiens regulatory factor X-associated ankyrin-containing protein (RFXANK), transcript variant 1, mRNA [NM_003721] |
| A_21_P0008083 | 0.011892423 | 2.5452583 | lnc-UCHL3-5 | LNCipedia lincRNA (lnc-UCHL3-5), lincRNA [lnc-UCHL3-5:1] |
| A_23_P162386 | 0.013079802 | 2.9340432 | BIN2 | Homo sapiens bridging integrator 2 (BIN2), transcript variant 1, mRNA [NM_016293] |
| A_21_P0002648 | 0.018740008 | 2.868777 | LOC102724948 | PREDICTED: Homo sapiens uncharacterized LOC102724948 (LOC102724948), ncRNA [XR_425373] |
| A_21_P0007628 | 0.002278488 | 2.0168073 | lnc-SDSL-5 | LNCipedia lincRNA (lnc-SDSL-5), lincRNA [lnc-SDSL-5:1] |
| A_23_P32165 | 0.012069974 | 3.369726 | LHX2 | Homo sapiens LIM homeobox 2 (LHX2), mRNA [NM_004789] |
| A_23_P71757 | 0.003721795 | 5.0725627 | IFNA6 | Homo sapiens interferon, alpha 6 (IFNA6), mRNA [NM_021002] |
| A_22_P00019442 | 0.01865728 | 2.7582023 |  | ALU1_HUMAN (P39188) Alu subfamily J sequence contamination warning entry, partial (5%) [THC2696963] |
| A_22_P00012626 | 0.017243527 | 2.1912231 | LOC101928823 | Homo sapiens uncharacterized LOC101928823 (LOC101928823), transcript variant 2, long non-coding RNA [NR_120560] |
| A_22_P00001140 | 0.004047977 | 3.2301683 | lnc-AMDHD1-2 | LNCipedia lincRNA (lnc-AMDHD1-2), lincRNA [lnc-AMDHD1-2:1] |
| A_21_P0014049 | 0.01055442 | 2.4984882 | LOC389834 | Homo sapiens ankyrin repeat domain 57 pseudogene (LOC389834), non-coding RNA [NR_027420] |
| A_23_P127579 | 0.015778573 | -2.4913297 | PTS | Homo sapiens 6-pyruvoyltetrahydropterin synthase (PTS), mRNA [NM_000317] |
| A_33_P3279158 | 0.006028791 | 3.485103 | NBPF6 | Homo sapiens neuroblastoma breakpoint family, member 6 (NBPF6), transcript variant 2, mRNA [NM_001143988] |
| A_22_P00018519 | 0.013569502 | 2.3950446 | lnc-MIS18A-3 | EST6308 human nasopharynx Homo sapiens cDNA, mRNA sequence [CD689785] |
| A_21_P0010436 | 0.003170984 | 2.3789306 | lnc-LARGE-2 | LNCipedia lincRNA (lnc-LARGE-2), lincRNA [lnc-LARGE-2:2] |
| A_21_P0013614 | 0.001425761 | 4.3137183 | XLOC_l2_015098 | BROAD Institute lincRNA (XLOC_l2_015098), lincRNA [TCONS_l2_00029169] |
| A_33_P3405369 | 7.57E-04 | 2.6978638 | KRTAP5-4 | Homo sapiens keratin associated protein 5-4 (KRTAP5-4), mRNA [NM_001012709] |
| A_21_P0005543 | 0.003956788 | 2.611561 | lnc-XRCC2-2 | LNCipedia lincRNA (lnc-XRCC2-2), lincRNA [lnc-XRCC2-2:1] |
| A_22_P00013409 | 0.011419726 | 2.6084032 |  |  |
| A_21_P0006836 | 0.019850597 | 2.6685717 | lnc-MCM10-1 | LNCipedia lincRNA (lnc-MCM10-1), lincRNA [lnc-MCM10-1:1] |
| A_33_P3369956 | 0.011370456 | 2.0049515 | ATXN2L | Homo sapiens ataxin 2-like (ATXN2L), transcript variant E, mRNA [NM_148416] |
| A_33_P3236833 | 0.015893705 | 4.79023 | GPR12 | Homo sapiens G protein-coupled receptor 12 (GPR12), mRNA [NM_005288] |
| A_22_P00013007 | 0.011022793 | 3.1891322 | lnc-RGL4-2 | DB088362 TESTI4 Homo sapiens cDNA clone TESTI4036562 5', mRNA sequence [DB088362] |
| A_22_P00025341 | 2.66E-04 | 6.237757 | lnc-MAPK6-6 | LNCipedia lincRNA (lnc-MAPK6-6), lincRNA [lnc-MAPK6-6:2] |
| A_22_P00018622 | 0.007881575 | 2.1986942 | lnc-LRRC53-1 | RST2286 Athersys RAGE Library Homo sapiens cDNA, mRNA sequence [BG183392] |
| A_21_P0006471 | 0.015286709 | 5.1799207 |  |  |
| A_33_P3418686 | 0.005600513 | 3.9673066 | HCN4 | Homo sapiens hyperpolarization activated cyclic nucleotide gated potassium channel 4 (HCN4), mRNA [NM_005477] |
| A_23_P317082 | 0.00384717 | 4.229 | SYTL5 | Homo sapiens synaptotagmin-like 5 (SYTL5), transcript variant 1, mRNA [NM_138780] |
| A_21_P0004079 | 0.012577059 | 2.01069 | lnc-FAM153A-2 | LNCipedia lincRNA (lnc-FAM153A-2), lincRNA [lnc-FAM153A-2:1] |
| A_21_P0005800 | 0.006488535 | 4.495322 | lnc-RP1L1-1 | LNCipedia lincRNA (lnc-RP1L1-1), lincRNA [lnc-RP1L1-1:1] |
| A_21_P0004823 | 0.019903338 | 3.036319 | lnc-BPHL-2 | LNCipedia lincRNA (lnc-BPHL-2), lincRNA [lnc-BPHL-2:2] |
| A_22_P00019627 | 0.007794031 | 2.8687131 | lnc-ALCAM-2 | Homo sapiens cDNA, FLJ98989. [AK308948] |
| A_24_P183264 | 0.003916526 | -2.0388725 | BTF3 | Homo sapiens basic transcription factor 3 (BTF3), transcript variant 1, mRNA [NM_001037637] |
| A_22_P00010047 | 0.007881575 | 2.1427069 | lnc-MOCS2-1 | DA240949 BRAWH3 Homo sapiens cDNA clone BRAWH3038621 5', mRNA sequence [DA240949] |
| A_23_P114353 | 0.005492279 | 3.5407097 | NXF5 | Homo sapiens nuclear RNA export factor 5 (NXF5), transcript variant 1, mRNA [NM_032946] |
| A_33_P3268622 | 0.016285634 | 2.1409502 | LY6D | Homo sapiens lymphocyte antigen 6 complex, locus D (LY6D), mRNA [NM_003695] |
| A_22_P00003289 | 8.79E-06 | 6.0127616 |  | BX091468 Soares_testis_NHT Homo sapiens cDNA clone IMAGp998A141864 ; IMAGE:757909, mRNA sequence [BX091468] |
| A_22_P00022593 | 7.60E-05 | 3.9493873 |  |  |
| A_22_P00022094 | 0.010054196 | 3.4247725 | lnc-LRTM2-1 | LNCipedia lincRNA (lnc-LRTM2-1), lincRNA [lnc-LRTM2-1:1] |
| A_22_P00014860 | 0.01375931 | 2.1539924 |  |  |
| A_21_P0002223 | 0.017542422 | 2.960975 | LINC01122 | Homo sapiens long intergenic non-protein coding RNA 1122 (LINC01122), long non-coding RNA [NR_033873] |
| A_21_P0001830 | 0.003579142 | 5.530267 | MYCNUT | Homo sapiens MYCN upstream transcript (non-protein coding) (MYCNUT), long non-coding RNA [NR_125783] |
| A_23_P46351 | 0.019543365 | 3.6584275 | TDRKH | Homo sapiens tudor and KH domain containing (TDRKH), transcript variant 3, mRNA [NM_006862] |
| A_21_P0008253 | 1.94E-04 | 6.7112904 | lnc-TSC22D1-1 | LNCipedia lincRNA (lnc-TSC22D1-1), lincRNA [lnc-TSC22D1-1:5] |
| A_22_P00007467 | 0.014384066 | 4.537278 |  |  |
| A_33_P3283460 | 0.015756283 | 2.1116931 | MIER1 | Homo sapiens mesoderm induction early response 1, transcriptional regulator (MIER1), transcript variant 11, mRNA [NM_001278215] |
| A_21_P0009494 | 0.005339755 | 3.6161332 | lnc-SOCS6-2 | LNCipedia lincRNA (lnc-SOCS6-2), lincRNA [lnc-SOCS6-2:1] |
| A_23_P121665 | 0.01226622 | -2.1139839 | SORCS2 | Homo sapiens sortilin-related VPS10 domain containing receptor 2 (SORCS2), mRNA [NM_020777] |
| A_33_P3212052 | 0.007304631 | 3.1470912 |  |  |
| A_22_P00018557 | 0.003967373 | 4.103369 |  | DB100140 TESTI4 Homo sapiens cDNA clone TESTI4051979 5', mRNA sequence [DB100140] |
| A_21_P0011050 | 0.009920881 | 3.913911 | XLOC_l2_003052 | BROAD Institute lincRNA (XLOC_l2_003052), lincRNA [TCONS_l2_00005763] |
| A_21_P0010258 | 0.002092737 | 2.8754628 | lnc-CRYAA-1 | LNCipedia lincRNA (lnc-CRYAA-1), lincRNA [lnc-CRYAA-1:1] |
| A_32_P458472 | 0.018414054 | 2.7097409 | C1orf95 | Homo sapiens chromosome 1 open reading frame 95 (C1orf95), mRNA [NM_001003665] |
| A_33_P3318243 | 0.001872603 | 3.8655782 |  | Q75NF4_PROFR (Q75NF4) Bifunctional cobalamin biosynthesis protein, partial (6%) [THC2677277] |
| A_21_P0000546 | 0.011555376 | 2.464613 | DPPA2P3 | Homo sapiens developmental pluripotency associated 2 pseudogene 3 (DPPA2P3), non-coding RNA [NR_027764] |
| A_33_P3319845 | 0.008065707 | 2.9089327 | OR51B6 | Homo sapiens olfactory receptor, family 51, subfamily B, member 6 (OR51B6), mRNA [NM_001004750] |
| A_21_P0011507 | 2.45E-04 | 2.0594835 | XLOC_l2_005602 | BROAD Institute lincRNA (XLOC_l2_005602), lincRNA [TCONS_l2_00010489] |
| A_22_P00000636 | 0.018486913 | 3.6143305 |  |  |
| A_21_P0006980 | 0.015434226 | 3.5868275 | lnc-MPP7-1 | LNCipedia lincRNA (lnc-MPP7-1), lincRNA [lnc-MPP7-1:1] |
| A_21_P0010915 | 0.003888986 | 2.7357044 |  |  |
| A_21_P0006976 | 0.001309748 | 2.5639467 | LOC101929117 | PREDICTED: Homo sapiens uncharacterized LOC101929117 (LOC101929117), transcript variant X1, ncRNA [XR_242740] |
| A_22_P00017626 | 0.012082747 | 4.1292577 |  |  |
| A_24_P922849 | 0.006028791 | 3.4691303 | ANHX | Homo sapiens anomalous homeobox (ANHX), mRNA [NM_001191054] |
| A_24_P772436 | 0.005637546 | 2.7385545 | DCTN1-AS1 | Homo sapiens DCTN1 antisense RNA 1 (DCTN1-AS1), long non-coding RNA [NR_024463] |
| A_33_P3358957 | 0.014760993 | 2.2594135 | PAPL | Homo sapiens iron/zinc purple acid phosphatase-like protein (PAPL), mRNA [NM_001004318] |
| A_22_P00021163 | 0.009587442 | 2.5304239 |  |  |
| A_22_P00013315 | 0.001167695 | 3.3632717 |  | ov36f06.x1 Soares_testis_NHT Homo sapiens cDNA clone IMAGE:1639427 3', mRNA sequence [AI025159] |
| A_22_P00016997 | 0.01590558 | 2.6758945 | DSCR9 | Homo sapiens Down syndrome critical region 9 (non-protein coding) (DSCR9), long non-coding RNA [NR_026719] |
| A_21_P0004694 | 0.013190938 | 2.86545 |  |  |
| A_24_P111054 | 8.61E-04 | 5.3523216 | SLC2A5 | solute carrier family 2 (facilitated glucose/fructose transporter), member 5 [Source:HGNC Symbol;Acc:HGNC:11010] [ENST00000377414] |
| A_22_P00022613 | 0.015092672 | 5.5648394 | LINC01007 | Homo sapiens long intergenic non-protein coding RNA 1007 (LINC01007), transcript variant 3, long non-coding RNA [NR_103749] |
| A_21_P0001051 | 0.015303658 | 3.5366325 |  | QV2-BT0685-010700-259-g10 BT0685 Homo sapiens cDNA, mRNA sequence [BE694275] |
| A_21_P0005587 | 2.18E-04 | 2.402091 | lnc-AC021218.2.1-2 | LNCipedia lincRNA (lnc-AC021218.2.1-2), lincRNA [lnc-AC021218.2.1-2:1] |
| A_24_P395814 | 0.017797079 | 2.2842271 | CGB | Homo sapiens chorionic gonadotropin, beta polypeptide (CGB), mRNA [NM_000737] |
| A_23_P162010 | 0.004017944 | 4.4399896 | CCKBR | Homo sapiens cholecystokinin B receptor (CCKBR), mRNA [NM_176875] |
| A_33_P3333542 | 0.018877055 | 2.2453666 |  | Homo sapiens clone pp8142 unknown mRNA. [AF289601] |
| A_22_P00007762 | 0.006144255 | 2.950654 | lnc-HMOX1-1 | LNCipedia lincRNA (lnc-HMOX1-1), lincRNA [lnc-HMOX1-1:1] |
| A_22_P00002102 | 3.91E-04 | 4.563687 | LOC101929445 | PREDICTED: Homo sapiens uncharacterized LOC101929445 (LOC101929445), ncRNA [XR_246525] |
| A_21_P0006293 | 0.007825878 | 2.4767444 |  | PREDICTED: Homo sapiens uncharacterized LOC100507103 (LOC100507103), ncRNA [XR_108974] |
| A_23_P83184 | 0.014358267 | 3.1439922 | LCN6 | Homo sapiens lipocalin 6 (LCN6), mRNA [NM_198946] |
| A_24_P391960 | 0.0192649 | -2.0631483 |  | eukaryotic translation initiation factor 1 pseudogene 3 [Source:HGNC Symbol;Acc:HGNC:49616] [ENST00000423841] |
| A_23_P321496 | 0.017750079 | 2.468117 | ANKRD30BP2 | Homo sapiens ankyrin repeat domain 30B pseudogene 2 (ANKRD30BP2), non-coding RNA [NR_026916] |
| A_33_P3350863 | 0.009028136 | 2.0534213 | RETN | Homo sapiens resistin (RETN), transcript variant 1, mRNA [NM_020415] |
| A_22_P00011456 | 0.001645564 | 6.266597 |  |  |
| A_21_P0007996 | 0.007796347 | -3.1330576 | lnc-LECT1-1 | LNCipedia lincRNA (lnc-LECT1-1), lincRNA [lnc-LECT1-1:1] |
| A_33_P3386344 | 0.007796347 | 2.8364818 | FANCA | Homo sapiens Fanconi anemia, complementation group A (FANCA), transcript variant 2, mRNA [NM_001018112] |
| A_22_P00012274 | 4.85E-04 | 4.1823916 |  | Homo sapiens cDNA clone IMAGE:4829680. [BC042560] |
| A_21_P0012793 | 0.005789592 | 4.845763 | LOC101927282 |  |
| A_23_P250462 | 0.016979946 | -2.2374299 | ATP6AP1 | Homo sapiens ATPase, H+ transporting, lysosomal accessory protein 1 (ATP6AP1), mRNA [NM_001183] |
| A_32_P10272 | 0.015872229 | -2.0557652 | PMS2P5 | Homo sapiens postmeiotic segregation increased 2 pseudogene 5 (PMS2P5), transcript variant 1, non-coding RNA [NR_027775] |
| A_33_P3381067 | 0.001854862 | 4.609297 | OR10Q1 | Homo sapiens olfactory receptor, family 10, subfamily Q, member 1 (OR10Q1), mRNA [NM_001004471] |
| A_21_P0013207 | 7.24E-05 | 5.0759654 | XLOC_l2_013434 | BROAD Institute lincRNA (XLOC_l2_013434), lincRNA [TCONS_l2_00025918] |
| A_21_P0003657 | 0.01439562 | 2.5304208 | lnc-MGST2-1 | LNCipedia lincRNA (lnc-MGST2-1), lincRNA [lnc-MGST2-1:1] |
| A_24_P540057 | 0.011555376 | 2.5104 | CA5A | Homo sapiens carbonic anhydrase VA, mitochondrial (CA5A), mRNA [NM_001739] |
| A_22_P00017272 | 0.018736346 | 4.018508 |  |  |
| A_33_P3408852 | 2.27E-04 | 3.391925 | EPHA8 | Homo sapiens EPH receptor A8 (EPHA8), transcript variant 2, mRNA [NM_001006943] |
| A_24_P816384 | 9.73E-04 | -2.366982 | UBE2Q2P1 | Homo sapiens ubiquitin-conjugating enzyme E2Q family member 2 pseudogene 1 (UBE2Q2P1), non-coding RNA [NR_003661] |
| A_21_P0002919 | 0.001162504 | 5.8918214 |  |  |
| A_21_P0008671 | 0.012613835 | 2.2234828 | lnc-FANCI-3 | LNCipedia lincRNA (lnc-FANCI-3), lincRNA [lnc-FANCI-3:2] |
| A_22_P00015240 | 0.018005375 | 3.9083364 |  |  |
| A_21_P0013715 | 0.012055238 | 3.0921323 |  | coactivator-associated arginine methyltransferase 1 pseudogene 1 [Source:HGNC Symbol;Acc:HGNC:23392] [ENST00000515723] |
| A_23_P160318 | 0.016926367 | -2.950788 | COL16A1 | Homo sapiens collagen, type XVI, alpha 1 (COL16A1), mRNA [NM_001856] |
| A_22_P00013395 | 0.003922251 | 2.586598 | LOC643711 | Homo sapiens platelet-activating factor acetylhydrolase 1b, catalytic subunit 2 (30kDa) pseudogene (LOC643711), transcript variant 2, non-coding RNA [NR_077241] |
| A_23_P74701 | 0.001890924 | 2.0063612 | COL24A1 | Homo sapiens collagen, type XXIV, alpha 1 (COL24A1), mRNA [NM_152890] |
| A_24_P313186 | 0.012930914 | -2.2546995 | CALM1 | Homo sapiens calmodulin 1 (phosphorylase kinase, delta) (CALM1), mRNA [NM_006888] |
| A_33_P3271196 | 0.01477626 | -3.0055392 | AMOTL1 | Homo sapiens angiomotin like 1 (AMOTL1), transcript variant 1, mRNA [NM_130847] |
| A_21_P0004941 | 0.011216838 | 2.4316425 | LINC01013 | Homo sapiens long intergenic non-protein coding RNA 1013 (LINC01013), long non-coding RNA [NR_038981] |
| A_22_P00009031 | 0.011101848 | 2.5518765 |  | 601341849F1 NIH_MGC_53 Homo sapiens cDNA clone IMAGE:3683988 5', mRNA sequence [BE568288] |
| A_23_P102706 | 0.019543365 | 2.130711 | SNPH | Homo sapiens syntaphilin (SNPH), mRNA [NM_014723] |
| A_21_P0008734 | 7.03E-04 | 5.5395207 | lnc-MYEF2-5 | LNCipedia lincRNA (lnc-MYEF2-5), lincRNA [lnc-MYEF2-5:1] |
| A_22_P00004604 | 0.013260091 | 2.429139 | lnc-CSMD1-8 | LNCipedia lincRNA (lnc-CSMD1-8), lincRNA [lnc-CSMD1-8:1] |
| A_33_P3317770 | 0.007796525 | 2.2593396 | DCST1 | DC-STAMP domain containing 1 [Source:HGNC Symbol;Acc:HGNC:26539] [ENST00000368420] |
| A_22_P00016202 | 0.019957 | 5.1310077 | LBX1-AS1 | Homo sapiens LBX1 antisense RNA 1 (head to head) (LBX1-AS1), long non-coding RNA [NR_029380] |
| A_21_P0008122 | 0.011724273 | 2.3047671 | LINC00354 | Homo sapiens long intergenic non-protein coding RNA 354 (LINC00354), transcript variant 1, long non-coding RNA [NR_120401] |
| A_21_P0010900 | 0.001068657 | 2.0131576 | XLOC_l2_002100 | BROAD Institute lincRNA (XLOC_l2_002100), lincRNA [TCONS_l2_00003754] |
| A_22_P00007107 | 0.004017944 | 2.3103087 | LOC101927735 | Homo sapiens uncharacterized LOC101927735 (LOC101927735), long non-coding RNA [NR_110058] |
| A_21_P0000530 | 0.005823871 | -2.566648 | LRRC75A-AS1 | Homo sapiens LRRC75A antisense RNA 1 (LRRC75A-AS1), transcript variant 27, long non-coding RNA [NR_045024] |
| A_21_P0002064 | 0.003198486 | 3.143662 |  |  |
| A_21_P0010106 | 0.016114652 | 5.420399 | lnc-GPCPD1-1 | LNCipedia lincRNA (lnc-GPCPD1-1), lincRNA [lnc-GPCPD1-1:4] |
| A_21_P0008924 | 0.007681231 | 2.5012228 | lnc-CTD-2144E22.5.1-4 | LNCipedia lincRNA (lnc-CTD-2144E22.5.1-4), lincRNA [lnc-CTD-2144E22.5.1-4:1] |
| A_33_P3313552 | 0.001192546 | 3.6834593 |  |  |
| A_33_P3355732 | 0.015752807 | 2.159694 | UMODL1 | uromodulin-like 1 [Source:HGNC Symbol;Acc:HGNC:12560] [ENST00000491559] |
| A_33_P3403643 | 0.01569922 | 2.6850042 | LOC101059906 | PREDICTED: Homo sapiens collagen alpha-2(VI) chain-like (LOC101059906), mRNA [XM_003960862] |
| A_21_P0001258 | 0.01569922 | 3.1497705 |  |  |
| A_24_P196117 | 0.010452952 | -2.4823458 | GTF2H5 | Homo sapiens general transcription factor IIH, polypeptide 5 (GTF2H5), mRNA [NM_207118] |
| A_21_P0002410 | 0.003501274 | 3.5141716 | lnc-HS1BP3-1 | LNCipedia lincRNA (lnc-HS1BP3-1), lincRNA [lnc-HS1BP3-1:1] |
| A_21_P0009816 | 0.006252873 | 2.116396 | lnc-FOXA2-8 | Homo sapiens mRNA; cDNA DKFZp781N2119 (from clone DKFZp781N2119). [CR627206] |
| A_21_P0005008 | 0.005484189 | 2.271349 | lnc-MBOAT1-3 | LNCipedia lincRNA (lnc-MBOAT1-3), lincRNA [lnc-MBOAT1-3:4] |
| A_21_P0002033 | 6.49E-04 | 7.008596 |  |  |
| A_33_P3274811 | 0.012049676 | 2.4076085 | SPRR2F | Homo sapiens small proline-rich protein 2F (SPRR2F), mRNA [NM_001014450] |
| A_21_P0001059 | 0.015645228 | 3.1432283 | LOC101929565 | Homo sapiens uncharacterized LOC101929565 (LOC101929565), long non-coding RNA [NR_125985] |
| A_24_P271830 | 4.27E-04 | 4.8380303 | LINC00670 | Homo sapiens long intergenic non-protein coding RNA 670 (LINC00670), transcript variant 1, long non-coding RNA [NR_034145] |
| A_21_P0005009 | 2.66E-04 | 3.9238997 | lnc-MBOAT1-3 | LNCipedia lincRNA (lnc-MBOAT1-3), lincRNA [lnc-MBOAT1-3:7] |
| A_21_P0010253 | 2.66E-04 | 3.3268003 | lnc-UMODL1-1 | LNCipedia lincRNA (lnc-UMODL1-1), lincRNA [lnc-UMODL1-1:1] |
| A_33_P3241786 | 0.016327148 | 3.6259887 | ADD2 | Homo sapiens adducin 2 (beta) (ADD2), transcript variant 2, mRNA [NM_017482] |
| A_22_P00003403 | 6.63E-04 | 2.7640545 | LOC101928885 | PREDICTED: Homo sapiens uncharacterized LOC101928885 (RP11-141O11.2), transcript variant X2, ncRNA [XR_241812] |
| A_21_P0002644 | 0.01693474 | 2.14153 | lnc-CMPK2-13 | LNCipedia lincRNA (lnc-CMPK2-13), lincRNA [lnc-CMPK2-13:6] |
| A_22_P00002087 | 0.017647024 | 4.517281 | lnc-BMP6-10 | LNCipedia lincRNA (lnc-BMP6-10), lincRNA [lnc-BMP6-10:2] |
| A_21_P0002273 | 0.005164471 | 6.277555 | lnc-DDX18-2 | LNCipedia lincRNA (lnc-DDX18-2), lincRNA [lnc-DDX18-2:1] |
| A_33_P3419632 | 0.005503832 | 3.2915132 | GLB1L3 | galactosidase, beta 1-like 3 [Source:HGNC Symbol;Acc:HGNC:25147] [ENST00000389887] |
| A_21_P0012527 | 0.018820966 | 3.8172865 |  |  |
| A_33_P3218491 | 7.75E-06 | 2.440728 |  |  |
| A_22_P00000878 | 0.00635109 | 2.7043564 |  |  |
| A_33_P3489228 | 0.010805532 | -4.01178 | PTPN20B | Homo sapiens protein tyrosine phosphatase, non-receptor type 20B (PTPN20B), transcript variant 2, mRNA [NM_015605] |
| A_21_P0010320 | 0.01693474 | 2.5319507 | lnc-C21orf54-3 | LNCipedia lincRNA (lnc-C21orf54-3), lincRNA [lnc-C21orf54-3:1] |
| A_32_P228268 | 0.012357703 | 2.3576236 | DNAH10 | Homo sapiens dynein, axonemal, heavy chain 10 (DNAH10), mRNA [NM_207437] |
| A_21_P0007830 | 0.006432454 | 4.673917 | MUC19 | Homo sapiens mucin 19, oligomeric (MUC19), mRNA [NM_173600] |
| A_22_P00011100 | 0.005720803 | 3.656725 |  |  |
| A_19_P00319276 | 0.007882567 | 2.3112295 | lnc-BMP7-2 | LNCipedia lincRNA (lnc-BMP7-2), lincRNA [lnc-BMP7-2:2] |
| A_32_P157208 | 0.017540125 | 2.7322173 | PGM5P3-AS1 | Homo sapiens PGM5P3 antisense RNA 1 (PGM5P3-AS1), transcript variant 1, long non-coding RNA [NR_121188] |
| A_33_P3362652 | 0.010407414 | 6.881017 | OR5H14 | Homo sapiens olfactory receptor, family 5, subfamily H, member 14 (OR5H14), mRNA [NM_001005514] |
| A_21_P0012663 | 8.68E-04 | 4.791871 | XLOC_l2_011102 | BROAD Institute lincRNA (XLOC_l2_011102), lincRNA [TCONS_l2_00021110] |
| A_32_P353072 | 0.015568356 | -2.481514 | TMEM106B | Homo sapiens transmembrane protein 106B (TMEM106B), transcript variant 1, mRNA [NM_018374] |
| A_23_P18342 | 0.005823871 | 2.5062745 | EPHA6 | Homo sapiens EPH receptor A6 (EPHA6), transcript variant 3, mRNA [NM_001278300] |
| A_22_P00020735 | 8.61E-04 | 3.3416185 |  | PREDICTED: Homo sapiens uncharacterized LOC101927496 (LOC101927496), ncRNA [XR_243856] |
| A_23_P164451 | 0.014765953 | -2.300741 | TBX2 | Homo sapiens T-box 2 (TBX2), mRNA [NM_005994] |
| A_23_P217946 | 0.012143905 | 2.2311473 | CDH23 | Homo sapiens cadherin-related 23 (CDH23), transcript variant 1, mRNA [NM_022124] |
| A_33_P3266783 | 0.019925974 | 2.2718906 |  | Q66I89_BRARE (Q66I89) PQ loop repeat containing 3, partial (6%) [THC2736258] |
| A_22_P00002383 | 0.014990651 | 2.2897553 | lnc-C13orf15-1 | LNCipedia lincRNA (lnc-C13orf15-1), lincRNA [lnc-C13orf15-1:1] |
| A_23_P8196 | 0.005862902 | 2.569397 | ME1 | Homo sapiens malic enzyme 1, NADP(+)-dependent, cytosolic (ME1), mRNA [NM_002395] |
| A_22_P00018976 | 0.01569922 | 5.7314606 |  |  |
| A_21_P0008065 | 0.014969504 | 2.1458592 | lnc-OLFM4-1 | LNCipedia lincRNA (lnc-OLFM4-1), lincRNA [lnc-OLFM4-1:1] |
| A_33_P3384617 | 0.002384241 | 2.042508 | XLOC_l2_003882 | BROAD Institute lincRNA (XLOC_l2_003882), lincRNA [TCONS_l2_00007046] |
| A_23_P207345 | 0.0192649 | 2.2544682 | ADAM11 | Homo sapiens ADAM metallopeptidase domain 11 (ADAM11), mRNA [NM_002390] |
| A_22_P00021819 | 0.007446792 | 4.8027716 |  |  |
| A_22_P00001356 | 0.002277691 | 5.48787 |  | DB148555 THYMU3 Homo sapiens cDNA clone THYMU3024557 5', mRNA sequence [DB148555] |
| A_21_P0003603 | 0.014822709 | 3.9257147 | lnc-KLF3-2 | LNCipedia lincRNA (lnc-KLF3-2), lincRNA [lnc-KLF3-2:1] |
| A_24_P29445 | 0.014524619 | -2.472233 | TMEM14B | Homo sapiens transmembrane protein 14B (TMEM14B), transcript variant 1, mRNA [NM_030969] |
| A_33_P3315325 | 0.004264157 | 2.6284642 | ANKRD46 | Homo sapiens ankyrin repeat domain 46 (ANKRD46), transcript variant 4, mRNA [NM_001270379] |
| A_21_P0012026 | 0.002130372 | 2.5260866 | TEX41 | testis expressed 41 (non-protein coding) [Source:HGNC Symbol;Acc:HGNC:48667] [ENST00000432608] |
| A_22_P00022486 | 0.008002416 | -2.4165826 | DNAJC9 | DnaJ (Hsp40) homolog, subfamily C, member 9 [Source:HGNC Symbol;Acc:HGNC:19123] [ENST00000512551] |
| A_23_P70468 | 8.79E-06 | 8.944703 | OR2B6 | Homo sapiens olfactory receptor, family 2, subfamily B, member 6 (OR2B6), mRNA [NM_012367] |
| A_21_P0002610 | 0.016433833 | 3.6865194 | lnc-PLEKHB2-1 | LNCipedia lincRNA (lnc-PLEKHB2-1), lincRNA [lnc-PLEKHB2-1:5] |
| A_22_P00013493 | 0.002705118 | 3.1171877 | LOC102723481 | PREDICTED: Homo sapiens uncharacterized LOC102723481 (LOC102723481), ncRNA [XR_424610] |
| A_33_P3382714 | 0.019137397 | 4.319087 | LINC00934 | Homo sapiens long intergenic non-protein coding RNA 934 (LINC00934), long non-coding RNA [NR_024246] |
| A_32_P129120 | 0.003409261 | 4.4287615 | FAM182A | Homo sapiens family with sequence similarity 182, member A (FAM182A), long non-coding RNA [NR_026713] |
| A_21_P0011965 | 0.014342867 | 3.6651008 |  | fatty acyl CoA reductase 2 pseudogene 1 [Source:HGNC Symbol;Acc:HGNC:49284] [ENST00000444030] |
| A_21_P0008339 | 0.01037101 | 2.4499538 | lnc-PELI2-1 | LNCipedia lincRNA (lnc-PELI2-1), lincRNA [lnc-PELI2-1:1] |
| A_33_P3214334 | 7.82E-04 | 4.0409546 | LY6G6F | Homo sapiens lymphocyte antigen 6 complex, locus G6F (LY6G6F), mRNA [NM_001003693] |
| A_23_P111860 | 0.016285634 | -2.5285444 | RADIL | Homo sapiens Ras association and DIL domains (RADIL), mRNA [NM_018059] |
| A_22_P00005616 | 0.015542324 | 2.3068943 | LIFR-AS1 | Homo sapiens LIFR antisense RNA 1 (LIFR-AS1), transcript variant 2, long non-coding RNA [NR_103554] |
| A_22_P00024412 | 0.006028791 | 3.7394009 | lnc-EHD3-3 | AGENCOURT_10402298 NIH_MGC_82 Homo sapiens cDNA clone IMAGE:6615934 5', mRNA sequence [BU568718] |
| A_22_P00025345 | 0.013718982 | 4.217216 | CASC6 | Homo sapiens cancer susceptibility candidate 6 (non-protein coding) (CASC6), long non-coding RNA [NR_104154] |
| A_23_P42241 | 0.002884686 | 5.588604 | OR5V1 | Homo sapiens olfactory receptor, family 5, subfamily V, member 1 (OR5V1), mRNA [NM_030876] |
| A_33_P6473819 | 9.49E-04 | 5.1121273 | LINC00382 | Homo sapiens long intergenic non-protein coding RNA 382 (LINC00382), long non-coding RNA [NR_120413] |
| A_33_P3345821 | 0.005392477 | 5.8897505 |  |  |
| A_33_P3284212 | 0.005183944 | 5.052825 | TPD52L3 | Homo sapiens tumor protein D52-like 3 (TPD52L3), transcript variant 2, mRNA [NM_001001874] |
| A_22_P00010742 | 3.38E-04 | 2.8485148 | lnc-NIPAL4-1 | LNCipedia lincRNA (lnc-NIPAL4-1), lincRNA [lnc-NIPAL4-1:1] |
| A_33_P3385656 | 0.018319929 | 2.7880592 | FNDC9 | Homo sapiens fibronectin type III domain containing 9 (FNDC9), mRNA [NM_001001343] |
| A_22_P00024664 | 0.00503919 | 2.51315 | lnc-LRRC1-5 | LNCipedia lincRNA (lnc-LRRC1-5), lincRNA [lnc-LRRC1-5:1] |
| A_22_P00006311 | 0.015197945 | 2.6476474 |  | EST97010 Testis I Homo sapiens cDNA 5' end, mRNA sequence [AA383583] |
| A_21_P0005646 | 0.006751373 | 2.851344 | lnc-MYOM2-1 | LNCipedia lincRNA (lnc-MYOM2-1), lincRNA [lnc-MYOM2-1:2] |
| A_33_P3261019 | 0.008372664 | -2.2677639 | SLC9A2 | Homo sapiens solute carrier family 9, subfamily A (NHE2, cation proton antiporter 2), member 2 (SLC9A2), mRNA [NM_003048] |
| A_22_P00002395 | 0.001574271 | 3.8718498 | OTX2-AS1 | Homo sapiens OTX2 antisense RNA 1 (head to head) (OTX2-AS1), long non-coding RNA [NR_029385] |
| A_33_P3396404 | 0.010564335 | 2.705576 | CLLU1OS | Homo sapiens chronic lymphocytic leukemia up-regulated 1 opposite strand (CLLU1OS), mRNA [NM_001025232] |
| A_21_P0005827 | 0.01038959 | 2.30558 | lnc-PLAT-2 | LNCipedia lincRNA (lnc-PLAT-2), lincRNA [lnc-PLAT-2:1] |
| A_33_P3247310 | 0.004554661 | 6.2155895 |  | olfactory receptor, family 5, subfamily J, member 1 pseudogene [Source:HGNC Symbol;Acc:HGNC:8348] [ENST00000313544] |
| A_22_P00001159 | 6.91E-05 | 3.3703816 |  | T cell receptor gamma variable 1 (non-functional) [Source:HGNC Symbol;Acc:HGNC:12284] [ENST00000390348] |
| A_21_P0001878 | 3.38E-04 | 4.6988244 | LINC01143 | Homo sapiens long intergenic non-protein coding RNA 1143 (LINC01143), long non-coding RNA [NR_126384] |
| A_33_P3375794 | 0.009440952 | 2.2654445 |  |  |
| A_24_P133584 | 0.016060872 | -2.831088 | MFGE8 | Homo sapiens milk fat globule-EGF factor 8 protein (MFGE8), transcript variant 1, mRNA [NM_005928] |
| A_21_P0001473 | 0.012117199 | 3.8941793 | lnc-RCC2-2 | LNCipedia lincRNA (lnc-RCC2-2), lincRNA [lnc-RCC2-2:1] |
| A_21_P0008024 | 0.002848276 | 4.995804 | LINC00442 | Homo sapiens long intergenic non-protein coding RNA 442 (LINC00442), long non-coding RNA [NR_026852] |
| A_33_P6816999 | 8.68E-04 | -2.4616406 | THUMPD3-AS1 | Homo sapiens THUMPD3 antisense RNA 1 (THUMPD3-AS1), long non-coding RNA [NR_027007] |
| A_21_P0002258 | 0.016494371 | 2.4291573 | lnc-EIF5B-1 | LNCipedia lincRNA (lnc-EIF5B-1), lincRNA [lnc-EIF5B-1:1] |
| A_21_P0003715 | 0.013417468 | 2.2057924 | lnc-CYTL1-1 | LNCipedia lincRNA (lnc-CYTL1-1), lincRNA [lnc-CYTL1-1:1] |
| A_21_P0010995 | 0.003778603 | 5.6980906 | XLOC_l2_002441 | BROAD Institute lincRNA (XLOC_l2_002441), lincRNA [TCONS_l2_00004725] |
| A_22_P00006403 | 0.014009653 | 2.3999705 | lnc-FBXL7-1 | BX091388 Soares_fetal_lung_NbHL19W Homo sapiens cDNA clone IMAGp998B01669 ; IMAGE:299040, mRNA sequence [BX091388] |
| A_21_P0011748 | 0.010815636 | 4.566553 |  | carcinoembryonic antigen-related cell adhesion molecule pseudogene 1 [Source:HGNC Symbol;Acc:HGNC:1821] [ENST00000434624] |
| A_22_P00019824 | 0.011216838 | 2.5414476 |  | DB095917 TESTI4 Homo sapiens cDNA clone TESTI4046711 5', mRNA sequence [DB095917] |
| A_21_P0006229 | 0.007604716 | 2.0617697 | lnc-CDK9-1 | LNCipedia lincRNA (lnc-CDK9-1), lincRNA [lnc-CDK9-1:2] |
| A_23_P142075 | 0.010572572 | 2.0161548 | ACP5 | Homo sapiens acid phosphatase 5, tartrate resistant (ACP5), transcript variant 4, mRNA [NM_001611] |
| A_21_P0004236 | 0.018450692 | 2.781079 | lnc-C5orf17-1 | LNCipedia lincRNA (lnc-C5orf17-1), lincRNA [lnc-C5orf17-1:2] |
| A_21_P0012517 | 0.015858151 | 2.7087107 | LINC01206 | Homo sapiens long intergenic non-protein coding RNA 1206 (LINC01206), long non-coding RNA [NR_104146] |
| A_23_P164773 | 0.004892541 | 2.2543554 | FCER2 | Homo sapiens Fc fragment of IgE, low affinity II, receptor for (CD23) (FCER2), transcript variant 1, mRNA [NM_002002] |
| A_21_P0013693 | 0.017981885 | 3.0757918 | ATP8B5P | ATPase, class I, type 8B, member 5, pseudogene [Source:HGNC Symbol;Acc:HGNC:27245] [ENST00000423138] |
| A_33_P3417123 | 0.017579252 | 2.120576 |  |  |
| A_22_P00002855 | 0.01437141 | 2.9283984 | GRM7-AS1 | Homo sapiens GRM7 antisense RNA 1 (GRM7-AS1), long non-coding RNA [NR_046606] |
| A_21_P0005317 | 0.007796347 | 2.70385 |  | PREDICTED: Homo sapiens uncharacterized LOC101927473 (LOC101927473), ncRNA [XR_250237] |
| A_21_P0001237 | 0.004406112 | 3.1941724 |  |  |
| A_22_P00010400 | 0.006165735 | 5.7426753 |  | long intergenic non-protein coding RNA 1067 [Source:HGNC Symbol;Acc:HGNC:49105] [ENST00000432575] |
| A_22_P00021088 | 0.002277691 | 3.6730251 | LOC101927132 | Homo sapiens uncharacterized LOC101927132 (LOC101927132), long non-coding RNA [NR_110650] |
| A_22_P00005810 | 0.008900492 | 2.6348352 |  | Homo sapiens cDNA clone IMAGE:5272798. [BC033941] |
| A_33_P3397785 | 0.012797289 | 2.234988 | SYT2 | Homo sapiens synaptotagmin II (SYT2), transcript variant 2, mRNA [NM_001136504] |
| A_33_P3377239 | 0.019596959 | -2.5594401 | LOC100270804 | Homo sapiens uncharacterized LOC100270804 (LOC100270804), long non-coding RNA [NR_026885] |
| A_21_P0010300 | 7.06E-04 | 3.6672502 | lnc-TSPEAR-1 | LNCipedia lincRNA (lnc-TSPEAR-1), lincRNA [lnc-TSPEAR-1:3] |
| A_33_P3316899 | 0.010407414 | 2.7245967 | RALYL | Homo sapiens RALY RNA binding protein-like (RALYL), transcript variant 3, mRNA [NM_173848] |
| A_22_P00014880 | 2.29E-04 | 2.3677597 | CDX1 | Homo sapiens caudal type homeobox 1 (CDX1), mRNA [NM_001804] |
| A_21_P0002095 | 0.007604716 | 4.149479 |  |  |
| A_21_P0003739 | 0.010529741 | 4.2909346 | lnc-AC021860.1-4 | LNCipedia lincRNA (lnc-AC021860.1-4), lincRNA [lnc-AC021860.1-4:1] |
| A_23_P25525 | 0.006619435 | -3.4226954 | GTF3A | Homo sapiens general transcription factor IIIA (GTF3A), mRNA [NM_002097] |
| A_21_P0004729 | 0.003956788 | 2.4523187 | LINC00518 | long intergenic non-protein coding RNA 518 [Source:HGNC Symbol;Acc:HGNC:28626] [ENST00000491317] |
| A_23_P250302 | 0.002822993 | 6.084044 | CCR3 | Homo sapiens chemokine (C-C motif) receptor 3 (CCR3), transcript variant 1, mRNA [NM_001837] |
| A_21_P0002480 | 0.017311543 | 2.8871908 | lnc-TGFBRAP1-11 | LNCipedia lincRNA (lnc-TGFBRAP1-11), lincRNA [lnc-TGFBRAP1-11:2] |
| A_22_P00001699 | 0.019570038 | 2.8331876 | lnc-ATF3-1 | Q6C5E4_YARLI (Q6C5E4) Similarity, partial (4%) [THC2723091] |
| A_22_P00009060 | 0.016265098 | 2.1998322 | lnc-LENG9-2 | 17000423781836 GRN_EB Homo sapiens cDNA 5', mRNA sequence [CN315368] |
| A_23_P70060 | 0.012784615 | -2.1097963 | PPAP2A | Homo sapiens phosphatidic acid phosphatase type 2A (PPAP2A), transcript variant 2, mRNA [NM_176895] |
| A_32_P6015 | 0.009375964 | 2.5564492 | MNX1 | Homo sapiens motor neuron and pancreas homeobox 1 (MNX1), transcript variant 1, mRNA [NM_005515] |
| A_23_P22565 | 6.49E-04 | 4.746804 | FATE1 | Homo sapiens fetal and adult testis expressed 1 (FATE1), mRNA [NM_033085] |
| A_22_P00021465 | 0.009910665 | 2.086431 | FLJ46836 | PREDICTED: Homo sapiens FLJ46836 protein (FLJ46836), misc_RNA [XR_108962] |
| A_33_P3318861 | 0.013622328 | 2.2152681 | DYTN | Homo sapiens dystrotelin (DYTN), mRNA [NM_001093730] |
| A_21_P0009030 | 7.53E-04 | 3.6186686 | C16orf97 | Homo sapiens chromosome 16 open reading frame 97 (C16orf97), mRNA [NM_001242473] |
| A_22_P00009224 | 0.005637546 | 2.8092134 | HTR2A-AS1 | Homo sapiens HTR2A antisense RNA 1 (HTR2A-AS1), transcript variant 1, long non-coding RNA [NR_046612] |
| A_23_P41713 | 0.007882567 | 3.2942307 | FAM71B | Homo sapiens family with sequence similarity 71, member B (FAM71B), mRNA [NM_130899] |
| A_33_P3409508 | 0.011863682 | 2.3316252 | MAPK11 | Homo sapiens mitogen-activated protein kinase 11 (MAPK11), transcript variant 1, mRNA [NM_002751] |
| A_22_P00024107 | 0.004458424 | 4.4511743 |  |  |
| A_33_P3320408 | 0.01484621 | 3.9781225 |  |  |
| A_22_P00011852 | 0.001606751 | 2.886532 | lnc-PHTF2-1 | LNCipedia lincRNA (lnc-PHTF2-1), lincRNA [lnc-PHTF2-1:1] |
| A_22_P00009003 | 2.48E-04 | 5.8281937 |  | 6551049H1 BRAFNON02 Homo sapiens cDNA clone 6551049 5', mRNA sequence [BU584707] |
| A_23_P329890 | 0.01657353 | -2.1134882 | TMEM136 | Homo sapiens transmembrane protein 136 (TMEM136), transcript variant 2, mRNA [NM_174926] |
| A_21_P0008992 | 0.008372501 | 3.6510162 | lnc-JPH3-3 | LNCipedia lincRNA (lnc-JPH3-3), lincRNA [lnc-JPH3-3:7] |
| A_32_P155826 | 0.013692818 | 2.092065 | USP27X-AS1 | Homo sapiens USP27X antisense RNA 1 (head to head) (USP27X-AS1), long non-coding RNA [NR_026742] |
| A_21_P0009950 | 0.012676228 | 2.1127229 | lnc-SSTR4-2 | LNCipedia lincRNA (lnc-SSTR4-2), lincRNA [lnc-SSTR4-2:4] |
| A_22_P00012585 | 0.018676445 | 4.040456 |  | Homo sapiens cDNA clone IMAGE:5442191, partial cds. [BC065363] |
| A_21_P0002415 | 0.014009653 | 2.178223 | lnc-ATAD2B-1 | LNCipedia lincRNA (lnc-ATAD2B-1), lincRNA [lnc-ATAD2B-1:1] |
| A_21_P0006275 | 0.011370456 | 3.918143 | lnc-PTAR1-1 | LNCipedia lincRNA (lnc-PTAR1-1), lincRNA [lnc-PTAR1-1:1] |
| A_21_P0001820 | 0.003409261 | 4.446706 |  | Q2RQD4_RHORT (Q2RQD4) Predicted signal transduction protein containing EFhand domain, partial (5%) [THC2776395] |
| A_22_P00025227 | 0.004436465 | 2.4136183 | lnc-IL3-3 | LNCipedia lincRNA (lnc-IL3-3), lincRNA [lnc-IL3-3:1] |
| A_21_P0009164 | 9.74E-04 | 3.2040741 | lnc-PIPOX-1 | LNCipedia lincRNA (lnc-PIPOX-1), lincRNA [lnc-PIPOX-1:4] |
| A_33_P3363859 | 0.00704779 | 3.8478866 | LOC100130452 | Homo sapiens uncharacterized LOC100130452 (LOC100130452), long non-coding RNA [NR_034036] |
| A_33_P3277898 | 0.010916906 | 2.8376796 | VSTM2A | Homo sapiens V-set and transmembrane domain containing 2A (VSTM2A), transcript variant 1, mRNA [NM_182546] |
| A_22_P00014206 | 0.0192649 | 2.182858 | lnc-SEC16B.1-4 | Homo sapiens, clone IMAGE:4770655, mRNA. [BC026287] |
| A_21_P0002439 | 0.005064829 | 5.629947 | lnc-PIGF-2 | UI-CF-EC1-acf-e-05-18-UI.s18 UI-CF-EC1 Homo sapiens cDNA clone UI-CF-EC1-acf-e-05-18-UI 3', mRNA sequence [CF890804] |
| A_23_P81158 | 0.001724501 | -2.415953 | ADH1C | Homo sapiens alcohol dehydrogenase 1C (class I), gamma polypeptide (ADH1C), mRNA [NM_000669] |
| A_21_P0014642 | 0.01579848 | 2.5753927 |  | PREDICTED: Homo sapiens uncharacterized LOC100507222 (LOC100507222), misc_RNA [XR_171713] |
| A_33_P3406843 | 3.97E-04 | 3.0754933 | LOC442132 | Homo sapiens golgin A6 family-like 1 pseudogene (LOC442132), non-coding RNA [NR_033906] |
| A_24_P535219 | 0.019070894 | -2.1457953 | PHF10 | PHD finger protein 10 [Source:HGNC Symbol;Acc:HGNC:18250] [ENST00000612128] |
| A_21_P0005733 | 0.011539546 | 4.9500494 | lnc-CA2-1 | LNCipedia lincRNA (lnc-CA2-1), lincRNA [lnc-CA2-1:1] |
| A_21_P0006901 | 0.017439516 | 2.1746926 | lnc-CNNM1-2 | LNCipedia lincRNA (lnc-CNNM1-2), lincRNA [lnc-CNNM1-2:1] |
| A_22_P00013988 | 0.013511646 | 2.903612 |  |  |
| A_33_P7363082 | 0.00704779 | -2.59096 | CWC15 | Homo sapiens CWC15 spliceosome-associated protein (CWC15), mRNA [NM_016403] |
| A_21_P0000497 | 0.006416088 | 2.2811568 | SNORD1B | Homo sapiens small nucleolar RNA, C/D box 1B (SNORD1B), small nucleolar RNA [NR_004396] |
| A_22_P00003413 | 9.54E-04 | 4.015142 | LOC102725407 | PREDICTED: Homo sapiens uncharacterized LOC102725407 (LOC102725407), transcript variant X1, ncRNA [XR_425532] |
| A_21_P0006565 | 2.48E-04 | 2.5833771 | LOC102724212 | PREDICTED: Homo sapiens uncharacterized LOC102724212 (LOC102724212), ncRNA [XR_426536] |
| A_22_P00015783 | 0.002526455 | 3.7377985 |  | DKFZp564E247_r1 564 (synonym: hfbr2) Homo sapiens cDNA clone DKFZp564E247 5', mRNA sequence [AL037786] |
| A_33_P3645079 | 0.008061801 | 2.4800808 | HOXA-AS3 | Homo sapiens HOXA cluster antisense RNA 3 (HOXA-AS3), transcript variant 1, long non-coding RNA [NR_038831] |
| A_33_P3424295 | 8.18E-06 | 4.152437 | TRAF2 | Homo sapiens TNF receptor-associated factor 2 (TRAF2), mRNA [NM_021138] |
| A_22_P00008195 | 0.01788836 | 2.9903421 | INSL6 | Homo sapiens insulin-like 6 (INSL6), mRNA [NM_007179] |
| A_22_P00011626 | 0.015121243 | 2.9154851 |  | BX112869 NCI_CGAP_GCB1 Homo sapiens cDNA clone IMAGp998D123288, mRNA sequence [BX112869] |
| A_21_P0014126 | 0.009688721 | -2.1598597 |  |  |
| A_21_P0006312 | 0.019620985 | 2.9140615 | lnc-SLC46A2-2 | LNCipedia lincRNA (lnc-SLC46A2-2), lincRNA [lnc-SLC46A2-2:1] |
| A_22_P00000490 | 0.019157581 | 2.4748774 |  | Homo sapiens full length insert cDNA clone ZE16D09. [AF086565] |
| A_21_P0009729 | 0.002455926 | 7.2435627 | lnc-NLRP4-1 | LNCipedia lincRNA (lnc-NLRP4-1), lincRNA [lnc-NLRP4-1:1] |
| A_21_P0011394 | 0.005949104 | 8.254878 | HERC2P9 | hect domain and RLD 2 pseudogene 9 [Source:HGNC Symbol;Acc:HGNC:30495] [ENST00000524476] |
| A_21_P0007071 | 0.004234207 | 2.2665074 | lnc-FRAT1-1 | LNCipedia lincRNA (lnc-FRAT1-1), lincRNA [lnc-FRAT1-1:2] |
| A_22_P00022752 | 0.006207719 | 3.385178 | lnc-C5orf27-2 | LNCipedia lincRNA (lnc-C5orf27-2), lincRNA [lnc-C5orf27-2:2] |
| A_22_P00021149 | 1.55E-04 | 2.9784398 | lnc-AL450307.1-1 | Homo sapiens cDNA FLJ46127 fis, clone TESTI2041976. [AK128008] |
| A_33_P3286318 | 0.017040258 | -2.1242301 |  | Homo sapiens phosphoglycerate mutase 1 (brain), mRNA (cDNA clone IMAGE:3689964), partial cds. [BC011771] |
| A_21_P0007385 | 2.86E-04 | 4.796076 | lnc-FAM76B-2 | LNCipedia lincRNA (lnc-FAM76B-2), lincRNA [lnc-FAM76B-2:1] |
| A_22_P00023030 | 0.013622328 | 2.4909227 |  |  |
| A_21_P0001640 | 0.00781545 | 2.670692 | lnc-AGT-1 | LNCipedia lincRNA (lnc-AGT-1), lincRNA [lnc-AGT-1:1] |
| A_33_P3400302 | 0.015843261 | 2.860427 | TTTY18 | Homo sapiens testis-specific transcript, Y-linked 18 (non-protein coding) (TTTY18), long non-coding RNA [NR_001550] |
| A_24_P141707 | 1.76E-04 | 4.1002913 | INHBE | Homo sapiens inhibin, beta E (INHBE), mRNA [NM_031479] |
| A_33_P3415972 | 0.004122045 | 3.764401 | SLC35F1 | Homo sapiens solute carrier family 35, member F1 (SLC35F1), mRNA [NM_001029858] |
| A_21_P0009335 | 0.005923995 | 2.579926 | lnc-C17orf67-2 | LNCipedia lincRNA (lnc-C17orf67-2), lincRNA [lnc-C17orf67-2:1] |
| A_24_P344711 | 0.018414054 | -2.5675592 | AGPAT3 | Homo sapiens 1-acylglycerol-3-phosphate O-acyltransferase 3 (AGPAT3), transcript variant 1, mRNA [NM_020132] |
| A_22_P00012875 | 0.002822993 | 3.1571598 | lnc-RBFOX1-1 | LNCipedia lincRNA (lnc-RBFOX1-1), lincRNA [lnc-RBFOX1-1:1] |
| A_21_P0001661 | 0.010154004 | 2.513644 | LOC102724429 | PREDICTED: Homo sapiens uncharacterized LOC102724429 (LOC102724429), ncRNA [XR_425225] |
| A_24_P677634 | 0.006488535 | -2.0842378 | LOC493754 | Homo sapiens RAB guanine nucleotide exchange factor (GEF) 1 pseudogene (LOC493754), transcript variant 6, non-coding RNA [NR_111977] |
| A_33_P3312251 | 0.00635109 | 3.8329759 |  |  |
| A_22_P00002072 | 0.003419576 | 3.1356776 | SPAG6 | sperm associated antigen 6 [Source:HGNC Symbol;Acc:HGNC:11215] [ENST00000487973] |
| A_23_P13102 | 0.002131335 | 2.5598664 | CASP12 | Homo sapiens caspase 12 (gene/pseudogene) (CASP12), transcript variant 1, mRNA [NM_001191016] |
| A_21_P0012849 | 0.003686276 | 5.5852985 | XLOC_l2_011744 | BROAD Institute lincRNA (XLOC_l2_011744), lincRNA [TCONS_l2_00022506] |
| A_24_P333532 | 0.019925974 | 2.2372503 | LINC00589 | Homo sapiens long intergenic non-protein coding RNA 589 (LINC00589), long non-coding RNA [NR_026765] |
| A_23_P384517 | 0.015057466 | -2.6591413 | GYG1 | Homo sapiens glycogenin 1 (GYG1), transcript variant 1, mRNA [NM_004130] |
| A_24_P380679 | 0.009316924 | -2.8154402 | LSMEM1 | Homo sapiens leucine-rich single-pass membrane protein 1 (LSMEM1), transcript variant 1, mRNA [NM_182597] |
| A_21_P0007927 | 0.01062803 | 2.0612874 | USP12-AS2 | Homo sapiens USP12 antisense RNA 2 (head to head) (USP12-AS2), long non-coding RNA [NR_046548] |
| A_33_P3357609 | 0.010105339 | 3.0186002 | ZBP1 | Homo sapiens Z-DNA binding protein 1 (ZBP1), transcript variant 4, mRNA [NM_001160419] |
| A_33_P3251663 | 0.013135852 | 6.0559793 |  | PREDICTED: Homo sapiens myomegalin-like (LOC102723955), transcript variant X3, mRNA [XM_006711717] |
| A_22_P00001901 | 0.013622328 | 4.5567245 |  |  |
| A_19_P00322997 | 0.016797222 | 2.3758926 | LOC101928682 | Homo sapiens uncharacterized LOC101928682 (LOC101928682), long non-coding RNA [NR_104667] |
| A_21_P0002104 | 0.00631611 | 2.2840674 | lnc-FAM168B-1 | LNCipedia lincRNA (lnc-FAM168B-1), lincRNA [lnc-FAM168B-1:3] |
| A_23_P314672 | 0.012042754 | 2.0849335 | C11orf40 | Homo sapiens chromosome 11 open reading frame 40 (C11orf40), mRNA [NM_144663] |
| A_21_P0002899 | 0.002822993 | 4.585223 | lnc-RP11-432B6.3.1-1 | LNCipedia lincRNA (lnc-RP11-432B6.3.1-1), lincRNA [lnc-RP11-432B6.3.1-1:1] |
| A_21_P0010354 | 0.008488857 | 3.2369108 |  |  |
| A_33_P3401317 | 0.017817039 | 2.429729 | POTEM | Homo sapiens POTE ankyrin domain family, member M (POTEM), mRNA [NM_001145442] |
| A_33_P3766635 | 0.00918235 | 3.9408433 |  | olfactory receptor, family 10, subfamily D, member 3 (non-functional) [Source:HGNC Symbol;Acc:HGNC:8168] [ENST00000318666] |
| A_23_P162702 | 0.015922625 | -2.3203707 | HECTD4 | Homo sapiens HECT domain containing E3 ubiquitin protein ligase 4 (HECTD4), mRNA [NM_001109662] |
| A_33_P3363685 | 0.004017944 | 4.131872 | OR52A5 | Homo sapiens olfactory receptor, family 52, subfamily A, member 5 (OR52A5), mRNA [NM_001005160] |
| A_24_P589266 | 0.015294367 | -2.5042279 | SH3RF3 | Homo sapiens SH3 domain containing ring finger 3 (SH3RF3), mRNA [NM_001099289] |
| A_22_P00013989 | 0.008649809 | 2.737191 |  | DB054543 TESTI2 Homo sapiens cDNA clone TESTI2045424 5', mRNA sequence [DB054543] |
| A_21_P0001186 | 9.46E-04 | 3.3516626 |  |  |
| A_21_P0013011 | 0.016775608 | 3.4283094 |  |  |
| A_22_P00009109 | 0.007796525 | 3.237138 | lnc-LIMCH1-2 | DA608549 IMR322 Homo sapiens cDNA clone IMR322005977 5', mRNA sequence [DA608549] |
| A_33_P3418486 | 0.007081766 | 4.2863503 |  | 602698696F1 NIH_MGC_97 Homo sapiens cDNA clone IMAGE:4830958 5', mRNA sequence [BG722090] |
| A_33_P3285602 | 0.016380092 | 2.358137 |  | MYCL pseudogene 1 [Source:HGNC Symbol;Acc:HGNC:7556] [ENST00000372451] |
| A_21_P0009584 | 0.010037025 | 3.3351848 | lnc-ZNF516-8 | LNCipedia lincRNA (lnc-ZNF516-8), lincRNA [lnc-ZNF516-8:1] |
| A_22_P00018755 | 0.011321843 | 2.085609 | lnc-CALCOCO2-5 | UI-H-BI1-abw-f-07-0-UI.s1 NCI_CGAP_Sub3 Homo sapiens cDNA clone IMAGE:2713476 3', mRNA sequence [AW138088] |
| A_32_P162192 | 1.72E-04 | 3.709347 | LINC00221 | Homo sapiens long intergenic non-protein coding RNA 221 (LINC00221), long non-coding RNA [NR_027457] |
| A_22_P00024899 | 0.01952078 | 6.4937305 |  | PREDICTED: Homo sapiens uncharacterized LOC100505775 (LOC100505775), misc_RNA [XR_133485] |
| A_33_P3300600 | 6.91E-05 | 2.3101354 | OR7G1 | Homo sapiens olfactory receptor, family 7, subfamily G, member 1 (OR7G1), mRNA [NM_001005192] |
| A_19_P00804922 | 0.002719358 | 2.264673 | lnc-OSTC-2 | LNCipedia lincRNA (lnc-OSTC-2), lincRNA [lnc-OSTC-2:1] |
| A_33_P3406090 | 0.012418595 | 2.2033658 |  |  |
| A_22_P00016669 | 4.69E-05 | 2.2632456 | LINC00661 | Homo sapiens long intergenic non-protein coding RNA 661 (LINC00661), long non-coding RNA [NR_026828] |
| A_33_P3258478 | 0.016549937 | 2.5498726 |  | killer cell immunoglobulin-like receptor, three domains, pseudogene 1 [Source:HGNC Symbol;Acc:HGNC:16343] [ENST00000611586] |
| A_23_P326414 | 0.014069748 | 3.2968597 | LINC00602 | Homo sapiens long intergenic non-protein coding RNA 602 (LINC00602), long non-coding RNA [NR_027284] |
| A_33_P3692984 | 0.004047977 | 3.5838966 |  | long intergenic non-protein coding RNA 567 [Source:HGNC Symbol;Acc:HGNC:43711] [ENST00000569854] |
| A_22_P00016492 | 3.77E-04 | 6.586146 |  |  |
| A_22_P00004097 | 0.016114652 | 5.266866 | lnc-CITED2-3 | LNCipedia lincRNA (lnc-CITED2-3), lincRNA [lnc-CITED2-3:1] |
| A_33_P3382403 | 0.004017944 | 7.4865246 | SPATA31A3 | Homo sapiens SPATA31 subfamily A, member 3 (SPATA31A3), mRNA [NM_001083124] |
| A_23_P80662 | 0.01865728 | 3.1374874 | SOX14 | Homo sapiens SRY (sex determining region Y)-box 14 (SOX14), mRNA [NM_004189] |
| A_21_P0007294 | 0.005132849 | 3.6515782 | lnc-RP11-688I9.2.1-3 | LNCipedia lincRNA (lnc-RP11-688I9.2.1-3), lincRNA [lnc-RP11-688I9.2.1-3:1] |
| A_33_P3278275 | 0.018005375 | 2.8703635 | TXNDC8 | thioredoxin domain containing 8 (spermatozoa) [Source:HGNC Symbol;Acc:HGNC:31454] [ENST00000374511] |
| A_21_P0002105 | 0.013157161 | 4.242441 | LOC101928067 | long intergenic non-protein coding RNA 349 [Source:HGNC Symbol;Acc:HGNC:42667] [ENST00000448748] |
| A_23_P43107 | 0.018050106 | 2.7839856 | DCSTAMP | Homo sapiens dendrocyte expressed seven transmembrane protein (DCSTAMP), transcript variant 1, mRNA [NM_030788] |
| A_23_P74870 | 9.80E-05 | 3.52126 | MROH9 | Homo sapiens maestro heat-like repeat family member 9 (MROH9), transcript variant 2, mRNA [NM_025063] |
| A_21_P0002800 | 0.005862902 | 2.0579846 |  | long intergenic non-protein coding RNA 1330 [Source:HGNC Symbol;Acc:HGNC:50536] [ENST00000496891] |
| A_22_P00006876 | 0.004047977 | 3.093749 |  |  |
| A_22_P00010173 | 0.018859727 | -2.0586436 | FGD5-AS1 | Homo sapiens FGD5 antisense RNA 1 (FGD5-AS1), transcript variant 1, long non-coding RNA [NR_046251] |
| A_22_P00004833 | 1.16E-04 | 2.254271 | lnc-CYB561D2-1 | LNCipedia lincRNA (lnc-CYB561D2-1), lincRNA [lnc-CYB561D2-1:1] |
| A_22_P00022284 | 0.005983065 | 2.8342204 |  | DA781732 OCBBF2 Homo sapiens cDNA clone OCBBF2017344 5', mRNA sequence [DA781732] |
| A_21_P0006126 | 0.013368452 | 3.3067567 | lnc-TYRP1-2 | LNCipedia lincRNA (lnc-TYRP1-2), lincRNA [lnc-TYRP1-2:1] |
| A_23_P171409 | 0.002151727 | 2.846953 | TTTY6 | Homo sapiens testis-specific transcript, Y-linked 6 (non-protein coding) (TTTY6), long non-coding RNA [NR_001527] |
| A_22_P00017268 | 3.77E-04 | 2.326364 | LOC101927210 | PREDICTED: Homo sapiens uncharacterized LOC101927210 (LOC101927210), ncRNA [XR_243979] |
| A_21_P0008550 | 0.008719602 | 2.2893705 | lnc-BCL11B-1 | LNCipedia lincRNA (lnc-BCL11B-1), lincRNA [lnc-BCL11B-1:3] |
| A_23_P111005 | 0.019578865 | -3.0752342 | YIPF3 | Homo sapiens Yip1 domain family, member 3 (YIPF3), mRNA [NM_015388] |
| A_33_P3546161 | 6.49E-04 | 3.8814104 |  | RC2-BT0841-021000-012-c08 BT0841 Homo sapiens cDNA, mRNA sequence [BF742660] |
| A_33_P3576797 | 0.016549198 | -2.243165 | LOC158863 | Homo sapiens mRNA; cDNA DKFZp586J1922 (from clone DKFZp586J1922) [AL110203] |
| A_21_P0012889 | 0.007796347 | 2.377873 |  |  |
| A_33_P3373745 | 0.01693474 | -2.1115408 | BRD4 | Homo sapiens bromodomain containing 4 (BRD4), transcript variant short, mRNA [NM_014299] |
| A_21_P0009300 | 0.004653566 | 5.6715093 | lnc-PMP22-2 | LNCipedia lincRNA (lnc-PMP22-2), lincRNA [lnc-PMP22-2:1] |
| A_21_P0006384 | 0.00540034 | 3.282175 | lnc-DIRAS2-1 | LNCipedia lincRNA (lnc-DIRAS2-1), lincRNA [lnc-DIRAS2-1:4] |
| A_33_P3321796 | 0.011058508 | 2.0814564 |  | DB153536 THYMU3 Homo sapiens cDNA clone THYMU3031175 5', mRNA sequence [DB153536] |
| A_21_P0005466 | 2.66E-04 | 3.2721307 | lnc-RADIL-3 | LNCipedia lincRNA (lnc-RADIL-3), lincRNA [lnc-RADIL-3:1] |
| A_24_P242688 | 0.018621914 | -2.310568 | HADHA | Homo sapiens hydroxyacyl-CoA dehydrogenase/3-ketoacyl-CoA thiolase/enoyl-CoA hydratase (trifunctional protein), alpha subunit (HADHA), mRNA [NM_000182] |
| A_23_P331700 | 0.010973383 | 2.715353 | SRRM3 | Homo sapiens serine/arginine repetitive matrix 3 (SRRM3), transcript variant 1, mRNA [NM_001291831] |
| A_21_P0000994 | 0.006640496 | 2.164992 |  |  |
| A_23_P94186 | 0.011191177 | 3.0703957 | LYPD2 | Homo sapiens LY6/PLAUR domain containing 2 (LYPD2), mRNA [NM_205545] |
| A_19_P00317904 | 0.00631611 | 2.2433283 | lnc-ZNF674-3 | Homo sapiens cDNA FLJ25917 fis, clone CBR04926. [AK098783] |
| A_21_P0011918 | 0.006844481 | 3.7971575 |  |  |
| A_23_P367071 | 0.012697827 | 2.89639 | UBE2DNL | Homo sapiens ubiquitin-conjugating enzyme E2D N-terminal like (pseudogene) (UBE2DNL), non-coding RNA [NR_024062] |
| A_21_P0011253 | 3.09E-04 | 2.8280659 | XLOC_l2_004283 | BROAD Institute lincRNA (XLOC_l2_004283), lincRNA [TCONS_l2_00007890] |
| A_21_P0007174 | 0.002705118 | 3.899542 | lnc-CTR9-3 | LNCipedia lincRNA (lnc-CTR9-3), lincRNA [lnc-CTR9-3:7] |
| A_21_P0011951 | 0.016926367 | 2.0936296 | XLOC_l2_008203 | BROAD Institute lincRNA (XLOC_l2_008203), lincRNA [TCONS_l2_00014795] |
| A_21_P0014043 | 0.002620795 | 3.7183566 |  | EF-hand calcium binding domain 8 [Source:HGNC Symbol;Acc:HGNC:34532] [ENST00000400522] |
| A_22_P00006811 | 0.005923995 | 2.2049053 | lnc-GABPA-5 | yf67e05.r1 Soares infant brain 1NIB Homo sapiens cDNA clone IMAGE:27056 5', mRNA sequence [R18879] |
| A_21_P0005666 | 0.006167846 | 2.367548 | lnc-INTS10-1 | LNCipedia lincRNA (lnc-INTS10-1), lincRNA [lnc-INTS10-1:1] |
| A_21_P0009684 | 0.013784705 | 2.5291722 | lnc-ZNF333-2 | LNCipedia lincRNA (lnc-ZNF333-2), lincRNA [lnc-ZNF333-2:1] |
| A_33_P3740427 | 0.018528204 | 6.1451225 | LINC00700 | Homo sapiens long intergenic non-protein coding RNA 700 (LINC00700), long non-coding RNA [NR_040253] |
| A_33_P3274422 | 0.008277085 | 3.4206495 |  | MAP/microtubule affinity-regulating kinase 2 pseudogene 10 [Source:HGNC Symbol;Acc:HGNC:39801] [ENST00000454959] |
| A_19_P00318187 | 0.003916526 | 3.4502838 | LOC102724861 |  |
| A_23_P166663 | 0.012477387 | -2.39802 | APPL1 | Homo sapiens adaptor protein, phosphotyrosine interaction, PH domain and leucine zipper containing 1 (APPL1), mRNA [NM_012096] |
| A_23_P58132 | 0.018612748 | 2.265605 | RHOH | Homo sapiens ras homolog family member H (RHOH), transcript variant 6, mRNA [NM_004310] |
| A_33_P3263459 | 0.007765223 | 2.647677 | MPPED1 | Homo sapiens metallophosphoesterase domain containing 1 (MPPED1), mRNA [NM_001044370] |
| A_21_P0009366 | 0.012042754 | 2.691114 |  |  |
| A_22_P00006543 | 0.011058508 | 2.93966 |  | UI-E-EJ1-ajj-n-21-0-UI.r1 UI-E-EJ1 Homo sapiens cDNA clone UI-E-EJ1-ajj-n-21-0-UI 5', mRNA sequence [BM929797] |
| A_23_P371758 | 0.010969911 | 3.1867936 | SDR9C7 | Homo sapiens short chain dehydrogenase/reductase family 9C, member 7 (SDR9C7), mRNA [NM_148897] |
| A_21_P0010285 | 0.004695749 | 2.379881 | lnc-PSMG1-2 | LNCipedia lincRNA (lnc-PSMG1-2), lincRNA [lnc-PSMG1-2:1] |
| A_21_P0004397 | 0.005803804 | 2.127116 | lnc-ACTBL2-1 | DA037643 BGGI11 Homo sapiens cDNA clone BGGI11000600 5', mRNA sequence [DA037643] |
| A_22_P00010444 | 0.003190245 | 4.514933 | LOC101927623 | Homo sapiens uncharacterized LOC101927623 (LOC101927623), long non-coding RNA [NR_110996] |
| A_22_P00019274 | 0.009997211 | 3.2828472 | lnc-MTERFD3-2 | 602466053F1 NIH_MGC_75 Homo sapiens cDNA clone IMAGE:4594260 5', mRNA sequence [BG401516] |
| A_24_P280762 | 0.013569303 | 2.2339177 | KHDC1 | Homo sapiens KH homology domain containing 1 (KHDC1), transcript variant 2, mRNA [NM_030568] |
| A_22_P00011633 | 0.015984988 | 4.251082 | lnc-PCYOX1-2 | DA451014 CTONG2 Homo sapiens cDNA clone CTONG2023314 5', mRNA sequence [DA451014] |
| A_23_P4133 | 0.012357703 | 2.1856067 | AOC2 | Homo sapiens amine oxidase, copper containing 2 (retina-specific) (AOC2), transcript variant 1, mRNA [NM_001158] |
| A_23_P130241 | 2.13E-04 | 2.7709332 | KRTAP1-3 | Homo sapiens keratin associated protein 1-3 (KRTAP1-3), mRNA [NM_030966] |
| A_33_P3285779 | 0.002373447 | 2.1978292 | LOC340113 | Homo sapiens uncharacterized LOC340113 (LOC340113), long non-coding RNA [NR_033832] |
| A_21_P0004554 | 0.013134644 | 2.3975532 | lnc-OXCT1-1 | LNCipedia lincRNA (lnc-OXCT1-1), lincRNA [lnc-OXCT1-1:1] |
| A_33_P3237050 | 7.31E-04 | 4.0954113 | OR4K14 | Homo sapiens olfactory receptor, family 4, subfamily K, member 14 (OR4K14), mRNA [NM_001004712] |
| A_33_P3330179 | 0.003311975 | 2.097412 | LOC100131372 | PREDICTED: Homo sapiens uncharacterized LOC100131372 (LOC100131372), misc_RNA [XR_112955] |
| A_23_P27538 | 0.016380092 | 3.1178393 | CALR3 | Homo sapiens calreticulin 3 (CALR3), mRNA [NM_145046] |
| A_23_P396115 | 0.007823153 | 2.003571 | SSX7 | Homo sapiens synovial sarcoma, X breakpoint 7 (SSX7), mRNA [NM_173358] |
| A_21_P0008646 | 0.010888848 | 3.8343678 | lnc-SLC24A1-1 | LNCipedia lincRNA (lnc-SLC24A1-1), lincRNA [lnc-SLC24A1-1:1] |
| A_22_P00022130 | 0.010153236 | 3.2777078 | lnc-GTPBP8-2 | LNCipedia lincRNA (lnc-GTPBP8-2), lincRNA [lnc-GTPBP8-2:1] |
| A_22_P00002499 | 0.008519789 | 2.7223935 | LOC102724118 | PREDICTED: Homo sapiens uncharacterized LOC102724118 (LOC102724118), ncRNA [XR_429782] |
| A_23_P169460 | 0.002001687 | 2.2600486 | FRMPD1 | Homo sapiens FERM and PDZ domain containing 1 (FRMPD1), mRNA [NM_014907] |
| A_21_P0005289 | 0.008852229 | 2.8836725 |  |  |
| A_33_P3310401 | 0.012357703 | 6.142648 |  |  |
| A_21_P0001462 | 0.009621772 | 3.6130347 | lnc-NPHP4-3 | LNCipedia lincRNA (lnc-NPHP4-3), lincRNA [lnc-NPHP4-3:1] |
| A_21_P0007427 | 0.019543365 | 4.556057 | lnc-TEAD1-1 | LNCipedia lincRNA (lnc-TEAD1-1), lincRNA [lnc-TEAD1-1:1] |
| A_21_P0005378 | 0.018612748 | 2.370309 | lnc-ABCA13-1 | Homo sapiens cDNA clone IMAGE:4827621. [BC042120] |
| A_21_P0002627 | 0.004578975 | 9.159227 | lnc-SPAG16-1 | LNCipedia lincRNA (lnc-SPAG16-1), lincRNA [lnc-SPAG16-1:2] |
| A_22_P00010898 | 0.015958356 | 3.4957414 |  |  |
| A_23_P397856 | 0.014878415 | -2.4186234 | TIRAP | Homo sapiens toll-interleukin 1 receptor (TIR) domain containing adaptor protein (TIRAP), transcript variant 3, mRNA [NM_001039661] |
| A_22_P00013163 | 0.014502258 | -3.1617362 | lnc-RNF113B-2 | Q8NM35_CORGL (Q8NM35) Predicted epimerase, PhzC/PhzF homolog, partial (6%) [THC2590035] |
| A_21_P0001593 | 0.013826597 | 2.952633 | LOC102724358 | PREDICTED: Homo sapiens uncharacterized LOC102724358 (LOC102724358), ncRNA [XR_425046] |
| A_21_P0005350 | 0.009085295 | 3.640952 | lnc-ELFN1-1 | LNCipedia lincRNA (lnc-ELFN1-1), lincRNA [lnc-ELFN1-1:3] |
| A_24_P237036 | 0.01682666 | 3.417718 | TNFSF14 | Homo sapiens tumor necrosis factor (ligand) superfamily, member 14 (TNFSF14), transcript variant 1, mRNA [NM_003807] |
| A_21_P0009261 | 0.013435287 | 2.842905 | lnc-MSI2-2 | LNCipedia lincRNA (lnc-MSI2-2), lincRNA [lnc-MSI2-2:1] |
| A_22_P00010938 | 0.019117348 | 2.8684204 |  | DB083763 TESTI4 Homo sapiens cDNA clone TESTI4030502 5', mRNA sequence [DB083763] |
| A_33_P3216232 | 0.018537153 | -3.1110973 | ITGB1BP1 | Homo sapiens integrin beta 1 binding protein 1 (ITGB1BP1), transcript variant 1, mRNA [NM_004763] |
| A_23_P72697 | 0.010289684 | -2.9307806 | GPIHBP1 | Homo sapiens glycosylphosphatidylinositol anchored high density lipoprotein binding protein 1 (GPIHBP1), transcript variant 1, mRNA [NM_178172] |
| A_22_P00014063 | 0.006252873 | 3.799912 |  | NUKM_SOLTU (Q43844) NADH-ubiquinone oxidoreductase 20 kDa subunit, mitochondrial precursor (Complex I-20KD) (CI-20KD) , partial (6%) [THC2633760] |
| A_21_P0002976 | 0.017359223 | 2.0397716 | lnc-GPR27-2 | LNCipedia lincRNA (lnc-GPR27-2), lincRNA [lnc-GPR27-2:1] |
| A_33_P3400482 | 0.008533617 | 3.7767942 | LOC728158 | PREDICTED: Homo sapiens hCG2044975 (LOC728158), misc_RNA [XR_246201] |
| A_23_P142560 | 0.004554661 | -3.2135744 | ZEB2 | Homo sapiens zinc finger E-box binding homeobox 2 (ZEB2), transcript variant 1, mRNA [NM_014795] |
| A_33_P3295233 | 0.014613021 | 2.833802 |  | PREDICTED: Homo sapiens uncharacterized LOC284577 (RP11-576D8.4), misc_RNA [XR_250930] |
| A_21_P0001841 | 0.009904035 | 8.01305 |  | AGENCOURT_10399365 NIH_MGC_82 Homo sapiens cDNA clone IMAGE:6614454 5', mRNA sequence [BU567770] |
| A_22_P00018912 | 0.008821026 | 5.6941366 | LOC101927908 | PREDICTED: Homo sapiens uncharacterized LOC101927908 (LOC101927908), ncRNA [XR_245289] |
| A_21_P0013175 | 0.005823871 | 3.726424 | XLOC_l2_013293 | BROAD Institute lincRNA (XLOC_l2_013293), lincRNA [TCONS_l2_00025688] |
| A_24_P414658 | 0.003703089 | 3.2800138 | HIST1H2AG | histone cluster 1, H2ag [Source:HGNC Symbol;Acc:HGNC:4737] [ENST00000359193] |
| A_21_P0002291 | 0.003113939 | 3.524995 | lnc-RAB6C-4 | LNCipedia lincRNA (lnc-RAB6C-4), lincRNA [lnc-RAB6C-4:1] |
| A_33_P3244669 | 0.01865728 | -2.9759164 | TMEM30A | Homo sapiens transmembrane protein 30A (TMEM30A), transcript variant 1, mRNA [NM_018247] |
| A_21_P0008395 | 0.004017944 | 3.491153 | lnc-DIO3.1-1 | LNCipedia lincRNA (lnc-DIO3.1-1), lincRNA [lnc-DIO3.1-1:1] |
| A_21_P0013458 | 0.015963214 | 2.8787637 | LINC01287 | Homo sapiens long intergenic non-protein coding RNA 1287 (LINC01287), long non-coding RNA [NR_125776] |
| A_21_P0005265 | 0.008850385 | 6.3151546 |  |  |
| A_22_P00019675 | 0.003721795 | 4.845062 | lnc-ELP4-1 | LNCipedia lincRNA (lnc-ELP4-1), lincRNA [lnc-ELP4-1:1] |
| A_23_P361604 | 0.006839102 | 3.4813375 | C20orf78 | Homo sapiens chromosome 20 open reading frame 78 (C20orf78), transcript variant 1, mRNA [NM_001242671] |
| A_33_P3210620 | 0.013622328 | 2.1538885 | LOC100131497 | human full-length cDNA 5-PRIME end of clone CS0CAP002YE20 of Thymus of Homo sapiens (human). [BX248745] |
| A_24_P315014 | 0.019758487 | 2.2096484 | XAGE-4 | PREDICTED: Homo sapiens XAGE-4 protein (XAGE-4), misc_RNA [XR_430517] |
| A_33_P3290318 | 0.014878415 | 2.0052025 |  | Homo sapiens cDNA FLJ46117 fis, clone TESTI2037572. [AK127999] |
| A_21_P0010420 | 0.008421822 | 2.069874 | lnc-RP1-32I10.10.1-1 | LNCipedia lincRNA (lnc-RP1-32I10.10.1-1), lincRNA [lnc-RP1-32I10.10.1-1:1] |
| A_22_P00006586 | 0.008826038 | 3.0224614 |  |  |
| A_33_P3224780 | 0.004754042 | 2.120905 |  |  |
| A_21_P0007002 | 0.011139295 | 2.2240186 | lnc-RTKN2-3 | LNCipedia lincRNA (lnc-RTKN2-3), lincRNA [lnc-RTKN2-3:1] |
| A_19_P00810085 | 0.004663298 | 2.7052307 | LINC00607 | long intergenic non-protein coding RNA 607 [Source:HGNC Symbol;Acc:HGNC:43944] [ENST00000419922] |
| A_24_P232790 | 0.016339479 | 3.1118097 | CCDC177 | Homo sapiens coiled-coil domain containing 177 (CCDC177), mRNA [NM_001271507] |
| A_23_P94296 | 0.005767673 | 2.2157416 | ADAM7 | Homo sapiens ADAM metallopeptidase domain 7 (ADAM7), mRNA [NM_003817] |
| A_22_P00023709 | 0.00396329 | 3.690028 |  |  |
| A_33_P3285815 | 0.002131335 | 5.38125 | WFDC6 | WAP four-disulfide core domain 6 [Source:HGNC Symbol;Acc:HGNC:16164] [ENST00000372665] |
| A_22_P00021230 | 0.01613658 | 5.3498836 | IL36RN | interleukin 36 receptor antagonist [Source:HGNC Symbol;Acc:HGNC:15561] [ENST00000514072] |
| A_33_P3333787 | 0.0192649 | 2.082588 |  | Homo sapiens PRO2259 mRNA, complete cds. [AF132204] |
| A_21_P0002717 | 0.008887917 | 2.40883 | LOC101927123 | Homo sapiens uncharacterized LOC101927123 (LOC101927123), long non-coding RNA [NR_110147] |
| A_21_P0004254 | 0.004784878 | 2.1698341 | lnc-GPBP1-3 | LNCipedia lincRNA (lnc-GPBP1-3), lincRNA [lnc-GPBP1-3:2] |
| A_22_P00016701 | 0.001606751 | 3.5559282 |  | yt65b11.r1 Soares retina N2b4HR Homo sapiens cDNA clone IMAGE:275276 5', mRNA sequence [R84760] |
| A_21_P0005125 | 0.015121243 | 2.5340695 | lnc-KLHL32-4 | LNCipedia lincRNA (lnc-KLHL32-4), lincRNA [lnc-KLHL32-4:4] |
| A_22_P00013708 | 0.004429192 | 6.3194423 |  |  |
| A_21_P0001559 | 0.008024973 | 2.7252924 | lnc-OLFM3-1 | LNCipedia lincRNA (lnc-OLFM3-1), lincRNA [lnc-OLFM3-1:4] |
| A_21_P0001521 | 0.001719054 | 3.8910232 | lnc-ELTD1-4 | LNCipedia lincRNA (lnc-ELTD1-4), lincRNA [lnc-ELTD1-4:1] |
| A_22_P00005808 | 0.01601929 | 2.593102 |  |  |
| A_33_P3273394 | 3.38E-04 | 4.116177 | LOC101928423 | PREDICTED: Homo sapiens uncharacterized LOC101928423 (LOC101928423), ncRNA [XR_242343] |
| A_21_P0012920 | 0.017546525 | 2.38355 | XLOC_l2_012168 | BROAD Institute lincRNA (XLOC_l2_012168), lincRNA [TCONS_l2_00023043] |
| A_23_P70583 | 0.004994969 | -2.1974657 | VPS52 | Homo sapiens vacuolar protein sorting 52 homolog (S. cerevisiae) (VPS52), transcript variant 1, mRNA [NM_022553] |
| A_33_P3270332 | 0.006883169 | 3.5937545 | LOC100287792 | Homo sapiens uncharacterized LOC100287792 (LOC100287792), long non-coding RNA [NR_040021] |
| A_33_P3347049 | 0.016064143 | 2.4282176 |  |  |
| A_33_P3276303 | 0.005104526 | 2.9898825 | CES5AP1 | Homo sapiens carboxylesterase 5A pseudogene 1 (CES5AP1), non-coding RNA [NR_037839] |
| A_23_P78504 | 0.006028791 | 2.4196198 | USP29 | Homo sapiens ubiquitin specific peptidase 29 (USP29), mRNA [NM_020903] |
| A_21_P0007014 | 2.27E-04 | 3.9861844 | lnc-FGF8-1 | LNCipedia lincRNA (lnc-FGF8-1), lincRNA [lnc-FGF8-1:1] |
| A_22_P00019874 | 0.002315945 | 3.1626296 | lnc-NPY5R-4 | AL555340 Homo sapiens HELA CELLS COT 25-NORMALIZED Homo sapiens cDNA clone CS0DK008YC04 5-PRIME, mRNA sequence [AL555340] |
| A_21_P0006368 | 0.016074667 | 2.1233242 | lnc-TLR4-2 | LNCipedia lincRNA (lnc-TLR4-2), lincRNA [lnc-TLR4-2:1] |
| A_24_P128563 | 0.014443688 | -2.0889876 | KPNA6 | Homo sapiens karyopherin alpha 6 (importin alpha 7) (KPNA6), mRNA [NM_012316] |
| A_22_P00009177 | 0.005621383 | 2.2474945 | LOC102725393 |  |
| A_22_P00024118 | 0.002111073 | 5.0024076 |  |  |
| A_33_P3365870 | 0.007513454 | -2.2659814 | BSDC1 | Homo sapiens BSD domain containing 1 (BSDC1), transcript variant 1, mRNA [NM_001143888] |
| A_21_P0001299 | 0.001457917 | 3.4437392 | lnc-ACOT11-1 | LNCipedia lincRNA (lnc-ACOT11-1), lincRNA [lnc-ACOT11-1:1] |
| A_22_P00006339 | 0.007796347 | 2.7570226 | lnc-FANCL-1 | Homo sapiens cDNA clone IMAGE:4827713. [BC036673] |
| A_33_P3380702 | 0.002277691 | 2.514313 | OR5L1 | Homo sapiens olfactory receptor, family 5, subfamily L, member 1 (gene/pseudogene) (OR5L1), mRNA [NM_001004738] |
| A_21_P0012687 | 0.001574271 | 3.4167633 | XLOC_l2_011302 | BROAD Institute lincRNA (XLOC_l2_011302), lincRNA [TCONS_l2_00021337] |
| A_23_P354217 | 0.018311022 | 2.3175337 | TMEM151A | Homo sapiens transmembrane protein 151A (TMEM151A), mRNA [NM_153266] |
| A_32_P206104 | 0.00689303 | 2.5394177 | LOC101929541 | Homo sapiens uncharacterized LOC101929541 (LOC101929541), transcript variant 2, long non-coding RNA [NR_125983] |
| A_23_P71880 | 0.001729655 | 3.176846 | SPINK4 | Homo sapiens serine peptidase inhibitor, Kazal type 4 (SPINK4), mRNA [NM_014471] |
| A_21_P0004069 | 1.41E-04 | 4.3431277 |  |  |
| A_21_P0012445 | 0.011597462 | 2.5161252 | XLOC_l2_010239 | BROAD Institute lincRNA (XLOC_l2_010239), lincRNA [TCONS_l2_00019353] |
| A_21_P0003221 | 0.00858845 | 5.899728 | lnc-OXNAD1-2 | LNCipedia lincRNA (lnc-OXNAD1-2), lincRNA [lnc-OXNAD1-2:3] |
| A_33_P3244073 | 0.008468922 | 2.8630474 | LOC100132146 | Homo sapiens uncharacterized LOC100132146 (LOC100132146), mRNA [NM_001195442] |
| A_22_P00012365 | 9.80E-04 | 3.933577 |  | FIZ1_HUMAN (Q96SL8) Flt3-interacting zinc finger protein 1, partial (5%) [THC2671691] |
| A_22_P00003476 | 0.005147894 | 3.6924973 | lnc-CCDC71L-1 | LNCipedia lincRNA (lnc-CCDC71L-1), lincRNA [lnc-CCDC71L-1:2] |
| A_22_P00001205 | 0.01062803 | 2.9481518 | LINC01386 | Homo sapiens long intergenic non-protein coding RNA 1386 (LINC01386), long non-coding RNA [NR_126410] |
| A_23_P206293 | 0.002599517 | 3.1546001 | GPR114 | G protein-coupled receptor 114 [Source:HGNC Symbol;Acc:HGNC:19010] [ENST00000564607] |
| A_22_P00002794 | 0.004784878 | 3.155319 | lnc-C2orf27A-3 | LNCipedia lincRNA (lnc-C2orf27A-3), lincRNA [lnc-C2orf27A-3:1] |
| A_21_P0003128 | 0.002705118 | 2.4225845 | lnc-FOXP1-1 | LNCipedia lincRNA (lnc-FOXP1-1), lincRNA [lnc-FOXP1-1:1] |
| A_33_P3341269 | 0.014250958 | 2.184434 | lnc-LGALS14-1 | LNCipedia lincRNA (lnc-LGALS14-1), lincRNA [lnc-LGALS14-1:1] |
| A_33_P3368755 | 0.001128016 | 8.026604 | MAGEB6 | Homo sapiens melanoma antigen family B, 6 (MAGEB6), mRNA [NM_173523] |
| A_21_P0005385 | 0.001856124 | 2.6890452 | lnc-AC006455.1-5 | LNCipedia lincRNA (lnc-AC006455.1-5), lincRNA [lnc-AC006455.1-5:1] |
| A_21_P0007236 | 0.0072738 | 2.4147027 | lnc-P2RY6-1 | LNCipedia lincRNA (lnc-P2RY6-1), lincRNA [lnc-P2RY6-1:1] |
| A_23_P153930 | 0.014878415 | -2.035613 | ACVR2A | Homo sapiens activin A receptor, type IIA (ACVR2A), transcript variant 2, mRNA [NM_001616] |
| A_24_P160874 | 0.015378306 | -2.246837 | DUT | Homo sapiens deoxyuridine triphosphatase (DUT), transcript variant 1, mRNA [NM_001025248] |
| A_23_P252928 | 0.002338846 | 5.1025896 | MAGEA12 | Homo sapiens melanoma antigen family A, 12 (MAGEA12), transcript variant 3, mRNA [NM_005367] |
| A_21_P0006227 | 1.19E-05 | 3.2266953 | lnc-STXBP1-2 | LNCipedia lincRNA (lnc-STXBP1-2), lincRNA [lnc-STXBP1-2:1] |
| A_21_P0009877 | 0.019925974 | 3.3458292 | LINC01428 | Homo sapiens long intergenic non-protein coding RNA 1428 (LINC01428), long non-coding RNA [NR_110609] |
| A_22_P00007758 | 0.0028129 | 4.0115595 | lnc-HMHB1-1 | 602412948F1 NIH_MGC_92 Homo sapiens cDNA clone IMAGE:4521466 5', mRNA sequence [BG387923] |
| A_21_P0005005 | 0.018414054 | 2.3357434 | LOC102724520 | PREDICTED: Homo sapiens uncharacterized LOC102724520 (LOC102724520), transcript variant X1, ncRNA [XR_431974] |
| A_22_P00011730 | 0.01865728 | 3.2121198 |  |  |
| A_22_P00022540 | 0.002248436 | 3.0345507 |  |  |
| A_32_P479743 | 0.003703089 | 5.098672 | LINC00593 | Homo sapiens long intergenic non-protein coding RNA 593 (LINC00593), long non-coding RNA [NR_026764] |
| A_21_P0009204 | 0.006269174 | 2.4692628 | LOC101928766 | Homo sapiens uncharacterized LOC101928766 (LOC101928766), long non-coding RNA [NR_110850] |
| A_21_P0001440 | 0.002682729 | 5.3353305 | lnc-NTPCR-1 | LNCipedia lincRNA (lnc-NTPCR-1), lincRNA [lnc-NTPCR-1:1] |
| A_21_P0000673 | 0.012409855 | 3.1194818 | ZNF847P | Homo sapiens zinc finger protein 847, pseudogene (ZNF847P), non-coding RNA [NR_036753] |
| A_22_P00017046 | 0.011155876 | 2.2234209 | LOC102725286 | PREDICTED: Homo sapiens uncharacterized LOC102725286 (LOC102725286), ncRNA [XR_426321] |
| A_33_P3277970 | 0.007276182 | 2.6249561 |  | double homeobox 4 like 26 [Source:HGNC Symbol;Acc:HGNC:50807] [ENST00000489078] |
| A_23_P139786 | 0.009551783 | 2.4276655 | OASL | Homo sapiens 2'-5'-oligoadenylate synthetase-like (OASL), transcript variant 1, mRNA [NM_003733] |
| A_21_P0006531 | 0.01065715 | 3.2263038 | lnc-RPGR-1 | LNCipedia lincRNA (lnc-RPGR-1), lincRNA [lnc-RPGR-1:1] |
| A_23_P31414 | 0.013820666 | -2.6152444 | ORC5 | Homo sapiens origin recognition complex, subunit 5 (ORC5), transcript variant 1, mRNA [NM_002553] |
| A_21_P0008463 | 0.005637546 | 2.950835 | lnc-NPC2-1 | LNCipedia lincRNA (lnc-NPC2-1), lincRNA [lnc-NPC2-1:2] |
| A_21_P0012088 | 0.008007883 | 2.4884984 | LOC100506797 |  |
| A_33_P3386746 | 0.019925974 | 4.315509 | MALRD1 | Homo sapiens MAM and LDL receptor class A domain containing 1 (MALRD1), mRNA [NM_001142308] |
| A_22_P00003148 | 0.016641514 | 4.555661 | lnc-C9orf170-4 | DKFZp779F2057_r1 779 (synonym: hncc1) Homo sapiens cDNA clone DKFZp779F2057 5', mRNA sequence [BX500577] |
| A_22_P00012008 | 0.005593198 | 2.4004898 | lnc-PLCL2-1 | BX119265 Soares_NFL_T_GBC_S1 Homo sapiens cDNA clone IMAGp998E225154, mRNA sequence [BX119265] |
| A_22_P00002753 | 0.008755149 | 2.497239 | lnc-C21orf62-1 | LNCipedia lincRNA (lnc-C21orf62-1), lincRNA [lnc-C21orf62-1:1] |
| A_22_P00022119 | 0.016008036 | 2.071823 | lnc-KCTD3-1 | LNCipedia lincRNA (lnc-KCTD3-1), lincRNA [lnc-KCTD3-1:1] |
| A_33_P3283669 | 0.019216066 | 2.0123901 | ATP1A3 | Homo sapiens ATPase, Na+/K+ transporting, alpha 3 polypeptide (ATP1A3), transcript variant 3, mRNA [NM_001256214] |
| A_21_P0005221 | 0.013190938 | 3.3219986 | LOC100506725 | Homo sapiens uncharacterized LOC100506725 (LOC100506725), transcript variant 1, long non-coding RNA [NR_108082] |
| A_33_P3399593 | 0.003963198 | 2.256855 | CCDC134 | Homo sapiens coiled-coil domain containing 134 (CCDC134), mRNA [NM_024821] |
| A_19_P00322886 | 0.011545449 | -2.1823196 | lnc-SNURF-3 | Homo sapiens cDNA clone IMAGE:6577956. [BC080646] |
| A_22_P00006319 | 0.003060623 | 2.3959796 | lnc-FAM92B-3 | Q2TWX5_ASPOR (Q2TWX5) Predicted protein, partial (5%) [THC2652111] |
| A_24_P194670 | 0.006768369 | 2.5421338 | CST13P | Homo sapiens cystatin 13, pseudogene (CST13P), non-coding RNA [NR_001279] |
| A_33_P3256257 | 0.017953182 | 2.8517098 | KRTAP4-7 | Homo sapiens keratin associated protein 4-7 (KRTAP4-7), mRNA [NM_033061] |
| A_33_P3268275 | 0.002791597 | 4.734858 |  | SNRPN upstream reading frame-like, pseudogene [Source:HGNC Symbol;Acc:HGNC:29543] [ENST00000309296] |
| A_33_P3320418 | 0.011475068 | 4.3398476 |  |  |
| A_22_P00013285 | 0.01876734 | 2.3630702 | lnc-RP11-1085N6.3.1-1 | LNCipedia lincRNA (lnc-RP11-1085N6.3.1-1), lincRNA [lnc-RP11-1085N6.3.1-1:1] |
| A_24_P494943 | 0.00781545 | 2.843663 | LINC01531 | Homo sapiens long intergenic non-protein coding RNA 1531 (LINC01531), long non-coding RNA [NR_040046] |
| A_22_P00004273 | 0.010188723 | 2.6477516 | lnc-CNIH-1 | LNCipedia lincRNA (lnc-CNIH-1), lincRNA [lnc-CNIH-1:1] |
| A_23_P201711 | 0.012240742 | -2.6029863 | S100A6 | Homo sapiens S100 calcium binding protein A6 (S100A6), mRNA [NM_014624] |
| A_22_P00009774 | 3.17E-04 | 2.6867642 |  |  |
| A_22_P00010232 | 2.35E-05 | 6.4700947 |  |  |
| A_21_P0003826 | 0.002130372 | 2.190508 | lnc-S100P-1 | LNCipedia lincRNA (lnc-S100P-1), lincRNA [lnc-S100P-1:2] |
| A_22_P00017935 | 0.017024012 | 3.0447097 |  |  |
| A_23_P7560 | 0.004754042 | 3.787416 | IL12B | Homo sapiens interleukin 12B (IL12B), mRNA [NM_002187] |
| A_22_P00002140 | 0.017480576 | 3.1744106 | BPIFA4P | Homo sapiens BPI fold containing family A, member 4, pseudogene (BPIFA4P), non-coding RNA [NR_026760] |
| A_22_P00005482 | 0.009551783 | 3.6516976 |  |  |
| A_21_P0001413 | 0.008795916 | 3.2529783 | lnc-CAMK1G-5 | LNCipedia lincRNA (lnc-CAMK1G-5), lincRNA [lnc-CAMK1G-5:5] |
| A_21_P0012444 | 0.003831321 | 4.0619326 | LINC00877 | long intergenic non-protein coding RNA 877 [Source:HGNC Symbol;Acc:HGNC:27706] [ENST00000498432] |
| A_32_P188860 | 0.016502148 | -2.2258947 | IL17RD | Homo sapiens interleukin 17 receptor D (IL17RD), mRNA [NM_017563] |
| A_33_P3244753 | 0.019850597 | 2.2478676 | DRP2 | Homo sapiens dystrophin related protein 2 (DRP2), transcript variant 1, mRNA [NM_001939] |
| A_21_P0011130 | 0.004672971 | 6.4935904 | XLOC_l2_003647 | BROAD Institute lincRNA (XLOC_l2_003647), lincRNA [TCONS_l2_00006749] |
| A_22_P00005293 | 0.001494066 | 4.2529607 |  | DKFZp781J1237_r1 781 (synonym: hlcc4) Homo sapiens cDNA clone DKFZp781J1237 5', mRNA sequence [BX645074] |
| A_22_P00017799 | 0.012794371 | 3.6015866 | lnc-ZBTB10-3 | LNCipedia lincRNA (lnc-ZBTB10-3), lincRNA [lnc-ZBTB10-3:1] |
| A_21_P0011927 | 0.013622328 | 2.7202094 | LOC102724482 | PREDICTED: Homo sapiens uncharacterized LOC102724482 (LOC102724482), ncRNA [XR_425413] |
| A_33_P3249345 | 0.00631611 | 2.2331593 | PRAME | Homo sapiens preferentially expressed antigen in melanoma (PRAME), transcript variant 9, mRNA [NM_001291719] |
| A_33_P3372099 | 0.008821026 | -4.5679865 | DDIT4L | Homo sapiens DNA-damage-inducible transcript 4-like (DDIT4L), mRNA [NM_145244] |
| A_21_P0007663 | 0.010269631 | 4.8730536 | lnc-TMEM132C-6 | LNCipedia lincRNA (lnc-TMEM132C-6), lincRNA [lnc-TMEM132C-6:1] |
| A_22_P00002501 | 0.011419726 | 2.130809 | lnc-C16orf62-1 | LNCipedia lincRNA (lnc-C16orf62-1), lincRNA [lnc-C16orf62-1:1] |
| A_23_P38649 | 0.011901807 | 2.6629858 | MC2R | Homo sapiens melanocortin 2 receptor (adrenocorticotropic hormone) (MC2R), mRNA [NM_000529] |
| A_33_P3608620 | 0.004653566 | 4.498329 | LRIT3 | Homo sapiens leucine-rich repeat, immunoglobulin-like and transmembrane domains 3 (LRIT3), mRNA [NM_198506] |
| A_22_P00013363 | 0.004740476 | 4.4005194 |  | PREDICTED: Homo sapiens uncharacterized LOC101928779 (LOC101928779), ncRNA [XR_429177] |
| A_21_P0007819 | 0.015457029 | 5.817799 | lnc-CLEC2D-5 | ou35e09.x1 Soares_NFL_T_GBC_S1 Homo sapiens cDNA clone IMAGE:1628296 3', mRNA sequence [AI017879] |
| A_33_P3388651 | 0.011753339 | -2.1262593 | ABLIM1 | Homo sapiens actin binding LIM protein 1 (ABLIM1), transcript variant 3, mRNA [NM_001003408] |
| A_24_P83272 | 0.013803282 | 2.2351866 | MAGEB4 | Homo sapiens melanoma antigen family B, 4 (MAGEB4), mRNA [NM_002367] |
| A_22_P00018743 | 0.012978336 | 2.935339 |  |  |
| A_21_P0008183 | 0.008645061 | 2.5328557 | lnc-RNF219-1 | LNCipedia lincRNA (lnc-RNF219-1), lincRNA [lnc-RNF219-1:2] |
| A_21_P0007411 | 0.014601309 | 3.1047978 | lnc-RP11-831A10.1.1-1 | LNCipedia lincRNA (lnc-RP11-831A10.1.1-1), lincRNA [lnc-RP11-831A10.1.1-1:4] |
| A_23_P36882 | 0.00540034 | 2.0644708 | NTS | Homo sapiens neurotensin (NTS), mRNA [NM_006183] |
| A_22_P00008661 | 9.45E-04 | 4.259905 | lnc-KIAA0226L-4 | LNCipedia lincRNA (lnc-KIAA0226L-4), lincRNA [lnc-KIAA0226L-4:1] |
| A_24_P400604 | 0.017995344 | 6.649798 | RBMY1B | Homo sapiens RNA binding motif protein, Y-linked, family 1, member B (RBMY1B), mRNA [NM_001006121] |
| A_22_P00000925 | 0.01569922 | 2.4535513 |  |  |
| A_21_P0010771 | 0.005484189 | -2.5447457 | ANKRD20A2 | Homo sapiens ankyrin repeat domain 20 family, member A2 (ANKRD20A2), mRNA [NM_001012421] |
| A_21_P0004505 | 9.30E-04 | 2.438716 | lnc-C5orf64-1 | LNCipedia lincRNA (lnc-C5orf64-1), lincRNA [lnc-C5orf64-1:2] |
| A_21_P0010114 | 0.006416088 | 6.272852 | lnc-THBD-1 | LNCipedia lincRNA (lnc-THBD-1), lincRNA [lnc-THBD-1:1] |
| A_22_P00000249 | 0.01693474 | 3.101617 | LOC101927914 | Homo sapiens uncharacterized LOC101927914 (LOC101927914), long non-coding RNA [NR_110157] |
| A_33_P3294342 | 0.012357703 | 3.6533833 |  |  |
| A_21_P0013090 | 0.001978628 | 2.8279045 |  |  |
| A_22_P00024345 | 0.009671135 | 3.5193524 | LINC01269 | Homo sapiens long intergenic non-protein coding RNA 1269 (LINC01269), long non-coding RNA [NR_125769] |
| A_33_P3317925 | 0.010246585 | 4.184313 | KRTAP20-4 | Homo sapiens keratin associated protein 20-4 (KRTAP20-4), non-coding RNA [NR_023342] |
| A_33_P3246063 | 5.41E-04 | 2.3913157 |  |  |
| A_23_P46879 | 0.014131701 | 2.3132303 | PNLIP | Homo sapiens pancreatic lipase (PNLIP), mRNA [NM_000936] |
| A_21_P0013495 | 0.001699763 | 5.309656 | LINC01289 | Homo sapiens long intergenic non-protein coding RNA 1289 (LINC01289), long non-coding RNA [NR_038875] |
| A_21_P0005270 | 0.004234207 | 2.5103855 |  |  |
| A_23_P340822 | 0.005593198 | 3.465534 | CATSPERD | Homo sapiens catsper channel auxiliary subunit delta (CATSPERD), mRNA [NM_152784] |
| A_33_P3287078 | 0.012613835 | -2.2283258 | GZF1 | Homo sapiens GDNF-inducible zinc finger protein 1 (GZF1), mRNA [NM_022482] |
| A_22_P00021913 | 0.009476511 | 2.2953827 | LOC101929034 | Homo sapiens uncharacterized LOC101929034 (LOC101929034), long non-coding RNA [NR_104602] |
| A_22_P00012513 | 0.014494789 | 2.7930982 | LOC101927870 | Homo sapiens uncharacterized LOC101927870 (LOC101927870), long non-coding RNA [NR_110141] |
| A_22_P00004837 | 0.003602211 | 2.2918992 |  | DA430127 COLON2 Homo sapiens cDNA clone COLON2006333 5', mRNA sequence [DA430127] |
| A_23_P167599 | 0.013417468 | -2.4098964 | FAM134B | Homo sapiens family with sequence similarity 134, member B (FAM134B), transcript variant 1, mRNA [NM_001034850] |
| A_23_P3302 | 0.00724684 | -2.6614952 | MNS1 | Homo sapiens meiosis-specific nuclear structural 1 (MNS1), mRNA [NM_018365] |
| A_23_P201845 | 0.004165739 | -3.0023646 | ZMYM6 | Homo sapiens zinc finger, MYM-type 6 (ZMYM6), mRNA [NM_007167] |
| A_33_P3249274 | 0.007657446 | 2.6213882 | KRT73-AS1 | Homo sapiens KRT73 antisense RNA 1 (KRT73-AS1), long non-coding RNA [NR_126005] |
| A_22_P00006633 | 0.012711116 | 2.239443 | lnc-FOXD3-2 | LNCipedia lincRNA (lnc-FOXD3-2), lincRNA [lnc-FOXD3-2:1] |
| A_21_P0002238 | 0.014343364 | 4.8481717 | lnc-PLEK-1 | LNCipedia lincRNA (lnc-PLEK-1), lincRNA [lnc-PLEK-1:1] |
| A_33_P3308476 | 8.61E-04 | 4.8046665 | POM121L2 | Homo sapiens POM121 transmembrane nucleoporin-like 2 (POM121L2), mRNA [NM_033482] |
| A_22_P00005416 | 1.37E-04 | 3.489962 | LOC102723727 | PREDICTED: Homo sapiens uncharacterized LOC102723727 (LOC102723727), ncRNA [XR_426896] |
| A_24_P5890 | 0.018820966 | 3.3732939 | KIR2DL5A | Homo sapiens killer cell immunoglobulin-like receptor, two domains, long cytoplasmic tail, 5A (KIR2DL5A), mRNA [NM_020535] |
| A_21_P0008371 | 0.003703089 | 2.563462 | lnc-GPR65-1 | LNCipedia lincRNA (lnc-GPR65-1), lincRNA [lnc-GPR65-1:12] |
| A_23_P139028 | 0.002130372 | 2.8717732 | POU2F3 | Homo sapiens POU class 2 homeobox 3 (POU2F3), transcript variant 1, mRNA [NM_014352] |
| A_21_P0012408 | 0.01437141 | 2.0679991 |  |  |
| A_23_P165061 | 0.008900492 | -2.7639585 | AES | Homo sapiens amino-terminal enhancer of split (AES), transcript variant 1, mRNA [NM_198969] |
| A_21_P0011517 | 0.014342867 | 7.9546604 | KRT14 | Homo sapiens keratin 14, type I (KRT14), mRNA [NM_000526] |
| A_21_P0009082 | 0.014358267 | 3.338725 | lnc-AC092327.1-3 | LNCipedia lincRNA (lnc-AC092327.1-3), lincRNA [lnc-AC092327.1-3:1] |
| A_21_P0005274 | 0.0192649 | 4.8315887 | lnc-AC006372.1-1 | LNCipedia lincRNA (lnc-AC006372.1-1), lincRNA [lnc-AC006372.1-1:5] |
| A_22_P00012313 | 7.57E-04 | 4.0539865 | LOC101927346 | PREDICTED: Homo sapiens uncharacterized LOC101927346 (LOC101927346), ncRNA [XR_245076] |
| A_22_P00021582 | 0.016380092 | 3.1035917 | lnc-TMEM114-4 | DB038929 TESTI2 Homo sapiens cDNA clone TESTI2025323 5', mRNA sequence [DB038929] |
| A_21_P0009804 | 0.016781604 | 2.5662532 |  |  |
| A_23_P165921 | 9.39E-04 | 2.9254143 | LINC00029 | Homo sapiens long intergenic non-protein coding RNA 29 (LINC00029), long non-coding RNA [NR_028295] |
| A_23_P356694 | 2.27E-04 | 6.7038445 | DEFB123 | Homo sapiens defensin, beta 123 (DEFB123), transcript variant 1, mRNA [NM_153324] |
| A_33_P3413795 | 0.01630629 | 2.0341005 |  |  |
| A_21_P0011785 | 0.014443688 | 3.205574 | XLOC_l2_007167 | BROAD Institute lincRNA (XLOC_l2_007167), lincRNA [TCONS_l2_00013421] |
| A_22_P00013854 | 0.009085295 | 2.315265 | LOC101926926 | Homo sapiens uncharacterized LOC101926926 (LOC101926926), long non-coding RNA [NR_125388] |
| A_23_P139050 | 0.001976492 | 6.691933 | OR8D2 | Homo sapiens olfactory receptor, family 8, subfamily D, member 2 (gene/pseudogene) (OR8D2), mRNA [NM_001002918] |
| A_21_P0009904 | 5.08E-04 | 3.322038 | LINC01522 | Homo sapiens long intergenic non-protein coding RNA 1522 (LINC01522), long non-coding RNA [NR_110027] |
| A_23_P361569 | 0.006129033 | -3.55913 | SLC35F5 | Homo sapiens solute carrier family 35, member F5 (SLC35F5), transcript variant 1, mRNA [NM_025181] |
| A_21_P0011714 | 0.003240238 | 2.181968 | LILRA6 | Homo sapiens leukocyte immunoglobulin-like receptor, subfamily A (with TM domain), member 6 (LILRA6), transcript variant 1, mRNA [NM_024318] |
| A_32_P66020 | 0.012935037 | -2.6811707 | SNX29 | Homo sapiens sorting nexin 29 (SNX29), mRNA [NM_032167] |
| A_22_P00013543 | 0.007604716 | 4.698784 | lnc-RP11-429E11.3.1-1 | 601565938F1 NIH_MGC_21 Homo sapiens cDNA clone IMAGE:3840897 5', mRNA sequence [BE733583] |
| A_22_P00023845 | 0.002584939 | 4.656062 |  | BX113253 NCI_CGAP_GC6 Homo sapiens cDNA clone IMAGp998B045552, mRNA sequence [BX113253] |
| A_23_P74068 | 0.015893705 | 2.4084842 | MSH4 | Homo sapiens mutS homolog 4 (MSH4), mRNA [NM_002440] |
| A_33_P3327063 | 0.019070894 | 3.410497 | STRC | Homo sapiens stereocilin (STRC), mRNA [NM_153700] |
| A_33_P3422085 | 0.001648589 | 3.5594912 | SPANXN2 | Homo sapiens SPANX family, member N2 (SPANXN2), mRNA [NM_001009615] |
| A_23_P411157 | 0.015121243 | 5.721693 | WNT1 | Homo sapiens wingless-type MMTV integration site family, member 1 (WNT1), mRNA [NM_005430] |
| A_33_P3246413 | 0.019566411 | 4.68877 |  |  |
| A_33_P3310709 | 0.01375931 | 4.115268 |  | olfactory receptor, family 10, subfamily G, member 6 [Source:HGNC Symbol;Acc:HGNC:14836] [ENST00000307002] |
| A_24_P336848 | 0.012695524 | -2.0009346 | ACYP2 | Homo sapiens acylphosphatase 2, muscle type (ACYP2), mRNA [NM_138448] |
| A_21_P0005295 | 0.001849529 | 2.4807594 |  | DA846917 PLACE6 Homo sapiens cDNA clone PLACE6015523 5', mRNA sequence [DA846917] |
| A_22_P00014064 | 0.019099172 | 2.2454882 |  | Homo sapiens cDNA FLJ30849 fis, clone FEBRA2002882. [AK055411] |
| A_23_P116614 | 0.014342867 | -2.24201 | ME3 | Homo sapiens malic enzyme 3, NADP(+)-dependent, mitochondrial (ME3), transcript variant 2, mRNA [NM_001014811] |
| A_23_P99386 | 0.011724273 | 2.4773636 | TNFSF11 | Homo sapiens tumor necrosis factor (ligand) superfamily, member 11 (TNFSF11), transcript variant 1, mRNA [NM_003701] |
| A_21_P0003666 | 0.019034809 | 3.1062639 | lnc-FAM160A1-3 | LNCipedia lincRNA (lnc-FAM160A1-3), lincRNA [lnc-FAM160A1-3:1] |
| A_22_P00016296 | 0.012477387 | 2.2035756 |  | BX101435 Soares_testis_NHT Homo sapiens cDNA clone IMAGp998N063521, mRNA sequence [BX101435] |
| A_22_P00000562 | 0.007681231 | 2.1798427 | lnc-AC127496.3-6 | DKFZp686P2320_r1 686 (synonym: hlcc3) Homo sapiens cDNA clone DKFZp686P2320 5', mRNA sequence [AL603067] |
| A_23_P10542 | 0.003916526 | 2.8813677 | HTRA3 | Homo sapiens HtrA serine peptidase 3 (HTRA3), transcript variant 1, mRNA [NM_053044] |
| A_21_P0001979 | 0.012342428 | 2.3963242 |  | AV654273 GLC Homo sapiens cDNA clone GLCDUB02 3', mRNA sequence [AV654273] |
| A_21_P0009106 | 0.009892808 | 4.0689526 | lnc-MT1A-1 | LNCipedia lincRNA (lnc-MT1A-1), lincRNA [lnc-MT1A-1:1] |
| A_21_P0006809 | 0.004403577 | 3.7569568 |  |  |
| A_21_P0010912 | 0.019641617 | -2.172951 | XLOC_l2_002176 | BROAD Institute lincRNA (XLOC_l2_002176), lincRNA [TCONS_l2_00003853] |
| A_33_P3284315 | 0.018810572 | 2.0858243 |  | RST12623 Athersys RAGE Library Homo sapiens cDNA, mRNA sequence [BG193489] |
| A_22_P00020951 | 0.011555376 | 2.2231078 |  | long intergenic non-protein coding RNA 1259 [Source:HGNC Symbol;Acc:HGNC:49899] [ENST00000507056] |
| A_23_P90453 | 0.002277691 | 3.5079234 | KRTDAP | Homo sapiens keratinocyte differentiation-associated protein (KRTDAP), transcript variant 1, mRNA [NM_207392] |
| A_23_P94647 | 0.001604094 | 5.7024198 | OR1L3 | Homo sapiens olfactory receptor, family 1, subfamily L, member 3 (OR1L3), mRNA [NM_001005234] |
| A_22_P00022044 | 0.007181479 | 2.1590526 |  |  |
| A_22_P00023733 | 0.017540125 | 2.728327 | lnc-ILK-3 | LNCipedia lincRNA (lnc-ILK-3), lincRNA [lnc-ILK-3:1] |
| A_33_P3411296 | 0.015778573 | -2.1825564 | PURA | Homo sapiens purine-rich element binding protein A (PURA), mRNA [NM_005859] |
| A_33_P3352263 | 0.011445833 | 2.8704724 | OR5B3 | Homo sapiens olfactory receptor, family 5, subfamily B, member 3 (OR5B3), mRNA [NM_001005469] |
| A_33_P3251205 | 0.018612748 | 3.0290208 |  | immunoglobulin lambda variable 3-9 (gene/pseudogene) [Source:HGNC Symbol;Acc:HGNC:5918] [ENST00000390316] |
| A_23_P125639 | 0.012171941 | -2.4256742 | ZFX | Homo sapiens zinc finger protein, X-linked (ZFX), transcript variant 1, mRNA [NM_003410] |
| A_22_P00018353 | 0.006044566 | 3.372654 | lnc-ZSWIM2-1 | LNCipedia lincRNA (lnc-ZSWIM2-1), lincRNA [lnc-ZSWIM2-1:1] |
| A_33_P3317392 | 0.0103703 | 2.0467541 | ADAMTS19 | Homo sapiens ADAM metallopeptidase with thrombospondin type 1 motif, 19 (ADAMTS19), mRNA [NM_133638] |
| A_21_P0006895 | 0.002075288 | 2.230746 | lnc-PCGF5-1 | LNCipedia lincRNA (lnc-PCGF5-1), lincRNA [lnc-PCGF5-1:1] |
| A_21_P0001157 | 0.01682666 | 2.9365244 | LOC101928169 | PREDICTED: Homo sapiens uncharacterized LOC101928169 (LOC101928169), ncRNA [XR_246377] |
| A_21_P0013620 | 0.002486502 | 7.0750146 |  | coactivator-associated arginine methyltransferase 1 pseudogene 1 [Source:HGNC Symbol;Acc:HGNC:23392] [ENST00000497195] |
| A_24_P401491 | 0.012055238 | 4.689526 | MORN5 | Homo sapiens MORN repeat containing 5 (MORN5), transcript variant 1, mRNA [NM_198469] |
| A_21_P0003481 | 5.36E-04 | 2.5708704 |  |  |
| A_24_P127691 | 0.008980311 | -2.4637737 | DNAH14 | dynein, axonemal, heavy chain 14 [Source:HGNC Symbol;Acc:HGNC:2945] [ENST00000495456] |
| A_21_P0006031 | 0.010898792 | 5.0364227 | LOC101928014 |  |
| A_22_P00005795 | 0.009551783 | 3.354975 |  |  |
| A_22_P00003732 | 3.69E-04 | 4.1765656 |  |  |
| A_24_P191067 | 0.019903338 | -2.7934148 | CLSTN1 | Homo sapiens calsyntenin 1 (CLSTN1), transcript variant 1, mRNA [NM_001009566] |
| A_22_P00021899 | 0.010186528 | 2.1713858 |  | Q6NT14_HUMAN (Q6NT14) ZNF80 protein (Fragment), partial (5%) [THC2673678] |
| A_21_P0009610 | 0.005381616 | 2.3267334 | lnc-WDR7-2 | AGENCOURT_14276563 NIH_MGC_180 Homo sapiens cDNA clone IMAGE:30386298 5', mRNA sequence [CD359767] |
| A_21_P0006616 | 0.004160471 | 5.878707 | LINC00841 | Homo sapiens long intergenic non-protein coding RNA 841 (LINC00841), long non-coding RNA [NR_033846] |
| A_33_P3278571 | 0.008958424 | 3.7019482 | MAGIX | MAGI family member, X-linked [Source:HGNC Symbol;Acc:HGNC:30006] [ENST00000616812] |
| A_33_P3421695 | 0.009920881 | -2.269606 | CTNNB1 | Homo sapiens catenin (cadherin-associated protein), beta 1, 88kDa (CTNNB1), transcript variant 3, mRNA [NM_001098210] |
| A_22_P00025071 | 4.74E-04 | 2.7005033 | lnc-C7orf11-6 | LNCipedia lincRNA (lnc-C7orf11-6), lincRNA [lnc-C7orf11-6:1] |
| A_33_P3255587 | 0.012613835 | 4.1453013 | LINC00330 | Homo sapiens long intergenic non-protein coding RNA 330 (LINC00330), long non-coding RNA [NR_038433] |
| A_21_P0013296 | 0.004677045 | 2.0029182 | XLOC_l2_013783 | BROAD Institute lincRNA (XLOC_l2_013783), lincRNA [TCONS_l2_00026507] |
| A_23_P49376 | 0.005972175 | 3.3480048 | CETP | Homo sapiens cholesteryl ester transfer protein, plasma (CETP), transcript variant 1, mRNA [NM_000078] |
| A_21_P0007935 | 0.005252403 | 7.31089 | lnc-KIAA1704-2 | LNCipedia lincRNA (lnc-KIAA1704-2), lincRNA [lnc-KIAA1704-2:1] |
| A_21_P0012405 | 0.007681231 | 2.1950457 | XLOC_l2_009929 | BROAD Institute lincRNA (XLOC_l2_009929), lincRNA [TCONS_l2_00018911] |
| A_33_P3365978 | 0.019951195 | 2.427788 | ST7-AS2 | Homo sapiens ST7 antisense RNA 2 (ST7-AS2), transcript variant 2, long non-coding RNA [NR_002331] |
| A_33_P3337119 | 6.49E-04 | 3.275516 |  |  |
| A_24_P359030 | 0.009075529 | 4.29166 | LINC00167 | Homo sapiens long intergenic non-protein coding RNA 167 (LINC00167), long non-coding RNA [NR_024233] |
| A_23_P31858 | 0.001978732 | 4.0136404 | ST18 | Homo sapiens suppression of tumorigenicity 18, zinc finger (ST18), mRNA [NM_014682] |
| A_22_P00017320 | 0.006028791 | 2.153943 | LOC339803 | Homo sapiens uncharacterized LOC339803 (LOC339803), long non-coding RNA [NR_036496] |
| A_33_P3880302 | 0.008314215 | 2.0854537 | EPHB2 | Homo sapiens EPH receptor B2 (EPHB2), transcript variant 2, mRNA [NM_004442] |
| A_23_P204998 | 0.017311543 | -2.3119814 | FARP1 | Homo sapiens FERM, RhoGEF (ARHGEF) and pleckstrin domain protein 1 (chondrocyte-derived) (FARP1), transcript variant 1, mRNA [NM_005766] |
| A_23_P257503 | 0.013190938 | -2.3522682 | HERC1 | Homo sapiens HECT and RLD domain containing E3 ubiquitin protein ligase family member 1 (HERC1), mRNA [NM_003922] |
| A_21_P0008735 | 0.003867281 | 3.8913672 | LINC01491 | long intergenic non-protein coding RNA 1491 [Source:HGNC Symbol;Acc:HGNC:51148] [ENST00000561238] |
| A_33_P3232677 | 5.08E-04 | 7.701385 |  |  |
| A_22_P00001485 | 0.005720803 | 2.8006802 |  | HESC2_7_F07.g1_A035 NIH_MGC_258 Homo sapiens cDNA clone IMAGE:7467039 5', mRNA sequence [CX163669] |
| A_33_P3325429 | 0.001574271 | 3.0194986 |  | Homo sapiens mRNA for T cell receptor beta variable 5, partial cds, clone: un 66. [AB306153] |
| A_23_P26117 | 0.014307342 | -2.2821689 | MAN2C1 | Homo sapiens mannosidase, alpha, class 2C, member 1 (MAN2C1), transcript variant 1, mRNA [NM_006715] |
| A_22_P00006427 | 0.00447771 | 3.494501 | LOC101929181 | Homo sapiens uncharacterized LOC101929181 (LOC101929181), long non-coding RNA [NR_104624] |
| A_21_P0004527 | 0.019475961 | 2.396519 | lnc-TCF7-3 | LNCipedia lincRNA (lnc-TCF7-3), lincRNA [lnc-TCF7-3:1] |
| A_21_P0005428 | 0.012357703 | 3.0644138 | lnc-RP11-305M3.3.1-1 | LNCipedia lincRNA (lnc-RP11-305M3.3.1-1), lincRNA [lnc-RP11-305M3.3.1-1:1] |
| A_21_P0005465 | 0.013614208 | 2.285301 | lnc-AC091801.1.1-5 | LNCipedia lincRNA (lnc-AC091801.1.1-5), lincRNA [lnc-AC091801.1.1-5:3] |
| A_19_P00316528 | 0.007048865 | 2.5219996 |  |  |
| A_33_P3293778 | 0.005823871 | 2.1639974 | KRTAP10-4 | Homo sapiens keratin associated protein 10-4 (KRTAP10-4), mRNA [NM_198687] |
| A_21_P0006640 | 0.006577116 | 2.5397153 | lnc-PFKP-12 | BX106593 Soares_fetal_lung_NbHL19W Homo sapiens cDNA clone IMAGp998L23694, mRNA sequence [BX106593] |
| A_21_P0012805 | 0.019596959 | 5.3070655 | LINC01095 | Homo sapiens long intergenic non-protein coding RNA 1095 (LINC01095), long non-coding RNA [NR_038331] |
| A_24_P69691 | 0.012797289 | -2.0186183 | ZNF25 | Homo sapiens zinc finger protein 25 (ZNF25), mRNA [NM_145011] |
| A_21_P0010341 | 0.00641353 | 2.4806626 |  |  |
| A_22_P00014967 | 0.005720803 | 3.4593093 | lnc-SMAD6-1 | Homo sapiens cDNA FLJ38816 fis, clone LIVER2007568. [AK096135] |
| A_32_P11096 | 0.015522051 | 4.1082344 | ZCCHC13 | Homo sapiens zinc finger, CCHC domain containing 13 (ZCCHC13), mRNA [NM_203303] |
| A_23_P121945 | 0.0103703 | 2.3116047 | SNCB | Homo sapiens synuclein, beta (SNCB), transcript variant 1, mRNA [NM_001001502] |
| A_23_P80940 | 0.018294731 | 2.338401 | PPAT | Homo sapiens phosphoribosyl pyrophosphate amidotransferase (PPAT), mRNA [NM_002703] |
| A_23_P127697 | 0.017403776 | 2.8953257 |  | olfactory receptor, family 5, subfamily AQ, member 1 pseudogene [Source:HGNC Symbol;Acc:HGNC:15259] [ENST00000527596] |
| A_21_P0008474 | 0.007121976 | 5.1015787 | lnc-KCNK10-1 | LNCipedia lincRNA (lnc-KCNK10-1), lincRNA [lnc-KCNK10-1:1] |
| A_33_P3228402 | 0.013739725 | 4.3631926 |  |  |
| A_21_P0008752 | 0.005862902 | 2.4435706 | lnc-ITGA11-2 | HY006595 RIKEN full-length enriched human cDNA library, testis Homo sapiens cDNA clone H04D022M15, mRNA sequence [HY006595] |
| A_24_P817863 | 0.010417916 | 5.749637 | UNC80 | Homo sapiens unc-80 homolog (C. elegans) (UNC80), transcript variant 1, mRNA [NM_032504] |
| A_22_P00005072 | 0.006182891 | 3.3364694 | LOC102724196 | PREDICTED: Homo sapiens uncharacterized LOC102724196 (LOC102724196), ncRNA [XR_424327] |
| A_33_P3328352 | 0.002282318 | 7.8633533 | DEFB115 | Homo sapiens defensin, beta 115 (DEFB115), mRNA [NM_001037730] |
| A_22_P00019831 | 0.015756283 | 4.0570145 | LOC101927137 | Homo sapiens cDNA FLJ26070 fis, clone PRS09374. [AK129581] |
| A_21_P0007674 | 0.012840175 | 4.730633 | lnc-P2RX2-1 | LNCipedia lincRNA (lnc-P2RX2-1), lincRNA [lnc-P2RX2-1:1] |
| A_23_P67661 | 0.01777922 | -3.938868 | COX7A1 | cytochrome c oxidase subunit VIIa polypeptide 1 (muscle) [Source:HGNC Symbol;Acc:HGNC:2287] [ENST00000292907] |
| A_33_P3217322 | 0.002705118 | 3.5682218 | CFAP46 | Homo sapiens cilia and flagella associated protein 46 (CFAP46), mRNA [NM_001200049] |
| A_33_P3287760 | 0.00540034 | 2.5537813 | RASGEF1A | Homo sapiens RasGEF domain family, member 1A (RASGEF1A), transcript variant 1, mRNA [NM_001282862] |
| A_33_P3255706 | 0.013712578 | 2.7750804 | OR6K6 | Homo sapiens olfactory receptor, family 6, subfamily K, member 6 (OR6K6), mRNA [NM_001005184] |
| A_22_P00017349 | 0.010270238 | -2.0070326 | lnc-USP8-1 | LNCipedia lincRNA (lnc-USP8-1), lincRNA [lnc-USP8-1:1] |
| A_22_P00008561 | 0.001092771 | -2.5651343 | KCNN3 | Homo sapiens potassium channel, calcium activated intermediate/small conductance subfamily N alpha, member 3 (KCNN3), transcript variant 3, mRNA [NM_001204087] |
| A_22_P00004822 | 0.013553805 | 2.057531 | lnc-CXorf56-2 | K-EST0019353 S6SNU620 Homo sapiens cDNA clone S6SNU620-19-G02 5', mRNA sequence [BM745483] |
| A_22_P00018933 | 0.001457917 | 3.00017 |  |  |
| A_24_P145019 | 0.006177985 | 3.0836542 | SCOC-AS1 | Homo sapiens SCOC antisense RNA 1 (SCOC-AS1), long non-coding RNA [NR_033939] |
| A_32_P5568 | 0.016114652 | 2.232465 | LOC101929154 | Homo sapiens uncharacterized LOC101929154 (LOC101929154), transcript variant 1, long non-coding RNA [NR_105012] |
| A_21_P0001981 | 0.004280046 | 5.3337092 |  |  |
| A_21_P0002441 | 2.62E-04 | 2.3775876 | lnc-C2orf63-1 | LNCipedia lincRNA (lnc-C2orf63-1), lincRNA [lnc-C2orf63-1:1] |
| A_33_P3277527 | 0.014648372 | -2.3199658 | LAMC3 | Homo sapiens laminin, gamma 3 (LAMC3), mRNA [NM_006059] |
| A_22_P00001390 | 0.017040258 | 3.398969 | lnc-AP3S1-6 | Homo sapiens tripartite motif-containing 36, mRNA (cDNA clone IMAGE:5015503). [BC017346] |
| A_21_P0012614 | 0.002777084 | 4.1147623 |  |  |
| A_21_P0005081 | 0.014837787 | 2.8846948 | lnc-AL078585.1-3 | LNCipedia lincRNA (lnc-AL078585.1-3), lincRNA [lnc-AL078585.1-3:1] |
| A_22_P00006973 | 0.01865728 | 2.3334584 |  |  |
| A_23_P114008 | 0.005339755 | 4.184538 | TM4SF20 | Homo sapiens transmembrane 4 L six family member 20 (TM4SF20), mRNA [NM_024795] |
| A_22_P00007250 | 0.002546426 | 3.5917506 | LINC01257 | Homo sapiens long intergenic non-protein coding RNA 1257 (LINC01257), long non-coding RNA [NR_026670] |
| A_23_P314789 | 0.01908123 | 2.9925964 | ZDHHC19 | Homo sapiens zinc finger, DHHC-type containing 19 (ZDHHC19), mRNA [NM_001039617] |
| A_33_P3463924 | 0.00540034 | 3.6318183 | LOC57399 | Homo sapiens uncharacterized gastric protein ZA52P mRNA, complete cds. [AF264626] |
| A_33_P3237507 | 0.006659397 | 2.9031575 | C4orf50 | chromosome 4 open reading frame 50 [Source:HGNC Symbol;Acc:HGNC:33766] [ENST00000531445] |
| A_21_P0001383 | 0.00858845 | 2.1533804 | lnc-TBX19-1 | LNCipedia lincRNA (lnc-TBX19-1), lincRNA [lnc-TBX19-1:1] |
| A_22_P00003518 | 0.007157139 | 5.36699 |  | Homo sapiens cDNA clone IMAGE:5271685. [BC039379] |
| A_21_P0005072 | 0.002472808 | 4.3032136 | lnc-NOX3-2 | LNCipedia lincRNA (lnc-NOX3-2), lincRNA [lnc-NOX3-2:1] |
| A_32_P62090 | 0.007173597 | 2.7948208 | CCDC182 | Homo sapiens coiled-coil domain containing 182 (CCDC182), mRNA [NM_001282544] |
| A_22_P00007788 | 0.007582563 | 3.2447853 | lnc-HNRNPH1-2 | qf32a12.x1 Soares_testis_NHT Homo sapiens cDNA clone IMAGE:1751710 3', mRNA sequence [AI149719] |
| A_21_P0004890 | 0.001113415 | 5.4098954 | LOC102724327 | PREDICTED: Homo sapiens uncharacterized LOC102724327 (LOC102724327), ncRNA [XR_427954] |
| A_22_P00015419 | 8.61E-04 | 3.0367715 |  |  |
| A_33_P3275893 | 3.35E-04 | 3.6119444 | LOC644070 | PREDICTED: Homo sapiens germ cell-specific gene 1-like protein 2-like (LOC644070), mRNA [XM_006710028] |
| A_33_P3257486 | 0.015121243 | 2.8244338 | IGLL1/IGLL5 (nome IPA) | immunoglobulin lambda variable 5-45 [Source:HGNC Symbol;Acc:HGNC:5924] [ENST00000390296] |
| A_33_P3404686 | 9.39E-04 | 5.566323 | TNK2-AS1 | Homo sapiens cDNA FLJ36796 fis, clone ADRGL2006817. [AK094115] |
| A_21_P0009944 | 0.01865728 | 3.1447558 | lnc-SSTR4-6 | LNCipedia lincRNA (lnc-SSTR4-6), lincRNA [lnc-SSTR4-6:1] |
| A_33_P3291510 | 0.015347909 | 2.2555506 | VCY | Homo sapiens variable charge, Y-linked (VCY), mRNA [NM_004679] |
| A_19_P00321398 | 0.013368452 | 2.3773208 |  |  |
| A_24_P795371 | 0.008649809 | -2.2551458 | NR2F2-AS1 | Homo sapiens NR2F2 antisense RNA 1 (NR2F2-AS1), transcript variant 1, long non-coding RNA [NR_102743] |
| A_33_P3776564 | 0.002705118 | 3.9840112 | LOC158434 | Homo sapiens uncharacterized LOC158434 (LOC158434), mRNA [NM_001256408] |
| A_22_P00013572 | 0.007157139 | 3.1113603 | LINC01568 | Homo sapiens long intergenic non-protein coding RNA 1568 (LINC01568), long non-coding RNA [NR_038234] |
| A_33_P3278013 | 0.008252968 | 2.0748236 | CD2 | CD2 molecule [Source:HGNC Symbol;Acc:HGNC:1639] [ENST00000369477] |
| A_21_P0006455 | 0.001327573 | 16.54398 | PABPC1L2B-AS1 | Homo sapiens PABPC1L2B antisense RNA 1 (head to head) (PABPC1L2B-AS1), long non-coding RNA [NR_110398] |
| A_22_P00023575 | 0.008953173 | 4.3109946 |  | DA576409 HHDPC2 Homo sapiens cDNA clone HHDPC2003849 5', mRNA sequence [DA576409] |
| A_22_P00017801 | 0.007794031 | 2.3188376 | LOC102723831 | Homo sapiens uncharacterized LOC102723831 (LOC102723831), long non-coding RNA [NR_125868] |
| A_24_P193498 | 0.008821026 | -2.5663261 | TM2D3 | Homo sapiens TM2 domain containing 3 (TM2D3), transcript variant 1, mRNA [NM_078474] |
| A_22_P00003121 | 0.012877549 | 2.3392873 |  | 603025214F1 NIH_MGC_114 Homo sapiens cDNA clone IMAGE:5195666 5', mRNA sequence [BI754780] |
| A_21_P0011126 | 0.017750079 | -2.596389 | NUS1 | Homo sapiens nuclear undecaprenyl pyrophosphate synthase 1 homolog (S. cerevisiae) (NUS1), mRNA [NM_138459] |
| A_33_P3383891 | 0.01303759 | 2.722203 |  | Homo sapiens cDNA FLJ45994 fis, clone SKMUS2009557. [AK127888] |
| A_33_P3237905 | 0.003243483 | 3.0551364 |  |  |
| A_21_P0002201 | 0.019216895 | 3.2462378 | lnc-GALM-2 | LNCipedia lincRNA (lnc-GALM-2), lincRNA [lnc-GALM-2:1] |
| A_21_P0009068 | 3.38E-04 | 4.234366 | lnc-CDYL2-5 | LNCipedia lincRNA (lnc-CDYL2-5), lincRNA [lnc-CDYL2-5:1] |
| A_21_P0003843 | 0.01306392 | 3.091811 |  |  |
| A_24_P281036 | 0.016639534 | 3.0744517 | LRRC31 | Homo sapiens leucine rich repeat containing 31 (LRRC31), transcript variant 1, mRNA [NM_024727] |
| A_24_P266728 | 0.008809855 | -2.3190281 | SF1 | Homo sapiens splicing factor 1 (SF1), transcript variant 1, mRNA [NM_004630] |
| A_21_P0006892 | 0.017797079 | 2.105183 | lnc-KIF20B-4 | LNCipedia lincRNA (lnc-KIF20B-4), lincRNA [lnc-KIF20B-4:2] |
| A_21_P0009978 | 0.002563245 | 4.7017365 | lnc-MC3R-3 | LNCipedia lincRNA (lnc-MC3R-3), lincRNA [lnc-MC3R-3:1] |
| A_21_P0004939 | 3.47E-04 | 4.024296 | lnc-LAMA2-1 | LNCipedia lincRNA (lnc-LAMA2-1), lincRNA [lnc-LAMA2-1:1] |
| A_22_P00017870 | 0.006368832 | 3.671651 | lnc-ZDHHC6-1 | LNCipedia lincRNA (lnc-ZDHHC6-1), lincRNA [lnc-ZDHHC6-1:1] |
| A_21_P0012516 | 0.006343113 | 4.7638955 | LINC01206 | Homo sapiens long intergenic non-protein coding RNA 1206 (LINC01206), long non-coding RNA [NR_104146] |
| A_23_P154832 | 0.013692818 | -3.2394965 | ATP5J | Homo sapiens ATP synthase, H+ transporting, mitochondrial Fo complex, subunit F6 (ATP5J), transcript variant 1, mRNA [NM_001003703] |
| A_22_P00020084 | 0.017609984 | 2.3731265 | lnc-MARCKS-2 | LNCipedia lincRNA (lnc-MARCKS-2), lincRNA [lnc-MARCKS-2:1] |
| A_22_P00014895 | 0.012371562 | 2.643701 | lnc-SLC7A6-1 | ALU7_HUMAN (P39194) Alu subfamily SQ sequence contamination warning entry, partial (6%) [THC2723783] |
| A_21_P0008987 | 0.002183284 | 3.8899527 | lnc-FOXF1-1 | LNCipedia lincRNA (lnc-FOXF1-1), lincRNA [lnc-FOXF1-1:1] |
| A_24_P195134 | 0.005459908 | 6.151669 |  | nuclear cap binding protein subunit 2-like [Source:HGNC Symbol;Acc:HGNC:31795] [ENST00000509000] |
| A_22_P00010419 | 0.014844837 | 2.1571295 | lnc-MYOF-1 | LNCipedia lincRNA (lnc-MYOF-1), lincRNA [lnc-MYOF-1:1] |
| A_22_P00007661 | 0.01601842 | 2.5189323 | LOC643623 | Homo sapiens uncharacterized LOC643623 (LOC643623), long non-coding RNA [NR_038906] |
| A_32_P213349 | 0.004234207 | 3.3958578 | lnc-DPP4-1 | Q56A81_HUMAN (Q56A81) TBR1 protein (Fragment), partial (10%) [THC2691455] |
| A_22_P00002378 | 0.008252968 | 3.5530188 |  |  |
| A_21_P0005249 | 0.002770355 | 2.6963239 | lnc-ASB4-3 | LNCipedia lincRNA (lnc-ASB4-3), lincRNA [lnc-ASB4-3:4] |
| A_21_P0005915 | 0.01037101 | 2.0392587 | lnc-SLC45A4-1 | LNCipedia lincRNA (lnc-SLC45A4-1), lincRNA [lnc-SLC45A4-1:1] |
| A_22_P00008414 | 0.016339479 | 3.121236 |  |  |
| A_33_P3290959 | 0.003209651 | 3.1332214 | lnc-POLR2B-1 | HUMMAC25X {Homo sapiens} (exp=-1; wgp=0; cg=0), partial (12%) [THC2614138] |
| A_22_P00014638 | 0.010246585 | 2.3235748 |  |  |
| A_22_P00011664 | 0.002130372 | 3.4510372 | lnc-PDE6A-1 | LNCipedia lincRNA (lnc-PDE6A-1), lincRNA [lnc-PDE6A-1:1] |
| A_22_P00017281 | 0.003198486 | 7.72638 | lnc-USO1-1 | LNCipedia lincRNA (lnc-USO1-1), lincRNA [lnc-USO1-1:1] |
| A_22_P00017771 | 0.001860961 | 2.3461707 |  | Homo sapiens cDNA FLJ37257 fis, clone BRAMY2010171. [AK094576] |
| A_23_P116249 | 0.002130372 | 2.3799138 | GRIK4 | Homo sapiens glutamate receptor, ionotropic, kainate 4 (GRIK4), transcript variant 2, mRNA [NM_014619] |
| A_21_P0011662 | 0.011555376 | 2.6362 | LINC00669 | long intergenic non-protein coding RNA 669 [Source:HGNC Symbol;Acc:HGNC:44332] [ENST00000591469] |
| A_33_P3287883 | 0.015845079 | 2.090987 | LOC100133331 | Homo sapiens uncharacterized LOC100133331 (LOC100133331), long non-coding RNA [NR_028327] |
| A_22_P00019904 | 0.002822993 | 5.113411 | lnc-ISLR-1 | Homo sapiens cDNA FLJ43811 fis, clone TESTI4001201. [AK125799] |
| A_33_P3413388 | 0.004403577 | 2.994384 |  | GB |
| A_33_P3272184 | 0.012461365 | 2.4240763 | LOC100131532 | Homo sapiens uncharacterized LOC100131532 (LOC100131532), long non-coding RNA [NR_027434] |
| A_24_P349196 | 0.014642934 | -2.234961 | CCDC30 | Homo sapiens coiled-coil domain containing 30 (CCDC30), mRNA [NM_001080850] |
| A_22_P00013869 | 0.0157653 | 2.4965334 |  |  |
| A_22_P00002241 | 0.002979886 | 2.3632698 |  | DB068425 TESTI4 Homo sapiens cDNA clone TESTI4010351 5', mRNA sequence [DB068425] |
| A_22_P00023645 | 0.001854862 | 7.1356974 |  |  |
| A_24_P108262 | 0.011555376 | 2.714468 | SDK2 | Homo sapiens sidekick cell adhesion molecule 2 (SDK2), mRNA [NM_001144952] |
| A_22_P00025812 | 0.002144959 | 2.6584835 | ELMO1-AS1 | Homo sapiens ELMO1 antisense RNA 1 (ELMO1-AS1), long non-coding RNA [NR_104120] |
| A_24_P932305 | 0.012374448 | 2.0621474 | QIQN5815 | Homo sapiens clone DNA129580 QIQN5815 (UNQ5815) mRNA, complete cds. [AY358807] |
| A_24_P116669 | 0.006565484 | 2.1462762 | CANT1 | Homo sapiens calcium activated nucleotidase 1 (CANT1), transcript variant 1, mRNA [NM_138793] |
| A_22_P00012902 | 0.001162504 | 2.8978472 |  | DB090595 TESTI4 Homo sapiens cDNA clone TESTI4039597 5', mRNA sequence [DB090595] |
| A_21_P0009574 | 0.00683727 | 2.1267226 |  | Homo sapiens cDNA clone IMAGE:4825594. [BC047643] |
| A_23_P101093 | 0.01601842 | -2.4314756 | COPZ2 | Homo sapiens coatomer protein complex, subunit zeta 2 (COPZ2), mRNA [NM_016429] |
| A_21_P0006812 | 9.80E-04 | 2.0667295 |  |  |
| A_21_P0013053 | 0.015241301 | 2.2843413 | XLOC_l2_012844 | BROAD Institute lincRNA (XLOC_l2_012844), lincRNA [TCONS_l2_00024560] |
| A_22_P00005302 | 0.006850744 | 4.4729543 | lnc-DNAJC11-2 | LNCipedia lincRNA (lnc-DNAJC11-2), lincRNA [lnc-DNAJC11-2:1] |
| A_23_P108564 | 0.003097098 | 2.5633914 | B3GALT1 | Homo sapiens UDP-Gal:betaGlcNAc beta 1,3-galactosyltransferase, polypeptide 1 (B3GALT1), mRNA [NM_020981] |
| A_33_P3262074 | 0.004403577 | 4.4919696 |  |  |
| A_22_P00000788 | 0.003660631 | 5.375774 |  |  |
| A_33_P3665749 | 0.014492409 | 2.9173446 | LINC00906 | Homo sapiens long intergenic non-protein coding RNA 906 (LINC00906), long non-coding RNA [NR_027318] |
| A_24_P220472 | 0.016327148 | 2.6500695 | SPATA12 | Homo sapiens spermatogenesis associated 12 (SPATA12), mRNA [NM_181727] |
| A_24_P818529 | 0.011796018 | -2.0910423 |  | ribonuclease H2, subunit C pseudogene 1 [Source:HGNC Symbol;Acc:HGNC:24117] [ENST00000454281] |
| A_21_P0012518 | 0.01693474 | 3.0082552 | LINC01206 | long intergenic non-protein coding RNA 1206 [Source:HGNC Symbol;Acc:HGNC:49637] [ENST00000476815] |
| A_21_P0008470 | 0.016431227 | 4.362093 | lnc-SEL1L-9 | LNCipedia lincRNA (lnc-SEL1L-9), lincRNA [lnc-SEL1L-9:1] |
| A_22_P00007742 | 0.017965812 | 2.2100263 | LINGO1-AS1 | Homo sapiens LINGO1 antisense RNA 1 (LINGO1-AS1), long non-coding RNA [NR_045123] |
| A_21_P0002574 | 0.010246585 | 3.7325773 | MYCNUT | Homo sapiens MYCN upstream transcript (non-protein coding) (MYCNUT), long non-coding RNA [NR_125783] |
| A_21_P0011854 | 0.01601929 | 2.0629113 |  | zinc finger protein 285 [Source:HGNC Symbol;Acc:HGNC:13079] [ENST00000614994] |
| A_32_P10936 | 0.006494935 | 3.7496035 | CDH12 | Homo sapiens cadherin 12, type 2 (N-cadherin 2) (CDH12), mRNA [NM_004061] |
| A_33_P3269636 | 0.01620116 | 2.5985546 | SBSN | Homo sapiens suprabasin (SBSN), transcript variant 1, mRNA [NM_001166034] |
| A_21_P0013224 | 0.002591965 | 3.161531 | XLOC_l2_013458 | BROAD Institute lincRNA (XLOC_l2_013458), lincRNA [TCONS_l2_00025960] |
| A_33_P3397180 | 0.007796347 | 2.2810464 | LOC100129324 | Homo sapiens cDNA FLJ46397 fis, clone THYMU3003958. [AK128261] |
| A_22_P00012286 | 0.012846116 | 2.2758172 | LOC101930611 | PREDICTED: Homo sapiens uncharacterized LOC101930611 (LOC101930611), ncRNA [XR_250546] |
| A_22_P00021828 | 0.006777925 | 3.3797567 |  |  |
| A_22_P00009957 | 0.016946392 | 2.5747573 |  | Q8IVU6_HUMAN (Q8IVU6) TMEM76 protein (Fragment), partial (8%) [THC2770369] |
| A_23_P303238 | 0.008019581 | 6.260737 | VN1R5 | Homo sapiens vomeronasal 1 receptor 5 (gene/pseudogene) (VN1R5), mRNA [NM_173858] |
| A_33_P3304170 | 0.01793104 | 2.5008557 | PIK3CG | Homo sapiens phosphatidylinositol-4,5-bisphosphate 3-kinase, catalytic subunit gamma (PIK3CG), transcript variant 1, mRNA [NM_002649] |
| A_22_P00018754 | 0.01986209 | 2.0321536 |  | DB066200 TESTI4 Homo sapiens cDNA clone TESTI4007460 5', mRNA sequence [DB066200] |
| A_32_P55840 | 0.010367587 | 2.1476362 | LINC01556 | Homo sapiens long intergenic non-protein coding RNA 1556 (LINC01556), long non-coding RNA [NR_103538] |
| A_21_P0000026 | 0.01693474 | 2.4464576 | C15orf40 | Homo sapiens chromosome 15 open reading frame 40 (C15orf40), transcript variant 4, mRNA [NM_001160115] |
| A_21_P0010301 | 0.002791597 | 4.682396 | lnc-TSPEAR-1 | LNCipedia lincRNA (lnc-TSPEAR-1), lincRNA [lnc-TSPEAR-1:2] |
| A_23_P430670 | 0.01693474 | 2.1319933 | CHST5 | Homo sapiens carbohydrate (N-acetylglucosamine 6-O) sulfotransferase 5 (CHST5), mRNA [NM_024533] |
| A_23_P141730 | 0.01928657 | -2.865581 | DSG2 | Homo sapiens desmoglein 2 (DSG2), mRNA [NM_001943] |
| A_22_P00010609 | 0.016114652 | 2.7673569 |  | TTLL11 intronic transcript 1 (non-protein coding) [Source:HGNC Symbol;Acc:HGNC:24214] [ENST00000411790] |
| A_22_P00000975 | 0.010546024 | -2.5596042 | lnc-AL117340.1-1 | LNCipedia lincRNA (lnc-AL117340.1-1), lincRNA [lnc-AL117340.1-1:1] |
| A_22_P00003114 | 0.017209308 | 2.8899727 | lnc-C9orf104-2 | wf48b12.x1 Soares_NFL_T_GBC_S1 Homo sapiens cDNA clone IMAGE:2358815 3', mRNA sequence [AI807473] |
| A_21_P0013472 | 0.002811484 | 2.2671895 | XLOC_l2_014182 | BROAD Institute lincRNA (XLOC_l2_014182), lincRNA [TCONS_l2_00027727] |
| A_21_P0006195 | 0.016838867 | 2.9846332 | lnc-C9orf107-2 | LNCipedia lincRNA (lnc-C9orf107-2), lincRNA [lnc-C9orf107-2:1] |
| A_23_P58407 | 0.002062888 | 3.2471027 | UGT2B15 | Homo sapiens UDP glucuronosyltransferase 2 family, polypeptide B15 (UGT2B15), mRNA [NM_001076] |
| A_33_P3792370 | 0.002024585 | 3.930362 | LINC00620 | Homo sapiens long intergenic non-protein coding RNA 620 (LINC00620), long non-coding RNA [NR_027103] |
| A_21_P0007668 | 2.71E-04 | 6.2530866 | lnc-FZD10-3 | LNCipedia lincRNA (lnc-FZD10-3), lincRNA [lnc-FZD10-3:1] |
| A_21_P0002150 | 7.61E-05 | 3.937302 |  |  |
| A_24_P357037 | 0.016939905 | -2.0557587 | UBE2G2 | Homo sapiens ubiquitin-conjugating enzyme E2G 2 (UBE2G2), transcript variant 2, mRNA [NM_182688] |
| A_22_P00007151 | 0.014822709 | 2.2482014 | SLC38A3 | Homo sapiens solute carrier family 38, member 3 (SLC38A3), mRNA [NM_006841] |
| A_22_P00025895 | 0.015536374 | 2.2761595 | lnc-AGPS-3 | Q9HB46_HUMAN (Q9HB46) CAMP/cGMP phosphodiesterase 11A2, partial (5%) [THC2658861] |
| A_21_P0013426 | 0.001728022 | 2.9501748 | LOC650226 | Homo sapiens ankyrin repeat domain 26 pseudogene (LOC650226), non-coding RNA [NR_029420] |
| A_23_P128855 | 3.17E-04 | 3.5477393 | SLC39A2 | Homo sapiens solute carrier family 39 (zinc transporter), member 2 (SLC39A2), transcript variant 1, mRNA [NM_014579] |
| A_22_P00003697 | 0.01693474 | 2.1503737 | LINC00922 | Homo sapiens long intergenic non-protein coding RNA 922 (LINC00922), long non-coding RNA [NR_027755] |
| A_33_P3345673 | 0.01865728 | 2.2523565 | MED15P9 | Homo sapiens hypothetical protein LOC285103, mRNA (cDNA clone IMAGE:5273139). [BC036597] |
| A_22_P00003663 | 0.00196988 | 6.011235 | lnc-CDC40-1 | Homo sapiens, clone IMAGE:5575984, mRNA. [BC035649] |
| A_33_P3411021 | 0.004917577 | 5.364807 | SLIT1 | Homo sapiens slit homolog 1 (Drosophila), mRNA (cDNA clone IMAGE:5247200), with apparent retained intron. [BC028105] |
| A_22_P00000458 | 0.011516959 | 2.2626376 | LINC01265 | Homo sapiens long intergenic non-protein coding RNA 1265 (LINC01265), long non-coding RNA [NR_104631] |
| A_33_P3382281 | 0.01437141 | 2.6171362 | SLX4IP | SLX4 interacting protein [Source:HGNC Symbol;Acc:HGNC:16225] [ENST00000488816] |
| A_33_P3413945 | 0.002278488 | 3.6005037 |  |  |
| A_21_P0010102 | 0.012042754 | 2.272799 | lnc-PPDPF-1 | LNCipedia lincRNA (lnc-PPDPF-1), lincRNA [lnc-PPDPF-1:5] |
| A_22_P00025017 | 0.004017944 | 5.3683357 | LOC101929535 | PREDICTED: Homo sapiens uncharacterized LOC101929535 (LOC101929535), ncRNA [XR_242017] |
| A_21_P0002642 | 0.00208499 | 6.1129646 | LINC01248 | Homo sapiens long intergenic non-protein coding RNA 1248 (LINC01248), long non-coding RNA [NR_110580] |
| A_21_P0012474 | 0.00635109 | 2.3474352 |  | HESC4_78_A05.g1_A037 NIH_MGC_262 Homo sapiens cDNA clone IMAGE:7487892 5', mRNA sequence [CX873203] |
| A_21_P0010318 | 0.010519957 | 4.96751 | lnc-AF165138.7.1-1 | LNCipedia lincRNA (lnc-AF165138.7.1-1), lincRNA [lnc-AF165138.7.1-1:1] |
| A_22_P00008458 | 0.016951011 | 3.5858786 |  |  |
| A_23_P93295 | 9.39E-04 | 10.728799 | OR12D2 | Homo sapiens olfactory receptor, family 12, subfamily D, member 2 (gene/pseudogene) (OR12D2), mRNA [NM_013936] |
| A_33_P3279715 | 0.019547656 | -5.1490793 | UHRF2 | Homo sapiens ubiquitin-like with PHD and ring finger domains 2, E3 ubiquitin protein ligase (UHRF2), transcript variant 1, mRNA [NM_152896] |
| A_22_P00018936 | 0.019260064 | 2.8945763 | lnc-VAX2-2 | DB448840 RIKEN full-length enriched human cDNA library, testis Homo sapiens cDNA clone H013038M12 5', mRNA sequence [DB448840] |
| A_21_P0007620 | 0.015121243 | 2.892628 | lnc-TXNRD1-1 | LNCipedia lincRNA (lnc-TXNRD1-1), lincRNA [lnc-TXNRD1-1:1] |
| A_23_P309381 | 0.012386669 | 2.5447092 | HIST2H2AA4 | Homo sapiens histone cluster 2, H2aa4 (HIST2H2AA4), mRNA [NM_001040874] |
| A_22_P00021229 | 0.015121243 | -2.2572694 | lnc-SHISA4-1 | Homo sapiens cDNA FLJ45279 fis, clone BRHIP3001338. [AK127212] |
| A_21_P0005152 | 0.018740008 | 2.411818 | lnc-GMDS-3 | LNCipedia lincRNA (lnc-GMDS-3), lincRNA [lnc-GMDS-3:2] |
| A_22_P00017244 | 0.019866189 | 2.7560134 |  | AGENCOURT_15668041 NCI_CGAP_St3 Homo sapiens cDNA clone IMAGE:30703667 5', mRNA sequence [CF595856] |
| A_21_P0007422 | 0.015664632 | 3.4885633 | lnc-RP11-890B15.2.1-2 | LNCipedia lincRNA (lnc-RP11-890B15.2.1-2), lincRNA [lnc-RP11-890B15.2.1-2:1] |
| A_21_P0011485 | 0.00605371 | 3.555445 | XLOC_l2_005503 | BROAD Institute lincRNA (XLOC_l2_005503), lincRNA [TCONS_l2_00010198] |
| A_21_P0007693 | 0.008795916 | 3.2574027 | lnc-IFLTD1-1 | LNCipedia lincRNA (lnc-IFLTD1-1), lincRNA [lnc-IFLTD1-1:1] |
| A_33_P3299665 | 0.011452953 | 2.0359776 | LOC100505530 | Homo sapiens uncharacterized LOC100505530 (LOC100505530), long non-coding RNA [NR_126057] |
| A_23_P377094 | 0.008821026 | 2.5854607 | PNMA3 | Homo sapiens paraneoplastic Ma antigen 3 (PNMA3), transcript variant 1, mRNA [NM_013364] |

**Supplementary Table 2** Enrichment analyses based on Gene Ontology using differentially expressed genes in Subclinical Acute Rejection vs Control specimens.

| **Category** | **Term** | **Count** | **%** | **P** | **GENES** |
| --- | --- | --- | --- | --- | --- |
| GO TERM: BIOLOGICAL PROCESS | detection of chemical stimulus involved in sensory perception of smell | 35 | 2,9 | 3,2E-5 | OR7A10, OR7G1, OR52J3, OR51H1, OR13H1, CGB3, OR2M3, HTR4, PIK3CG, OR5H14, GNGT2, FFAR2, MRGPRX4, OR5B3, OR8D2, OR56B4, GHSR, LHCGR, OR51G1, OR5F1, OR2L2, AVPR1A, OR10Q1, OR5V1, OR52R1, CCKBR, ADGRF4, OR4K14, VN1R5, OR4C16, OR4C15, OR52A5, OR6K6, OR10H4, OR5M3, MCHR1, OR1Q1, MC2R, ADGRG5, OR51S1, OR10D3, OR51B6, OR12D2, OR2B6, GPR12, OR10G2, OR5L1, OR1L3, HTR5A, OR10G6, CALM1, RAMP1, OR10G9 |
|  | G protein-coupled receptor signaling pathway | 53 | 4,4 | 3,1E-4 | OR7A10, OR7G1, OR52J3, OR51H1, OR13H1, CGB3, OR2M3, HTR4, PIK3CG, OR5H14, GNGT2, FFAR2, MRGPRX4, OR5B3, OR8D2, OR56B4, GHSR, LHCGR, OR51G1, OR5F1, OR2L2, AVPR1A, OR10Q1, OR5V1, OR52R1, CCKBR, ADGRF4, OR4K14, VN1R5, OR4C16, OR4C15, OR52A5, OR6K6, OR10H4, OR5M3, MCHR1, OR1Q1, MC2R, ADGRG5, OR51S1, OR10D3, OR51B6, OR12D2, OR2B6, GPR12, OR10G2, OR5L1, OR1L3, HTR5A, OR10G6, CALM1, RAMP1, OR10G9 |
|  |  |  |  |  |  |
|  |  |  |  |  |  |
| GO TERM:CELLULAR COMPONENT | plasma membrane | 205 | 16,8 | 3,8E-4 | OR7G1, SLC46A2, FRMPD1, OR2M3, SLA2, ANKRD20A1, HTR4, MYLK, EVA1A, TNFSF11, EPHB2, OR5B3, PRKACB, SCN1A, DYNC2H1, EPHA6, PRKCH, EPHA8, OR2L2, KCNK16, SLC6A11, SYTL5, TM4SF1, BIN2, OR4K14, OR4C16, OR4C15, PLPP1, SPTBN4, GYPB, PCDH15, LYPD2, LYPD4, CD79B, GHRHR, MC2R, OR10D3, KCNN3, GPIHBP1, TNFSF14, GPR12, SIGLEC12, LRRN4, PARVA, VASN, TREML1, XKR7, RAET1E, CALM1, SIGLEC9, KCNC1, OR10J5, GRIK4, SPPL3, PHEX, SMPD3, FFAR2, CCR3, APPL1, VAV3, OR5F1, INSRR, RHOH, AVPR1A, IL17RD, TMC3, NCR1, DCSTAMP, FCER2, OR5V1, F9, CCKBR, OR52R1, SLC29A4, TMEM106B, VN1R5, OR52A5, LILRA6, ADH1C, OR10H4, DCST1, LILRA4, DRP2, TM4SF20, PRAME, JAM2, LY6G6F, AOC2, OR10G2, YIPF3, ECEL1, OR1L3, KIR3DL1, SORBS2, LHFPL3, GRIN1, RGP1, OR10G6, RIMBP2, CTNNB1, RAMP1, OR10G9, UPK2, OR5H14, MPZ, SLC39A2, PDGFRA, GHSR, MYLIP, LHCGR, ADAM11, MUC3A, SLC9A2, S100A6, LY6D, KLRF2, ADAM7, XPR1, DPEP3, DSC3, BLK, OR6K6, GSG1L2, CACNA1B, MCHR1, SPTA1, OR1Q1, NPC1L1, ADGRG5, OR51S1, CDH23, OR51B6, KLRC1, OR2B6, SLC38A3, WNT1, KIR2DL5A, ATP8B3, SYT2, ZG16B, MUC19, POU2F3, LRFN1, SLCO1A2, ALPI, AMOTL1, SLC26A3, SDK2, SLC26A5, OR7A10, OR52J3, OR51H1, HSP90AB1, OR13H1, SLC2A5, TREM1, PIK3CG, UNC80, INSL6, RASGEF1A, ME1, ME3, IGLC1, MRGPRX4, OR8D2, OR56B4, OR51G1, PCDHGA5, ATP6AP1, HTR3E, GLP2R, HTR3C, OR10Q1, TIRAP, TBC1D3, CATSPERD, CDH12, ITGB1BP1, DNAJC9, DSG2, MS4A2, HCN4, CANT1, PON2, OR5M3, OPRL1, ATP1A3, TM2D3, AGPAT3, ADD2, TMEM202, SLIT1, OR12D2, S1PR5, TMEM30A, NOS2, OR5L1, HTR5A, ACVR2A, DYTN, CD2, KLF9, TRIM36, CD247, RHEBL1 |
|  |  |  |  |  |  |
|  |  |  |  |  |  |
| GO TERM MOLECULAR FUNCTION | olfactory receptor activity | 35 | 2,9 | 9,7E-5 | OR7A10, OR7G1, OR6K6, OR52J3, OR51H1, OR10J5, OR13H1, OR10H4, OR5M3, OR2M3, OR1Q1, OR5H14, OR51S1, OR10D3, OR51B6, OR12D2, OR5B3, OR8D2, OR2B6, OR56B4, OR51G1, OR5F1, OR10G2, OR5L1, OR2L2, OR1L3, OR10Q1, OR5V1, OR52R1, OR4K14, OR10G6, OR4C16, OR4C15, OR10G9, OR52A5 |
|  | G protein-coupled receptor activity | 47 | 3,9 | 1,1E-4 | OR7A10, OR7G1, OR6K6, OR52J3, OR51H1, OR10J5, OR13H1, OR10H4, OR5M3, OPRL1, OR2M3, MCHR1, GHRHR, OR1Q1, OR5H14, MC2R, ADGRG5, OR51S1, OR10D3, OR51B6, FFAR2, MRGPRX4, OR12D2, S1PR5, OR5B3, OR8D2, OR2B6, OR56B4, GHSR, OR51G1, OR5F1, GPR12, OR10G2, GLP2R, OR5L1, OR2L2, OR1L3, OR10Q1, OR5V1, OR52R1, ADGRF4, OR4K14, OR10G6, OR4C16, OR4C15, OR10G9, OR52A5 |

*Benjamini FDR corrected P-value

**Supplementary Table 3.** Top 10 upregulated genes ranked by fold change and lowest adjusted *p*-values

| **ProbeName** | **p (Corr)** | **Log2FC** | **GeneSymbol** | **Description** |
| --- | --- | --- | --- | --- |
| A_21_P0004623 | 7.75082E-06 | 4.0386887 | lnc-AL035696.1-3 | AF153341 winged helix/forkhead transcription factor {Homo sapiens} (exp=-1; wgp=0; cg=0), partial (6%) [THC2667051] |
| A_33_P3218491 | 7.75082E-06 | 2.440728 |  |  |
| A_33_P3424295 | 8.18199E-06 | 4.152437 | TRAF2 | Homo sapiens TNF receptor-associated factor 2 (TRAF2), mRNA [NM_021138] |
| A_23_P70468 | 8.7929E-06 | 8.944703 | OR2B6 | Homo sapiens olfactory receptor, family 2, subfamily B, member 6 (OR2B6), mRNA [NM_012367] |
| A_22_P00003289 | 8.7929E-06 | 6.0127616 |  | BX091468 Soares_testis_NHT Homo sapiens cDNA clone IMAGp998A141864 ; IMAGE:757909, mRNA sequence [BX091468] |
| A_21_P0006227 | 1.18933E-05 | 3.2266953 | lnc-STXBP1-2 | LNCipedia lincRNA (lnc-STXBP1-2), lincRNA [lnc-STXBP1-2:1] |
| A_22_P00010232 | 2.35218E-05 | 6.4700947 |  |  |
| A_23_P164596 | 4.67949E-05 | 16.52069 | SIGLEC12 | Homo sapiens sialic acid binding Ig-like lectin 12 (gene/pseudogene) (SIGLEC12), transcript variant 1, mRNA [NM_053003] |
| A_22_P00009823 | 4.67949E-05 | 7.81589 | lnc-METAP1-3 | Homo sapiens, clone IMAGE:5165147, mRNA. [BC038532] |
| A_22_P00023337 | 4.67949E-05 | 5.3977585 |  | PREDICTED: Homo sapiens uncharacterized LOC102723446 (LOC102723446), ncRNA [XR_426177] |

**Supplementary Table 4.** 25 most upregulated Cytokines secreted in the extracellular space

| Symbol | Entrez Gene Name | Agilent | Expr p-value | Expr Fold Change | Location |  | Type(s) |
| --- | --- | --- | --- | --- | --- | --- | --- |
| FGG | fibrinogen gamma chain | A_23_P148088 | 0.024 | 4.0 | Extracellular Space |  | other |
| WNT1 | Wnt family member 1 | A_23_P411157 | 0.013 | 3.8 | Extracellular Space |  | cytokine |
| PRAMEF11 | PRAME family member 11 | A_33_P3303594 | 0.035 | 3.6 | Extracellular Space |  | other |
| FGA | fibrinogen alpha chain | A_23_P375372 | 0.025 | 3.5 | Extracellular Space |  | other |
| CXCL10 | C-X-C motif chemokine ligand 10 | A_24_P303091 | 0.042 | 3.5 | Extracellular Space |  | cytokine |
| SMR3A | submaxillary gland androgen regulated protein 3A | A_23_P41365 | 0.017 | 3.4 | Extracellular Space |  | other |
| MZB1 | marginal zone B and B1 cell specific protein | A_33_P3351290 | 0.000 | 3.2 | Extracellular Space |  | other |
| LINGO2 | leucine rich repeat and Ig domain containing 2 | A_23_P157926 | 0.002 | 3.2 | Extracellular Space |  | other |
| ALOXE3 | arachidonate lipoxygenase 3 | A_24_P347880 | 0.011 | 3.0 | Extracellular Space |  | enzyme |
| DEFB4A/  DEFB4B | defensin beta 4A | A_23_P157628 | 0.032 | 3.0 | Extracellular Space |  | other |
| CRP | C-reactive protein | A_24_P342484 | 0.021 | 3.0 | Extracellular Space |  | other |
| CFP | complement factor properdin | A_23_P22444 | 0.001 | 2.9 | Extracellular Space |  | other |
| INSL3 | insulin like 3 | A_33_P3334828 | 0.005 | 2.8 | Extracellular Space |  | growth factor |
| HCRT | hypocretin neuropeptide precursor | A_33_P3306624 | 0.007 | 2.8 | Extracellular Space |  | other |
| LCN1 | lipocalin 1 | A_33_P3317790 | 0.007 | 2.8 | Extracellular Space |  | transporter |
| ITIH1 | inter-alpha-trypsin inhibitor heavy chain 1 | A_23_P18223 | 0.022 | 2.6 | Extracellular Space |  | other |
| DAND5 | DAN domain BMP antagonist family member 5 | A_23_P435636 | 0.016 | 2.6 | Extracellular Space |  | other |
| F13B | coagulation factor XIII B chain | A_33_P3397496 | 0.040 | 2.6 | Extracellular Space |  | enzyme |
| C17orf99 | chromosome 17 open reading frame 99 | A_33_P3384133 | 0.037 | 2.5 | Extracellular Space |  | other |
| CSPG5 | chondroitin sulfate proteoglycan 5 | A_24_P342944 | 0.006 | 2.5 | Extracellular Space |  | growth factor |
| FASLG | Fas ligand | A_23_P369815 | 0.007 | 2.5 | Extracellular Space |  | cytokine |
| CSH1/CSH2 | chorionic somatomammotropin hormone 1 | A_23_P207174 | 0.014 | 2.5 | Extracellular Space |  | other |
| OTOA | otoancorin | A_33_P3364308 | 0.036 | 2.4 | Extracellular Space |  | other |
| DEFB105A  /DEFB105B | defensin beta 105A | A_23_P380871 | 0.0115 | 2.4 | Extracellular Space |  | other |
| LIPM | lipase family member M | A_33_P3401422 | 0.00841 | 2.4 | Extracellular Space |  | enzyme |
